# Biocatalytic synthesis of ribonucleoside analogues using nucleoside transglycosylase-2†

**DOI:** 10.1039/d4sc07521h

**Published:** 2024-12-11

**Authors:** Admir Salihovic, Alex Ascham, Petja S. Rosenqvist, Andrea Taladriz-Sender, Paul A. Hoskisson, David R. W. Hodgson, Gideon Grogan, Glenn A. Burley

**Affiliations:** a Department of Pure & Applied Chemistry, University of Strathclyde 295 Cathedral Street Glasgow G1 1XL UK glenn.burley@strath.ac.uk; b Strathclyde Centre for Molecular Bioscience, University of Strathclyde Glasgow UK; c Department of Chemistry, University of York Heslington York YO10 5DD UK gideon.grogan@york.ac.uk; d Department of Chemistry, Durham University South Road Durham DH1 3LE UK; e Strathclyde Institute of Pharmacy & Biomedical Sciences, University of Strathclyde 161 Cathedral Street Glasgow G4 0RE UK

## Abstract

Ribonucleosides are essential building blocks used extensively in antiviral and oligonucleotide therapeutics. A major challenge in the further development of nucleoside analogues for therapeutic applications is access to scalable and environmentally sustainable synthetic strategies. This study uses the type II nucleoside 2′-deoxyribosyltransferase from *Lactobacillus leichmannii* (*Ll*NDT-2) to prepare a suite of ribonucleoside analogues using naturally-occurring uridine and cytidine sugar donors. Crystal structure and mutational analyses are used to define the substrate tolerance of the nucleobase exchange and the 2′-substituent of the nucleoside sugar donor. Nucleobase profiling identified acceptance of both purine and pyrimidine nucleobases. Finally, the scalability of the approach is showcased, enabling the preparation of ribonucleosides on millimolar scales. This biocatalytic strategy opens up opportunities to establish chemoenzymatic routes to prepare nucleoside analogues incorporating 2′ modifications that are of therapeutic importance.

## Abstract 2

Ribonucleosides are essential building blocks used extensively in antiviral and oligonucleotide therapeutics.

## Graphical abstract

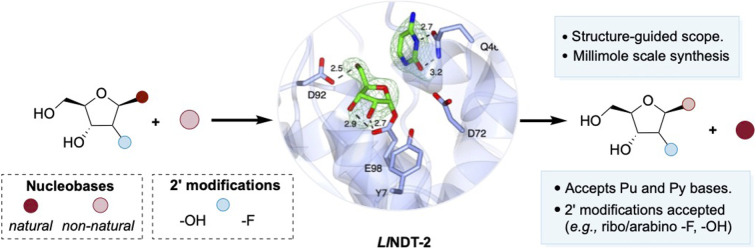

## Introduction

**Fig. 1:**
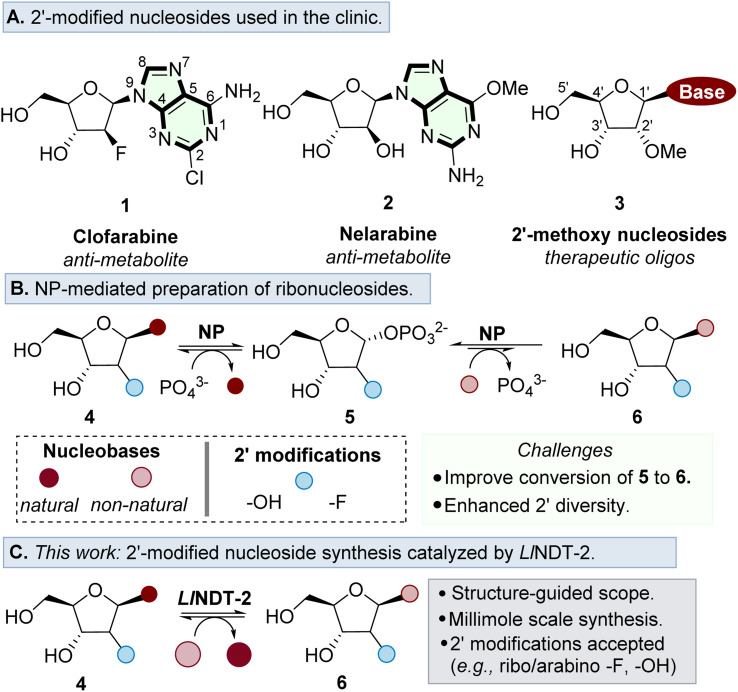
(A) Examples of prominent nucleoside analogues used as therapeutics. (B) Overview of the utility of NPs as biocatalysts for the preparation of ribonucleosides. (C) This work: establishing a structure-guided workflow to prepare ribonucleoside analogues catalysed by *Ll*NDT-2.

Ribonucleosides are essential building blocks used throughout nature. They serve as basic constituents of RNA, and are key motifs found in a variety of cofactors and secondary messengers.^1–3^ In addition, nucleoside analogues are used extensively in the development of antiviral and anti-cancer agents,^4–7^ as well as forming the key building blocks for the development of therapeutic oligonucleotides (Fig. 1A).^8^

A major challenge in the further development of nucleoside analogues for therapeutic applications is access to step efficient, environmentally sustainable and preferably convergent methods of synthesis.^9^ Whilst conventional chemical synthetic strategies typically require anhydrous conditions and toxic reagents to form the desired nucleosidic linkage,^10,11^ biocatalytic methods are performed in aqueous conditions and with superior stereoselectivity to form the desired β-anomer product.^12–14^

Nucleoside purine (PuNP) and pyrimidine (PyNP) phosphorylases have been the most prominent enzymes to prepare ribonucleosides,^15,16^ enabling the preparation of purine and pyrimidine products with substrate tolerance at the 2′ position (Fig. 1B).^17–21^ An enzymatic alternative to the use of NPs to prepare ribonucleoside analogues is to use nucleoside 2′-deoxyribosyltransferases (NDTs).^22,23^ These enzymes catalyse the formation of nucleoside analogues *via* an overall nucleobase exchange much akin to NPs. However, NDTs are mechanistically distinct, forming a covalent adduct within the active site, thus controlling stereoselective nucleophilic attack of the incoming nucleobase on the β face. The mechanistic divergence of NDTs negates issues associated with the undesirable buildup of the C1′ phosphate intermediate associated with NPs,^24^ which is thermodynamically favoured over the formation of the desired nucleoside product.^25,26^

Exploration of the substrate promiscuity of the nucleoside sugar donor of various NDTs has revealed a preference for 2′-deoxyribose donors over ribonucleoside analogues.^23,27–31^ More recent work by Tang *et al.* revealed that the wildtype thermophilic NDT derived from *Chroococcidiopsis thermalis* (*Ct*NDT) prefers 2′-deoxyribonucleosides as the corresponding sugar donor, in a process that is driven by a faster *k*_cat_ relative to a ribonucleoside donor.^32^ Previous crystal structure studies have been reported, emphasizing the deoxyribose acceptance of NDTs.^23,27,32,33^ At present, a defined substrate scope and structure analysis has not been conducted for *Ll*NDT-2 around the nucleobase and the 2′-position of the nucleoside donor.

In this study, we demonstrate enzymatic transglycosylation catalysed by *Ll*NDT-2 using a 2′-modified nucleoside sugar donor either in the *arabino* or *ribo* configuration (Fig. 1C). We define the mechanistic basis for substrate acceptance and highlight how the nature of the modification may interact with the active site of the enzyme. Finally, we demonstrate the potential of *Ll*NDT-2 to prepare high value nucleoside analogues on a 1 mmol scale.

## Results and discussion

### *Ll*NDT-2 catalyses transglycosylation of ribonucleoside donors

**Fig. 2:**
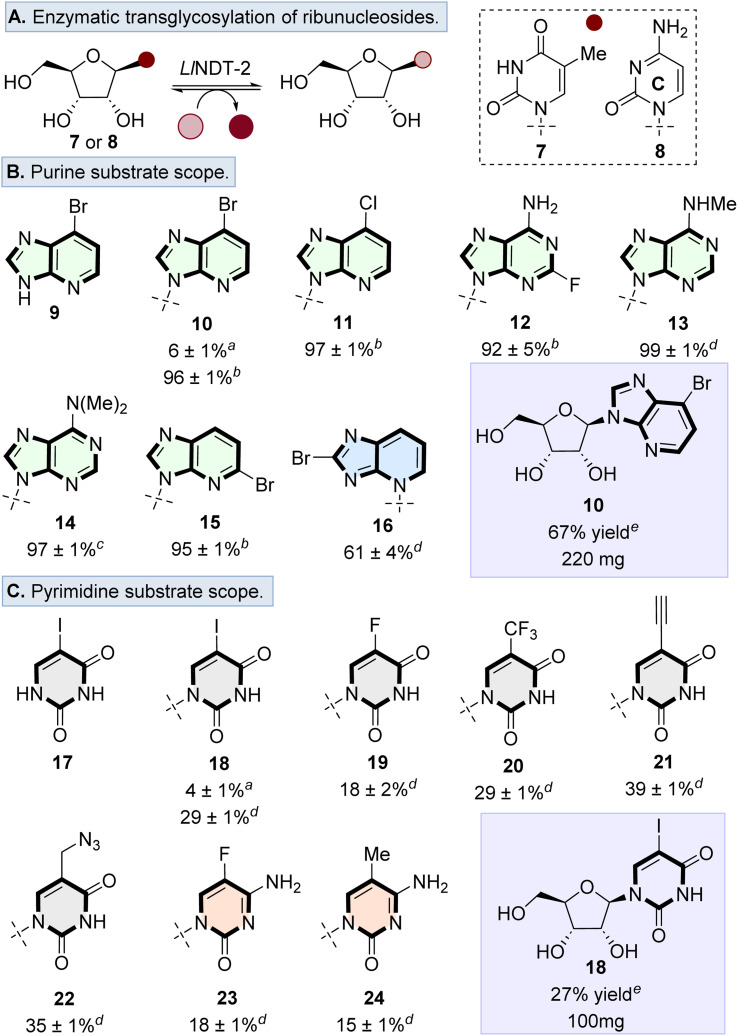
(A) Overview of the transglycosylation reaction to form ribonucleosides catalysed by *Ll*NDT-2. Substrate scope of transglycosylation using (B) purine, and (C) pyrimidine nucleobases. General reaction conditions: 7 or 8 (5–10 equiv.), nucleobase (1 equiv.), solvent (DMSO : H_2_O, 1 : 4), *Ll*NDT-2 (100 to 300 μg mL^−1^). Enzyme loading at ^*a*^ 2 μg mL^−1^, ^*b*^ 100 μg mL^−1^, ^*c*^ 200 μg mL^−1^, ^*d*^ 300 μg mL^−1^, and ^*e*^ 500 μg mL^−1^. For general purine nucleosides 10–16 and pyrimidine nucleosides 18, 20, 21 and 22, nucleoside donor 8 was used, for the formation of pyrimidine nucleosides 19, 23 and 24, nucleoside donor 7 was used. Reactions were investigated in triplicate and the % conversion was calculated by the ratio of the peak area of nucleobase to the peak area of the nucleoside product.

We first explored the potential of *Ll*NDT-2 to accept a ribonucleoside donor (*e.g.*, 7 or 8) and to catalyse transglycosylation using a range of non-natural nucleobase analogues (Fig. 2A). Although wildtype (WT) *Ll*NDT-2 has been reported to be specific for 2′-deoxyribonucleosides over ribonucleoside analogues,^27,34^ acceptance of 2′,3′-dideoxy analogues^25,27,34^ and 2′-fluoro-2′-deoxynucleosides was demonstrated.^30,31^ We therefore undertook a systematic exploration of the acceptance of ribonucleoside donors as a function of enzyme loading and nucleobase. Ribonucleosides 7 or 8 were used as representative sugar donors, with 9 and 17 used as the corresponding nucleobases (Fig. 2B and C). Using 2 μg mL^−1^ loading of *Ll*NDT-2 formed the desired nucleosides 10 and 18 with 6% and 4% conversion, respectively. However, when the enzyme loading was increased, conversion to 10 (100 μg mL^−1^) and 18 (300 μg mL^−1^) increased to 96% and 29%, respectively.

Based on the ability of WT-*Ll*NDT-2 to undergo nucleobase swapping with ribonucleoside donors, the nucleobase scope was surveyed using a range of purine and pyrimidine analogues. For the purine scope, substituents were tolerated in a variety of positions, resulting in conversion to the desired products in >80% for nucleosides 10–15. Only bulky substituents in the purine 8-position (*i.e.*, 16) resulted in the conversion dropping to 61% as well as forming a glycosidic bond at the N3 position rather than N9 as observed with nucleosides 10–15 (Fig. 2B).

Transglycosylation was also possible with pyrimidine analogues, tolerating modifications to the 5-position of both cytosine and uracil nucleobases (Fig. 2C). Conversions to the desired ribonucleosides 18–24 were lower than the observed conversions for purine analogues. Finally, we showed that the transglycosylation reaction to form ribonucleosides was scalable, by forming nucleosides 10 and 18 on a 1 mmol scale in 67% and 27% yield, respectively. Therefore, despite previous studies highlighting that WT-*Ll*NDT-2 ribonucleosides are poor substrates,^30,35,36^ this can be overcome by increasing the enzyme loading.

### Structural basis for the differences in acceptance of ribonucleoside *versus* 2′-deoxyribonucleoside donors

To gain further insight into how the presence of a 2′-OH substituent on the ribonucleoside donor influences substrate recognition and subsequent catalytic steps, structural studies were conducted on the wildtype *Ll*NDT-2 and a double mutant Y7F/D72N. These two mutations were chosen based on the enhanced acceptance of a ribonucleoside donor in an enzymatic transglycosylation reaction by an analogous double mutant of a type I NDT derived from *Trypanosoma brucei* (*Tb*NDT).^29^ The corresponding Y7F/D72N mutant of *Ll*NDT-2 showed inferior catalytic transglycosylation compared to the wildtype enzyme using a range of nucleoside donor and nucleobases (Fig. S4†).

**Fig. 3:**
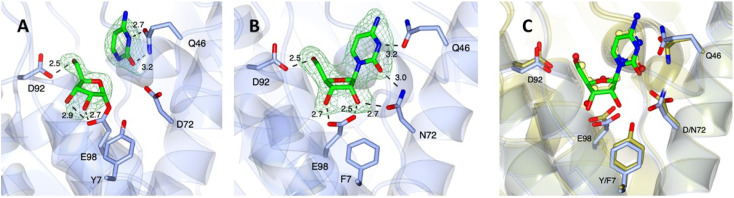
(A) Structure of WT-*Ll*NDT-2 complexed with cytidine 8, yielding an active site with the ribosylated enzyme and cytosine (PDB code 9GN2). (B) Structure of Y7F/D72N mutant of *Ll*NDT-2 complexed with cytidine, yielding intact mutant-8 complex (PDB code 9GN4). (C) Superimposition of Y7F/D72N mutant of *Ll*NDT-2 with the complex of WT-*Ll*NDT-2 with 2′-deoxycytidine (side-chain carbon atoms in gold; ligand in ball-and-stick format with carbon atoms in yellow; PDB code 9F09). Electron density maps are the *F*_o_ − *F*_c_ omit maps obtained before refinement of ligand atoms and at a level of 3*σ*. Selected active site-ligand interactions are illustrated with black dashed lines with distances in Å.

To explore the structural implications of the differences between the two enzymes, WT-*Ll*NDT-2 and the Y7F/D72N mutant were both crystallised using conditions determined for the WT-*Ll*NDT-2 enzyme previously.^33^ Complexes were obtained by soaking cytidine 8 into the crystals. All structures contained two molecules in the asymmetric unit, constituting one third of the hexamer routinely observed in solution. In the case of WT-*Ll*NDT-2, electron density in the omit maps obtained could be clearly modelled as the ribosylated intermediate (Fig. 3A), and the cleaved cytosine nucleobase, whereas in the Y7F/D72N structure cytidine 8 was intact (Fig. 3B), presumably reflective of its poorer *k*_cat_ as observed for *Ct*NDT.^32^

A comparison of the ribosylated WT-*Ll*NDT-2 structure with that of the 2′-deoxyribosylated structure previously obtained by our groups (9F08),^33^ shows only a small rotation of the ribose ring towards the side chain of D92, resulting in a slightly extended interaction of 2.9 Å between the 3′-OH and the OE2 atom of E98 compared to the 2′-deoxyribosylated complex (2.7 Å). By contrast, the ribosylated intermediate of *Ct*NDT reported by Tang and co-workers^32^ features the ribose flipped approximately 90° from this position, with the 2′-OH interacting with the side chain of D62 (D72 in *Ll*NDT-2), despite the conservation of most active site residues, although the authors report multiple conformations of the ligand in this complex, evidenced by ambiguous electron density.

A comparison of the Y7F/D72N-*Ll*NDT-2 and WT-*Ll*NDT-2 (9F09)^33^ complexes with cytidine and 2′-deoxycytidine respectively. Fig. 3C shows close agreement between the atom positions of the active site sidechains and ligands in each structure. These include those between the 5′-OH and D92, and the OE2 E98 carboxylate oxygen and the 3′-OH of (deoxy)ribose. However, the side chain of N72, which, like that of D72 in the WT-*Ll*NDT-2, forms an interaction with the O2 of cytosine, has different rotamer conformations in each monomer in the asymmetric unit, one of which permits two interactions with cytidine (Fig. 3B). In addition, in the Y7F/D72N structure, the interactions between the nucleobase of 8 and the side chain of Q46 are slightly different, with the interaction between the nitrogen of the amide and the O2 of 8 observed in the WT complex disrupted by a movement of the Q46 side chain.

Whereas in the WT-*Ll*NDT-2-deoxycytidine complex (9F09) the 3′-OH of 2′-deoxycytidine is bound only by the OE2 atom of the D98 carboxylate, in the Y7F/D72N–WT-*Ll*NDT-2 complex the 2′-OH and 3′-OH of the ribonucleoside interact with one carboxylate atom of E98 each. The 2′-OH also interacts with the side chain of N72 at a distance of 2.7 Å, but only in one rotameric orientation. The interaction between E98 and the 2′-OH alone would militate against the activity of E98 as a nucleophile for attack at C1′ of the substrate; additionally, the even lower activity of the Y7F/D72N mutant *versus* the WT-*Ll*NDT-2 for ribonucleosides would be compromised by the removal of the interaction between the phenol of Y7 and E98, which may suppress the ionisation of E98 for nucleophilic attack at C1.

### Broadening substrate scope: transglycosylation using 2′-modified nucleoside donors

**Fig. 4:**
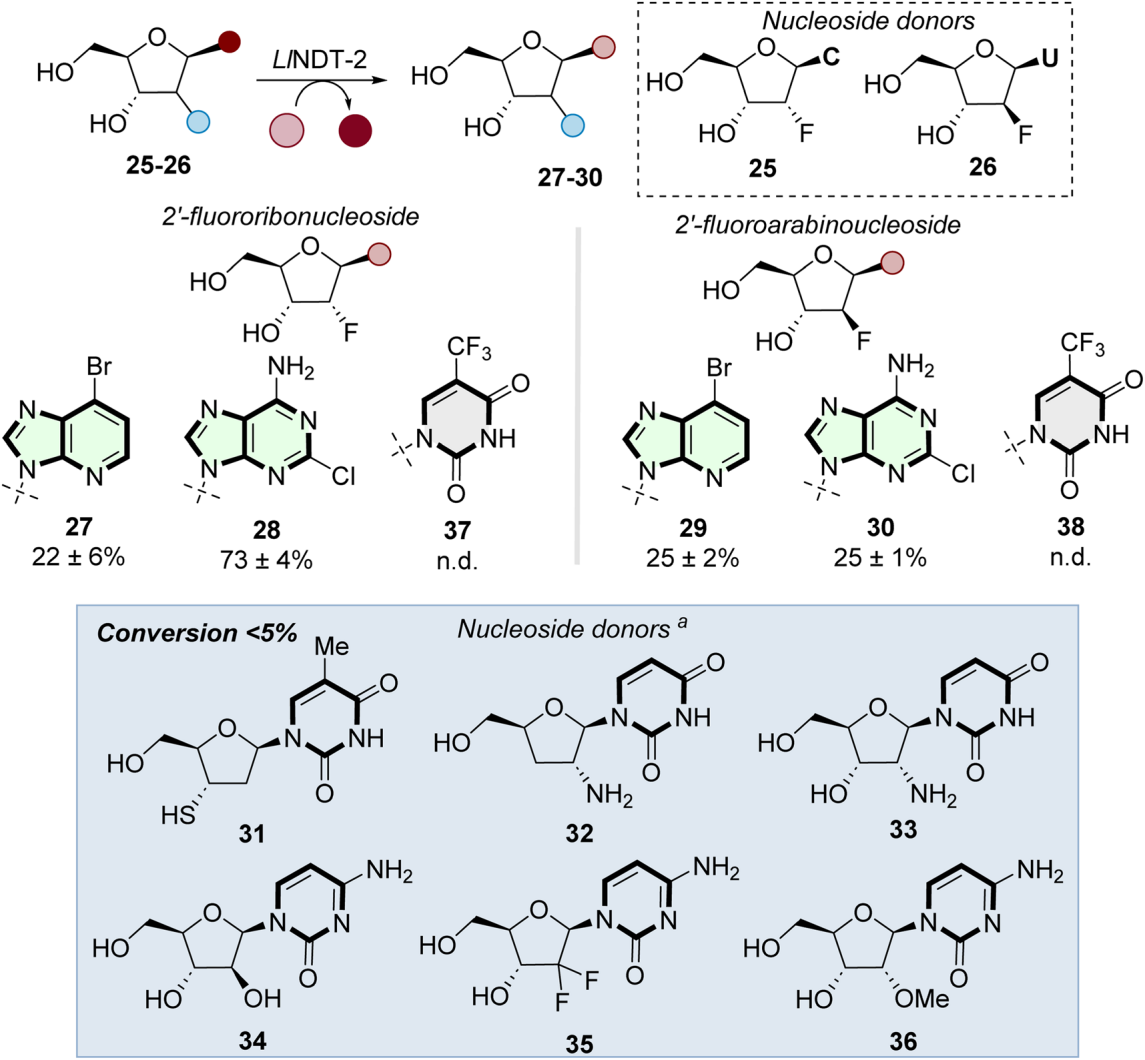
Scope of nucleoside donors in the enzymatic transglycosylation reaction catalyzed by *Ll*NDT-2. Reaction conditions: 25 (16 equiv.) or 26 (8 equiv.), nucleobase (1 equiv.), solvent (H_2_O : DMSO, 8 : 2), *Ll*NDT-2 (1 mg mL^−1^). ^*a*^ Nucleoside donors (10–20 equiv.) used with nucleobase 9 (1 equiv.) and *Ll*NDT-2 (1 mg mL^−1^). n.d. = not detected.

With the structural basis for the recognition of ribonucleosides established, we investigated the potential of WT-*Ll*NDT-2 to accept a range of modified nucleoside donor substrates (Fig. 4). Both 2′-fluoro-2′-deoxyribonucleosides 25 and 26 were accepted as sugar donor substrates. Proof of concept was demonstrated by the formation of ribonucleosides 27–28, arabinonucleosides 29–30 and also the formation of the antileukemia drug clofarabine (30) in 25% conversion.

Whilst existing industrial routes to prepare 30 involve a 4-step synthesis and can suffer from a number of process impurities found in the final product.^37,38^ This enzymatic route forms 30 in one step and isolated by a single chromatographic purification. Although 2′-fluorinated donors 25 and 26 were effective at preparing purine nucleosides 27–30, nucleobase swapping using 5-trifluoromethyluracil 37–38 6-dimethyladenine 39 (Fig. S1a and b†) did not result in conversions to the corresponding nucleosides above 5%. Our findings align with those reported by Yoo *et al.*,^31^ where a L59Q NDT mutant transglycosylated 2′-fluoro-2′-deoxyuridine to produce 2′-fluoro-2′-deoxyadenosine.

Finally, we investigated the substrate scope of nucleoside donors using a suite of analogues (31–36) modified at the 2′ and 3′ positions. In all cases, little to no conversion was observed when nucleobase 9 was used as acceptor, indicating limited tolerance of the sugar donor to modifications at these positions.

## Conclusions

In summary, we have demonstrated that WT-*Ll*NDT-2 catalyses the formation of ribonucleosides and 2′-fluoro-2′deoxynucleosides in a one-step and environmentally benign process. This expands the substrate repertoire of the nucleoside transferases and provides the basis for further development for the step-efficient preparation of 2′-modified nucleoside scaffolds with engineered variants, such as those found in the building blocks of therapeutic oligonucleotides^39^ and anti-viral/anti-cancer nucleosides.^14^

## Abbreviations

(*Ll*NDT-2): Type II nucleoside 2′-deoxyribosyltransferase from *Lactobacillus leichmannii*
(NP): Nucleoside phosphorylase

## Data availability

Data for this article, including the spectroscopic raw data for the characterisation of nucleoside products are available at https://pure.strath.ac.uk. The data supporting this article have been included as part of the ESI.† Crystallographic data for has been deposited in the PDB under 9F09 and can be obtained from https://www.rcsb.org/.

## Author contributions

The manuscript was written through contributions of all authors. All authors have given approval to the final version of the manuscript.

## Conflicts of interest

There are no conflicts to declare.

## Supplementary Material

SC-016-D4SC07521H-s001
