# Supplementary material for: Biocatalytic synthesis of ribonucleoside analogues using nucleoside transglycosylase-2
Source: Chem Sci. 2024 Dec 11;16(3):1302–7. doi: 10.1039/d4sc07521h (PMC11647913; doi:10.1039/d4sc07521h)
Supplement: SC-016-D4SC07521H-s001 [file SC-016-D4SC07521H-s001.pdf]

Supplementary Information (SI) for Chemical Science.
This journal is © The Royal Society of Chemistry 2024

Supplementary Information

Biocatalytic Synthesis of Ribonucleoside Analogues Using Nucleoside Transglycosylase-2

Admir Salihovic,[a,b] Alex Ascham,[c] Petja S. Rosenqvist,[d] Andrea Taladriz-Sender,[a,b] Paul A. Hoskisson,[e] David R.W. Hodgson,[d] Gideon Grogan,[c]* Glenn A. Burley[a,b]*

[a] Department of Pure & Applied Chemistry, University of Strathclyde, 295 Cathedral Street, Glasgow, United Kingdom G1 1XL. [b] Strathclyde Centre for Molecular Bioscience, University of Strathclyde. [c] Department of Chemistry, University of York, Heslington, York, United Kingdom, YO10 5DD. [d] Department of Chemistry, Durham University, South Road, Durham, United Kingdom, DH1 3LE.[e] Strathclyde Institute of Pharmacy & Biomedical Sciences, University of Strathclyde, 161 Cathedral Street, Glasgow, G4 0RE, United Kingdom.

1. General Information S2-S3
    1.1 Reagents and solvents
    1.2 NMR spectroscopy
    1.3 LC-MS
    1.4 HPLC
2. General experimental techniques and procedures S3-S7
    2.1 Preparation of stock solutions
    2.2 General procedures
    2.3 Analytical RP-HPLC method parameters
    2.4 RP-HPLC semi preparatory method parameters
3. Reaction scope of transglycosylation using ribonucleoside analogues S7-S23
    3.1 Characterisation of scaled up nucleosides
    3.2 Characterisation of nucleosides prepared on a smaller scale
    3.3 Characterisation of 2' Fluoro Nucleoside Analogues
4. Protein Expression and Crystallisation S23-S30
    4.1 Expression
    4.2 Purification of NDT-WT
    4.3 Purification of NDT-Y7F/D72N
    4.4 Crystallisation
    4.5 Data Collection and Refinement
5. Mutant NDT-Y7F/D72N transglycosylation conversions S30

5.1 Ribose transglycosylations using Mutant NDT-Y7F/D72N

6. Synthesis of 2' and 3' modified nucleosides for substrate scope transglycosylation investigations S31-S37
    6.1 Synthesis of 2',3' -dideoxy-3'-thiothymidine (31)
    6.2 Synthesis of 2'-amino-2',3'-dideoxyuridine (32)
    6.3 Synthesis of 2'-amino-2'-deoxyuridine (33)

7. Appendix S37 – S117

8. References S117 – S178

1. General information

1.1 Reagents and Solvents

All reagents and solvents were used as supplied from commercial sources and used without any further purification unless otherwise specified. Solvents were all HPLC grade and used without any further purification, unless otherwise specified. Thin layer chromatography (TLC) was performed using Merck silica plates coated with fluorescent indicator UV254. TLC plates were analysed under 254 nm UV light or developed in p-Anisaldehyde. Normal-phase column chromatography was carried out using Fluorochem Silicagel 60 Å 40-63 µm. Normal phase auto column chromatography was carried out using Silicycle silicagel on an interchim puriflash XS520 plus.

1.2 NMR Spectroscopy

NMR spectroscopy was carried out using a Bruker 400 UltraShieldTM "Avance I" spectrometer and Bruker Avance III-HD-400 and Varian VNMRS-600 spectrometer. All chemical shifts (δ) in $CDCl_3$ were referenced at 7.26 ppm (^1H) and 77.16 ppm (^{13}C), in $(CD_3)_2SO$ at 2.50 ppm (^1H) and 39.52 ppm (^{13}C), in CD_3CN at 1.94 ppm (^1H) and 118.26 ppm (^{13}C), in D_2O at 4.79 ppm (^1H), and in CD_3OD at 3.31 ppm (^1H) and 49.00 ppm (^{13}C). Chemical shifts are reported in parts per million (ppm) and coupling constants are quoted in hertz (Hz). Abbreviations for splitting patterns are s (singlet), d (doublet), t (triplet), q (quartet) and m (multiplet). App (apparent) denotes signals in which similar *J* values have resulted in false equivalence. All NMR data was processed using MestRenova 11.0.3 software.

1.3 Liquid Chromatography-Mass Spectrometry (LC-MS)

LC-MS was carried out on an Agilent 1200 series HPLC instrument in conjunction with an Agilent Quadrupole mass detector 9 (HPLC Agilent Technologies 6130 Quadrupole), using an agilent Infinity Lab Poroshell 120, 4.6 x 100 mm, 2.7 μ C18 column. A combination of electrospray ionization (ESI) and atmospheric pressure chemical ionization (APCI) was used in all cases (MM-ES+APCI). The solvent system used was Acetonitrile/Water with 5 mM Ammonium Acetate buffer pH 7.0.

1.4 High-Pressure Liquid Chromatography (HPLC)

HPLC analysis was performed on a Dionex Ultimate 300 instrument utilising the VWD3400 variable wavelength detector. Analytical reversed-phase HPLC (RP-HPLC) was carried out on a Shimadzu Prominence instrument utilising a PDA Detector scanning from 190-600 nm. Semi-preparative RP-HPLC purification was carried out on a Dionex Ultimate 3000 series instrument using a 150 x 21.2 mm Kinetex 5 μm C18 column.

2. General experimental techniques and procedures

2.1 Preparation of stock solutions

100 mM Phosphate buffer solution pH 6

$Na_2HPO_4 \cdot 7H_2O$ (1.84 g) and $NaH_2PO_4 \cdot H_2O$ (5.96 g) were dissolved in 500 mL of mQ H_2O, adjusted using 0.2 mL of 1M HCl obtaining a final pH of 6.

10 mM Phosphate buffer solution pH 7

$Na_2HPO_4 \cdot 7H_2O$ (1.549 g) and $NaH_2PO_4 \cdot H_2O$ (0.582 g) were dissolved in 1 L of mQ H_2O, adjusted using 0.2 mL of 1M HCl obtaining a final pH of 7.

Preparation of stock solutions of nucleoside donors

5-Methyluridine **7** (775 mg) was dissolved in 30 mL of mQ H_2O to prepare a 100 mM solution. Solution was shaken by hand for 1 min to ensure full solubility of 5-methyluridine.
Cytidine **8** (730 mg) was dissolved in 30 mL of mQ H_2O to prepare a 100 mM solution. Solution was shaken for 1 min to ensure full solubility of cytidine.
Sugar donor stock solutions were stored at rt.

Preparation of stock solutions of nucleobase acceptors

As an example, 26 mg of 6-bromo-deazapurine were dissolved by sonication in 2626 µL of chosen solvent to form a 50 mM solution. Nucleobase solution was stored at rt.

Preparation of stock solutions of *Ll*NDT

Lactobacillus Leichmanii NDT (*Ll*NDT, N2665 Sigma-Aldrich, expressed in *E. coli*) stock solution was prepared by dissolving 0.921 mg of powder enzyme in 0.921 µL of 100 mM Na_2HPO_4 pH 7 and 20% glycerol (1 mg/mL). Solution stored in −18 ˚C.

2.2 General Procedures

General Enzymatic Nucleoside Transglycosylation Procedure

A 50 mM nucleobase stock solution in mQ H_2O (10.0 µmol, 200 µL, 1 equiv) was mixed with a 100 mM 5-methyluridine or cytidine solution dissolved in mQ H_2O (50.0 µmol, 500 µL, 5 equiv) Next, 100 µL of NDT stock solution (0.1 mg/mL) were added as well as 200 µL extra of mQ H_2O to afford a final reaction volume of 1 mL. The resulting reaction mixture was shaken at 950 rpm at 40 °C for 24 h. Then, an aliquot of 100 µL of reaction mixture was transferred to a 1 mL HPLC vial, quenched with 100 µL of HFIP or Proteinase K (5 mg/mL) and diluted with 800 µL of 100 mM Na_2HPO_4 buffer. The crude was analysed by analytical RP-HPLC, and conversion of the target nucleoside was calculated by monitoring the ratio of the peak area of starting material to peak area of product. Reactions were carried out in triplicate and conversions were expressed as the average with the corresponding standard deviation.

2.3 Analytical RP-HPLC method parameters

Method A

Column specification: Luna C18 Polar Omega 3 µ (100 x 4.6 mm)
Column temperature: 40 °C
Mobile phase A: 0.1% v/v TFA in water
Mobile phase B: 0.1% v/v TFA in Acetonitrile
Flow rate 1.5 mL/min
Gradient profile:

Time (min)	% A	% B
0	99	1
0.5	99	1
4	88	12
6.5	50	50
7	5	95
8	5	95
9	99	1
11.30	99	1

Detection wavelength of 254 nm.

Method B

Column specification: Luna C18 Polar Omega 3 µ (100 x 4.6 mm)

Column temperature: 40 °C

Mobile phase A: 0.1% v/v TFA in water

Mobile phase D: Acetonitrile

Flow rate 1.5 mL/min

Gradient profile:

Time (min)	% A	% D
0	95	5
8	40	60
8.5	5	95
11	5	95
11.5	95	5
14	95	5

Detection wavelength of 254 nm.

Method C

Column specification: Phenomenex Luna C18 3 µ phenyl-hexyl (150 x 4.6 mm)

Column temperature: 40 °C

Mobile phase A: 0.1% v/v TFA in water

Mobile phase B: 0.1% v/v TFA in Acetonitrile

Flow rate 1.2 mL/min

Gradient profile:

Time (min)	% A	% B
0	99	1

8	70	30
9	5	95
11	5	95
11	99	1
13	99	1

Detection wavelength of 254 nm.

2.4 RP-HPLC semi preparatory method parameters:

Method D

Column specification: Kinetex 5 µm XB-C18 100 Å, 150 x 21.2 mm

Column temperature: 40 °C

Mobile phase A: Water

Mobile phase B: Acetonitrile

Flow rate: 12 mL/min

Time (min)	% A	% B
0	95	5
18	82	18
19	5	95
23	5	95
24	95	5
28	95	5

Detection wavelength of 254 nm.

3. Ribose scope characterisation

3.1 Scaled up preparation of ribonucleosides

6-bromo-1-deaza-ribosyl-purine (10)

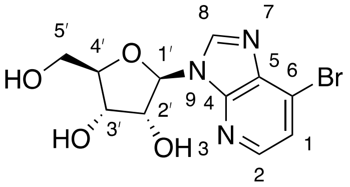

Cytidine **8** (1.22 g, 5.0 mmol, 5 equiv) and 6-bromo-deazapurine **9** (198 mg, 1.0 mmol, 1 equiv) were dissolved in 5 mL of mQ H_2O. The solution was left stirring for an hour at 40 °C followed by addition of 5 mL of *LINDT* (from a 1 mg/mL stock solution). The reaction was stirred for 72 h at 40 °C. The crude residue was concentrated *in vacuo*, dry loaded onto silica and purified

by flash column chromatography (silica gel, 0-20% CH_2Cl_2/MeOH) to obtain the nucleoside **10** in 67% yield as a brown solid (220 mg, 0.67 mmol).

^{1}H NMR (400 MHz, $(CD_3)_2SO$, ppm) δ 8.82 (s, 1H, H^8), 8.25 (d, J = 5.2 Hz, 1H, H^2), 7.63 (d, J = 5.2 Hz, 1H, H^1), 6.06 (d, J = 5.6 Hz, 1H, $H^{1'}$), 5.50 (d, J = 6.0 Hz, 1H, O$H^{2'}$), 5.20 (d, J = 5.0 Hz, 1H, O$H^{3'}$), 5.14 (dd, J = 6.2, 5.0 Hz, 1H, O$H^{5'}$), 4.63 (*app* q, J = 5.6 Hz, 1H, $H^{2'}$), 4.19 (*app* td, J = 4.9, 3.7 Hz, 1H, $H^{3'}$), 3.98 (*app* q, J = 3.8 Hz, 1H, $H^{4'}$), 3.70 (ddd, J = 12.0, 5.0, 3.9 Hz, 1H, $H^{5'}$ or $H^{5''}$), 3.58 (ddd, J = 12.0, 6.2, 3.9 Hz, 1H $H^{5'}$ or $H^{5''}$)

^{13}C{^{1}H}-NMR (101 MHz, $(CD_3)_2SO$, ppm) δ 146.4(C^4), 144.6(C^8), 144.4(C^2), 134.8(C^5), 123.2(C^6), 121.8(C^1), 87.9($C^{1'}$), 85.5($C^{4'}$), 73.6($C^{2'}$), 70.3($C^{3'}$), 61.3($C^{5'}$).

RP-HPLC (Method A): t_R = 6.09 min, 75% conversion.

HRMS (ESI) *m/z*: $[M+Na]^+$ calcd for $C_{11}H_{12}{}^{79}BrN_3O_4Na$ 351.9903; found 351.9906.

5-iodo-uridine (18)

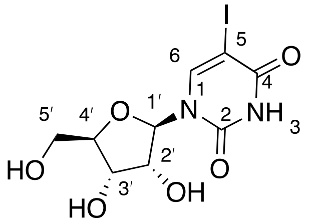

Cytidine **7** (2.43 g, 10.0 mmol, 10 equiv) and 5-iodouracil **17** (238 mg, 1.0 mmol, 1 equiv) were dissolved in 5 mL of mQ H_2O. The solution was left stirring for an hour at 40 °C followed by addition of 5 mL of *LINDT* (from a 1 mg/mL stock solution). The reaction was stirred for 72 h at 40 °C. The crude residue was concentrated *in vacuo*, dry loaded onto silica and purified by flash column chromatography (silica gel, 0-30% CH_2Cl_2/MeOH) to obtain the nucleoside **18** in 27% yield as a white solid (100 mg, 0.27 mmol).

^{1}H NMR (400 MHz, $(CD_3)_2SO$, ppm) δ 11.67 (s, 1H, NH), 8.48 (s, 1H, H^6), 5.73 (d, J = 4.6 Hz, 1H, $H^{1'}$), 5.40 (d, J = 5.4 Hz, 1H, O$H^{2'}$), 5.25 (t, J = 4.7 Hz, 1H, O$H^{5'}$), 5.06 (d, J = 5.3 Hz, 1H, O$H^{3'}$), 4.04 (*app* q, J = 5.0 Hz, 1H, $H^{2'}$), 3.99 (*app* q, J = 4.9 Hz, 1H $H^{3'}$), 3.87 (*app* dt, J = 4.9, 2.8 Hz, 1H, $H^{4'}$), 3.68 (ddd, J = 12.0, 4.8, 2.9 Hz, 1H $H^{5'}$ or $H^{5''}$), 3.57 (ddd, J = 12.0, 4.7, 2.7 Hz, 1H $H^{5'}$ or $H^{5''}$).

^{13}C{^{1}H}-NMR (101 MHz, $(CD_3)_2SO$, ppm) δ 160.4(C^4), 150.3(C^5), 145.1(C^6), 88.3($C^{1'}$), 84.7($C^{4'}$), 73.9($C^{2'}$), 69.3($C^{3'}$), 69.2 (C^2), 60.2($C^{5'}$).

NMR values in agreement with literature.[1]

RP-HPLC (Method A): t_R = 3.49 min, 31 % conversion.

HRMS (ESI) *m/z*: $[M+Na]^+$ calcd for $C_9H_{11}IN_2O_6Na$ 392.9554; found, 392.9545.

3.2 Small scale

6-bromo-1-deaza-ribosyl-purine (10)

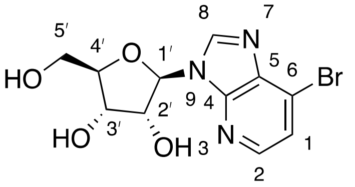

Prepared using the general enzymatic nucleoside transglycosylation procedure A. A 50 mM nucleobase stock solution (15 mg dissolved in 1515 µL) in DMSO (10.0 µmol, 200 µL, 1 equiv) was mixed with a 100 mM cytidine solution (243 mg in 10 mL), which was dissolved in mQ H_2O (50.0 µmol, 500 µL, 5 equiv). Next, 100 µL of NDT stock solution (0.1 mg/mL) was added alongside 200 µL of mQ H_2O to afford a final volume of 1 mL and the resulting reaction mixture was shaken at 950 rpm at 40 °C for 24 h. The crude reaction mixture was purified by preparative HPLC (method D) affording the target nucleoside **10**.

RP-HPLC (Method A): t_R = 6.09 min, 96% conversion (std = 1).

Characterisation of the target nucleoside was confirmed through ^1H NMR in comparison with the large-scale characterisation.

6-chloro-1-deaza-ribosyl-purine (11)

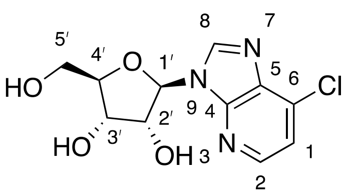

Prepared using the general enzymatic nucleoside transglycosylation procedure A. A 50 mM nucleobase stock solution (9.6 mg dissolved in 1250 µL) in mQ H_2O (10.0 µmol, 200 µL, 1 equiv) was mixed with a 100 mM cytidine solution (243 mg in 10 mL), which was dissolved in mQ H_2O (50.0 µmol, 500 µL, 5 equiv). Next, 100 µL of NDT stock solution (0.1 mg/mL) was added alongside 200 µL of mQ H_2O to afford a final volume of 1 mL and the resulting reaction

mixture was shaken at 950 rpm at 40 °C for 24 h. The crude reaction mixture was purified by preparative HPLC (method D) affording the target nucleoside **11**.

^{1}H NMR (400 MHz, $(CD_3)_2SO$, ppm) δ 8.82 (s, 1H, *H^8*), 8.35 (d, *J* = 5.2 Hz, 1H, *H^1 or H^2*), 7.51 (d, *J* = 5.3 Hz, 1H, *H^1 or H^2*), 6.07 (d, *J* = 5.6 Hz, 1H, *$H^{1'}$*), 5.50 (d, *J* = 5.9 Hz, 1H, O*$H^{2'}$*), 5.21 (d, *J* = 5.0 Hz, 1H, O*$H^{3'}$*), 5.15 (dd, *J* = 6.2, 5.0 Hz, 1H, O*$H^{5'}$*), 4.63 (*app* q, *J* = 5.4 Hz, 1H, *$H^{2'}$*), 4.19 (*app* q, *J* = 4.7 Hz, 1H, *$H^{3'}$*), 3.99 (*app* q, *J* = 3.8 Hz, 1H, *$H^{4'}$*), 3.70 (ddd, *J* = 12.0, 5.0, 3.9 Hz, 1H, *$H^{5'}$ or $H^{5''}$*), 3.58 (ddd, *J* = 12.0, 6.1, 3.9 Hz, 1H, *$H^{5'}$ or $H^{5''}$*).

NMR in agreement with literature.[2]

^{13}C{^{1}H}-NMR (101 MHz, $(CD_3)_2SO$, ppm) δ 147.3(*C^4*), 144.7(*C^2* or *C^1*), 144.6(*C^8*), 133.3(*C^5* or *C^6*), 133.0(*C^5* or *C^6*), 118.8(*C^2* or *C^1*), 87.8(*$C^{1'}$*), 85.6(*$C^{4'}$*), 73.6(*$C^{2'}$*), 70.3(*$C^{3'}$*), 61.3(*$C^{5'}$*).

RP-HPLC (Method A): t_R = 5.86 min, 97% conversion (std = 1).

HRMS (ESI) *m/z*: $[M+Na]^+$ calcd for $C_{11}H_{12}ClN_3O_4Na$ 308.0409; found 308.0406.

2-fluoro-ribosyl-adenosine (12)

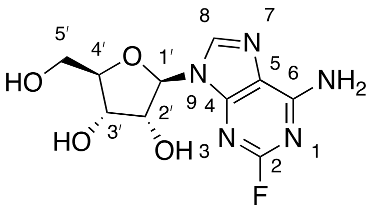

Prepared using the general enzymatic nucleoside transglycosylation procedure A. A 50 mM nucleobase stock solution (10 mg dissolved in 1306 µL) in mQ H_2O (10.0 µmol, 200 µL, 1 equiv) was mixed with a 100 mM cytidine solution (243 mg in 10 mL), which was dissolved in mQ H_2O (50.0 µmol, 500 µL, 5 equiv). Next, 100 µL of NDT stock solution (0.1 mg/mL) was added alongside 200 uL of mQ H_2O to afford a final volume of 1 mL and the resulting reaction mixture was shaken at 950 rpm at 40 °C for 24 h. The crude reaction mixture was purified by preparative HPLC (method D) affording the target nucleoside (**12**).

^{1}H NMR (400 MHz, $(CD_3)_2SO$, ppm) δ 8.35 (s, 1H, *C^8*), 7.86 (s, 2H, *NH_2*), 5.79 (d, *J* = 5.9 Hz, 1H, *$H^{1'}$*), 5.47 (dd, *J* = 6.1, 3.0 Hz, 1H, O*$H^{2'}$*), 5.19 (dd, *J* = 4.9, 3.0 Hz, 1H, O*$H^{3'}$*), 5.05 (t, *J* = 5.6 Hz, 1H, O*$H^{5'}$*), 4.52 (*app* q, *J* = 5.6 Hz, 1H, *$H^{2'}$*), 4.17 – 4.09 (m, 1H, *$H^{3'}$*), 3.94 (*app* q, *J* = 3.9 Hz, 1H, *$H^{4'}$*), 3.67 (*app* dt, *J* = 12.0, 4.3 Hz, 1H, *$H^{5'}$ or $H^{5''}$*), 3.55 (ddd, *J* = 11.9, 6.1, 4.0 Hz, 1H *$H^{5'}$ or $H^{5''}$*).

$^{13}C\{^1H\}$-NMR (101 MHz, $(CD_3)_2SO$, ppm) δ 158.6(d, *J* = 180.5, C^2), 157.62(C^6), 150.55(d, *J* = 20.0 Hz, C^4), 139.9(C^8), 117.5(C^5), 87.4($C^{1'}$), 85.6($C^{4'}$), 73.5($C^{2'}$), 70.3($C^{3'}$), 61.3($C^{5'}$). 146.4(C^4), 144.4(C^8), 134.7(C^5), 130.7(C^6), 122.2(C^1), 87.2($C^{1'}$), 85.6($C^{4'}$), 73.5($C^{2'}$), 70.4($C^{3'}$), 61.3($C^{5'}$)

$^{19}F\{^1H\}$- NMR (376 MHz, $(CD_3)_2SO$, ppm) δ −52.1.

NMR values in agreement with literature.[5]

RP-HPLC (Method A): t_R = 4.20 min, 92% conversion (std = 5).

HRMS (ESI) *m/z*: $[M+Na]^+$ calcd for $C_{10}H_{12}FN_5O_4Na$ 308.0766; found 308.0762.

N-6-methyl-adenosine (13)

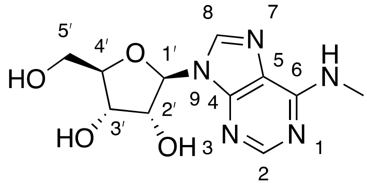

Prepared using the general enzymatic nucleoside transglycosylation procedure A. A 50 mM nucleobase stock solution (20 mg dissolved in 2682 µL) in mQ H_2O (10.0 µmol, 200 µL, 1 equiv) was mixed with a 200 mM cytidine solution (243 mg in 5 mL), which was dissolved in mQ H_2O (100.0 µmol, 500 µL, 10 equiv). Next, 300 µL of NDT stock solution (0.3 mg/mL) was added to afford a final volume of 1 mL and the resulting reaction mixture was shaken at 950 rpm at 40 °C for 24 h. The crude reaction mixture was purified by preparative HPLC (method D) affording the target nucleoside (**13**).

^1H NMR (400 MHz, $(CD_3)_2SO$, ppm) δ 8.34 (s, 1H, H^8), 8.23 (s, 1H, H^2), 7.81 (s, 1H, *NH*), 5.89 (d, *J* = 6.2 Hz, 1H, $H^{1'}$), 5.44 – 5.39 (m, 2H, $OH^{2'}$ and $OH^{5'}$), 5.20 (s, 1H, $OH^{3'}$), 4.61 (dd, *J* = 6.2, 4.9 Hz, 1H, $H^{2'}$), 4.15 (dd, *J* = 5.0, 3.0 Hz, 1H, $H^{3'}$), 3.97 (*app* q, *J* = 3.4 Hz, 1H, $H^{4'}$), 3.68 (*app* dd, *J* = 12.1, 3.6 Hz, 1H, $H^{5'}$ *or* $H^{5''}$), 3.56 (*app* d, *J* = 11.0 Hz, 1H, $H^{5'}$ *or* $H^{5''}$), 2.97 (s, 3H, $NHCH_3$).

$^{13}C\{^1H\}$-NMR (101 MHz, $(CD_3)_2SO$, ppm) δ 155.1(C^6), 152.4(C^2), 148.1(C^4), 139.6(C^8), 119.9(C^5), 87.9($C^{1'}$), 85.9($C^{4'}$), 73.5($C^{2'}$), 70.6($C^{3'}$), 61.6($C^{5'}$), 26.9(CH_3).

NMR values in agreement with literature.[4]

RP-HPLC (Method A): t_R = 3.04 min, 99% conversion (std = 1).

HRMS (ESI) *m/z*: $[M+H]^+$ calcd for $C_{11}H_{16}N_5O_4$ 282.1197; found 282.1194.

6-dimethylamino-adenosine (14)

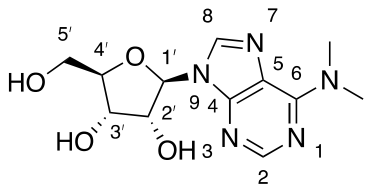

Prepared using the general enzymatic nucleoside transglycosylation procedure A. A 50 mM nucleobase stock solution (12 mg dissolved in 1471 µL) in mQ H_2O (10.0 µmol, 200 µL, 1 equiv) was mixed with a 100 mM cytidine solution (243 mg in 10 mL), which was dissolved in mQ H_2O (50.0 µmol, 500 µL, 5 equiv). Next, 200 µL of NDT stock solution (0.2 mg/mL) was added alongside 100 uL of mQ H_2O to afford a final volume of 1 mL and the resulting reaction mixture was shaken at 950 rpm at 40 °C for 24 h. The crude reaction mixture was purified by preparative HPLC (method D) affording the target nucleoside (**14)**.

^{1}H NMR (400 MHz, $(CD_3)_2SO$*,* ppm) δ 8.33 (s, 1H, H^2 or H^8), 8.21 (s, 1H, H^2 or H^8), 5.92 (d, *J* = 5.9 Hz, 1H, $H^{1'}$), 5.35 - 5.29 (m, 1H, $OH^{2'}$), 5.22 (dd, *J* = 6.8, 4.7 Hz, 1H $OH^{5'}$), 5.05 (d, *J* = 4.5 Hz, 1H, $OH^{3'}$), 4.58 (*app* q, *J* = 5.1 Hz, 1H, $H^{2'}$), 4.20 - 4.13 (*m*, 1H, $H^{3'}$), 3.97 (*app* q, *J* = 3.6 Hz, 1H, $H^{4'}$), 3.68 (ddd, *J* = 12.1, 4.5, 3.6 Hz*,* 1H, $H^{5'}$ *or* $H^{5''}$), 3.57 (ddd, *J* = 12.0, 6.7, 3.8 Hz, 1H, $H^{5'}$ *or* $H^{5''}$), 3.46 *(*s*, 6H,* CH_3*)*.

^{13}C{^{1}H}-NMR (101 MHz, $(CD_3)_2SO$, ppm) δ 154.3(C^4 or C^5 or C^6), 151.7(C^2 or C^8), 149.9 (C^4 or C^5 or C^6), 138.6(C^2 or C^8), 119.8(C^4 or C^5 or C^6), 87.8($C^{1'}$), 85.7($C^{4'}$), 73.5($C^{2'}$), 70.5($C^{3'}$), 61.5*(*$C^{5'}$), 37.8 (NCH_3CH_3).

NMR values in agreement with literature.[3]

RP-HPLC (Method A): t_R = 4.2 min, 97% conversion (std = 1).

HRMS (ESI) *m/z*: $[M+H]^+$ calcd for $C_{12}H_{18}N_5O_4$ 296.1353; found 296.1350.

2-bromo-1-deaza-ribosyl-purine (15)

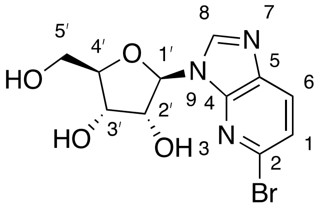

Prepared using the general enzymatic nucleoside transglycosylation procedure A. A 50 mM nucleobase stock solution (14.2 mg dissolved in 1434 µL) in mQ H_2O (10.0 µmol, 200 µL, 1 equiv) was mixed with a 100 mM cytidine solution (243 mg in 10 mL), which was dissolved in mQ H_2O (50.0 µmol, 500 µL, 5 equiv). Next, 100 µL of NDT stock solution (0.1 mg/mL) was added alongside 200 uL of mQ H_2O to afford a final volume of 1 mL and the resulting reaction mixture was shaken at 950 rpm at 40 °C for 24 h. The crude reaction mixture was purified by preparative HPLC (method D) affording the target nucleoside (**15**).

^{1}H NMR (400 MHz, $(CD_3)_2SO$*,* ppm) δ 8.76 (s, 1H, *C^8*), 8.12 (d, *J* = 8.4 Hz, 1H, *H^6*), 7.62 – 7.49 (m, 1H, *H^1*), 6.01 (d, *J* = 5.9 Hz, 1H, *$H^{1'}$*), 5.51 (s, 1H, O*$H^{2'}$*), 5.25 (s, 1H O*$H^{3'}$*), 5.02 (t, *J* = 5.5 Hz, 1H, O*$H^{5'}$*), 4.61 (*app* t, *J* = 5.5 Hz, 1H, *$H^{2'}$*), 4.17 (*app* t, *J* = 4.2 Hz, 1H *$H^{3'}$*), 3.97 (*app* q, *J* = 4.0 Hz, 1H, *$H^{4'}$*), 3.68 (*app* dd, *J* = 10.6, 5.9 Hz, 1H, *$H^{5'}$ or $H^{5''}$*), 3.62 – 3.49 (m, 1H, *$H^{5'}$ or $H^{5''}$*).

^{13}C{^{1}H}-NMR (101 MHz, $(CD_3)_2SO$, ppm) δ 146.4(*C^4*), 144.4(*C^8*), 134.7(*C^5*), 130.7*(C^6*), 122.2(*C^1*), 87.2(*$C^{1'}$*), 85.6(*$C^{4'}$*), 73.5(*$C^{2'}$*), 70.4(*$C^{3'}$*), 61.3*($C^{5'}$*). Missing C2 due to poor signal strength.

RP-HPLC (Method A): t_R = 6.10 min, 96% conversion (std = 1).

HRMS (ESI) *m/z*: $[M+Na]^+$ calcd for $C_{11}H_{12}BrN_3O_4Na$ 351.9903; found 351.9902.

8-bromo-1-deaza-N3-ribosyl-purine (16)

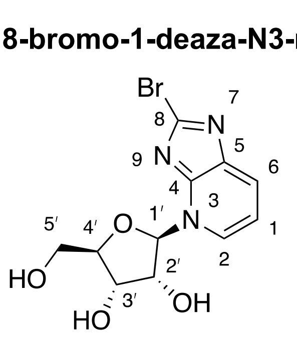

Prepared using the general enzymatic nucleoside transglycosylation procedure A. A 50 mM nucleobase stock solution (20 mg dissolved in 2020 µL) in DMSO (10.0 µmol, 200 µL, 1 equiv) was mixed with a 200 mM cytidine solution (486 mg in 10 mL), which was dissolved in mQ

H_2O (50.0 µmol, 500 µL, 10 equiv). Next, 300 µL of NDT stock solution (0.3 mg/mL) was added to afford a final volume of 1 mL and the resulting reaction mixture was shaken at 950 rpm at 40 °C for 24 h. The crude reaction mixture was purified by preparative HPLC (method D) affording the target nucleoside **16**.

^{1}H NMR (400 MHz, $(CD_3)_2SO$, ppm) δ 8.70 (dd, *J* = 6.6, 1.1 Hz, 1H, H^{2}), 8.31 (dd, *J* = 7.7, 1.0 Hz, 1H, H^{6}), 7.35 (dd, *J* = 7.7, 6.6 Hz, 1H, H^{1}), 6.42 (d, *J* = 4.1 Hz, 1H, $H^{1'}$), 5.73 (d, *J* = 5.7 Hz, 1H, O$H^{2'}$), 5.49 (dd, *J* = 5.8, 4.7 Hz, 1H, O$H^{5'}$), 5.29 – 5.21 (m, 1H, O$H^{3'}$), 4.39 (*app* qd, *J* = 4.1, 1.8 Hz, 1H, $H^{2'}$), 4.13 (m, 2H, $H^{3'}$ and $H^{4'}$), 3.84 (ddd, *J* = 12.3, 4.8, 2.2 Hz, 1H, $H^{5'}$ or $H^{5'}$), 3.70 (ddd, *J* = 12.3, 6.0, 2.3 Hz, 1H, $H^{5'}$ or $H^{5'}$).

^{13}C{^{1}H}-NMR (101 MHz, $(CD_3)_2SO$, ppm) δ 151.0(C^{4}), 148.6(C^{8}), 143.6(C^{5}), 128.9(C^{6}), 128.3(C^{2}), 113.6(C^{1}), 94.3($C^{1'}$), 86.1($C^{4'}$), 75.3($C^{2'}$), 69.4($C^{3'}$), 60.3($C^{5'}$).

RP-HPLC (Method A): t_R = 4.21 min, 61% conversion (std = 4).

HRMS (ESI) *m/z*: $[M+H]^+$ calcd for $C_{11}H_{13}{}^{79}BrN_3O_4$ 330.0084; found 330.0079.

5-iodo-uridine (18)

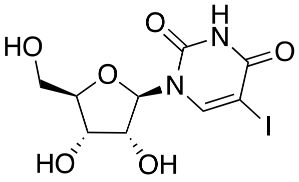

Prepared using the general enzymatic nucleoside transglycosylation procedure A. A 50 mM nucleobase stock solution (12 mg dissolved in 1008 µL) in DMSO (10.0 µmol, 200 µL, 1 equiv) was mixed with a 200 mM cytidine solution (486 mg in 10 mL), which was dissolved in mQ H_2O (100.0 µmol, 500 µL, 10 equiv). Next, 300 µL of NDT stock solution (0.3 mg/mL) to afford a final volume of 1 mL and the resulting reaction mixture was shaken at 950 rpm at 40 °C for 24 h. The crude reaction mixture was purified by preparative HPLC (method D) affording the target nucleoside **18**.

RP-HPLC (Method A): t_R = 3.75 min, 29% conversion (std = 1).

Characterisation of the target nucleoside was confirmed through ^{1}H NMR in comparison with the large-scale characterisation.

5-fluorouridine (19)

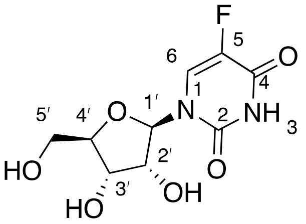

Prepared using the general enzymatic nucleoside transglycosylation procedure A. A 50 mM nucleobase stock solution (11 mg dissolved in 1691 µL) in H_2O (10.0 µmol, 200 µL, 1 equiv) was mixed with a 200 mM 5-methyluridine solution (516 mg in 10 mL), which was dissolved in mQ H_2O (100.0 µmol, 500 µL, 10 equiv). Next, 300 µL of NDT stock solution (0.3 mg/mL) to afford a final volume of 1 mL and the resulting reaction mixture was shaken at 950 rpm at 40 °C for 24 h. The crude reaction mixture was purified by preparative HPLC (method D) affording the target nucleoside (**19**).

^{1}H NMR (400 MHz, $(CD_3)_2SO$, ppm) δ 11.83 (s, 1H, *NH*), 8.14 (s, 1H, H^6), 5.73 (dd, *J* = 4.6, 1.9 Hz, 1H, $H^{1'}$), 5.35 (d, *J* = 5.2 Hz, 1H, O$H^{2'}$), 5.21 (t, *J* = 4.9 Hz, 1H, O$H^{5'}$), 5.04 (s, 1H, O$H^{3'}$), 4.00 (*app* dq, *J* = 9.6, 4.8 Hz, 2H, $H^{3'}$ and $H^{2'}$), 3.83 (*app* q, *J* = 3.2 Hz, 1H, $H^{4'}$), 3.71 – 3.62 (m, 1H, $H^{5'}$ *or* $H^{5''}$), 3.56 (ddd, *J* = 12.1, 4.9, 3.0 Hz, 1H, $H^{5'}$ *or* $H^{5''}$).

^{13}C{^{1}H}-NMR (101 MHz, $(CD_3)_2SO$, ppm) δ 164.9(C^4 or C^2), 151.5(C^4 or C^2), 137.7(C^6), 107.6, 88.3($C^{1'}$), 84.6($C^{4'}$), 73.7($C^{2'}$), 69.4($C^{3'}$), 60.4($C^{5'}$). Missing C5 due to poor signal strength.

^{19}F{^{1}H}-NMR (376 MHz, $(CD_3)_2SO$, ppm) δ −167.5.

NMR values in agreement with literature.[6]

RP-HPLC (Method A): t_R = 1.83 min, 18% conversion (std = 2).

HRMS (ESI) *m/z*: [M+Na]$^+$ calcd for $C_9H_{11}FN_2O_6Na$ 285.0493; found, 285.0489.

5-trifluorouridine (20)

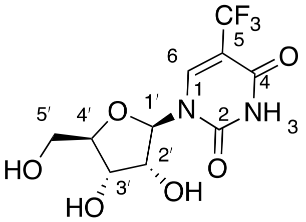

Prepared using the general enzymatic nucleoside transglycosylation procedure A. A 50 mM nucleobase stock solution (15 mg dissolved in 1666 µL) in DMSO (10.0 µmol, 200 µL, 1 equiv)

was mixed with a 200 mM cytidine solution (486 mg in 10 mL), which was dissolved in mQ H_2O (100.0 µmol, 500 µL, 10 equiv). Next, 300 µL of NDT stock solution (0.3 mg/mL) to afford a final volume of 1 mL and the resulting reaction mixture was shaken at 950 rpm at 40 °C for 24 h. The crude reaction mixture was purified by preparative HPLC (method D) affording the target nucleoside (**20**).

^{1}H NMR (400 MHz, $(CD_3)_2SO$, ppm) δ 11.83 (s, 1H, N*H*), 8.83 (s, 1H, *H^6*), 5.72 (d, *J* = 3.1 Hz, 1H, *$H^{1'}$*), 5.48 (d, *J* = 4.9 Hz, 1H, O*$H^{2'}$*), 5.31 (t, *J* = 4.4 Hz, 1H, O*$H^{5'}$*), 5.04 (d, *J* = 5.8 Hz, 1H, O*$H^{3'}$*), 4.06 (*app* q, *J* = 4.0 Hz, 1H, *$H^{2'}$*), 4.00 (*app* q, *J* = 5.0 Hz, 1H, *$H^{3'}$*), 3.90 (*app* dt, *J* = 6.2, 2.3 Hz, 1H, *$H^{4'}$*), 3.74 (*app* dt, *J* = 12.2, 3.3 Hz, 1H, *$H^{5'}$ or $H^{5''}$*), 3.64 – 3.55 (m, 1H, *$H^{5'}$ or $H^{5''}$*).

^{13}C{^{1}H}-NMR (101 MHz, $(CD_3)_2SO$, ppm) δ 160.0(*C^4* or *C^2*), 149.9(*C^4* or *C^2*), 142.3(*C^6*), 89.4(*$C^{1'}$*), 84.2(*$C^{4'}$*), 74.3(*$C^{2'}$*), 68.5(*$C^{3'}$*), 59.3(*$C^{5'}$*). Missing C5 due to poor signal strength.

^{19}F{^{1}H}-NMR (376 MHz, $CD_3)_2SO$, ppm) δ −61.5.

NMR values in agreement with literature.[1]

RP-HPLC (Method A): t_R = 3.80 min, 29% conversion (std = 1).

HRMS (ESI) *m/z*: $[M+Na]^+$ calcd for $C_{10}H_{11}F_3N_2O_6Na$ 335.0459; found, 335.0456

5-ethynyluridine (21)

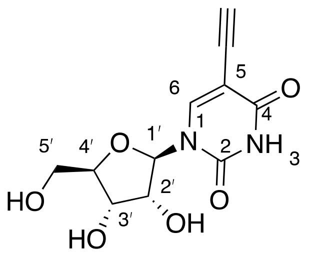

Prepared using the general enzymatic nucleoside transglycosylation procedure A. A 50 mM nucleobase stock solution (15 mg dissolved in 2204 µL) in DMSO (10.0 µmol, 200 µL, 1 equiv) was mixed with a 200 mM cytidine solution (486 mg in 10 mL), which was dissolved in mQ H_2O (100.0 µmol, 500 µL, 10 equiv). Next, 300 µL of NDT stock solution (0.3 mg/mL) to afford a final volume of 1 mL and the resulting reaction mixture was shaken at 950 rpm at 40 °C for 24 h. The crude reaction mixture was purified by preparative HPLC (method D) affording the target nucleoside (**21**).

^{1}**H NMR** (400 MHz, $(CD_3)_2SO$, ppm) δ 11.63 (s, 1H, N*H*), 8.38 (s, 1H, H^6), 5.75 (d, *J* = 4.6 Hz, 1H, $H^{1'}$), 5.42 (d, *J* = 5.3 Hz, 1H, O$H^{2'}$), 5.23 (t, *J* = 4.8 Hz, 1H, O$H^{5'}$), 5.06 (d, *J* = 5.3 Hz, 1H, O$H^{3'}$), 4.10 (s, 1H, C*H*), 4.05 (*app* q, *J* = 5.0 Hz, 1H, $H^{2'}$), 3.99 (*app* q, *J* = 4.9 Hz, 1H, $H^{3'}$), 3.87 (dd, *J* = 4.8, 2.7 Hz, 1H, $H^{4'}$), 3.69 (ddd, *J* = 12.1, 4.9, 3.0 Hz, 1H, $H^{5'}$ *or* $H^{5''}$), 3.58 (ddd, *J* = 12.1, 4.8, 2.7 Hz, 1H, $H^{5'}$ *or* $H^{5''}$).

^{13}**C{^{1}H}-NMR** (101 MHz, $(CD_3)_2SO$, ppm) δ 161.6(C^4 or C^2), 149.6(C^4 or C^2), 144.6(C^6), 97.6(*C*CH), 88.4($C^{1'}$), 84.7(C^5*C*), 83.6($C^{4'}$), 76.3(C^5), 73.9($C^{2'}$), 69.3($C^{3'}$), 60.2($C^{5'}$).

NMR values in agreement with literature.[8]

RP-HPLC (Method A): t_R = 3.1 min, 39% conversion (std = 2).

HRMS (ESI) *m/z*: $[M+Na]^+$ calcd for $C_{11}H_{12}N_2O_6Na$ 291.0588; found, 291.0584

5-azidouridine (22)

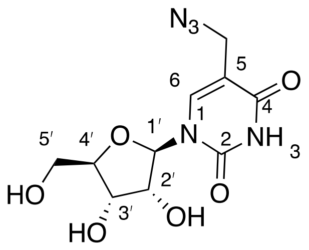

Prepared using the general enzymatic nucleoside transglycosylation procedure A. A 50 mM nucleobase stock solution (11 mg dissolved in 1316 µL) in DMSO (10.0 µmol, 200 µL, 1 equiv) was mixed with a 200 mM cytidine solution (486 mg in 10 mL), which was dissolved in mQ H_2O (100.0 µmol, 500 µL, 10 equiv). Next, 300 µL of NDT stock solution (0.3 mg/mL) to afford a final volume of 1 mL and the resulting reaction mixture was shaken at 950 rpm at rt for 24 h. The crude reaction mixture was purified by preparative HPLC (method D) affording the target nucleoside (**22**).

^{1}**H NMR** (400 MHz, $(CD_3)_2SO$, ppm) δ 11.54 (s, 1H, N*H*), 8.08 (s, 1H, H^6), 5.77 (d, *J* = 5.2 Hz, 1H. $H^{1'}$), 5.39 (d, *J* = 5.6 Hz, 1H, O$H^{2'}$), 5.16 – 5.02 (m, 2H, O$H^{3'}$ and O$H^{5'}$), 4.08 – 4.00 (m, 3H, $H^{2'}$ and C*H*), 3.97 (*app* q, *J* = 4.9 Hz, 1H, , $H^{3'}$), 3.88 – 3.80 (m, 1H, $H^{4'}$), 3.66 (ddd, *J* = 12.0, 5.3, 3.4 Hz, 1H, , $H^{5'}$ *or* $H^{5''}$), 3.60 – 3.49 (m, 1H, , $H^{5'}$ *or* $H^{5''}$).

^{13}C{^{1}H}-NMR (101 MHz, $(CD_3)_2SO$, ppm) δ 164.0(*C^{4}*), 150.7(*C^{2}*), 140.1(*C^{6}*), 108.4(*C^{5}*), 88.4(*C$^{1'}$*), 85.3*(C$^{4'}$*), 74.0*(C$^{2'}$*), 70.1(*C$^{3'}$*), 61.2*(C$^{5'}$*), 47.4(C^{5}*C*H).

NMR values in agreement with literature.[9]

RP-HPLC (Method A): t_R = 3.2 min, 35% conversion (std = 1).

HRMS (ESI) *m/z*: $[M-H]^-$ calcd for $C_{10}H_{12}N_5O_6$ 298.0793; found, 298.0792

5-fluorocytidine (23)

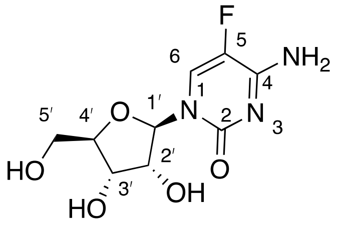

Prepared using the general enzymatic nucleoside transglycosylation procedure A. A 50 mM nucleobase stock solution (14 mg dissolved in 2169 μL) in H_2O (10.0 μmol, 200 μL, 1 equiv) was mixed with a 200 mM 5-methyluridine solution (516 mg in 10 mL), which was dissolved in mQ H_2O (100.0 μmol, 500 μL, 10 equiv). Next, 300 μL of NDT stock solution (0.3 mg/mL) to afford a final volume of 1 mL and the resulting reaction mixture was shaken at 950 rpm at 40 °C for 24 h. The crude reaction mixture was purified by preparative HPLC (method D) affording the target nucleoside (**23**).

^{1}H NMR (500 MHz, $(CD_3)_2SO$, ppm) δ 8.21 (d, *J* = 7.4 Hz, 1H, *H6*), 7.73 (s, 1H, N*H*), 7.50 (s, 1H, N*H*), 5.72 (dd, *J* = 3.9, 1.9 Hz, 1H, *H$^{1'}$*), 5.33 (d, *J* = 5.2 Hz, 1H, O*H$^{3'}$* or O*H$^{2'}$*), 5.19 (t, *J* = 5.0 Hz, 1H O*H$^{5'}$*), 4.98 (d, *J* = 5.5 Hz, 1H, O*H$^{3'}$* or O*H$^{2'}$*), 3.95 (*app* tt, *J* = 9.4, 5.1 Hz, 2H, *H$^{3'}$* and *H$^{2'}$*), 3.84 (*app* dt, *J* = 5.6, 2.9 Hz, 1H, *H$^{4'}$*), 3.74 – 3.67 (m, 1H, *H$^{5'}$ or H$^{5''}$*), 3.62 – 3.54 (m, 1H, *H$^{5'}$ or H$^{5''}$*).

^{13}C{^{1}H}-NMR (126 MHz, $(CD_3)_2SO$, ppm) δ 157.3 (d, J = 13.3 Hz, *C^{4}*), 154.0(*C^{2}*), 136.0 (d, J = 240.1 Hz, *C^{5}*), 125.7(d, *J* = 32.3*, C^{6})*, 89.7(*C$^{1'}$*), 84.5*(C$^{4'}$*), 74.7*(C$^{2'}$*), 69.5(*C$^{3'}$*), 60.6(*C$^{5'}$*).

^{19}F{^{1}H}-NMR (471 MHz, $(CD_3)_2SO$, ppm) δ −167.7.

NMR values in agreement with literature.[7]

RP-HPLC (Method A): t_R = 1.50 min, 18% conversion (std = 1).

HRMS (ESI) *m/z*: $[M+Na]^+$ calcd for $C_9H_{12}FN_3O_5Na$ 284.0653; found, 284.0650.

5-methylcytosine (24)

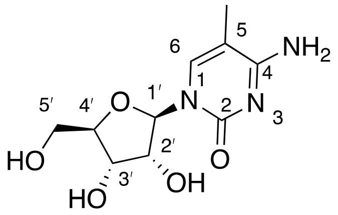

Prepared using the general enzymatic nucleoside transglycosylation procedure A. A 50 mM nucleobase stock solution (8 mg dissolved in 1279 µL) in H_2O (10.0 µmol, 200 µL, 1 equiv) was mixed with a 200 mM 5-methyluridine solution (516 mg in 10 mL), which was dissolved in mQ H_2O (100.0 µmol, 500 µL, 10 equiv). Next, 300 µL of NDT stock solution (0.3 mg/mL) to afford a final volume of 1 mL and the resulting reaction mixture was shaken at 950 rpm at 40 °C for 24 h. The crude reaction mixture was purified by preparative HPLC (method D) affording the target nucleoside (**24**).

^{1}H NMR (400 MHz, $(CD_3)_2SO$, ppm) δ 7.68 (s, 1H, H^6), 7.25 (s, 1H, *NH*), 6.78 (s, 1H, *NH*), 5.80 – 5.74 (m, 1H, $H^{1'}$), 5.26 – 5.19 (m, 1H, $OH^{2'}$), 5.06 (t, *J* = 5.3 Hz, 1H, $OH^{5'}$), 4.97 – 4.91 (m, 1H, $OH^{3'}$), 4.00 – 3.90 (m, 2H, $H^{3'}$ and $H^{2'}$), 3.80 (*app* q, *J* = 3.6 Hz, 1H, $H^{4'}$), 3.66 (ddd, *J* = 12.0, 5.2, 3.1 Hz, 1H, $H^{5'}$ *or* $H^{5''}$), 3.54 (ddd, *J* = 12.0, 5.5, 3.5 Hz, 1H, $H^{5'}$ *or* $H^{5''}$).

^{13}C{^{1}H}-NMR (101 MHz, $(CD_3)_2SO$, ppm) δ 165.8(C^4 or C^5), 139.3(C^6), 101.5(C^4 or C^5), 89.5($C^{1'}$), 84.5($C^{4'}$), 74.3($C^{2'}$), 69.9($C^{3'}$), 61.2($C^{5'}$), 13.8(C^5C). C2 missing due to poor signal strength.

RP-HPLC (Method A): t_R = 1.97 min, 15% conversion (std = 1).

HRMS (ESI) *m/z*: $[M+Na]^+$ calcd for $C_{10}H_{16}N_3O_5$ 258.1085; found, 258.1082.

3.3 2'-Fluoro Nucleoside Analogues

6-Bromo-1-deazapurine-2'-arabino-fluoro-ribose (29)

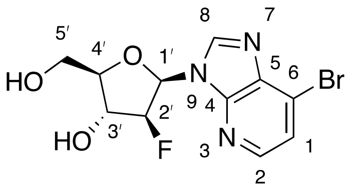

Prepared using the general enzymatic nucleoside transglycosylation procedure A. A 50 mM nucleobase stock solution (20 mg dissolved in 2020 µL) in mQ H_2O (5.0 µmol, 200 µL, 1 equiv) was mixed with a 100 mM 1-(2-Deoxy-2-fluoro-b-D-arabinofuranosyl)uracil solution (246 mg in 10 mL), which was dissolved in mQ H_2O (40.0 µmol, 800 µL, 8 equiv). Next, 1 mL of NDT stock solution (2 mg/mL) was added to afford a final volume of 2 mL and the resulting reaction mixture was shaken at 950 rpm at 40 °C for 24 h. The crude reaction mixture was purified by preparative HPLC (method D) affording the target nucleoside (**29**).

^{1}H NMR (400 MHz, $(CD_3)_2SO$, ppm) δ 8.70 (d, *J* = 1.9 Hz, 1H, H^8), 8.27 (d, *J* = 5.2 Hz, 1H, H^2), 7.65 (d, *J* = 5.2 Hz, 1H, H^1), 6.58 (dd, *J* = 13.8, 4.6 Hz, 1H, $H^{1'}$), 5.98 (d, *J* = 4.9 Hz, 1H, O$H^{3'}$), 5.27 (ddd, *J* = 52.6, 4.6, 3.9 Hz, 1H, $H^{2'}$), 5.13 (t, *J* = 5.6 Hz, 1H, O$H^{5'}$), 4.47 (*app* dq, *J* = 18.8, 4.6 Hz, 1H, $H^{3'}$), 3.90 (*app* tdd, *J* = 5.1, 4.1, 0.9 Hz, 1H, $H^{4'}$), 3.77 – 3.60 (m, 2H, $H^{5'}$ *or* $H^{5''}$).

^{13}C{^{1}H}-NMR (101 MHz, $(CD_3)_2SO$, ppm) δ 146.1(C^4), 144.8(C^2), 144.5 (d, *J* = 3.9 Hz, C^8), 134.0(C^5), 123.1(C^6), 122.0(C^1), 95.3 (d, *J* = 192.3 Hz, $C^{2'}$), 83.6 (d, *J* = 5.6 Hz, $C^{4'}$), 81.8 (d, *J* = 16.9 Hz, $C^{1'}$), 72.5 (d, *J* = 23.2 Hz, $C^{3'}$), 60.3($C^{5'}$).

^{19}F NMR (471 MHz, $(CD_3)_2SO$, ppm) δ –197.95 (ddd, *J* = 52.8, 19.0, 13.8 Hz).

RP-HPLC (Method A): t_R = 6.56 min, 25% conversion (std = 2).

HRMS (ESI) *m/z*: $[M+Na]^+$ calcd for $C_{11}H_{11}O_3N_3BrFNa$ 353.9860; found 353.9854.

6-Bromo-1-deazapurine-2'-fluoro-ribose (27)

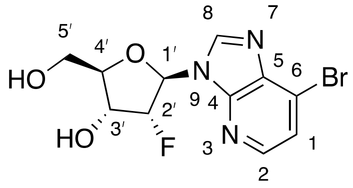

Prepared using the general enzymatic nucleoside transglycosylation procedure A. A 50 mM nucleobase stock solution (20 mg dissolved in 2020 µL) in mQ H_2O (5.0 µmol, 200 µL, 1 equiv) was mixed with a 200 mM 2-deoxyfluorocytidine solution (246 mg in 10 mL), which was dissolved in mQ H_2O (80.0 µmol, 800 µL, 16 equiv). Next, 1 mL of NDT stock solution (2 mg/mL) was added to afford a final volume of 2 mL and the resulting reaction mixture was shaken at 950 rpm at 40 °C for 24 h. The crude reaction mixture was purified by preparative HPLC (method D) affording the target nucleoside (**27**).

^{1}H NMR (400 MHz, $(CD_3)_2SO$, ppm) δ 8.82 (s, 1H, H^8), 8.27 (d, J = 5.2 Hz, 1H, H^2), 7.66 (d, J = 5.2 Hz, 1H, H^1), 6.39 (dd, J = 17.2, 2.4 Hz, 1H, $H^{1'}$) 5.73 (d, J = 6.1 Hz, 1H, O$H^{3'}$), 5.46 (ddd, J = 52.9, 4.5, 2.4 Hz, 1H, $H^{2'}$), 5.17 (t, J = 5.4 Hz, 1H, O$H^{5'}$), 4.52 (*app* dtd, J = 20.1, 6.6, 4.4 Hz, 1H, $H^{3'}$), 4.01 (*app* dt, J = 6.8, 3.2 Hz, 1H, $H^{4'}$), 3.79 (ddd, J = 12.4, 5.1, 2.7 Hz, 1H, $H^{5'}$ *or* $H^{5''}$), 3.61 (ddd, J = 12.4, 5.7, 3.9 Hz, 1H $H^{5'}$ *or* $H^{5''}$).

^{13}C{^{1}H}-NMR (101 MHz, $(CD_3)_2SO$, ppm) δ 145.9(C^4), 144.4 (d, J = 50.4, C^8), 134.8(C^5), 123.2(C^6), 122.0(C^1), 93.5 (d, J = 186.4 Hz, $C^{2'}$), 86.2 (d, J = 33.5 Hz, $C^{1'}$), 83.8($C^{4'}$), 68.1 (d, J = 16.1 Hz, $C^{3'}$), 60.0($C^{5'}$).

^{19}F NMR (471 MHz, $(CD_3)_2SO$) δ –202.8 – –204.2 (m).

RP-HPLC (Method A): t_R = 6.50 min, 22% conversion (std = 6).

HRMS (ESI) *m/z*: $[M+Na]^+$ calcd for $C_{11}H_{12}O_3N_3BrFNa$ 353.9860; found 353.9869.

2-chloro-adenosine-2'-fluoro-ribose (28)

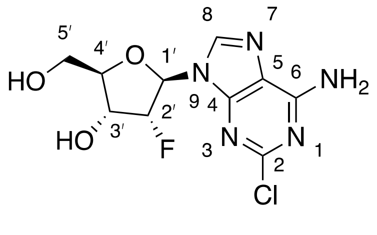

Prepared using the general enzymatic nucleoside transglycosylation procedure A. A 50 mM nucleobase stock solution (9 mg dissolved in 1068 µL) in mQ H_2O (5.0 µmol, 200 µL, 1 equiv) was mixed with a 200 mM 2-deoxyfluorocytidine solution (246 mg in 10 mL), which was dissolved in mQ H_2O (80.0 µmol, 800 µL, 16 equiv). Next, 1 mL of NDT stock solution (2 mg/mL) was added to afford a final volume of 2 mL and the resulting reaction mixture was

shaken at 950 rpm at 40 °C for 24 h. The crude reaction mixture was purified by preparative HPLC (method D) affording the target nucleoside (**28**).

^{1}H NMR (500 MHz, $(CD_3)_2SO$, ppm) δ 8.31 (s, 1H, *H^8*), 7.80 (s, 2H, N*H*), 6.10 (dd, *J* = 16.8, 2.6 Hz, 1H, *H$^{1'}$*), 5.64 (d, *J* = 6.2 Hz, 1H, O*H$^{3'}$*), 5.30 (ddd, *J* = 52.7, 4.5, 2.7 Hz, 1H, *H$^{2'}$*), 5.04 (t, *J* = 5.5 Hz, 1H, O*H$^{5'}$*), 4.42 – 4.31 (m, 1H, *H$^{3'}$*), 3.90 (*app* dd, *J* = 6.8, 3.4 Hz, 1H, *H$^{4'}$*), 3.68 (ddd, *J* = 12.4, 5.2, 2.8 Hz, 1H, *H$^{5'}$ or H$^{5''}$*), 3.52 (ddd, *J* = 12.3, 5.8, 4.0 Hz, 1H, *H$^{5'}$ or H$^{5''}$*).

^{13}C{^{1}H}-NMR (126 MHz, $(CD_3)_2SO$, ppm) δ 157.3, 153.6, 150.3, 140.0(*C^8*), 118.6, 93.9 (d, *J* = 187.4 Hz, *C$^{2'}$*), 86.2 (d, *J* = 33.1 Hz, *C$^{1'}$*), 84.5(*C$^{4'}$*), 68.6 (d, *J* = 15.8 Hz, *C$^{3'}$*), 60.7(*C$^{5'}$*).

NMR in agreement with literature values.[10]

^{19}F{^{1}H}-NMR (471 MHz, $(CD_3)_2SO$, ppm) δ – 204.0.

RP-HPLC (Method A): t_R = 5.63 min, 73% conversion (std = 4).

HRMS (ESI) *m/z*: $[M+Na]^+$ calcd for $C_{10}H_{11}O_3N_5ClFNa$ 326.0427; found 326.0429.

2-chloro-adenosine-2'-arabino-fluoro-ribose (30)

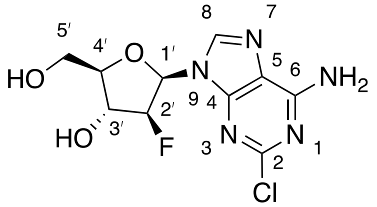

Prepared using the general enzymatic nucleoside transglycosylation procedure A. A 50 mM nucleobase stock solution (9 mg dissolved in 1068 µL) in mQ H_2O (5.0 µmol, 200 µL, 1 equiv) was mixed with a 100 mM 1-(2-Deoxy-2-fluoro-b-D-arabinofuranosyl)uracil solution (246 mg in 10 mL), which was dissolved in mQ H_2O (40.0 µmol, 800 µL, 8 equiv). Next, 1 mL of NDT stock solution (2 mg/mL) was added to afford a final volume of 2 mL and the resulting reaction mixture was shaken at 950 rpm at 40 °C for 24 h. The crude reaction mixture was purified by preparative HPLC (method D) affording the target nucleoside (**30**).

^{1}H NMR (400 MHz, $(CD_3)_2SO$, ppm) δ 8.27 (d, *J* = 2.1 Hz, 1H, *H^8*), 7.88 (s, 2H, N*H$_2$*), 6.33 (dd, *J* = 13.8, 4.6 Hz, 1H, *H$^{1'}$*), 5.96 (d, *J* = 5.1 Hz, 1H, O*H$^{3'}$*), 5.37 – 5.13 (m, 1H, *H$^{2'}$*), 5.08 (t, *J* =

5.7 Hz, 1H, O*H*[5']), 4.43 (*app* dq, *J* = 18.9, 4.8 Hz, 1H, *H*[3']), 3.90 – 3.81 (m, 1H, *H*[4']), 3.75 – 3.59 (m, 2H, *H*[5'] and *H*[5'']).

$^{13}C\{^1H\}$-NMR (101 MHz, $(CD_3)_2SO$, ppm) δ 157.3 (*C*[2] or *C*[6]), 153.7 (*C*[2] or *C*[6]), 150.6 (*C*[5] or *C*[4]), 140.5 (d, *J* = 4.4 Hz, *C*[8]), 117.8 (*C*[5] or *C*[4]), 95.8 (d, *J* = 192.1 Hz, *C*[2']), 84.0 (d, *J* = 5.6 Hz, *C*[4']), 81.9 (d, *J* = 16.9 Hz, *C*[1']) 73.0 (d, *J* = 23.0 Hz, *C*[3']), 60.8(*C*[5']).

NMR values in agreement with literature.[11]

^{19}F NMR (471 MHz, $(CD_3)_2SO$, ppm) δ – 198.0

RP-HPLC (Method A): t_R = 5.63 min, conversion 25% (std = 1).

HRMS (ESI) *m/z*: $[M+Na]^+$ calcd for $C_{10}H_{11}O_3N_5ClFNa$ 326.0427; found 326.0432.

HPLC trace – non-acceptance of 5-trifluoromethyluracil with fluorinated nucleoside donors

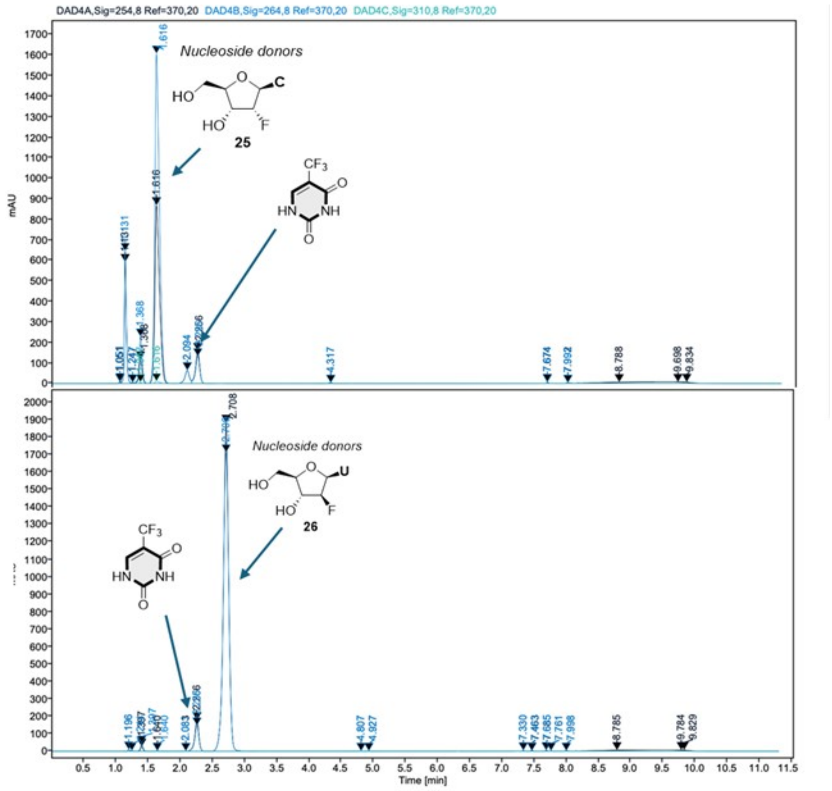

Figure S1a – HPL chromatogram showing no formation of the corresponding fluorinated pyrimidine nucleosides. Retention times; 2.20 min = Nucleobase **37**, 2.71 min = 2'-fluoro-arabino-uridine, 1.62 min = 2'-fluoro-cytidine.

HPLC trace – non-acceptance of 6-dimethyladenine with fluorinated nucleoside donors

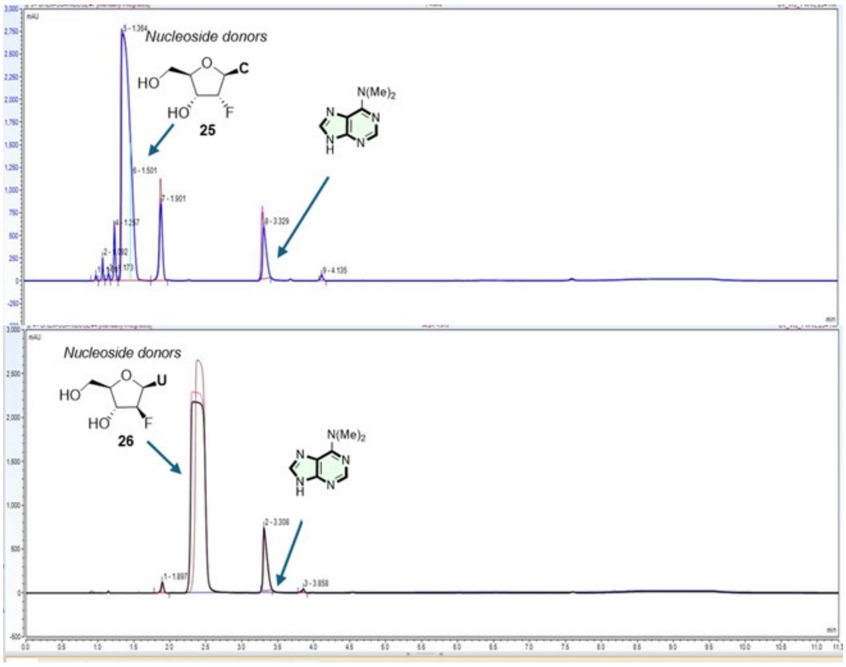

Figure S1b – HPL chromatogram showing no formation of the corresponding fluorinated purine nucleosides. Retention times; 2.30 min = Nucleobase **38**, 2.71 min = 2'-fluoro-arabinose-uridine, 1.62 min = 2'-fluoro-cytidine.

4. Protein Expression and Crystallisation

4.1 Expression

The coding sequence for the mutants of *Lactobacillus leichmannii* NDT (Uniprot: Q9R5V5) with an N-terminal 6-His tag, was synthesized by GenScript (with codon optimization for *Escherichia coli*) and subcloned into a pET29a+ plasmid. The plasmids were received from GenScript in the lyophilised form. Plasmid DNA was resuspended in nuclease-free water (6 µL) according to manufacturer protocol.

Amino acid sequence of protein used in this work:

***Ll*NDT-WT**

GSSHHHHHHSSGLEVLFQGPAMPKKTIYFGAGWFTDRQNKAYKEAMEALKENPTIDLENS
YVPLDNQYKGIRVDEHPEYLHDKVWATATYNNDLNGIKTNDIMLGVYIPDEEDVGLGMELG
YALSQGKYVLLVIPDEDYGKPINLMSWGVSDNVIKMSQLKDFNFNKPRFDFYEGAVY

NDT-Mutant (Tyr7→ Phe & Asp72 → Asn)

GSSHHHHHHSSGLEVLFQGPAMPKKTI**F**FGAGWFTDRQNKAYKEAMEALKENPTIDLENS
YVPLDNQYKGIRVDEHPEYLHDKVWATATYNN**N**LNGIKTNDIMLGVYIPDEEDVGLGMELG
YALSQGKYVLLVIPDEDYGKPINLMSWGVSDNVIKMSQLKDFNFNKPRFDFYEGAVY

Plasmid (0.8 μL) containing target gene pET29a+-NDT was transformed into BL21 (DE3) *E. coli* competent cells (Invitrogen) using the heat shock method. Transformants harbouring the plasmid were plated on LB agar containing 50 μg/ mL kanamycin and incubated at 37 °C overnight. Colonies were inoculated into 10 mL LB media containing 50 μg/ mL kanamycin and grown overnight at 37 °C while shaking gently at 200 rpm. Overnight transformants were used to inoculate 450 mL of LB media supplemented with 50 μg/ mL kanamyc into an OD of 0.01 and incubated at 37 °C, 200 rpm until reaching an OD of ~0.4. When OD reached ~0.4 cells were induced with IPTG (0.5 mM, 2.5 mL) solution for 3 hours. The cells were harvested by centrifugation (10,000 x g for 20 min at 4 °C) and the supernatant was discarded. The cell pellet was resuspended in 10 mM sodium phosphate buffer pH 7 (~10 mL buffer per 1 g of cell pellet). The resuspended cell pellets were lysed using a French press. The resulting cell lysate was separated by centrifugation (10,000 g, 30 mins, 4 °C) and the supernatant collected.

4.2 Purification of *Ll*NDT-WT

Binding Buffer (Buffer A): 10 mM sodium phosphate, 10 mM imidazole, 100 mM NaCl, pH 7.0
Binding Buffer (Buffer B): 10 mM sodium phosphate, 500 mM imidazole, 100 mM NaCl, pH 7.0.

The collected supernatant was filtered through a 0.45 μM PES filter and the proteins were purified by affinity chromatography. A 5 mL His trap FF column (GE Healthcare 17525501) was fitted on an Akta Pure protein purification system and equilibrated with 5 column volumes (CV) of binding buffer A before the supernatant of the cell lysate was loaded on to the column. The flow-through was collected as the non-absorbed fraction (NAF). The column was then washed with binding buffer A for 13 CV and when the UV absorbance was stable the protein was eluted with a gradient of imidazole (Buffer B 0-100%) over 17 CV. The fractions were analysed by SDS-PAGE.

General SDS-PAGE conditions involved mixing the sample with appropriate amount of 4X SDS dye and loaded into wells of NovexTM 4-20% Tris-Glycine Mini-Gels 50:50. Gels were

run in 1x running buffer for 60 minutes at 140 V. SDS-PAGE gels were stained in Coomassie Blue for 1 hour prior to de-staining overnight at room temperature, on a shaking-platform.

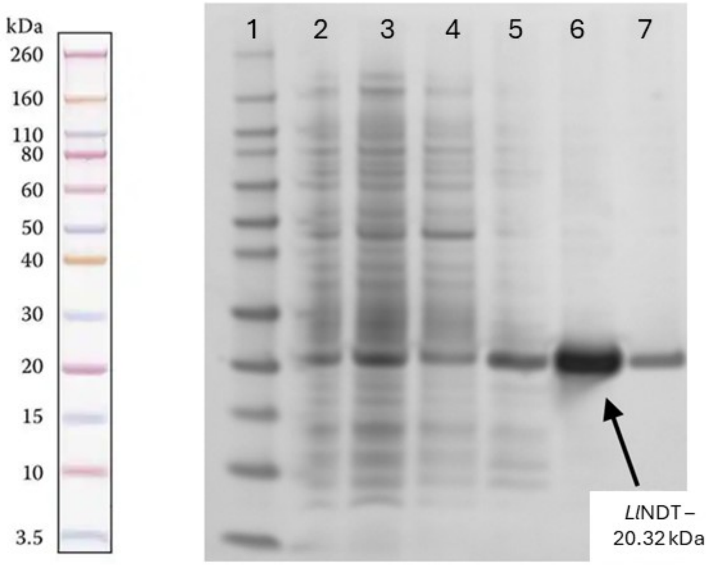

Figure S2a – HisTRAP FF SDS PAGE gel – lane order, 1 = Marker, 2 = flow through, 3 = flow through, 4 = column wash, 5 = pure NDT fraction, 6 = pure NDT fraction, 7 = post fraction wash out.

Appropriate fractions were pooled together, and 200 μL (400 units) of HRV 3C protease was added and dialysed over 48 hours at 4 °C into 20 mM sodium phosphate, 100 mM NaCl, pH 7.0 for his tag removal. His tag cleavage was monitored and analysed by SDS-PAGE. Once his cleavage was complete, fractions were pooled and loaded onto a 5 mL His trap FF column (GE Healthcare 17525501) using a Akta Pure protein purification system. The system was once again equilibrated with 5 column volumes of binding buffer A before loading of the pooled fractions. The flow-through was collected as the non-absorbed fraction containing the cleaved LlNDT protein. This was confirmed once again by SDS-PAGE, before appropriate fractions were applied to a 5,000 MW Amicon Centrifugal filter unit to be concentrated (10 mg/mL) and calculated by nanodrop.

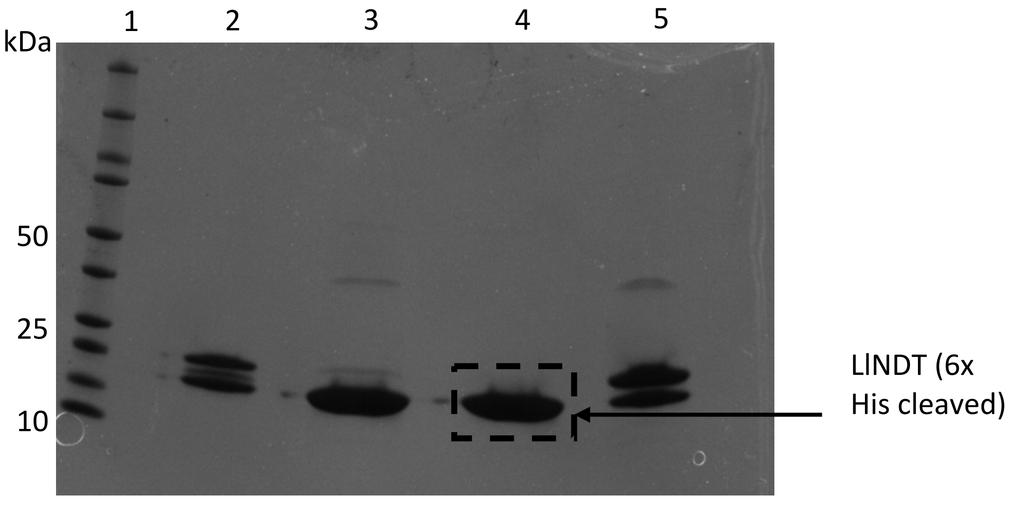

Figure S2b – SDS PAGE gel – lane order: 1 = ladder, 2 = HRV 3C protease histag removal after 24 hours, 3 = HRV 3C protease histag removal after 48 hours, 4 = pooled fractions after the second IMAC following histag removal, 5= HRV 3C protease histag removal after 30 hours.

For crystallization studies, pooled fractions were additionally purified by SEC using the following protocol (SEC buffer: 20 mM sodium phosphate, 100 mM NaCl, pH 7.0). A Superdex 200 prep grade 16/60 Size Exclusion chromatography (SEC) column was equilibrated with SEC buffer and loaded with 2.5 mL aliquots of the His Trap concentrate (10 mg/mL). The column was eluted with SEC buffer with 1 mL fractions collected and analysed by SDS-PAGE. Fractions containing NDT were concentrated using a 5,000 MW Amicon Centrifugal filter unit to 1.5 mL at a concentration of 21 mg/mL, with 0.75 mL in 10 % glycerol (20 mM sodium phosphate, 100 mM NaCl, pH 7.0) and an alternative 0.75 mL in 20 mM sodium phosphate, 100 mM NaCl, pH 7.0. This was flash frozen with N_2 (l) and stored at 20°C.

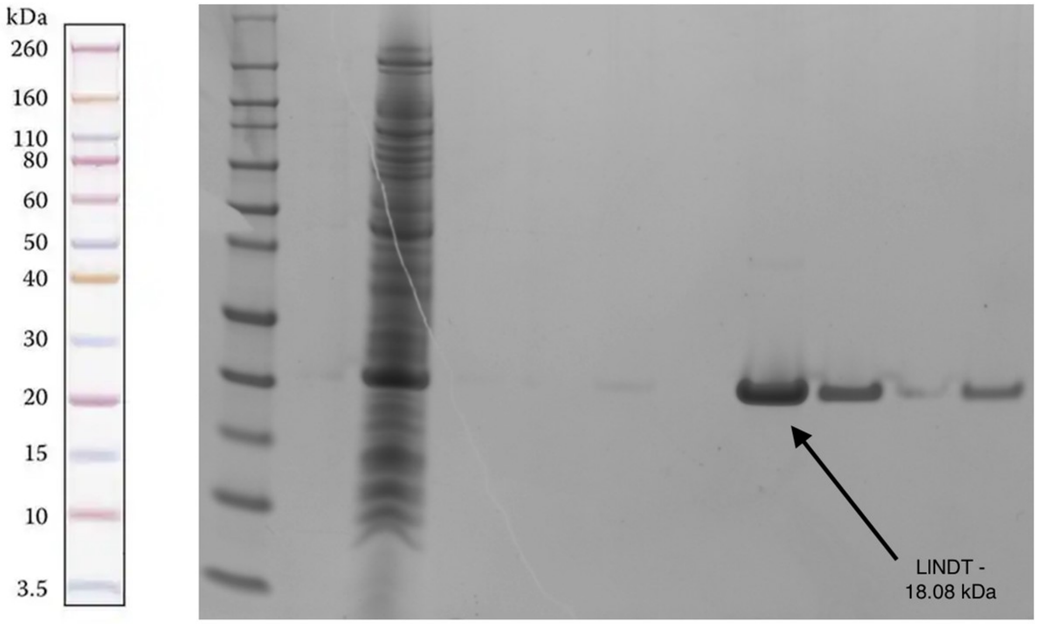

Figure S2c - Size exclusion chromatography (SEC) SDS PAGE gel – lane order, blank, HisTrap flow through, blank, HisTrap elution, blank, SEC pure fraction, SEC pure fraction, blank, SEC pure fraction**.**

4.3 Purification of NDT-Y7F/D72N

Binding Buffer (Buffer A): 10 mM sodium phosphate, 10 mM imidazole, 100 mM NaCl, pH 7.0
Binding Buffer (Buffer B): 10 mM sodium phosphate, 500 mM imidazole, 100 mM NaCl, pH 7.0.

The collected supernatant was filtered through a 0.45 μM PES filter and the proteins were purified by affinity chromatography. A 5 mL Histrap FF column (GE Healthcare 17525501) was fitted on an Akta Pure protein purification system and equilibrated with 5 column volumes (CV) of binding buffer A before the supernatant of the cell lysate was loaded on to the column. The flow-through was collected as the non-absorbed fraction (NAF). The column was then washed with binding buffer A for 13 CV and when the UV absorbance was stable the protein was eluted with a gradient of imidazole (Buffer B 0-100%) over 17 CV. The fractions were analysed by SDS-PAGE.

Appropriate fractions were pooled together, and 500 μL (400 units) of HRV 3C protease was added and dialysed over 24 hours at 4 °C into 20 mM sodium phosphate, 100 mM NaCl, pH 7.0 for his tag removal. His tag cleavage was monitored and analysed by SDS-PAGE. Once his tag cleavage was complete, fractions were pooled and loaded onto a 5 mL His trap FF column (GE Healthcare 17525501) using a Akta Pure protein purification system. The system was once again equilibrated with 5 column volumes of binding buffer A before loading of the pooled fractions. The flow-through was collected as the non-absorbed fraction containing the cleaved LINDT protein. This was confirmed once again by SDS-PAGE, before appropriate fractions were applied to a 5,000 MW Amicon Centrifugal filter unit to be concentrated (10 mg/mL) and calculated by nanodrop.

For crystallization studies, pooled fractions were additionally purified by SEC using the following protocol (SEC buffer: 20 mM sodium phosphate, 100 mM NaCl, pH 7.0). A Superdex 200 prep grade 16/60 Size Exclusion chromatography (SEC) column was equilibrated with SEC buffer and loaded with 10 mL of the His Trap concentrate (10 mg/mL). The column was eluted with SEC buffer with 1 mL fractions collected and analysed by SDS-PAGE. Fractions containing NDT were concentrated using a 5,000 MW Amicon Centrifugal filter unit to 4 mL at a concentration of 12 mg/mL, with 2 mL in 10 % glycerol (20 mM sodium phosphate, 100 mM NaCl, pH 7.0) and an alternative 2 mL in 20 mM sodium phosphate, 100 mM NaCl, pH 7.0. This was flash frozen with N_2 (l) and stored at 20°C.

General SDS-PAGE conditions involved mixing the sample with appropriate amount of 4X SDS dye and loaded into wells of NovexTM 4-20% Tris-Glycine Mini-Gels 50:50. Gels were run in 1x running buffer for 60 minutes at 140 V. SDS-PAGE gels were stained in Coomassie Blue for 1 hour prior to de-staining overnight at room temperature, on a shaking-platform.

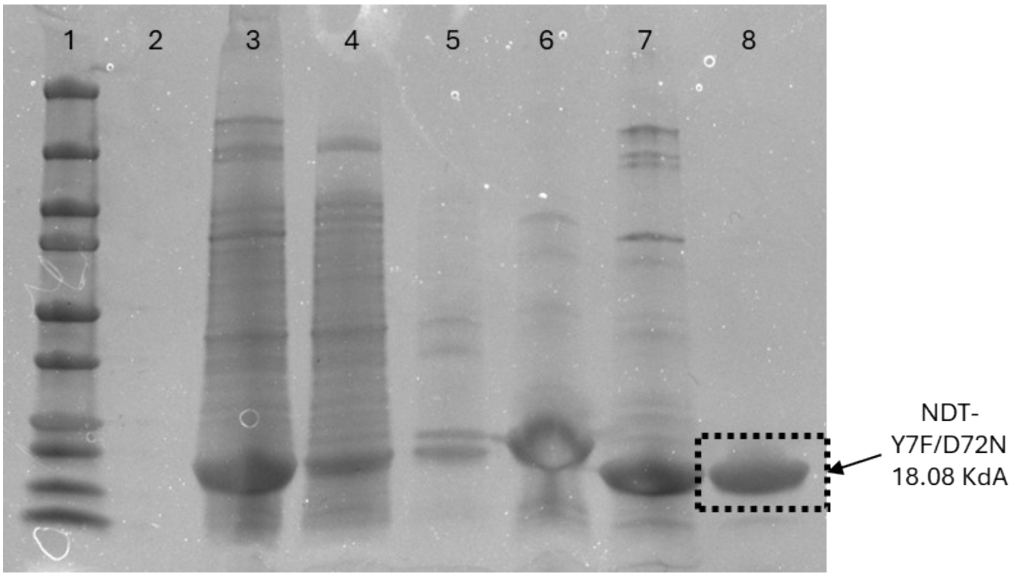

Figure S3 – SDS PAGE gel of the three mutants expressed. Lane 1 = ladder, lane 2 = empty, lane 3 = NDT-C lysate, lane 4 = NDT-C Histrap flow through, lane 5 = Histrap wash, lane 6 = NDT-C histrap fraction pooled, lane 7 = NDT-C cleaved with HRVC 3C, lane 8 = NDT-C following histrap cleavage, IMAC and SEC

4.4 Crystallisation

*Ll*NDT-2 and the Y7F/D72N mutant were concentrated to 21 mg mL^{-1} and 12 mg mL^{-1} respectively, in 20 mM NaH_2PO_4 pH 7.0 buffer with 100 mM NaCl. Crystals were grown in drops containing 500 nL protein and 500 nL mother liquor. Crystals of *Ll*NDT-2 were obtained in drops containing 35 % Tacsimate pH 7.0, and crystals of the Y7F/D72N mutant were obtained in drops containing 25 % Tacsimate pH 7.0. Crystals were soaked in their mother liquor containing 10 % ethylene glycol and 10 mM cytidine, which had been derived from a 100 mM stock solution in DMSO.

4.5 Data Collection and Refinement

The datasets described were collected at the Diamond Light Source, Didcot, Oxfordshire U.K. on beamline I03. Data were processed and integrated using XDS[12] and scaled using SCALA[13] included in the Xia2 processing system[14]. Data collection statistics are provided in Table S1. Crystals for all Datasets were obtained in space group $I2_13$, with approximately the same cell dimensions and with two molecules in the asymmetric unit that constituted one third of the *Ll*NDT-2 hexamer. The structures of *Ll*NDT-2 were solved by molecular replacement using MOLREP[15] with the monomer of LLNDT (PDB code 1F8Y[16]) as the model. The structures were built and refined using iterative cycles in Coot[17] and REFMAC[18], employing local NCS restraints in the refinement cycles. Following building and refinement of the protein and water

molecules in the structural complexes, residual density was observed in the omit maps at the active sites. For the WT enzyme, this could be modelled as the ribosylated enzyme with cytosine; modelling and refinement with intact cytidine gave rose to negative density between these species. For the Y7F/D72N mutant, the density was successfully modelled as cytidine.

The final structures of the ribosylated-WT/cytosine and Y7F/D72N-cytidine complexes gave R_{cryst}/R_{free} values of 0.19/0.22 and 0.16/0.19 respectively. Refinement statistics for the structures are presented in Table S1. The coordinates and structure factors have been deposited in the Protein Databank as **ribosylated-WT-*Ll*NDT-2/cytosine (PDB code 9GN2)**, and *Ll*NDT-2-Y7F/D72N-cytidine (**PDB code 9GN4**) respectively.

Table S1: Data collection and refinement statistics for datasets in this manuscript. Numbers in brackets refer to data for highest resolution shells.

	***LL*NDT-2 ribosylated with cytosine**	***LL*NDT-2 Y7F/D72N with cytidine**
Beamline	I03	I03
Wavelength (Å)	0.976269	0.976277
Resolution (Å)	60.82-2.41 (2.50-2.41)	61.29 – 2.48 (2.58-2.48)
Space Group	$I2_13$	$I2_13$
Unit cell (Å)	a = b = c =148.98; α = β = γ = 90.00˚	a = b = c =150.13; α = β = γ = 90.00˚
No. of molecules in the asymmetric unit	2	2
Unique reflections	21394 (2231)	20103 (2256)
Completeness (%)	100.0 (100.0)	100.0 (100.0)
R_{merge} (%)	0.07 (1.65)	0.16 (1.56)
$R_{p.i.m.}$	0.02 (0.37)	0.04 (0.34)
Multiplicity	40.5 (40.6)	41.6 (43.5)
$<I/\sigma(I)>$	35.0 (3.0)	26.1 (3.9)
Overall *B* from Wilson plot ($Å_2$)	68	47
$CC_{1/2}$	1.00 (0.87)	1.00 (0.90)
R_{cryst}/ R_{free} (%)	0.19/0.22	0.16/0.19
r.m.s.d 1-2 bonds (Å)	0.007	0.008
r.m.s.d 1-3 angles (°)	1.69	1.70

Avge main chain B (Å^2)	69	51
Avge side chain B (Å^2)	76	57
Avge waters B (Å^2)	69	54
Avge Ligand B (Å^2)	87	67

5. Mutant NDT-Y7F/D72N Tranglycosylation conversions

5.1 Ribose transglycosylations using NDT-Y7F/D72N

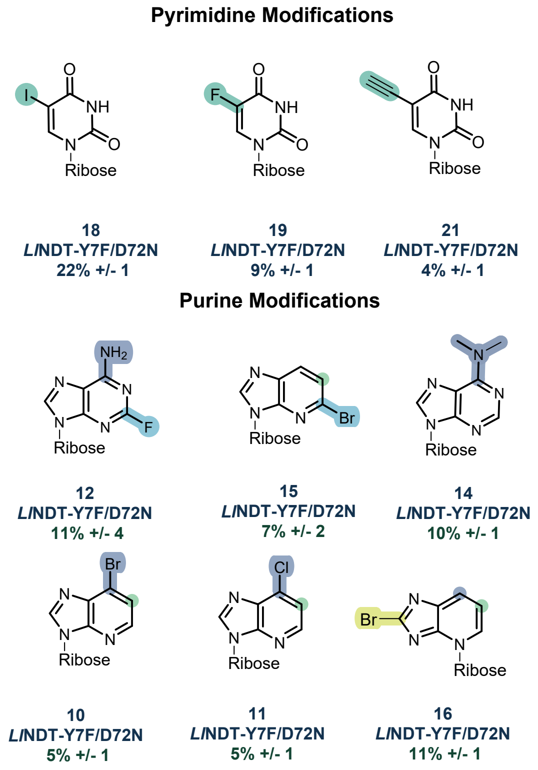

Figure S4 – Scope reactions using mutant *L*/NDT-Y7F/D72N

L/NDT-Y7F/D72N reaction conditions were replicated using existing conditions for *L*/NDT-WT. General reaction conditions: **7** or **8** (5-10 equiv.), nucleobase (1 equiv.), solvent (DMSO:H_2O,1:4), *L*/NDT-2 (100 to 300 μg/mL).

6. Synthesis of 2' and 3' modified nucleosides for substrate scope transglycosylation investigations

6.1 Synthesis of 2',3' -dideoxy-3'-thiothymidine (31)

Intermediate **38** was synthesized according to literature protocols[19] followed by deprotection in acidic conditions yielding the intermediate **39** (Scheme S1). Deacylation by aminolysis with 40 % methylamine afforded **31.**

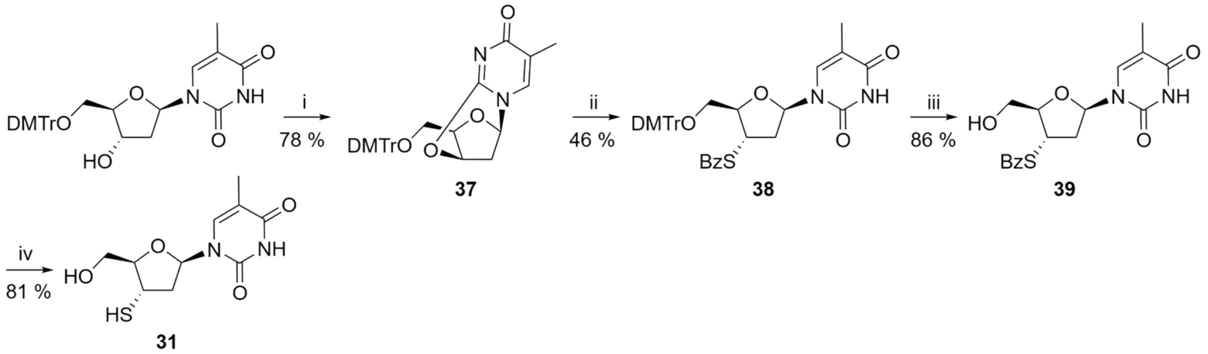

Scheme S1. Synthesis of **31**. Conditions: i) PPh_3, EtOAc, DIAD, r.t., 16 h; ii) NaSBz, DMF, 100 °C, 24 h; iii) conc. HCl/MeOH, $HO(CH_2)_2SH$, DCM, rt 5 min; iv) 40 % $MeNH_2$ (aq), rt, 16 h.

5'-O-(4,4'-dimethoxytrityl)-2,3'-anhydrothymidine (**37**). To a solution of 5'-*O*-(4,4'-dimethoxytrityl)-thymidine (9.0 g, 16.5 mmol) and PPh_3 (4.8 g, 18.2 mmol) in EtOAc (45 mL) was added dropwise diisopropyl azodicarboxylate (3.6 mL, 18.2 mmol) over 12 min and the resulting solution was stirred at rt for 16 h. The reaction solution was concentrated under a vacuum and the residue was purified by flash silica column chromatography eluting with a gradient of 2 % to 20 % MeOH: EtOAc in affording **37** (6.8 g, 78 %) as a white solid. ^{1}H NMR (400 MHz, $(CD_3)_2SO$) δ 7.63 (d, J = 1.2 Hz, 1H), 7.41 – 7.33 (m, 2H), 7.32 – 7.15 (m, 7H), 6.88 – 6.83 (m, 4H), 5.89 (d, J = 3.8 Hz, 1H), 5.33 – 5.28 (m, 1H), 4.41 (ddd, J = 7.7, 4.9, 2.5 Hz, 1H), 3.73 (s, 3H), 3.72 (s, 3H), 3.16 – 3.03 (m, 2H), 2.62 – 2.54 (m, 1H), 2.49 – 2.42 (m, 1H), 1.78 (d, J = 1.2 Hz, 3H). ^{13}C{^{1}H} NMR (101 MHz, $(CD_3)_2SO$) δ 170.7, 158.1, 158.0, 153.3, 144.6, 136.7, 135.2, 135.0, 129.6 (4C), 127.8 (2C), 127.6 (2C), 126.6, 116.1, 113.2 (2C), 113.2 (2C), 86.8, 85.8, 83.5, 77.1, 62.3, 55.0 (2C), 32.7, 13.0. HRMS (ESI): *m/z* [M+H]$^+$ calcd for $C_{31}H_{31}N_2O_6^+$ 527.2177, found 527.2170.

3'-deoxy-3'-α-benzylthio-5'-O-(4,4'-dimethoxytrityl)thymidine (**38**). Sodium thiobenzoate (15.0 g, 93.7 mmol) was added to a solution of **37** (6.7 g, 12.7 mmol) in anhydrous DMF (38 mL) and the reaction solution was stirred at 100 °C for 24 h. The reaction mixture was cooled to rt and diluted with EtOAc (100 mL). The solution was washed with saturated $NaHCO_3$ (2 x 90 mL) and brine (2 x 90 mL), dried with Na_2SO_4 and evaporated to dryness under a vacuum. The crude material was purified by flash silica column chromatography eluting with a gradient of 0 % to 2 % MeOH in DCM affording **38** (3.9 g, 46 %) as a pale-yellow solid. ^{1}H NMR (400 MHz, CD_3CN) δ 9.22 (s, 1H), 7.94 – 7.87 (m, 2H), 7.71 – 7.62 (m, 1H), 7.58 (d, J = 1.3 Hz, 1H), 7.54 – 7.43 (m, 4H), 7.35 – 7.15 (m, 7H), 6.84 – 6.78 (m, 4H), 6.18 (dd, J = 7.2, 4.7 Hz,

1H), 4.44 (q, *J* = 8.4 Hz, 1H), 4.13 – 4.09 (m, 1H), 3.71 (s, 3H), 3.70 (s, 3H), 3.44 – 3.32 (m, 2H), 2.70 (ddd, *J* = 13.7, 8.8, 4.7 Hz, 1H), 2.48 (ddd, *J* = 14.1, 8.2, 7.2 Hz, 1H), 1.56 (d, *J* = 1.3 Hz, 3H). $^{13}C\{^1H\}$ NMR (101 MHz, CD_3CN) δ 190.2, 163.8, 158.8, 158.7, 150.4, 144.9, 136.4, 135.8, 135.7, 135.6, 134.1, 130.1 (2C), 129.0 (2C), 128.0 (2C), 127.9 (2C), 127.1 (4C), 126.9, 113.1 (4C), 110.3, 86.5, 84.5, 83.6, 62.6, 54.9 (2C), 39.6, 38.3, 11.5. HRMS (ESI): *m/z* $[M+Na]^+$ calcd for $C_{38}H_{36}N_2NaO_7S^+$ 687.2135, found 687.2139.

3'-deoxy-3'-α-benzylthio-thymidine (**39**). **38** (3.8 g, 5.7 mmol) and mercaptoethanol (600 µl, 8.5 mmol) were dissolved in DCM (40 mL) and the solution was titrated with conc. HCl/MeOH (1:9) (200 µl) until complete detritylation was observed on TLC (10 % MeOH in DCM). The reaction solution was diluted with DCM (100 mL) and the organic phase was washed with saturated $NaHCO_3$ (80 mL) and brine (80 mL), dried with Na_2SO_4 and evaporated to dryness under a vacuum. The crude material was purified by flash silica column chromatography eluting with a gradient of 0 % to 3 % MeOH in DCM to afford **39** (2.0 g, 86 %) as pale-yellow solid. ^1H NMR (400 MHz, $(CD_3)_2SO$) δ 11.33 (s, 1H), 7.95 – 7.89 (m, 2H), 7.85 (d, *J* = 1.3 Hz, 1H), 7.76 – 7.66 (m, 1H), 7.61 – 7.54 (m, 2H), 6.16 (dd, *J* = 6.8, 5.6 Hz, 1H), 5.24 (app t, *J* = 5.3 Hz, 1H), 4.26 – 4.18 (m, 1H), 4.07 – 3.99 (m, 1H), 3.78 – 3.69 (m, 1H), 3.69 – 3.59 (m, 1H), 2.66 – 2.56 (m, 1H), 2.44 – 2.34 (m, 1H), 1.81 (d, *J* = 1.2 Hz, 3H). $^{13}C\{^1H\}$ NMR (101 MHz, $(CD_3)_2SO$) δ 190.8, 164.2, 150.9, 136.6, 136.4, 134.7, 129.7 (2C), 127.4 (2C), 109.9, 85.0, 84.3, 61.3, 40.3, 38.5, 12.8. HRMS (ESI): *m/z* $[M+H]^+$ calcd for $C_{17}H_{19}N_2O_5S^+$ 363.1009, found 363.1015.

3'-deoxy-3'-α-thiothymidine (**31**). **39** (1.9 g, 5.2 mmol) was dissolved in 40 % aqueous methylamine (45 mL) under nitrogen and the reaction solution was stirred at r.t. for 16 h. Ethanol (20 mL) was added, and the resulting mixture was concentrated under a vacuum. The crude material was purified by flash silica column chromatography eluting with gradient of 0 % to 10 % MeOH in DCM affording **31** (1.1 g, 81 %) as a white solid. ^1H NMR (400 MHz, MeOD) δ 7.95 (d, *J* = 1.2 Hz, 1H), 6.13 (dd, *J* = 7.2, 2.9 Hz, 1H), 3.95 (m, 1H), 3.90 – 3.76 (m, 3H), 3.44 (ddd, *J* = 10.2, 8.8, 7.8 Hz, 1H), 2.55 (ddd, *J* = 13.7, 7.8, 2.9 Hz, 1H), 2.37 (ddd, *J* = 13.8, 10.2, 7.2 Hz, 1H), 1.89 (d, *J* = 1.2 Hz, 3H). $^{13}C\{^1H\}$ NMR (101 MHz, MeOD) δ 166.5, 152.3, 138.3, 111.0, 90.6, 85.7, 60.4, 44.1, 34.6, 12.5. HRMS (ESI): *m/z* $[M+H]^+$ calcd for $C_{10}H_{15}N_2O_4S^+$ 259.0747, found 259.0755.

6.2 Synthesis of 2'-amino-2',3'-dideoxyuridine (32)

The intermediate **43** was synthesized form 5'-(4,4'-dimethoxytrityl)uridine using a reported procedure[20] followed by Staudinger reduction to afford **32** (Scheme S2).

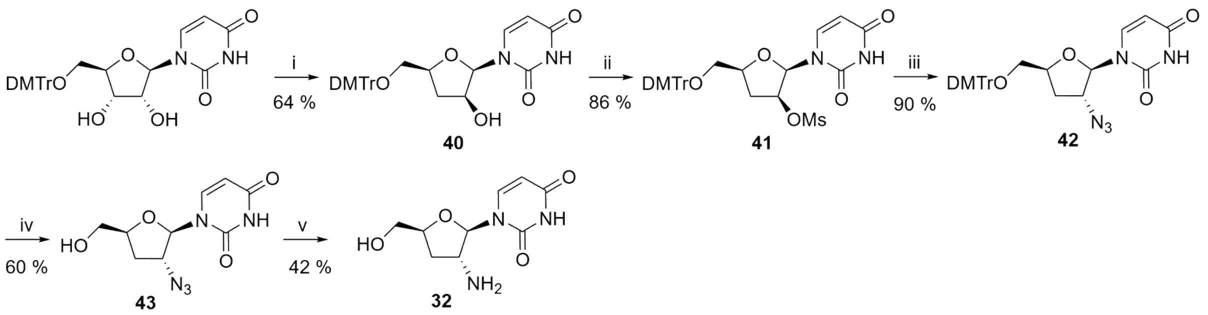

Scheme S2. Synthesis of 32. Conditions: i) (1) pivaloyl chloride, pyridine, 0 °C, 1h, (2) MsCl, 0 °C, 1 h, (3) $NaBH_4$, KOH, MeOH, 0 °C, 16 h; ii) MsCl, pyridine, rt 16 h; iii) NaN_3, DMF, 110 °C , 16 h; iv) conc. HCl/MeOH, $HO(CH_2)_2SH$, DCM, rt 30 min; v) PPh_3, 1,4-dioxane/H_2O, rt, 16 h.

1-(3-deoxy-5-O-(4,4'-dimethoxytrityl)-β-D-threo-pentofuranosyl)-uracil (**40**). 5'-(4,4'-dimethoxytrityl)uridine (5.9 g, 10.8 mmol) was dissolved in anhydrous pyridine (64 mL) under nitrogen and the resulting solution was cooled to 0 °C. To this solution, pivaloyl chloride (2.3 mL, 18.4 mmol) was added dropwise, and the resulting mixture was stirred at 0 °C for 1 h. Methanesulfonyl chloride (3.4 mL, 43.3 mmol) was added and stirring was continued at 0 °C for 1h and then at rt for further 3 h. The reaction was quenched by adding 50 % aqueous pyridine (4.3 mL) at 0 °C and the mixture was partitioned between chloroform (300 mL) and water (100 mL). The organic phase was washed with saturated $NaHCO_3$ (80 mL) and brine (80 mL), dried with Na_2SO_4 and evaporated to dryness under a vacuum. The crude material was dissolved in MeOH (43 mL), and the solution was cooled to 0 °C followed by addition of a 1 M solution of KOH in MeOH (32 ml, 32 mmol) and $NaBH_4$ (820 mg, 21.7 mmol). The reaction mixture was stirred at rt for 16 h and quenched by adjusting the pH to 7 – 8 with MeOH/conc. HCl (9:1 *v*/*v*). To the mixture was added DCM (200 mL) and the organic phase was washed with water (3 x 50 mL), dried with Na_2SO_4 and evaporated to dryness under a vacuum. The crude material was purified by flash silica column chromatography eluting with a gradient of 1 % to 5 % MeOH in DCM containing 0.2 % triethylamine to afford **40** (3.7 g, 64 %) as a white solid. ^{1}H NMR (400 MHz, DMSO) δ 11.25 (s, 1H), 7.56 (d, J = 8.1 Hz, 1H), 7.44 – 7.17 (m, 9H), 6.93 – 6.87 (m, 3H), 5.91 (d, J = 4.9 Hz, 1H), 5.37 (d, J = 4.6 Hz, 1H), 5.35 (d, J = 8.1 Hz, 1H), 4.37 – 4.29 (m, 1H), 4.19 – 4.11 (m, 1H), 3.74 (s, 6H), 3.28 – 3.22 (m, 1H), 3.21 – 3.13 (m, 1H), 2.33 – 2.23 (m, 1H), 1.78 – 1.68 (m, 1H). ^{13}C{^{1}H} NMR (101 MHz, DMSO) δ 163.2, 158.1 (2C), 150.5, 144.8, 142.0, 135.5, 135.4, 129.7 (2C), 129.7 (2C), 127.9 (2C), 127.7 (2C), 126.7, 113.2 (4C), 99.8, 85.6, 85.5, 75.8, 69.2, 65.0, 55.0 (2C), 34.6. HRMS (ESI): *m/z* $[M+H]^+$ calcd for $C_{30}H_{31}N_2O_7^+$ 531.2126, found 531.2112.

1-(3-deoxy-5-O-(4,4'-dimethoxytrityl)-2-O-mehanesulfonyl-b-D-erythro-pentofuranosyl)-uracil (**41**). Under nitrogen, methanesulfonyl chloride (1.6 mL, 20.5 mmol) was added to a solution of **40** (3.6 g, 6.8 mmol) in anhydrous pyridine (20 mL) and the mixture was stirred at rt for 16 h. The reaction was quenched at 0 °C with 50 % aqueous pyridine (7 mL). The mixture was partitioned between DCM (50 mL) and water (30 mL) and the organic phase was washed with saturated $NaHCO_3$ (30 mL) and brine (30 mL), dried with Na_2SO_4 and evaporated to dryness under a vacuum. The crude material was purified by flash silica column chromatography eluting with a gradient from 0 % to 2 % MeOH in DCM containing 0.2 % triethylamine to afford **41** (3.58 g, 86 %) as a pale-yellow solid. ^{1}H NMR (400 MHz, DMSO) δ 11.39 (d, *J* = 2.2 Hz, 1H), 7.53 (d, *J* = 8.1 Hz, 1H), 7.44 – 7.30 (m, 4H), 7.30 – 7.22 (m, 5H), 6.96 – 6.86 (m, 4H), 6.12 (d, *J* = 4.8 Hz, 1H), 5.44 (dd, *J* = 8.1, 2.2 Hz, 1H), 5.34 (dt, *J* = 6.9, 4.9 Hz, 1H), 4.30 – 4.21 (m, 1H), 3.74 (s, 6H), 3.26 (d, *J* = 4.5 Hz, 2H), 3.13 (s, 3H), 2.65 – 2.54 (m, 1H), 2.16 – 2.05 (m, 1H). ^{13}C{^{1}H} NMR (101 MHz, DMSO) δ 162.9, 158.1 (2C), 150.2, 144.7, 140.6, 135.4, 135.3, 129.8 (2C), 129.7 (2C), 127.9 (2C), 127.7 (2C), 126.8, 113.3 (4C), 101.0, 85.7, 83.6, 77.7, 75.7, 64.2, 55.0 (2C), 37.5, 32.5. HRMS (ESI): *m/z* $[M+H]^+$ calcd for $C_{31}H_{33}N_2O_9S^+$ 609.1901, found 609.1910.

1-(2-Azido-2,3-dideoxy-5-O-(4,4'-dimethoxytrityl)-β-D-erythro-pentofuranosyl)-uracil (**42). **Under nitrogen, sodium azide (1.1 g, 17.2 mmol) was added to a solution of **41** (3.5 g, 5.7 mmol) in anhydrous DMF (50 mL), and the resulting mixture was stirred at 110 °C for 16 h. After cooling to rt, the mixture was diluted with a mixture of Et_2O (300 mL) and DCM (60 mL). The organic solution was washed with water (5 x 100 mL) and dried with Na_2SO_4 and evaporated to dryness under a vacuum. The crude material was purified by flash silica column chromatography eluting with a gradient of 0 % to 1.4 % MeOH in DCM containing 0.2 % triethylamine to afford **42** (2.9 g, 90 %) as a pale-yellow solid. ^{1}H NMR (400 MHz, DMSO) δ 11.42 (s, 1H), 7.75 (d, *J* = 8.1 Hz, 1H), 7.44 – 7.18 (m, 9H), 6.93 – 6.88 (m, 4H), 5.76 (d, *J* = 1.6 Hz, 1H), 5.23 (d, *J* = 8.1 Hz, 1H), 4.55 (dt, *J* = 6.3, 1.7 Hz, 1H), 4.38 – 4.28 (m, 1H), 3.74 (s, 6H), 3.34 – 3.24 (m, 2H), 2.26 – 2.16 (m, 1H), 2.02 – 1.93 (m, 1H). ^{13}C{^{1}H} NMR (101 MHz, DMSO) δ 163.6, 158.6 (2C), 150.7, 145.1, 140.1, 135.8, 135.5, 130.2, 128.4 (2C), 128.2 (2C), 127.3, 113.8 (2C), 113.7 (2C), 101.5, 90.2, 86.4, 80.2, 66.1, 63.7, 55.5 (2C), 31.2. HRMS (ESI): *m/z* $[M+Na]^+$ calcd for $C_{30}H_{29}N_5NaO_6^+$ 578.2010, found 578.2057.

1-(2-Azido-2,3-dideoxy-β-D-erythro-pentofuranosyl)-uracil (**43**). **42** (2.7 g, 4.86 mmol) was dissolved in DCM and treated with mercaptoethanol (445 µl, 6.3 mmol) and MeOH/conc. HCl (9:1 *v*:*v*, 400 µl). The reaction solution was stirred at r.t. for 30 min, and then diluted with DCM (45 mL). The organic phase was extracted with H_2O (5 x 50 mL), and the aqueous phase was

concentrated under a vacuum. The crude material was dry loaded to flash silica column and purified by eluting with a gradient of 1 % to 10 % MeOH in DCM to afford **43** (740 mg, 60 %) as a white solid. ^{1}H NMR (400 MHz, DMSO) δ 11.37 (s, 1H), 7.99 (d, *J* = 8.1 Hz, 1H), 5.74 (d, *J* = 1.9 Hz, 1H), 5.60 (d, *J* = 8.1 Hz, 1H), 5.18 (t, *J* = 5.2 Hz, 1H), 4.48 (dt, *J* = 6.4, 2.3 Hz, 1H), 4.25 – 4.17 (m, 1H), 3.80 – 3.71 (m, 1H), 3.58 – 3.50 (m, 1H), 2.16 – 2.04 (m, 1H), 1.94 – 1.84 (m, 1H). ^{13}C{^{1}H} NMR (101 MHz, DMSO) δ 163.3, 150.3, 139.8, 101.1, 89.3, 81.4, 65.7, 60.9, 30.0. HRMS (ESI): *m/z* [M+H]$^+$ calcd for $C_9H_{12}N_5O_4^+$ 254.0884, found 254.0899.

2'-amino-2',3'-dideoxyuridine (**32**). Triphenylphosphine (1.38 g, 5.25 mmol) was added to a solution of **43** (700 mg, 2.8 mmol) in a mixture of 1,4-dioxane (12 mL) and H_2O (3 mL) and the reaction mixture was stirred at rt for 16 h. The mixture was partitioned between DCM and H_2O, and the aqueous phase was concentrated under a vacuum. The crude material was purified by flash C18 column chromatography eluting with gradient of 5 % to 20 % MeCN in H_2O affording **32** (281 mg, 42 %) as a colourless oil. ^{1}H NMR (600 MHz, D_2O) δ 7.89 (d, *J* = 8.1 Hz, 1H), 5.87 (d, *J* = 8.1, 1H), 5.76 (d, *J* = 3.6 Hz, 1H), 4.50 (m, 1H), 3.87 (dd, *J* = 12.5, 3.1 Hz, 1H), 3.75 – 3.66 (m, 2H), 2.13 (dt, *J* = 13.2, 7.8 Hz, 1H), 2.00 – 1.92 (m, 1H). ^{13}C{^{1}H} NMR (151 MHz, D_2O) δ 166.8, 152.1, 141.5, 101.7, 92.0, 80.5, 62.7, 56.0, 32.8. HRMS (ESI): *m/z* [M+H]$^+$ calcd for $C_9H_{14}N_3O_4^+$ 228.0979, found 228.0984.

6.3 Synthesis of 2'-amino-2'-deoxyuridine (33)

Intermediate **45** was synthesized from 2'-anhydrouridine according to literature protocol.[21] Deprotection of **45** in acidic conditions afforded **33** (Scheme S3).

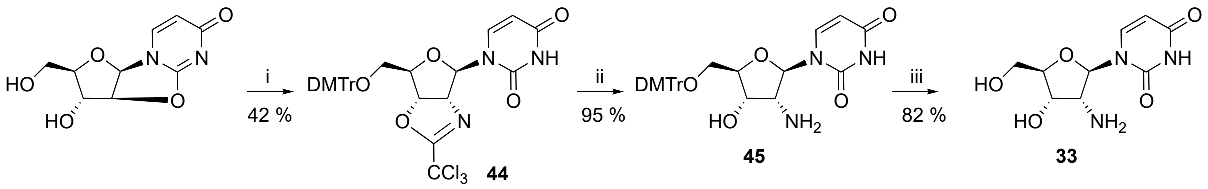

Scheme S3. Synthesis of **33**. Conditions: i) (1) DMTrCl, DMAP, Py/DMF, rt, 16 h, (2) CCl_3CN, TEA, 95 °C, 18 h, ii) (1) NaOH, EtOH, 100 °C, 16 h, iii) TCA, $HO(CH_2)_2SH$, DCM, rt 1 h.

5'-O-(4,4'-dimethoxytrityl)-2'-N,3'-O-(2-(trichloromethyl)oxazolino-2'-deoxy-1-(β-D-ribofuranosyl)uracil (**44**). Under nitrogen, 2,2'-anhydrouridine (13.1 g, 57.8 mmol), 4,4'-dimethoxytrityl chloride (20.6 g, 60.7 mmol) and DMAP (0.7 g, 5,8 mmol) were dissolved in a mixture of anhydrous pyridine (44 mL) and anhydrous DMF (35 mL) and the resulting mixture was stirred at rt for 16 h. The reaction mixture was concentrated under a vacuum and the residue was partitioned between DCM (75 mL) and water (75 mL). The organic phase was

separated and washed with saturated $NaHCO_3$ (2 x 60 mL) and brine (2 x 60 mL), dried with Na_2SO_4 and concentrated to dryness under a vacuum and co-evaporated from toluene. Under nitrogen, the residue was dissolved in trichloroacetonitrile (40 mL) and triethylamine (1.1 mL, 7.5 mmol) was added. After refluxing the reaction solution at 95 °C for 18 h, the mixture was concentrated to a dark oil, which was dry loaded to a flash silica gel column. The product was purified by eluting with a gradient of 40 % to 60 % ethyl acetate in hexane containing 0.2 % triethylamine to afford **44** (16.3 g, 42 %) as pale-yellow solid. ^1H NMR (400 MHz, $(CD_3)_2SO$) δ 11.42 (s, 1H), 7.83 (d, *J* = 8.0 Hz, 1H), 7.50 – 7.34 (m, 2H), 7.29 – 7.18 (m, 7H), 6.94 – 6.81 (m, 4H), 5.91 (d, *J* = 2.2 Hz, 1H), 5.63 (d, *J* = 8.0 Hz, 1H), 5.45 (dd, *J* = 8.4, 4.5 Hz, 1H), 5.29 (dd, *J* = 8.4, 2.2 Hz, 1H), 4.14 (app dt, *J* = 8.1, 4.1 Hz, 1H), 3.73 (s, 3H), 3.72 (s, 3H), 3.48 (dd, *J* = 10.2, 7.7 Hz, 1H), 3.19 (dd, *J* = 10.2, 3.8 Hz, 1H).$^{13}C\{^1H\}$ NMR (101 MHz, $(CD_3)_2SO$) δ 163.2, 161.5, 158.1, 158.1, 150.2, 144.8, 143.6, 135.3, 135.3, 129.7 (2C), 129.6 (2C), 127.8 (2C), 127.6 (2C), 126.7, 113.2 (4C), 101.8, 93.3, 86.7, 86.2, 85.7, 76.5, 63.9, 55.1, 55.0, 54.9. HRMS (ESI): *m/z* $[M+Na]^+$ calcd for $C_{32}H_{28}{}^{35}Cl_3N_3NaO_7{}^+$ 694.0891, found 694.0888 (see figure S143 for isotopic pattern).

2'-amino-5'-O-(4,4'-dimethoxytrityl)-2'-deoxyuridine (**45**). 6 M NaOH solution (30 mL, 180 mmol) was added dropwise to a solution of **44** (16.3 g, 24.2 mmol) in ethanol (60 mL), and the resulting mixture was refluxed at 100 °C for 16 h. The reaction solution was cooled to rt and evaporated to dryness under a vacuum. The residue was taken up in DCM (240 mL) and saturated ammonium chloride (150 mL). The organic phase was separated, and the aqueous phase was extracted with DCM (2 x 100 mL). The combined organic phases were dried with Na_2SO_4 and evaporated to dryness under a vacuum. The crude material was purified by flash silica column chromatography eluting with 1-5 % MeOH in DCM containing 0.2 % triethylamine, affording the product **45** (12.6 g, 95 %) as white solid. ^1H NMR (400 MHz, MeOD) δ 7.81 (d, *J* = 8.1 Hz, 1H), 7.44 – 7.37 (m, 2H), 7.35 – 7.18 (m, 7H), 6.91 – 6.83 (m, 4H), 5.86 (d, *J* = 6.9 Hz, 1H), 5.32 (d, *J* = 8.1 Hz, 1H), 4.29 (dd, *J* = 5.5, 2.9 Hz, 1H), 4.11 (q, *J* = 3.1 Hz, 1H), 3.77 (s, 6H), 3.53 (dd, *J* = 6.9, 5.5 Hz, 1H), 3.46 – 3.34 (m, 2H). $^{13}C\{^1H\}$ NMR (101 MHz, MeOD) δ 166.1, 160.3 (2C), 152.7, 146.0, 142.2, 136.8, 136.6, 131.4 (4C), 129.4 (2C), 128.9 (2C), 128.1, 114.2 (2C), 114.2 (2C), 102.8, 90.3, 88.3, 86.4, 72.7, 64.7, 59.8, 55.7 (2C). HRMS (ESI): *m/z* $[M+Na]^+$ calcd for $C_{30}H_{32}N_3O_7{}^+$ 546.2235, found 546.2270.

2'-amino-2'-deoxyuridine (**33**). **45** (500 mg, 0.9 mmol) was dissolved in DCM containing 3 % trichloroacetic acid (12 mL) and mercaptoethanol (65 µl, 0.9 mmol) was added. The mixture was stirred at rt for 1 h. The mixture was diluted with DCM (20 mL), neutralized with triethylamine and concentrated under a vacuum. The residue was partitioned between DCM (15 mL) and H_2O (15 mL) and the separated aqueous phase was washed with DCM (2 x 5

mL). Evaporating the aqueous phase to dryness under a vacuum afforded **33** (183 mg, 82 %) as white solid. ^{1}H NMR (400 MHz, D2O) δ 7.89 (d, *J* = 8.2 Hz, 1H), 6.22 (d, *J* = 6.6 Hz, 1H), 5.94 (d, *J* = 8.1 Hz, 1H), 4.58 (dd, *J* = 6.3, 3.4 Hz, 1H), 4.29 (app q, *J* = 3.6 Hz, 1H), 4.12 (t, *J* = 6.4 Hz, 1H), 3.99 – 3.75 (m, 2H). ^{13}C NMR (101 MHz, D2O) δ 166.0, 151.7, 141.3, 102.7, 86.6, 86.5, 68.9, 60.7, 55.9. HRMS (ESI): *m/z* [M+Na]$^+$ calcd for $C_9H_{14}N_3O_5^+$ 244.0928, found 244.0934.

7. Appendix

6-bromo-deaza-ribosyl-purine (10)

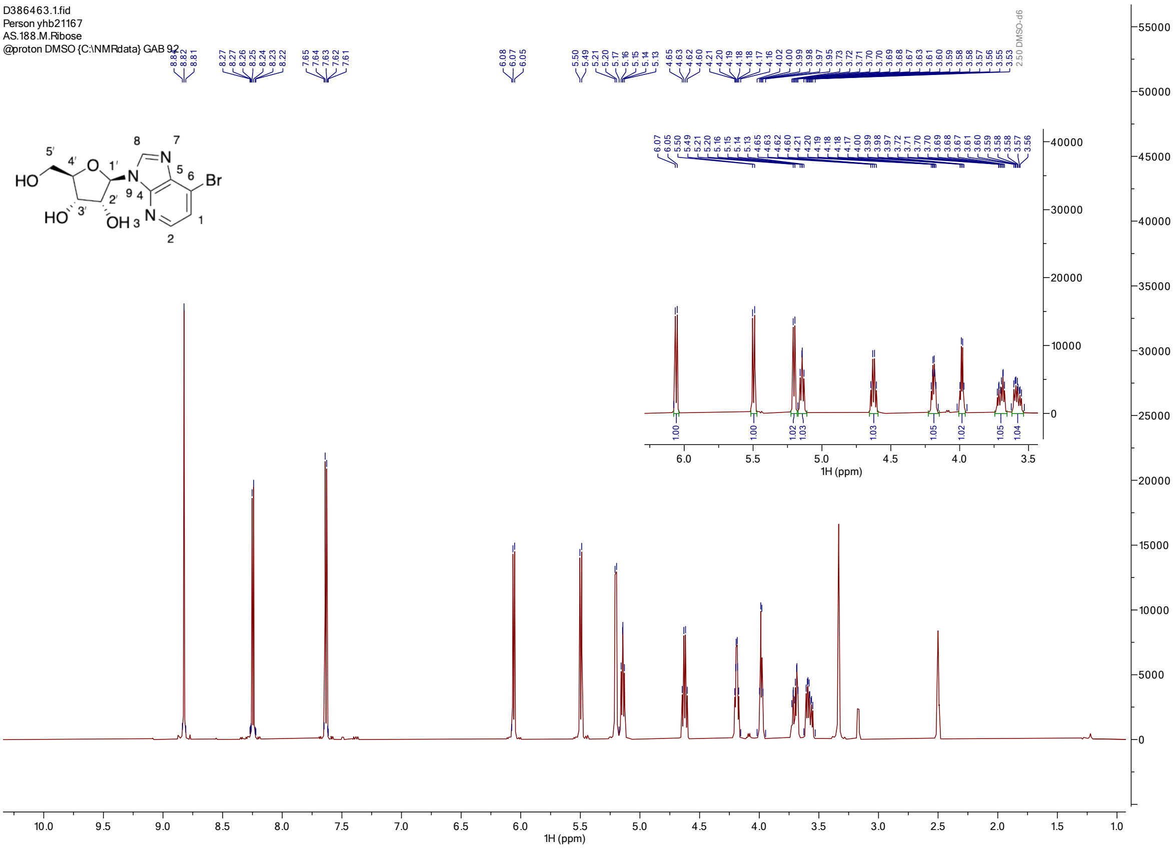

Figure S5 – ^{1}H NMR of compound 10.

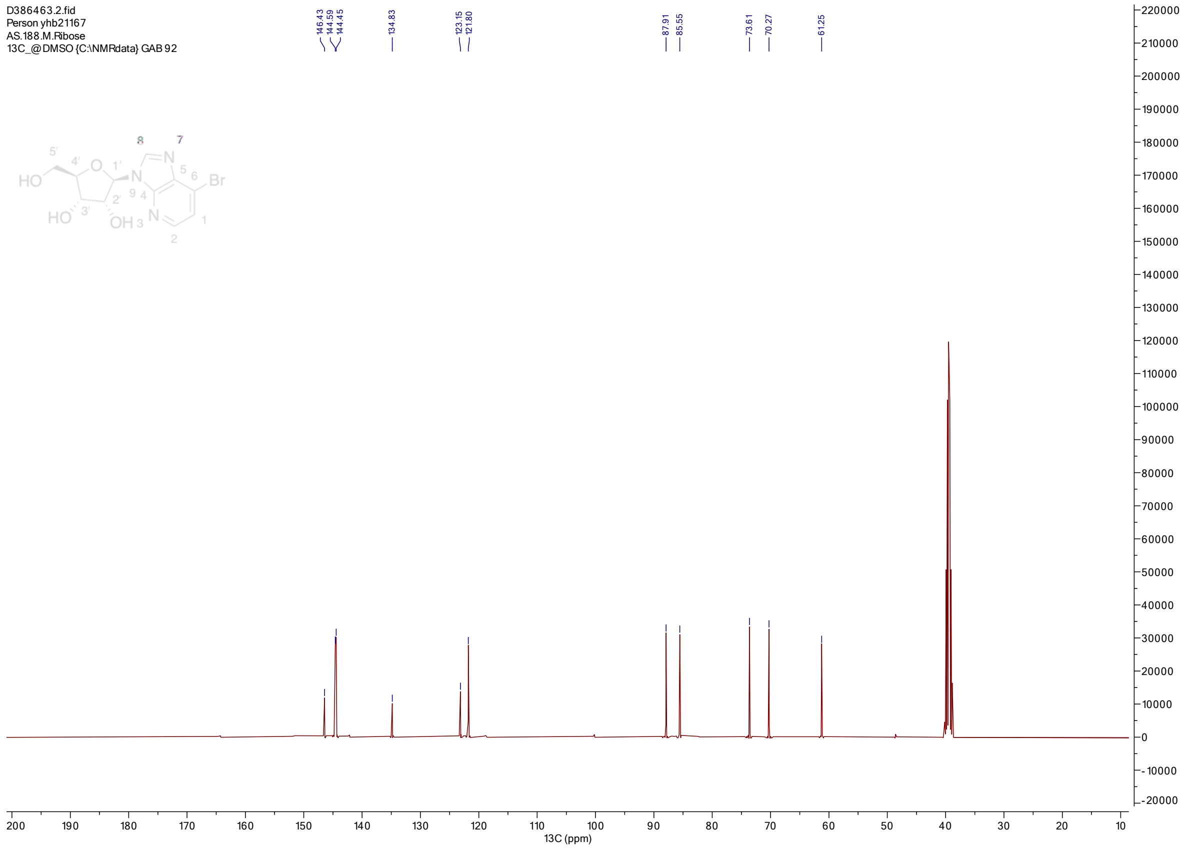

Figure S6 – ^{13}C NMR of compound 10.

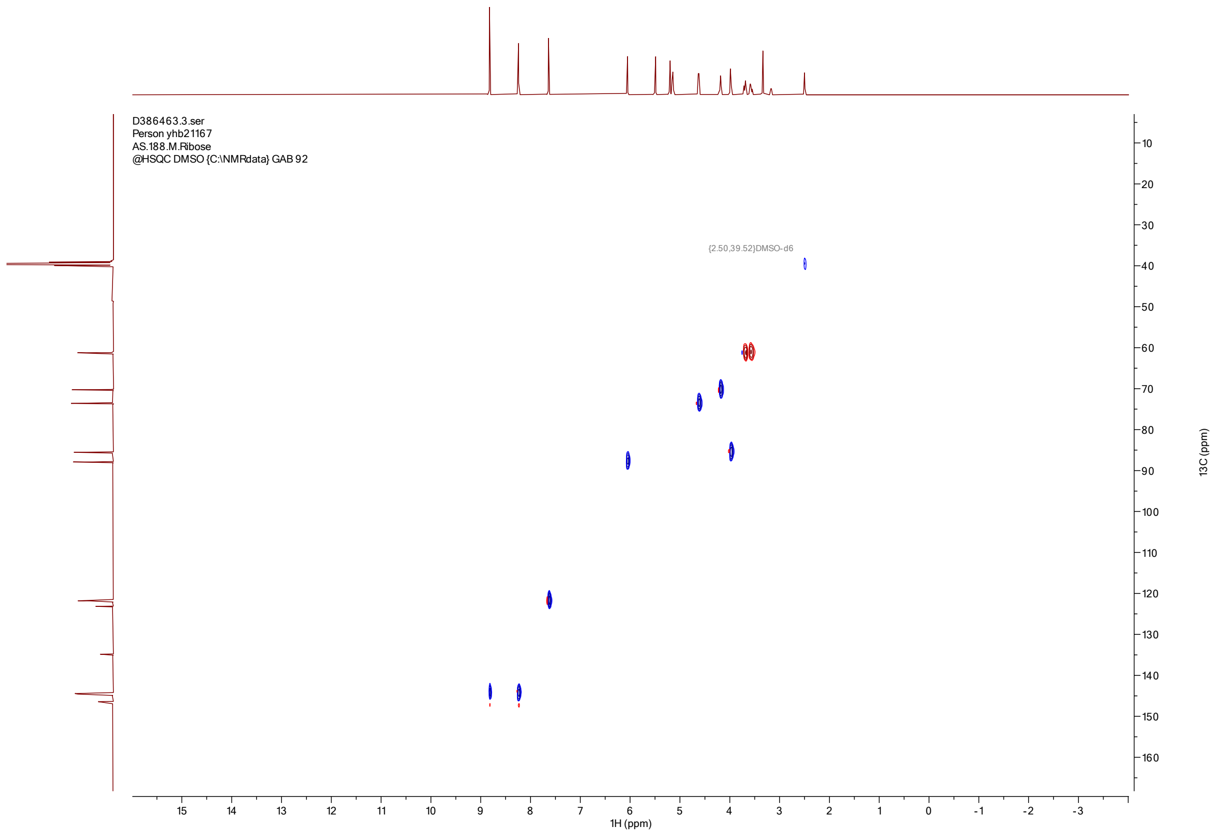

Figure S7- HSQC of compound 10.

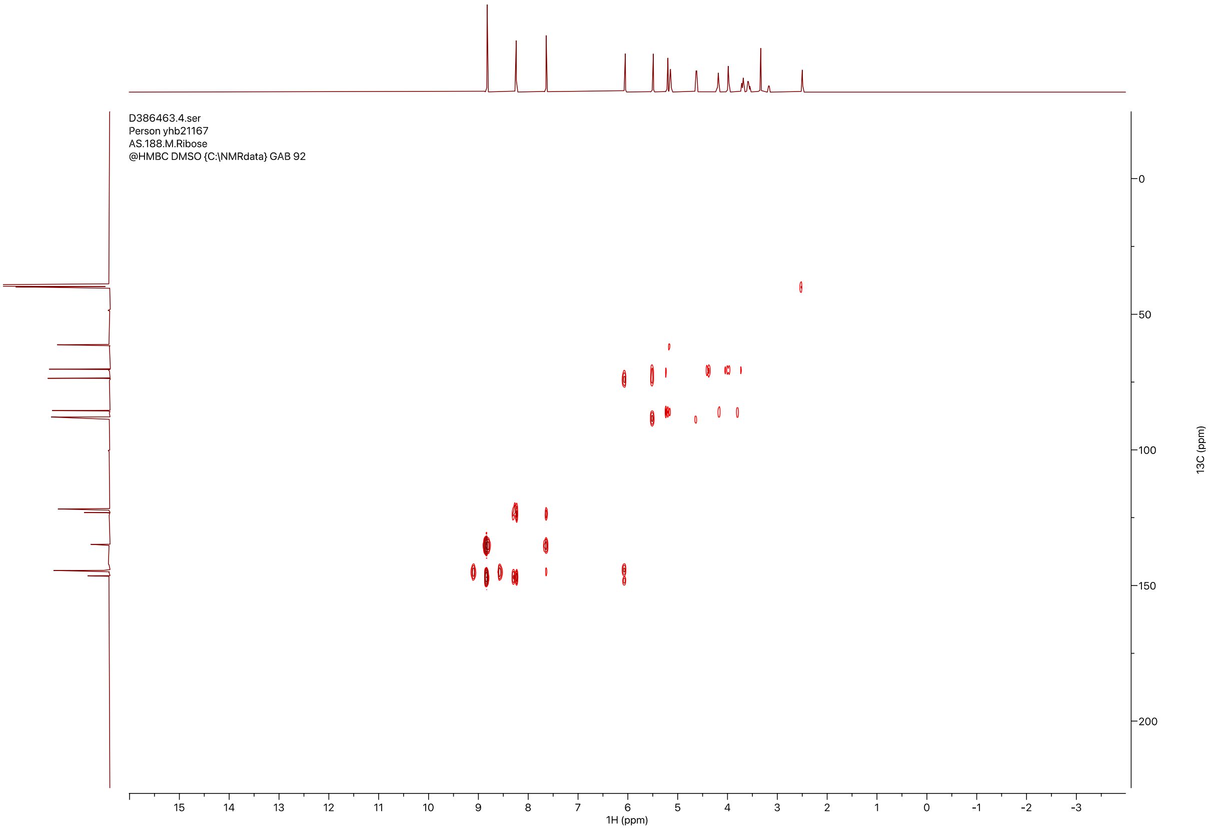

Figure S8 – HMBC of compound 10.

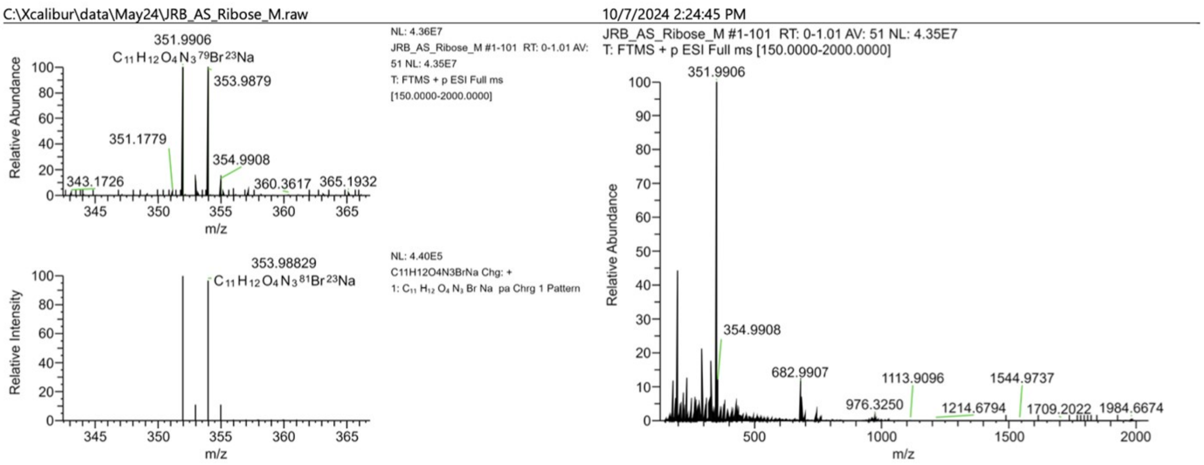

Peak Mass	Display For...	S Fit	RDB	Delta [ppm]	Theo. mass	Rank	Combined S...	# Matched I...	# Missed Iso.	MS Cov. [%]	Pattern Cov....	MSMS Matc...
351.9906	$C_{11}H_{12}O_4N_3{}^{79}Br^{23}Na$	65.406605896759	6.50	0.81	351.99034	1	93.73	6	0	95.3	100	(Collection)
351.9906	$C_9H_7O_2N_9{}^{79}Br$	46.3957557984132	10.50	1.61	351.99006	2	92.2	5	0	94.74	99.65	(Collection)
351.9906	$C_{10}H_{13}O_7N_2{}^{79}Br$	40.2030509438763	5.00	1.59	351.99006	3	91.87	6	0	94.74	99.12	(Collection)
351.9906	$C_9H_{10}O_3N_6{}^{79}Br^{23}Na$	32.3679644934851	7.00	4.63	351.98900	4	91.46	5	0	94.74	99.56	(Collection)
351.9906	$C_{11}H_9O_3N_6{}^{79}Br$	28.0815036512167	10.00	-2.20	351.99140	5	86.6	5	0	89.85	94.16	(Collection)
351.9906	$C_{12}H_8N_7{}^{79}Br^{23}Na$	23.1064485172512	11.50	-2.99	351.99168	6	86.34	5	0	89.85	93.94	(Collection)
351.9906	$C_{13}H_{14}O_5{}^{79}Br^{23}Na$	17.1763515836112	6.00	-3.00	351.99168	7	86.03	6	0	89.85	93.05	(Collection)
351.9906	$C_{10}H_8O_{14}$	28.3072404190904	7.00	-0.65	351.99086	8	46.69	2	2	47.71	96.88	(Collection)
351.9906	$C_{10}HO_6N_8{}^{23}Na$	27.1389775787979	14.00	-1.42	351.99113	9	46.63	2	2	47.71	98.37	(Collection)
351.9906	$C_{11}H_7O_{11}N^{23}Na$	26.6416896961059	8.50	-1.43	351.99113	10	46.6	2	2	47.71	97.39	(Collection)

Figure S9 – HRMS of compound 10.

Sample Name : m
Sample ID :
Data Filename : runs_23032024_008.lcd
Method Filename : Admir Trans glyco Run.lcm
Batch Filename : runs.lcb
Vial # : 1-23 Sample Type : Unknown
Injection Volume : 10 uL
Date Acquired : 23/03/2024 15:14:09 Acquired by : Shimadzu
Date Processed : 23/03/2024 15:25:29 Processed by : Shimadzu

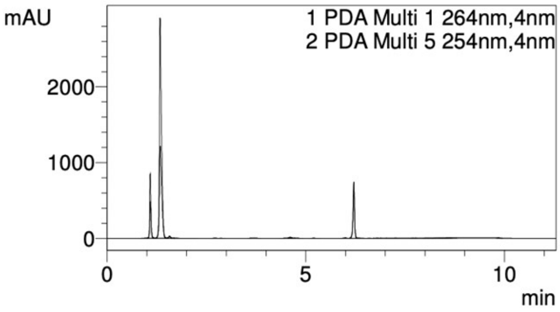

PDA Ch1 264nm

Peak#	Ret. Time	Area	Height	ID#
1	0.886	1540	699	
2	0.958	3889	1333	
3	1.088	1860021	813180	
4	1.337	10447252	2822394	
5	1.574	99030	27400	
6	1.743	5743	793	
7	1.968	2957	342	
8	2.123	1645	253	
9	2.721	3720	597	
10	2.865	1067	196	
11	3.737	20431	3503	
12	4.613	76171	14509	
13	4.790	6701	1496	
14	5.206	2548	563	
15	6.001	13726	3722	
16	6.212	1706204	653866	
17	6.589	1166	331	
18	6.799	2741	771	
19	7.036	2090	430	
20	7.261	4380	1264	
21	7.690	1726	644	
22	7.768	1883	782	

Figure S10 – HPLC trace of the reaction forming compound 10.

5-Iodouridine (18)

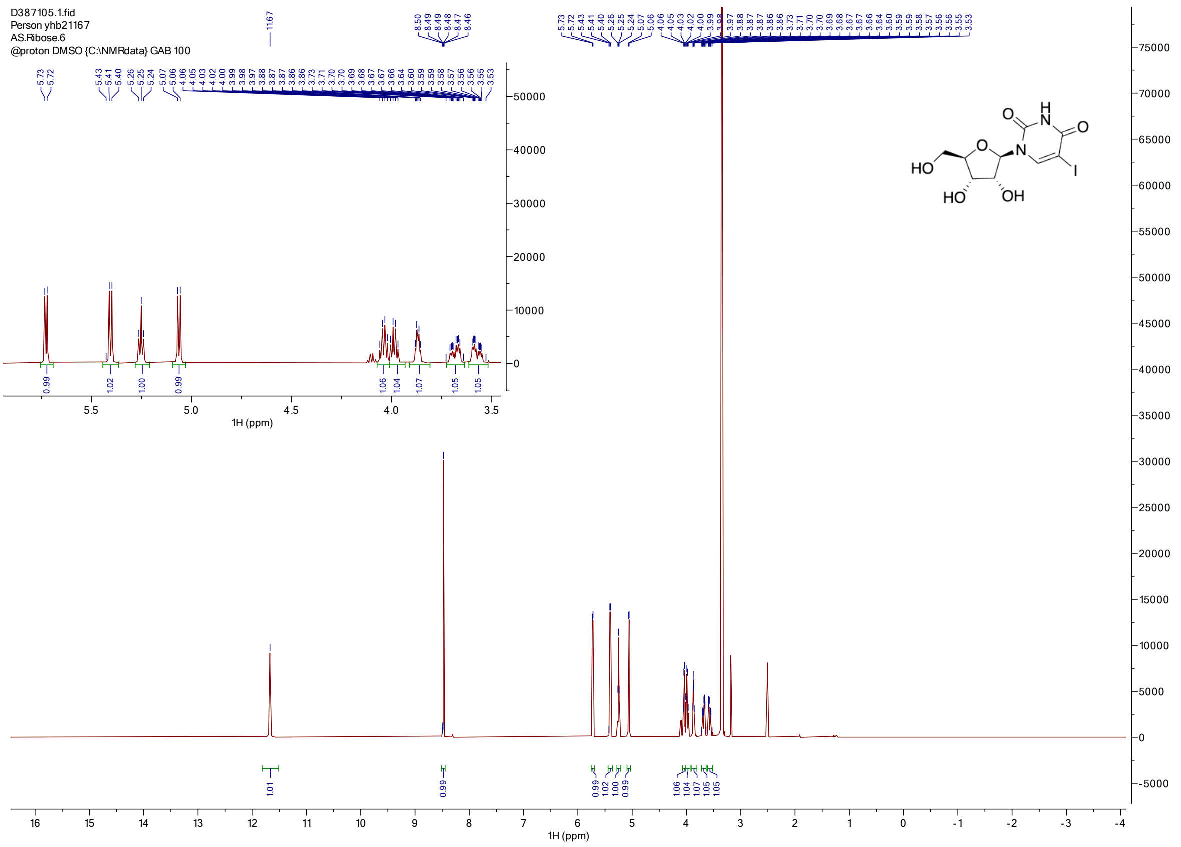

Figure S11 – ^{1}H NMR of compound 18

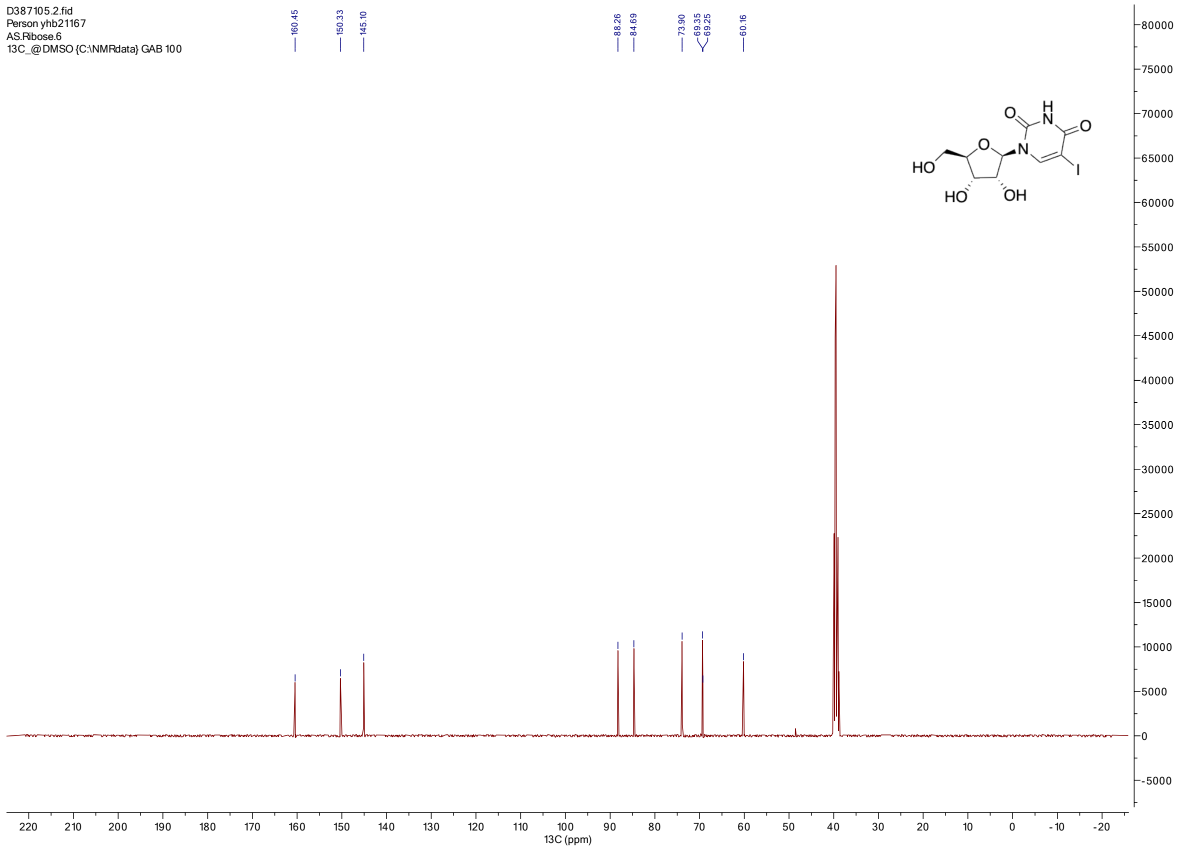

Figure S12 – ^{13}C NMR of compound 18

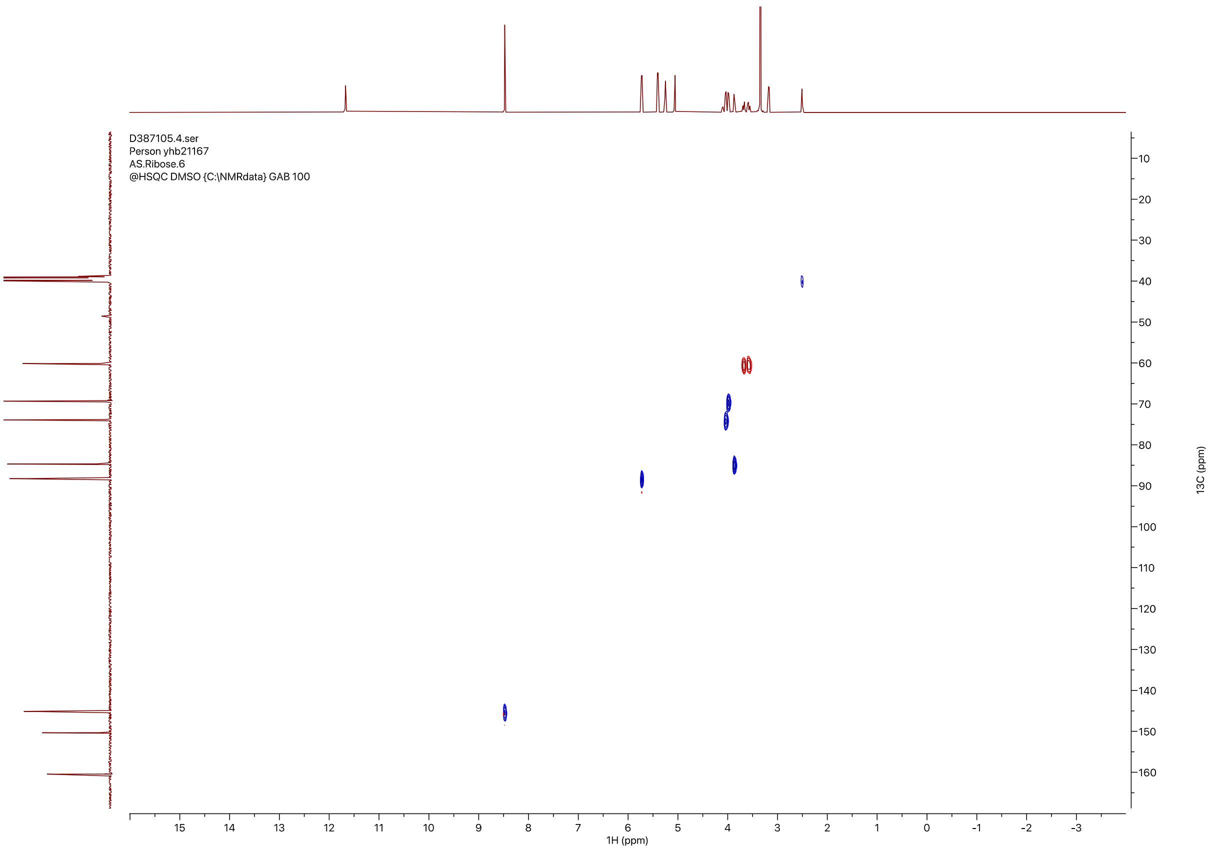

Figure S13 – HSQC of compound 18

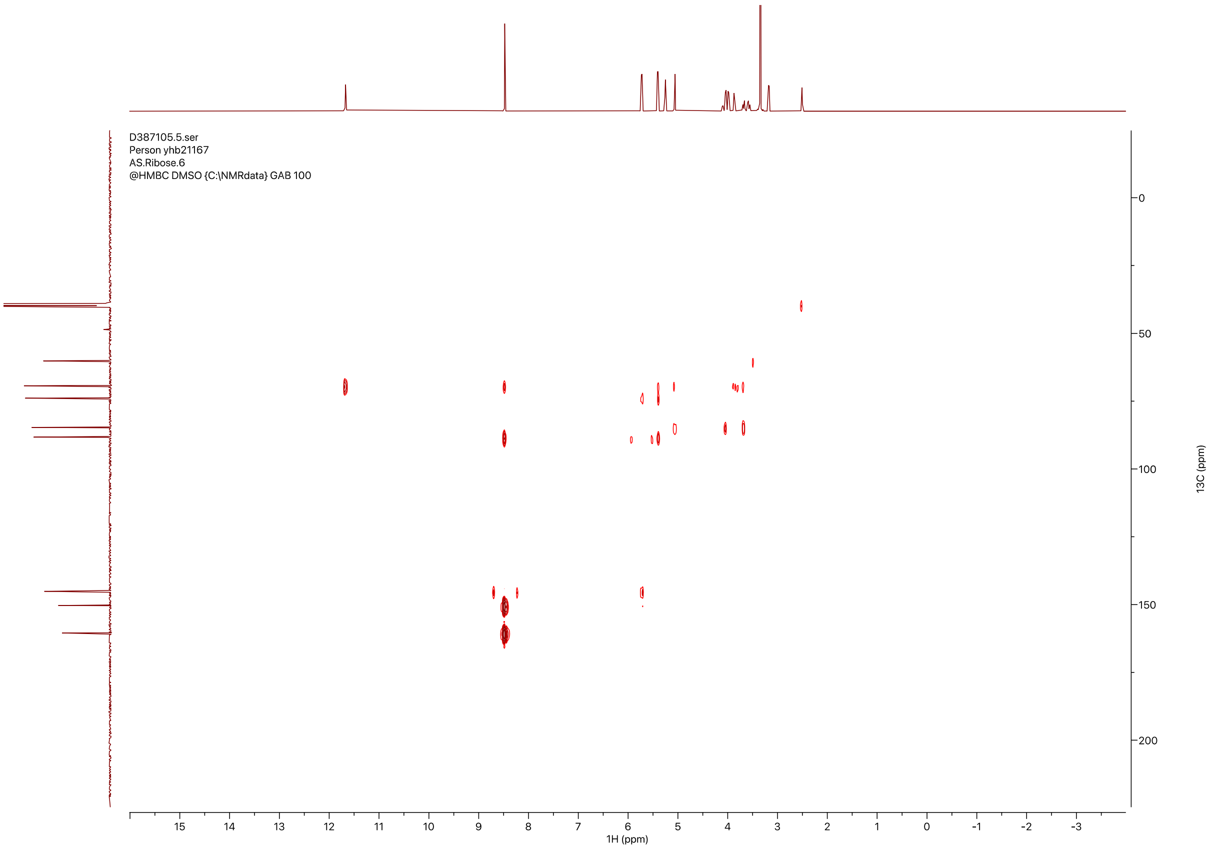

Figure S14 – HMBC of compound 18.

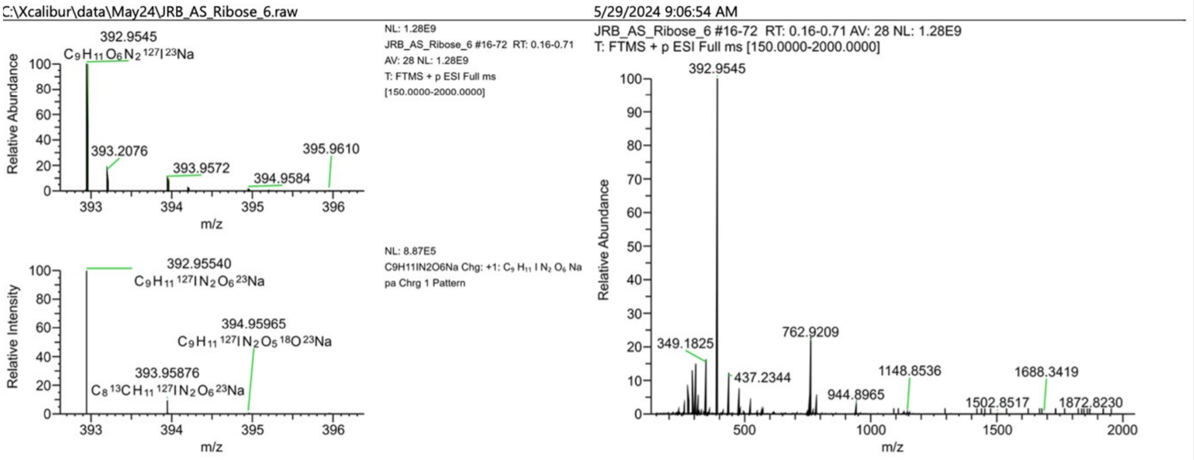

Peak Mass	Display For...	S Fit	RDB	Delta [ppm]	Theo. mass	Rank	Combined S...	# Matched I...	# Missed Iso.	MS Cov. [%]	Pattern Cov....	MSMS Matc...
392.9545	$C_7H_9O_5N_5{}^{127}I{}^{23}Na$	56.9524898269364	5.00	1.25	392.95406	1	84.33	3	0	85.85	100	(Collection)
392.9545	$C_9H_{11}O_6N_2{}^{127}I{}^{23}Na$	32.5768491562261	4.50	-2.17	392.95540	2	83.04	4	0	85.85	99.89	(Collection)
392.9545	$C_8H_5ON_9{}^{127}I{}^{23}Na$	33.5005266281784	10.00	-2.16	392.95540	3	81.97	2	1	84.67	99.48	(Collection)
392.9545	$C_7H_6O_4N_8{}^{127}I$	30.9200192723284	8.50	-1.46	392.95512	4	81.84	3	0	84.67	98.99	(Collection)
392.9545	$C_8O_{11}N_7{}^{23}Na$	27.5222170973524	12.00	-0.64	392.95480	5	81.66	4	0	84.67	97.48	(Collection)
392.9545	$C_7HO_{14}N_6$	25.3659880312483	10.50	0.06	392.95453	6	81.55	4	0	84.67	96.96	(Collection)
392.9545	$C_8H_{12}O_9N{}^{127}I$	23.9856427796847	3.00	-1.47	392.95513	7	81.47	4	0	84.67	97.95	(Collection)
392.9545	$C_7H_4O_{15}N_3{}^{23}Na$	22.6608315542822	7.00	2.76	392.95346	8	81.4	4	0	84.67	96.8	(Collection)
392.9545	$C_6H_{10}O_8N_4{}^{127}I$	40.6765490600966	3.50	1.94	392.95378	9	76.33	4	0	78.31	93.55	(Collection)
392.9545	$C_6H_{13}O_9N{}^{127}I{}^{23}Na$	20.6228424829234	0.00	4.65	392.95272	10	74.16	4	0	77.13	91.79	(Collection)

Figure S15 – HRMS of compound 18.

Sample Name	: 6		
Sample ID	:		
Data Filename	: runs_25032024_004.lcd		
Method Filename	: Admir Trans glyco Run.lcm		
Batch Filename	: runs.lcb		
Vial #	: 1-64	Sample Type	: Unknown
Injection Volume	: 10 uL		
Date Acquired	: 25/03/2024 11:34:50	Acquired by	: Shimadzu
Date Processed	: 25/03/2024 11:46:10	Processed by	: Shimadzu

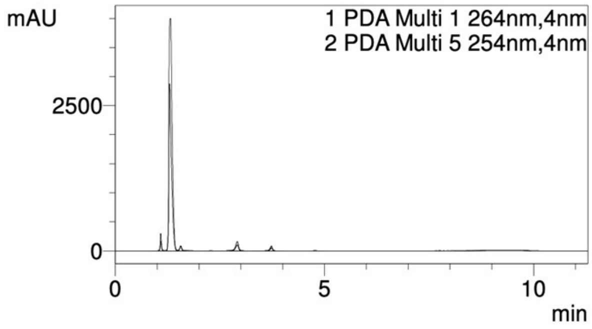

PDA Ch1 264nm

Peak#	Ret. Time	Area	Height	ID#
1	1.088	612858	280490	
2	1.318	21658448	3998808	
3	1.566	311402	83491	
4	2.289	8779	1028	
5	2.916	834052	158728	
6	3.446	1876	416	
7	3.735	329018	78599	
8	4.781	10821	2591	
9	7.671	1810	660	
10	7.749	4459	2358	
11	8.133	1493	497	
12	8.245	1569	383	
13	8.409	3039	1211	
14	8.643	3148	385	
15	8.760	1065	162	
16	9.840	4670	1546	
Total		23788507	4611352	

Figure S16 – HPLC trace of the reaction forming compound 18.

8-bromo-deaza-ribosyl-purine (16)

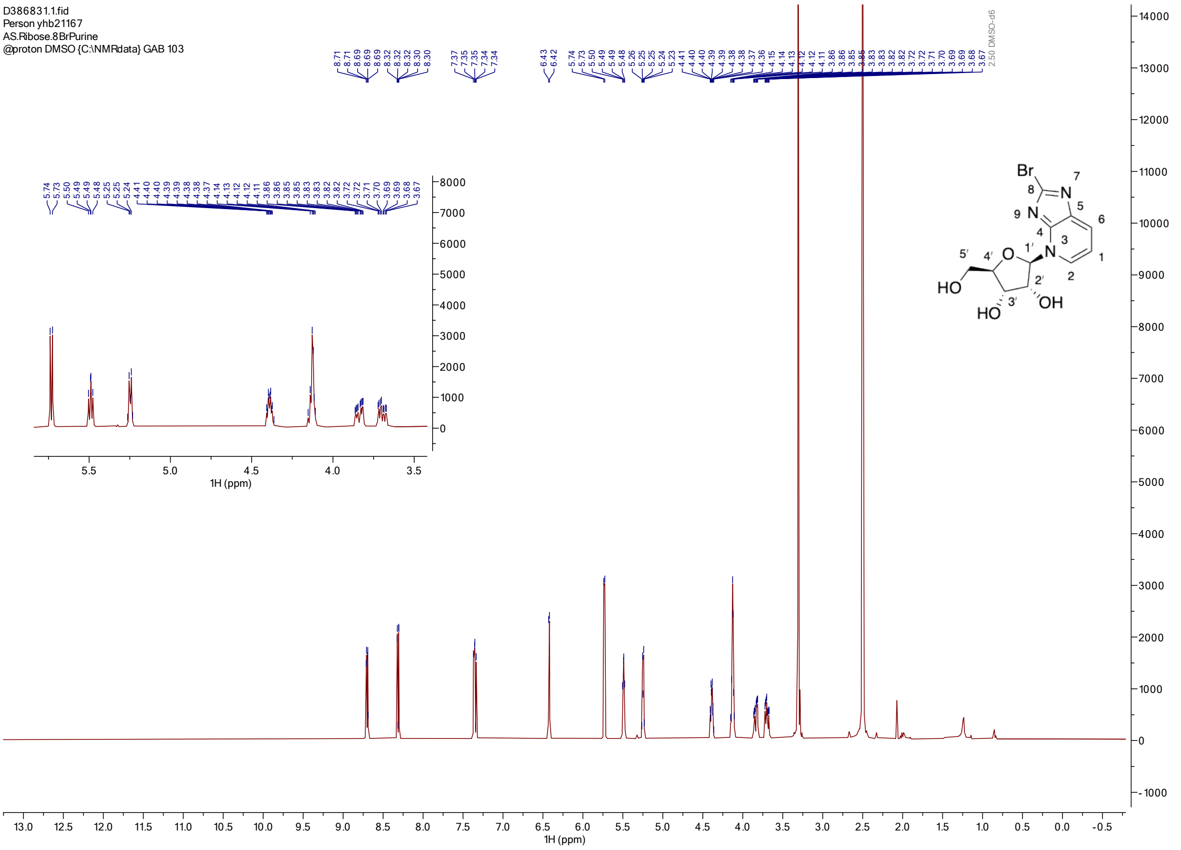

Figure S17 – ^{1}H NMR of compound 16

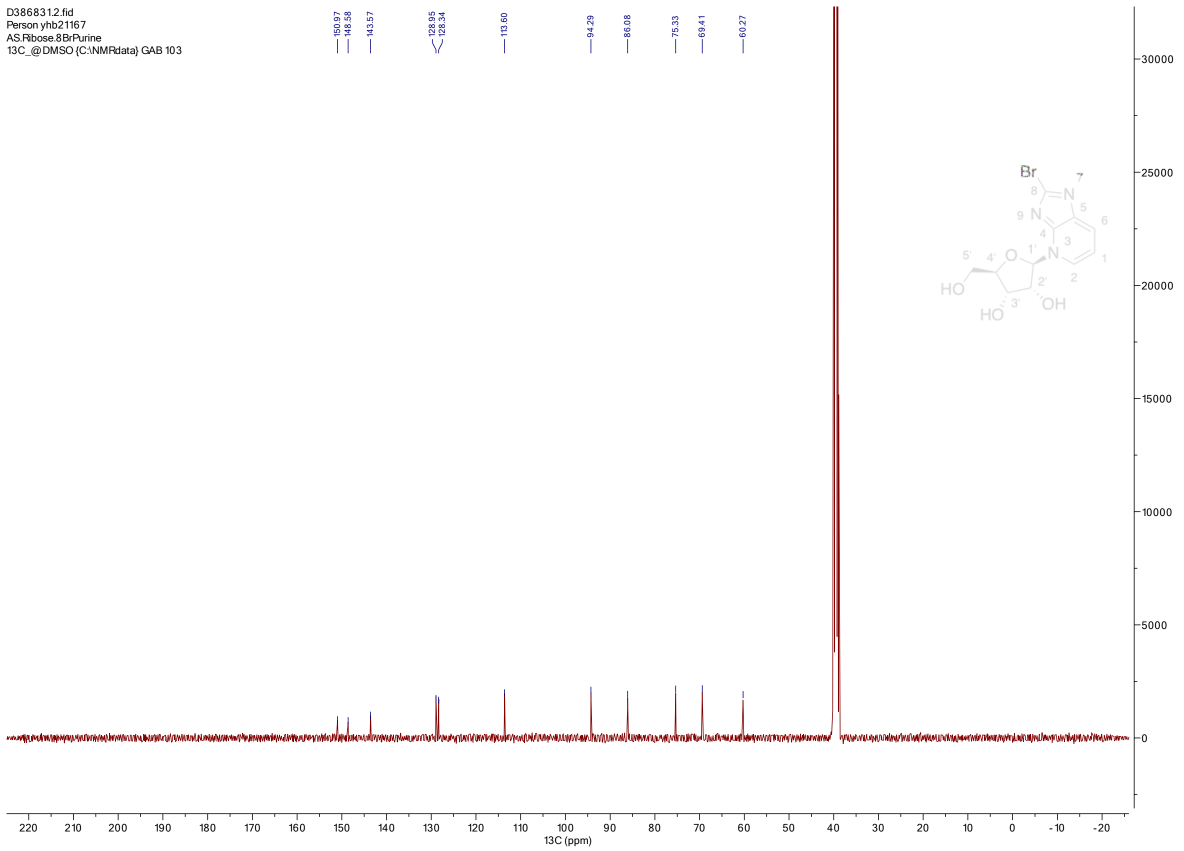

Figure S18 – ¹³C NMR of compound 16.

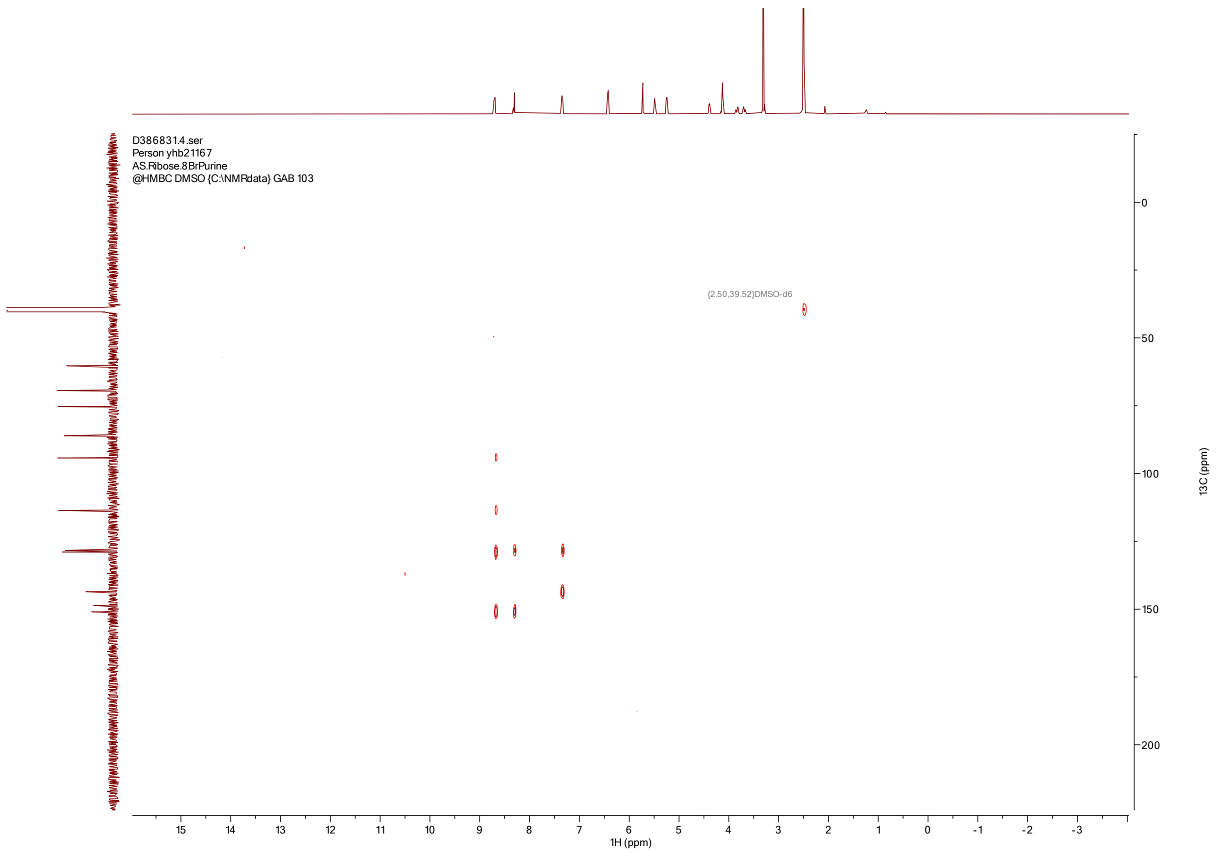

Figure S19 – HMBC of compound 16.

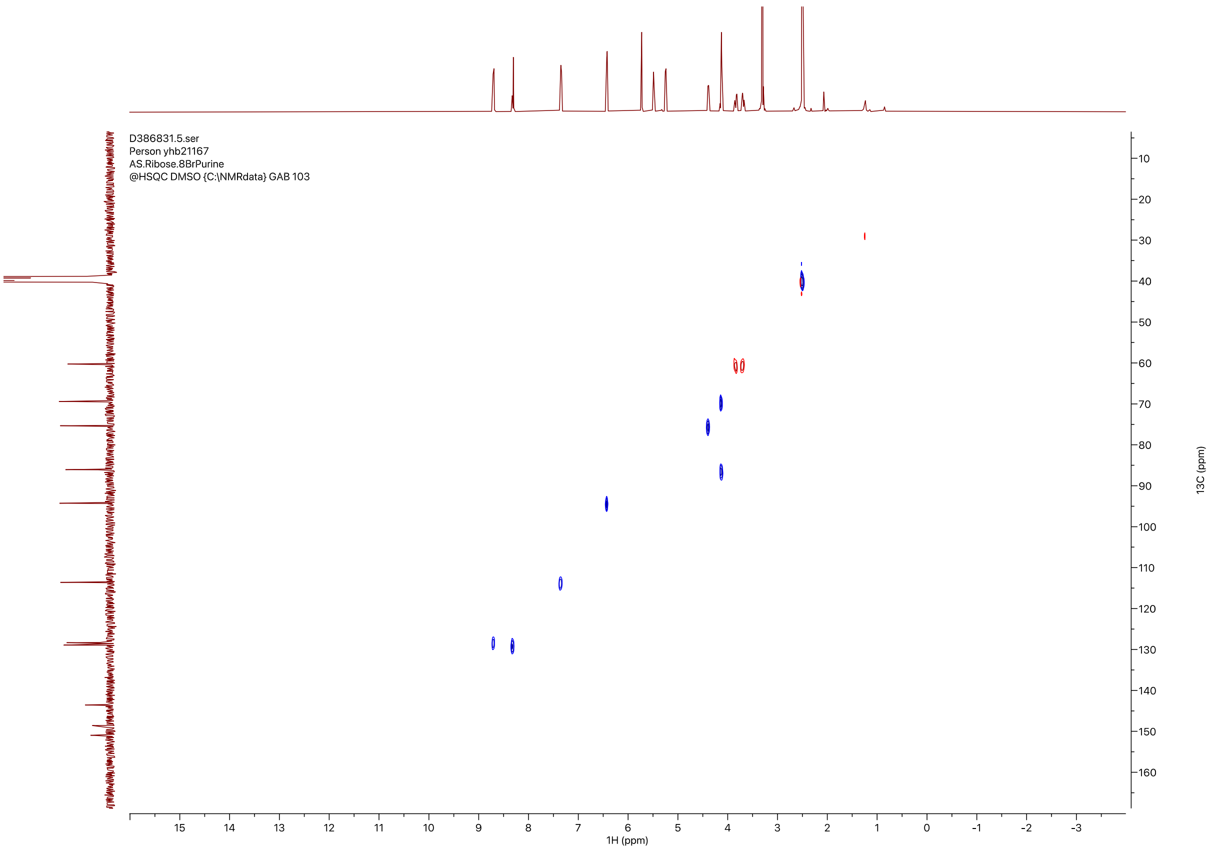

Figure S20 – HSQC of compound 16.

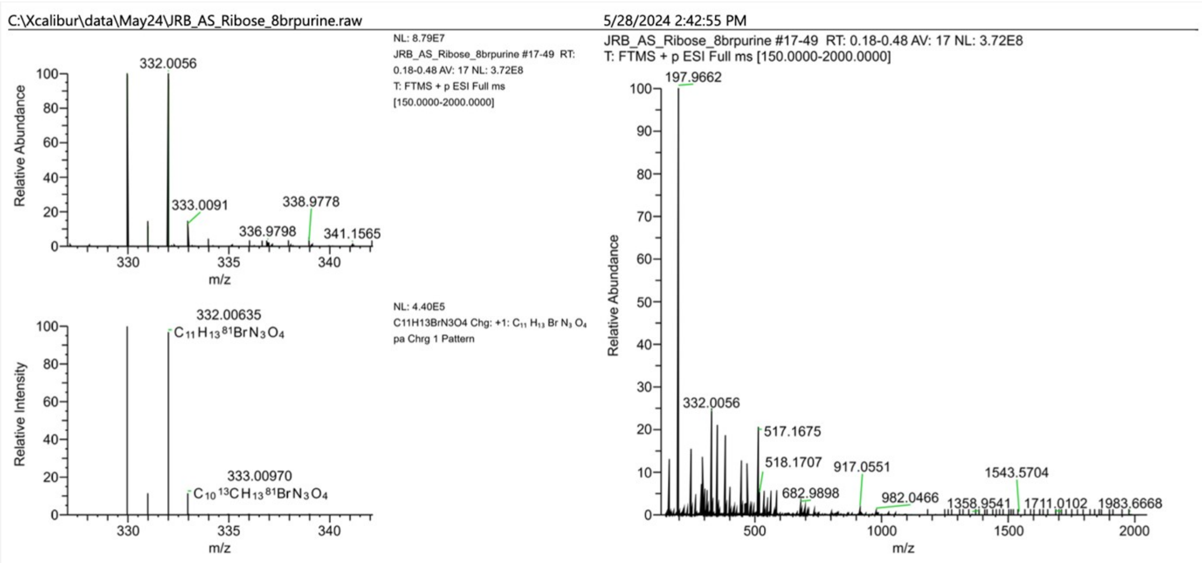

Peak Mass	Display For...	S Fit	RDB	Delta [ppm]	Theo. mass	Rank	Combined S...	# Matched I...	# Missed Iso.	MS Cov. [%]	Pattern Cov....	MSMS Matc...
330.0079	$C_{11}H_{13}O_4N_3{}^{79}Br$	63.8938297516157	6.50	-1.60	330.00839	1	96.62	6	0	98.44	100	(Collection)
330.0079	$C_9H_{11}O_3N_6{}^{79}Br$	51.1024095898677	7.00	2.47	330.00705	2	95.91	5	0	98.4	100	(Collection)
330.0079	$C_9H_6O_{10}N_4$	27.1440204514731	9.00	0.08	330.00784	3	47.72	2	2	48.86	97.72	(Collection)
330.0079	$C_{10}H_2O_6N_8$	20.3476536966238	14.00	-3.98	330.00918	4	47.36	2	2	48.86	98.4	(Collection)
330.0079	$C_{11}H_8O_{11}N$	19.7130829973747	8.50	-3.99	330.00919	5	47.33	2	2	48.86	97.41	(Collection)
330.0079	$C_{24}N_3$	11.6939963939733	26.50	-2.44	330.00867	6	42.06	2	2	43.75	77.23	(Collection)
330.0079	$C_8H_{10}O_{14}$	8.60872287748269	4.00	4.13	330.00651	7	41.9	2	2	43.75	88.84	(Collection)
330.0079	$C_7H_4O_9N_7$	8.57321338241225	9.50	4.14	330.00650	8	41.9	2	2	43.75	90.75	(Collection)

Figure S21 – HRMS of compound 16.

Sample Name	: 8brp		
Sample ID	:		
Data Filename	: runs_23032024_023.lcd		
Method Filename	: Admir Trans glyco Run.lcm		
Batch Filename	: runs.lcb		
Vial #	: 1-38	Sample Type	: Unknown
Injection Volume	: 10 uL		
Date Acquired	: 23/03/2024 18:10:57	Acquired by	: Shimadzu
Date Processed	: 23/03/2024 18:22:18	Processed by	: Shimadzu

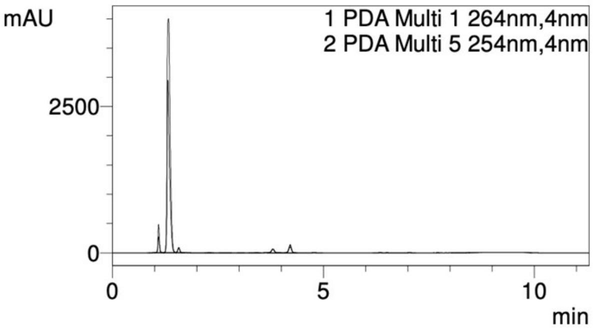

PDA Ch1 264nm

Peak#	Ret. Time	Area	Height	ID#
1	0.884	1843	789	
2	1.094	1161869	473040	
3	1.329	20355138	3992654	
4	1.574	369584	89252	
5	2.297	3750	606	
6	2.735	3155	377	
7	3.044	1365	236	
8	3.437	4317	555	
9	3.805	352406	67800	
10	4.216	618169	135266	
11	4.787	12696	2314	
12	6.209	1081	366	
13	6.352	15636	5582	
14	6.512	5028	1738	
15	6.712	1343	315	
16	6.772	1287	380	
17	7.039	12350	3912	
18	7.708	3929	1948	
19	8.138	1331	343	
20	8.252	1502	306	
21	8.414	2789	1272	
22	8.645	3010	346	
23	9.845	3502	1147	
Total		22937081	4780543	

Figure S22 – HPLC trace of the reaction forming compound 16.

6-chloro-deaza-ribosyl-purine (11)

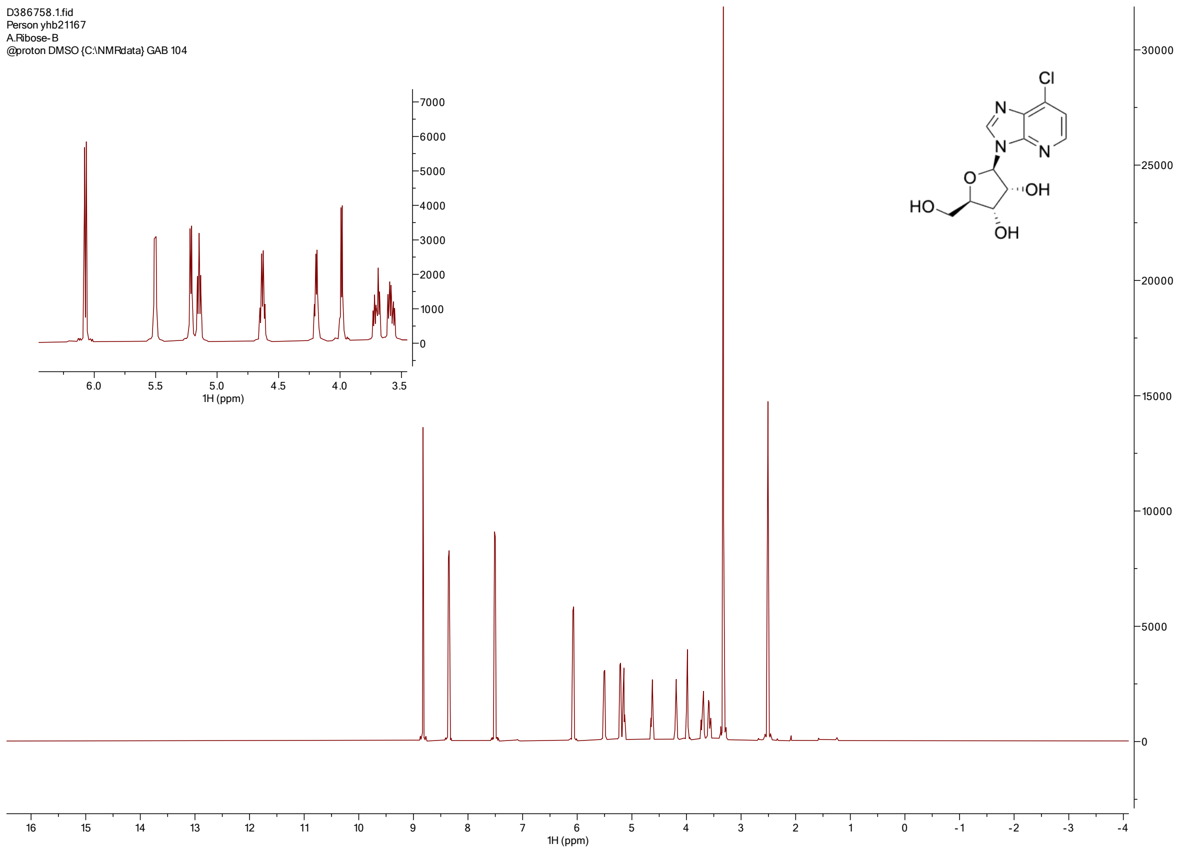

Figure S23 – ^{1}H NMR of compound 11.

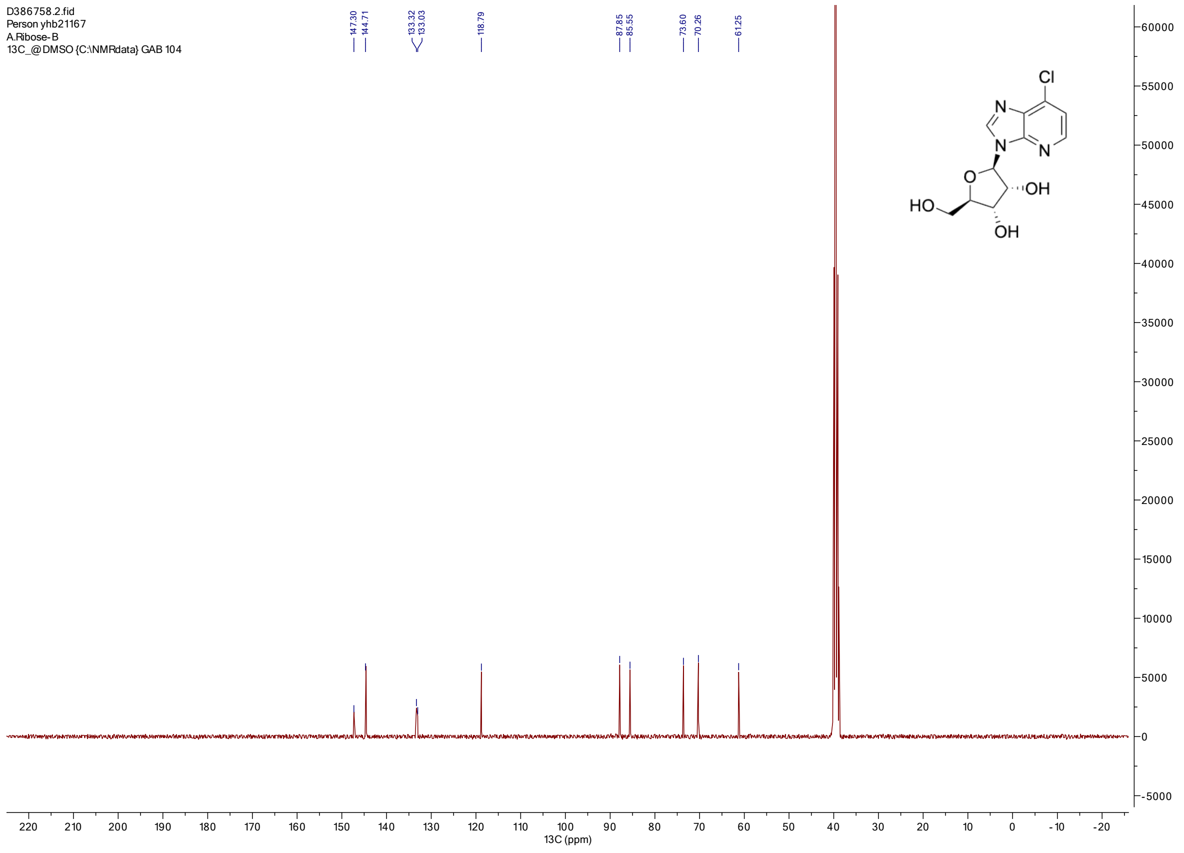

Figure S24 – ^{13}C NMR of compound 11.

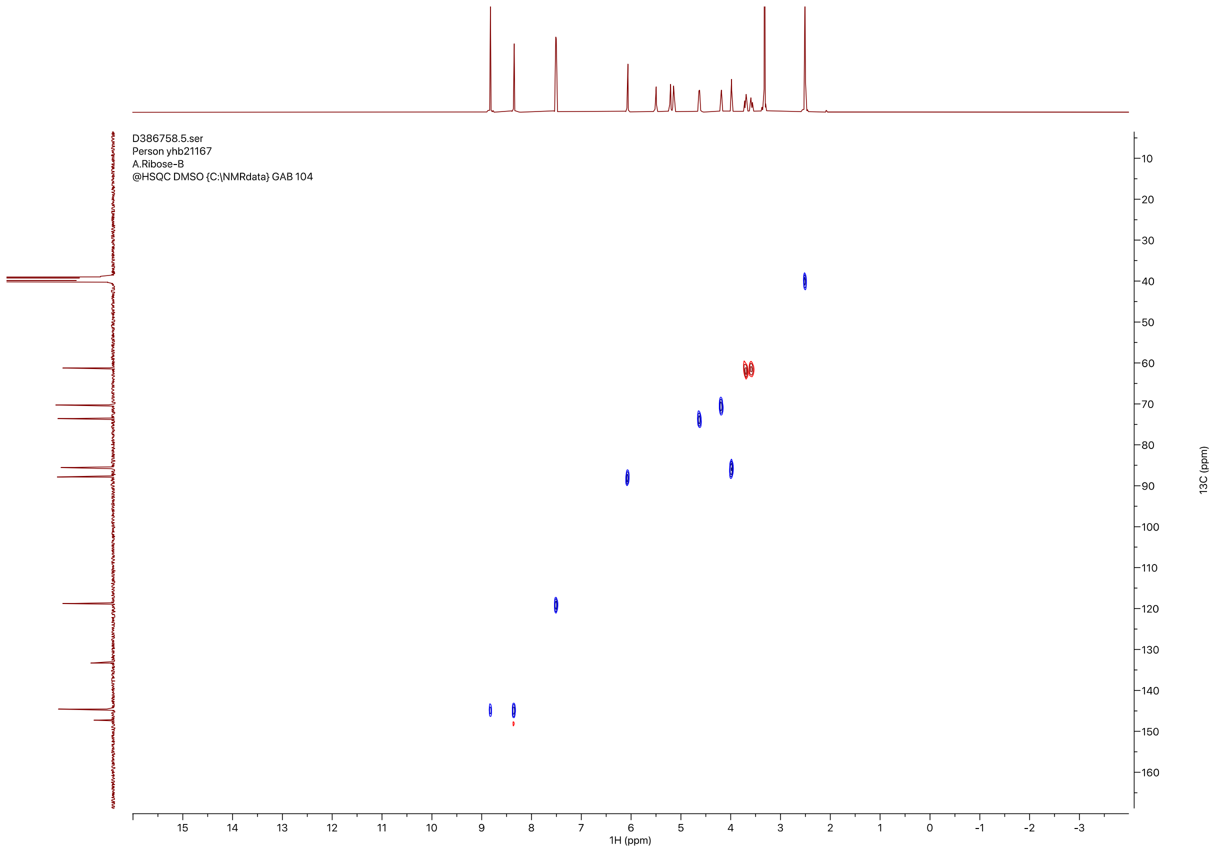

Figure S25 – HSQC of compound 11.

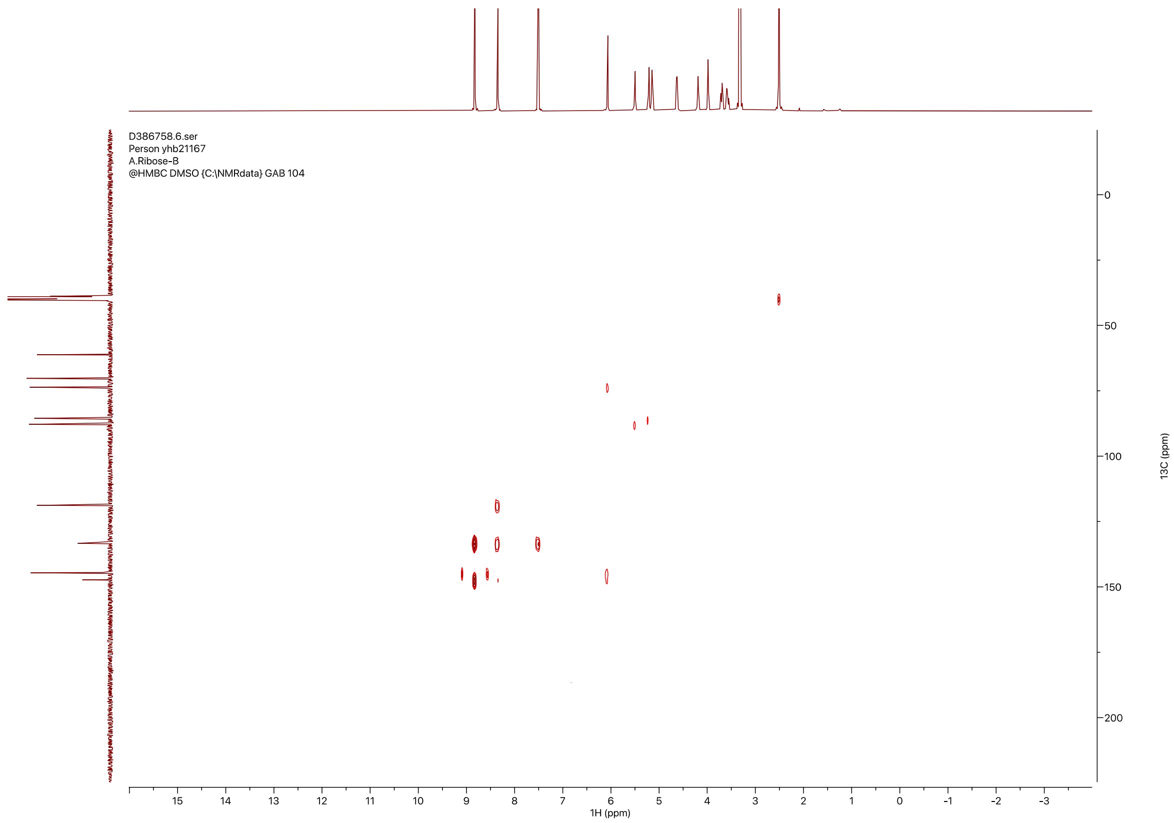

Figure S26 – HMBC of compound 11.

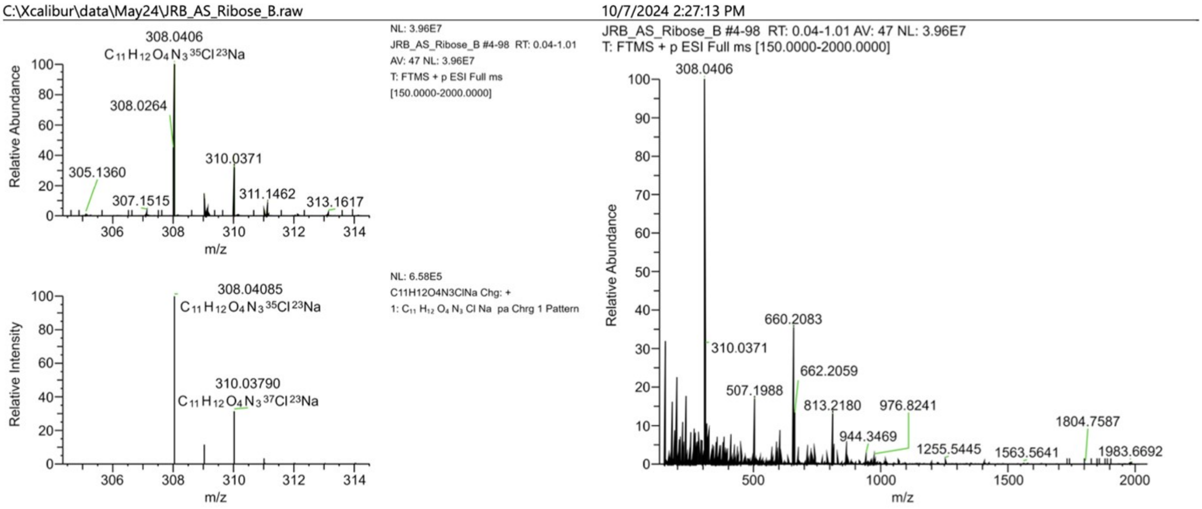

Peak Mass	Display For...	S Fit	RDB	Delta [ppm]	Theo. mass	Rank	Combined S...	# Matched I...	# Missed Iso.	MS Cov. [%]	Pattern Cov....	MSMS Matc...
308.0406	$C_{11}H_{12}O_4N_3{}^{35}Cl^{23}Na$	49.5763107755324	6.50	-0.78	308.04085	1	85.37	5	1	87.36	99.15	(Collection)
308.0406	$C_9H_{10}O_3N_6{}^{35}Cl^{23}Na$	48.5886297950758	7.00	3.58	308.03951	2	85.32	5	0	87.36	100	(Collection)
308.0406	$C_{10}H_{13}O_7N_2{}^{35}Cl$	52.0080020512075	5.00	0.11	308.04058	3	85.3	5	0	87.15	99.62	(Collection)
308.0406	$C_9H_7O_2N_9{}^{35}Cl$	29.4017969733165	10.50	0.13	308.04057	4	84.11	5	2	87.15	96.81	(Collection)
308.0406	$C_{12}H_4O_3N_8$	37.6765288579056	15.00	1.71	308.04009	5	64.53	2	1	66.03	98.95	(Collection)
308.0406	$C_{13}H_3N_9{}^{23}Na$	25.837587090504	16.50	0.82	308.04036	6	63.91	2	2	66.03	98.79	(Collection)
308.0406	$C_{13}H_{10}O_8N$	24.2361547145013	9.50	1.69	308.04009	7	63.83	2	2	66.03	97.89	(Collection)
308.0406	$C_{11}H_9O_3N_6{}^{35}Cl$	10.1869386403185	10.00	-4.23	308.04192	8	63.09	5	1	66.03	75	(Collection)

Figure S27 – HRMS of compound 11.

Sample Name : b
Sample ID :
Data Filename : base b_19032024_003.lcd
Method Filename : Admir Trans glyco Run.lcm
Batch Filename : base b.lcb
Vial # : 1-93
Injection Volume : 10 uL
Date Acquired : 19/03/2024 22:38:19
Date Processed : 19/03/2024 22:49:41

Sample Type : Unknown
Acquired by : Shimadzu
Processed by : Shimadzu

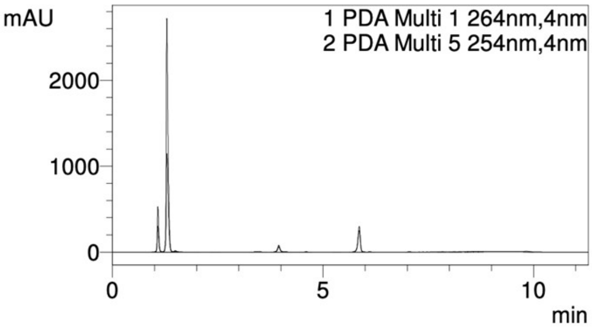

PDA Ch1 264nm

Peak#	Ret. Time	Area	Height	ID#
1	1.081	1296572	509525	
2	1.295	8058066	2672410	
3	1.494	36347	14405	
4	2.481	2129	354	
5	3.498	15777	2834	
6	3.951	350694	78320	
7	4.603	4343	1081	
8	5.330	1154	248	
9	5.862	1005059	248505	
10	6.113	4488	1011	
11	7.048	7877	3149	
12	7.834	4325	1235	
13	8.113	2633	696	
14	8.238	1230	393	
15	8.411	1197	436	
16	8.480	1860	267	
17	8.645	1791	344	
18	8.768	1126	227	
19	9.854	5851	1727	
Total		10802517	3537168	

Figure S28 – HPLC trace of the reaction forming compound 11.

6-dimethylamino-adenosine (14)

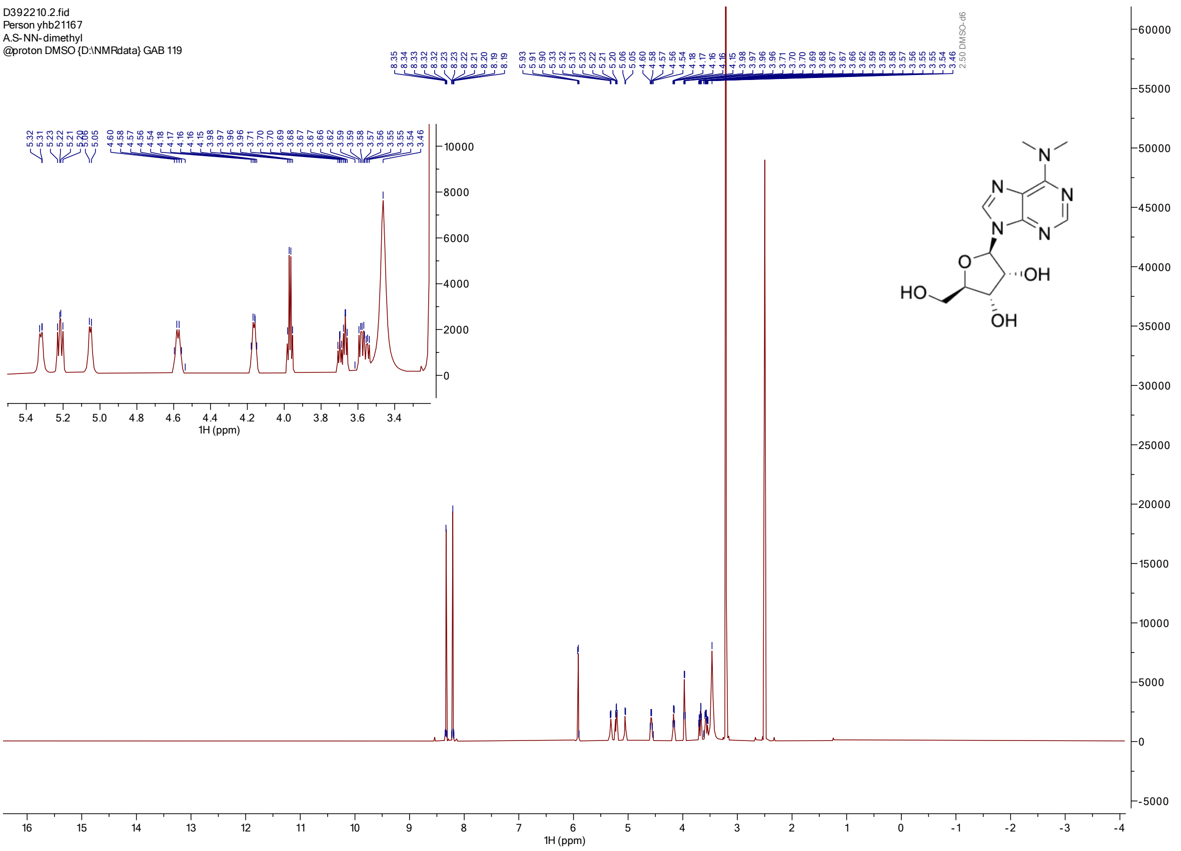

Figure S29 – 1H NMR of compound 14.

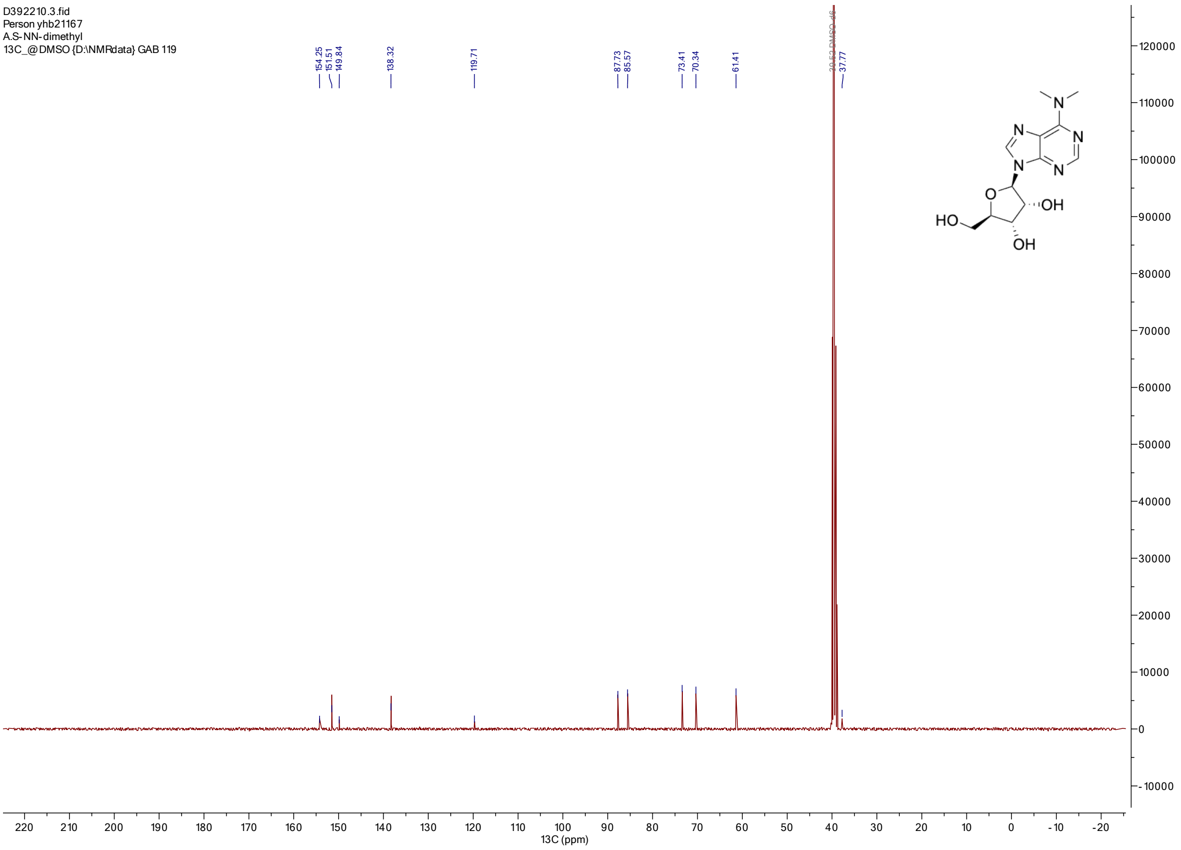

Figure S30 – 13C NMR of compound 14.

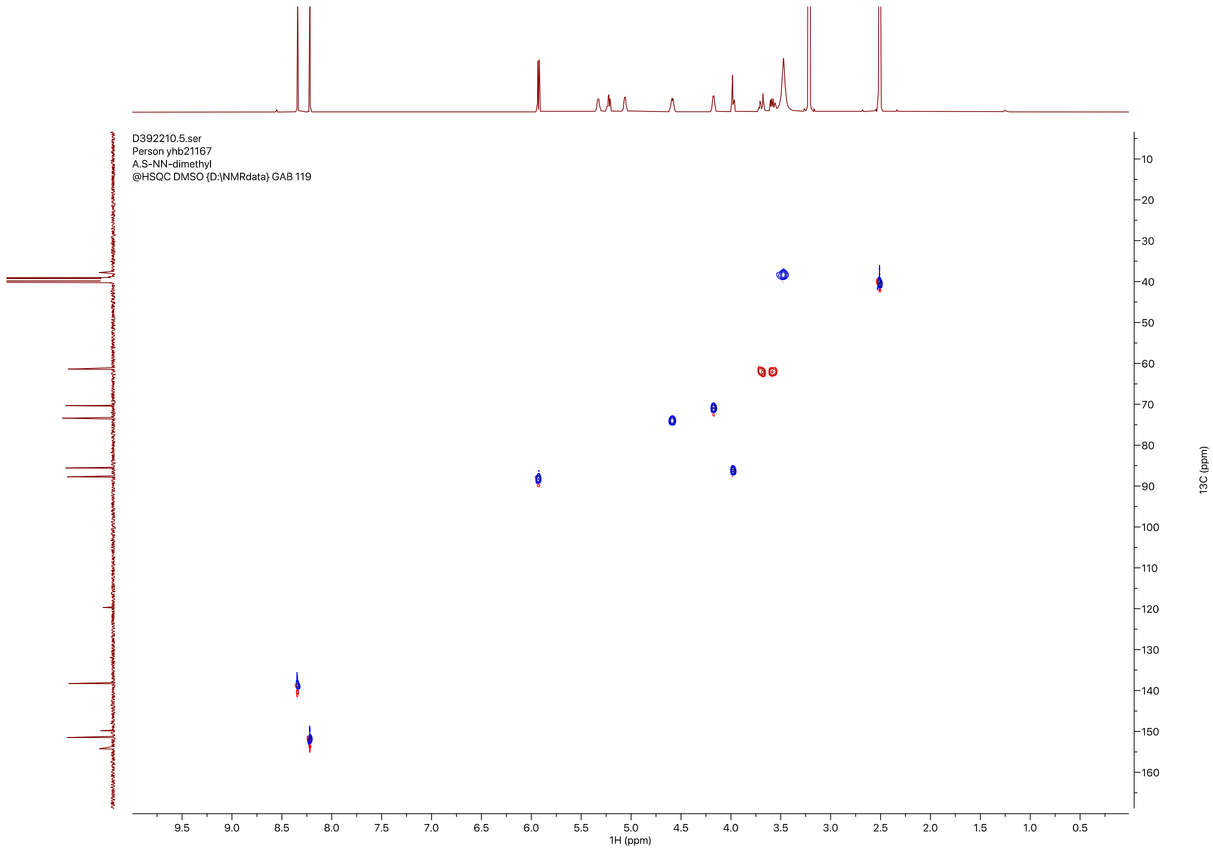

Figure S31 – HSQC of compound 14.

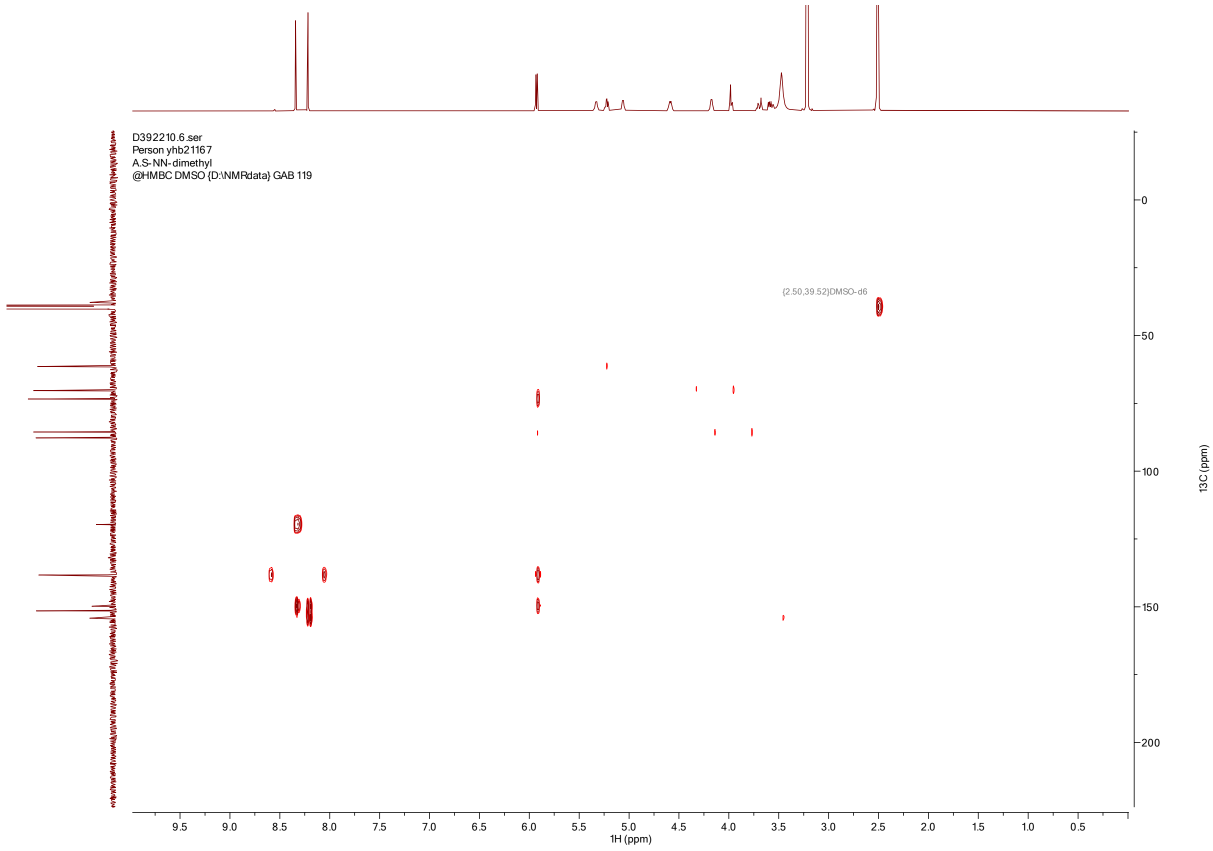

Figure S32 – HMBC of compound 14.

Sample Name : 6-nndimethyl-purine
Sample ID :
Data Filename : runs_21032024_015.lcd
Method Filename : Admir Trans glyco Run.lcm
Batch Filename : runs.lcb
Vial # : 1-75
Injection Volume : 10 uL
Date Acquired : 21/03/2024 21:05:00
Date Processed : 21/03/2024 21:16:20

Sample Type : Unknown
Acquired by : Shimadzu
Processed by : Shimadzu

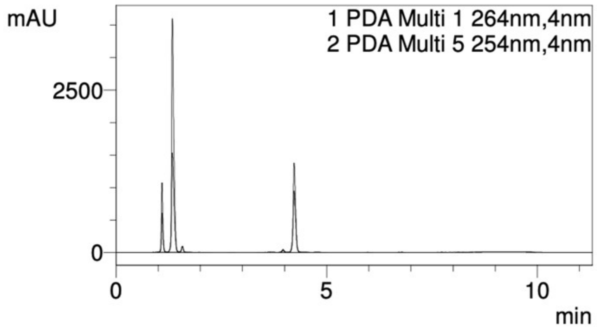

PDA Ch1 264nm

Peak#	Ret. Time	Area	Height	ID#
1	0.886	3013	1466	
2	0.958	6967	2526	
3	1.094	2629559	1061047	
4	1.340	12924562	3577178	
5	1.577	321610	96373	
6	1.748	7232	1125	
7	1.976	4305	543	
8	2.129	1247	185	
9	2.726	1563	265	
10	3.454	1817	451	
11	3.545	2141	563	
12	3.632	3753	1156	
13	3.730	20195	4932	
14	3.967	180274	45931	
15	4.231	5578970	1364969	
16	4.784	20870	4097	
17	5.978	1609	547	
18	6.718	1749	928	
19	6.786	4266	1690	
20	7.727	4003	2065	
21	8.128	3074	829	
22	8.248	1174	407	
23	8.414	1867	409	
24	8.647	1576	277	
25	9.838	5866	1629	
Total		21733263	6171589	

Figure S33 – HPLC trace of the reaction forming compound 14.

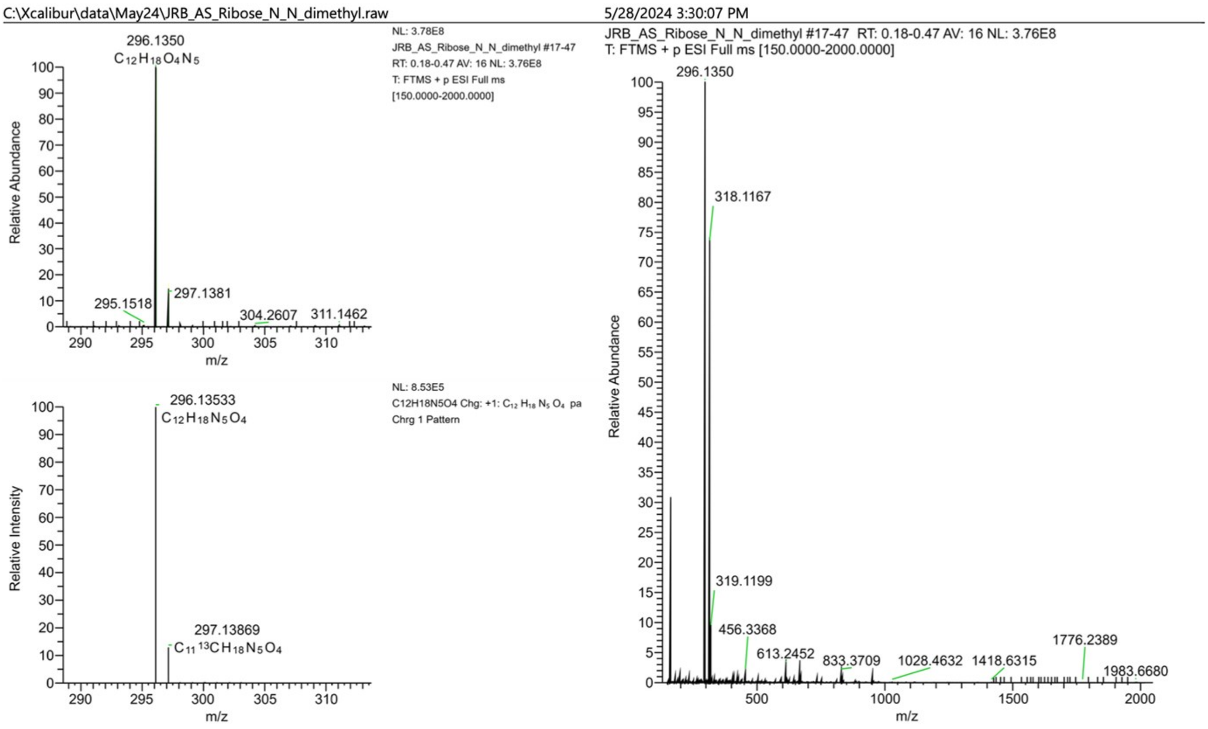

Peak Mass	Display For...	S Fit	RDB	Delta [ppm]	Theo. mass	Rank	Combined S...	# Matched I...	# Missed Iso.	MS Cov. [%]	Pattern Cov....	MSMS Matc...
296.1350	$C_{12}H_{18}O_4N_5$	44.11223061 45794	6.50	-1.22	296.13533	1	96.88	3	1	99.82	99.89	(Collection)
296.1350	$C_{11}H_{22}O_8N$	23.74932120 55287	1.50	3.29	296.13399	2	94.84	3	1	98.79	98.06	(Collection)
296.1350	$C_{10}H_{16}O_3N_8$	21.98139174 50887	7.00	3.31	296.13399	3	94.74	3	1	98.79	96.51	(Collection)

Figure S34 – HRMS of compound 14.

2-bromo-deaza-ribosyl-purine (15)

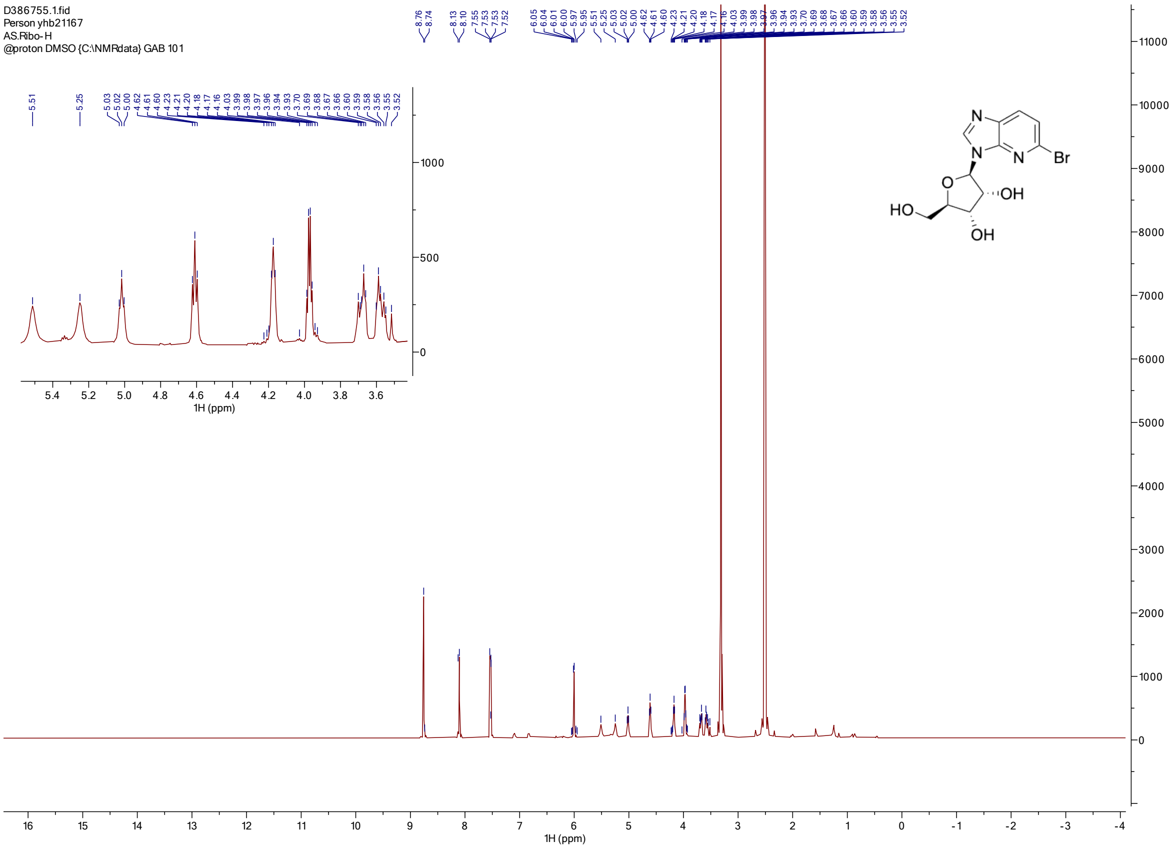

Figure S35 – ^{1}H NMR of compound 15.

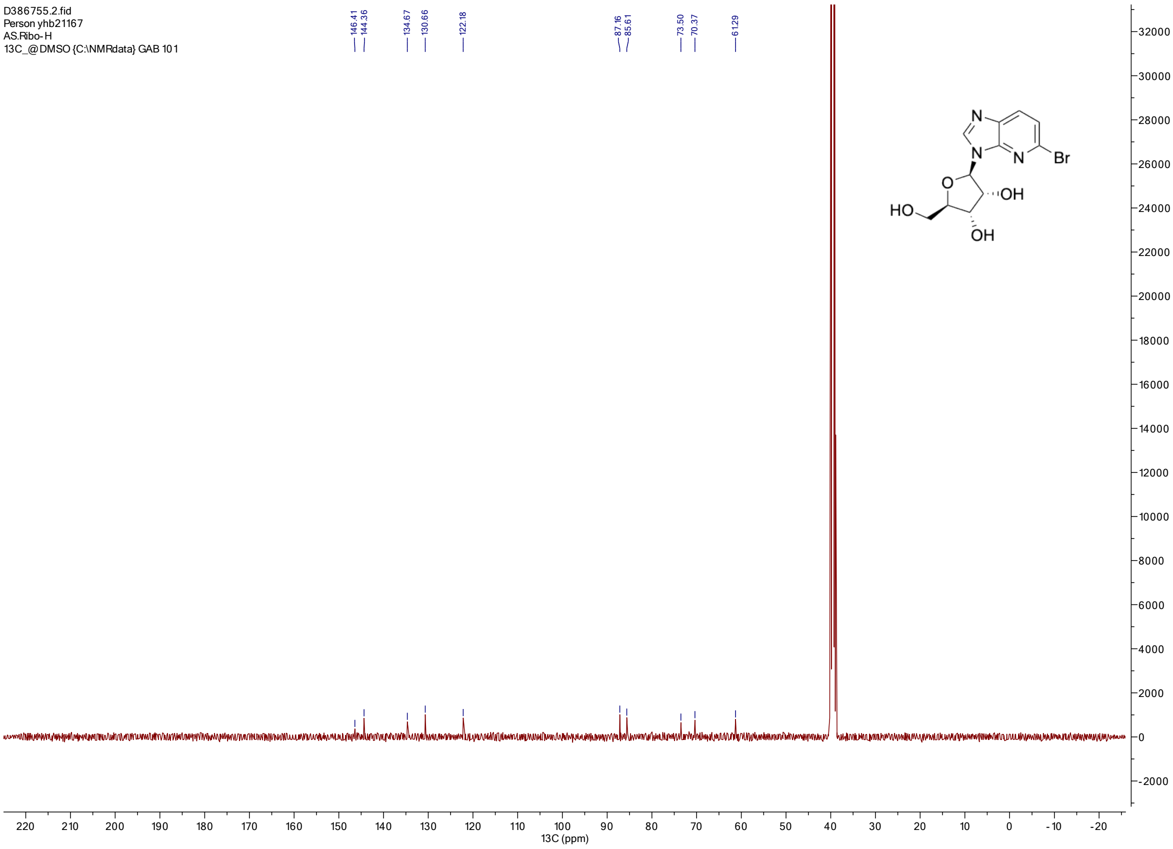

Figure S36 – 13C NMR of compound 15.

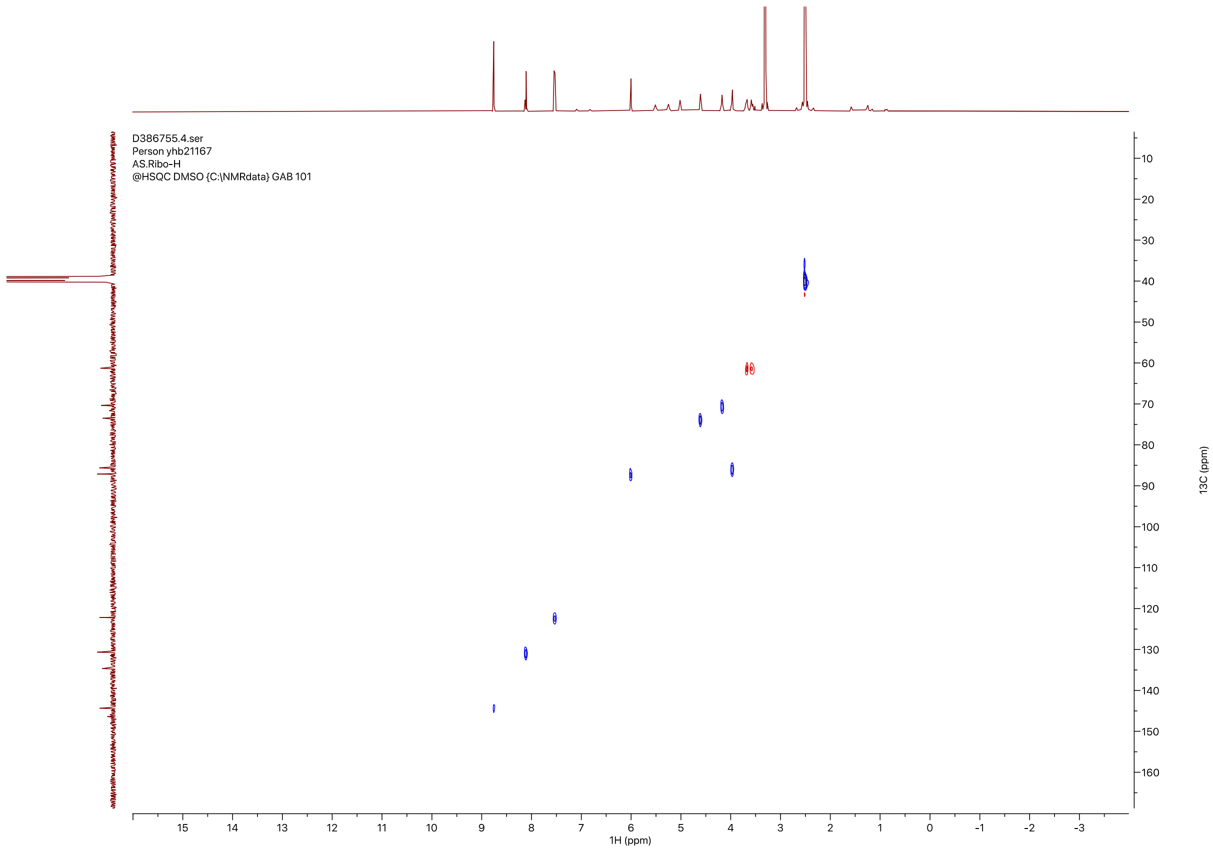

Figure S37 – HSQC of compound 15.

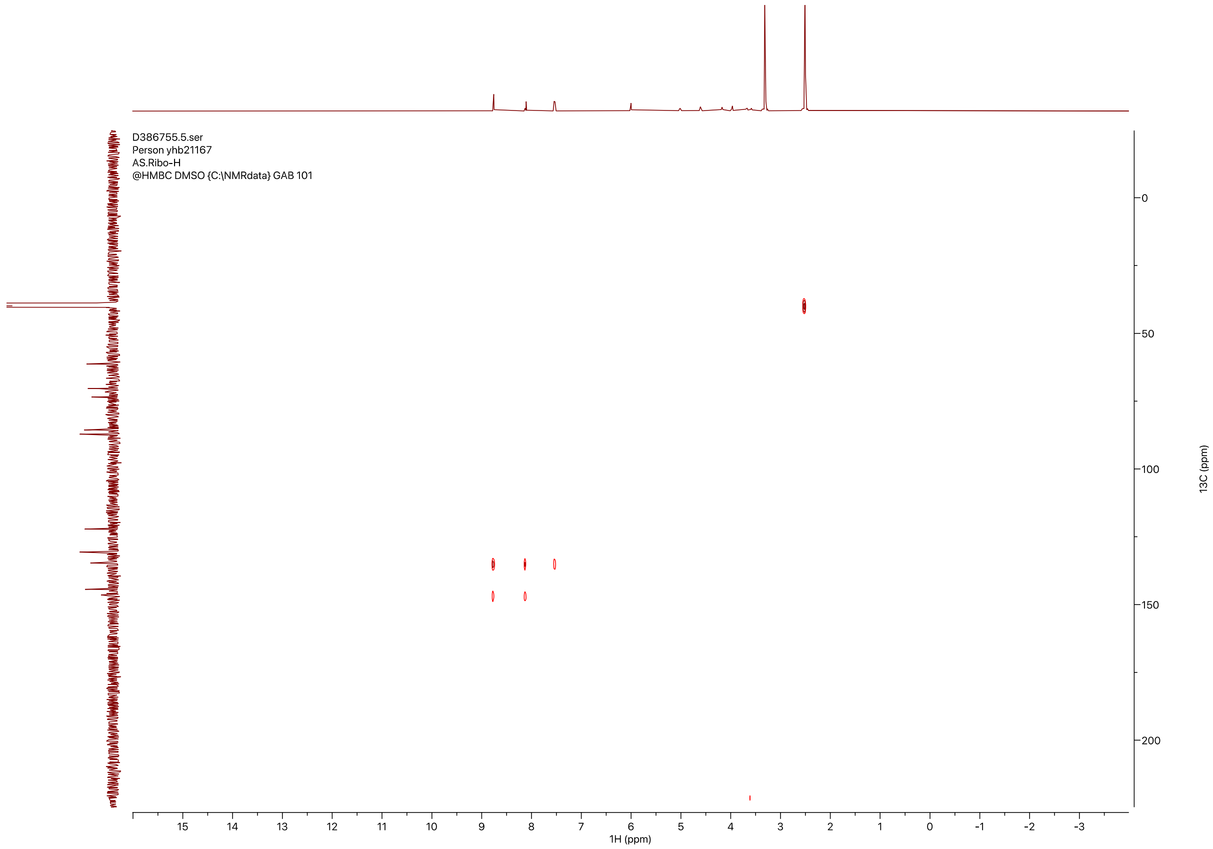

Figure S38 – HMBC of compound 15.

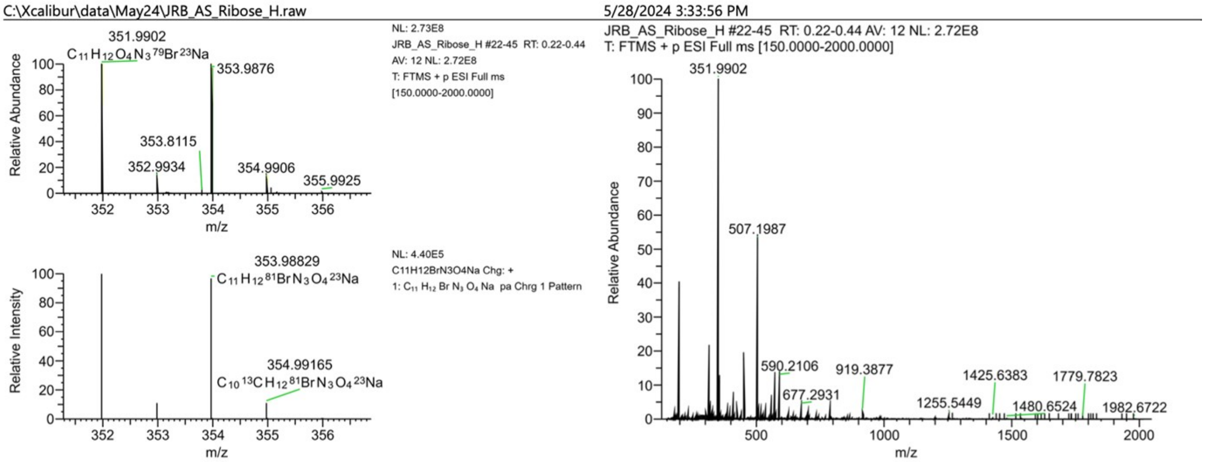

Peak Mass	Display For...	S Fit	RDB	Delta [ppm]	Theo. mass	Rank	Combined S...	# Matched I...	# Missed Iso.	MS Cov. [%]	Pattern Cov....	MSMS Matc...
351.9902	$C_{11}H_{12}O_4N_3{}^{79}Br^{23}Na$	62.2185925720359	6.50	-0.34	351.99034	1	96.36	6	0	98.26	100	(Collection)
351.9902	$C_9H_{10}O_3N_6{}^{79}Br^{23}Na$	52.7115464480711	7.00	3.47	351.98900	2	95.82	5	0	98.21	100	(Collection)
351.9902	$C_9H_7O_2N_9{}^{79}Br$	46.5595430310779	10.50	0.45	351.99006	3	95.01	5	0	97.71	99.64	(Collection)
351.9902	$C_{10}H_{13}O_7N_2{}^{79}Br$	39.88831211799	5.00	0.44	351.99006	4	94.66	6	0	97.71	99.12	(Collection)
351.9902	$C_8H_{11}O_6N_5{}^{79}Br$	23.1113044846924	5.50	4.25	351.98872	5	84.14	6	0	87.53	91.1	(Collection)
351.9902	$C_9H_5O_{10}N_4{}^{23}Na$	26.2670047618208	9.00	1.22	351.98979	6	48.2	2	2	49.42	97.69	(Collection)
351.9902	$C_{10}H_8O_{14}$	26.2191779379565	7.00	-1.81	351.99086	7	48.2	2	2	49.42	96.88	(Collection)
351.9902	$C_9H_2O_9N_7$	24.671026693524	12.50	-1.80	351.99085	8	48.12	2	2	49.42	97.86	(Collection)
351.9902	$C_{10}HO_6N_8{}^{23}Na$	24.2992791803204	14.00	-2.58	351.99113	9	48.1	2	2	49.42	98.37	(Collection)
351.9902	$C_{11}H_7O_{11}N^{23}Na$	23.6923471216253	8.50	-2.59	351.99113	10	48.06	2	2	49.42	97.38	(Collection)

Figure S39 – HRMS of compound 15.

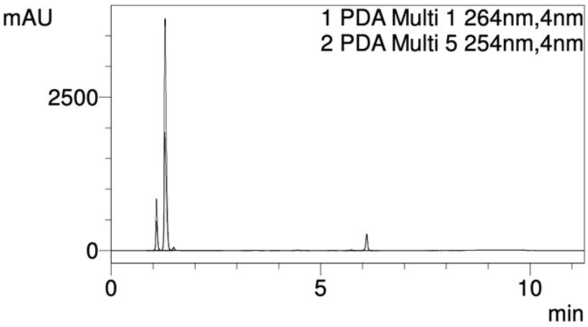

PDA Ch1 264nm

Peak#	Ret. Time	Area	Height	ID#
1	0.890	1315	601	
2	0.960	3423	1253	
3	1.085	2121338	829090	
4	1.289	12963039	3716184	
5	1.494	160852	52096	
6	1.624	7248	1210	
7	1.806	3927	682	
8	1.931	3121	436	
9	2.152	1627	190	
10	2.376	1072	165	
11	2.476	2487	459	
12	2.592	1090	211	
13	3.198	1038	265	
14	3.392	4191	1066	
15	3.496	20191	4943	
16	3.926	2877	641	
17	4.456	43716	9490	
18	4.601	7702	1934	
19	5.291	4748	1145	
20	5.735	43295	9937	
21	6.103	747862	244812	
22	7.637	1492	389	

Figure S40 – HPLC trace of the reaction forming compound 15.

N-6-methyl-adenosine (13)

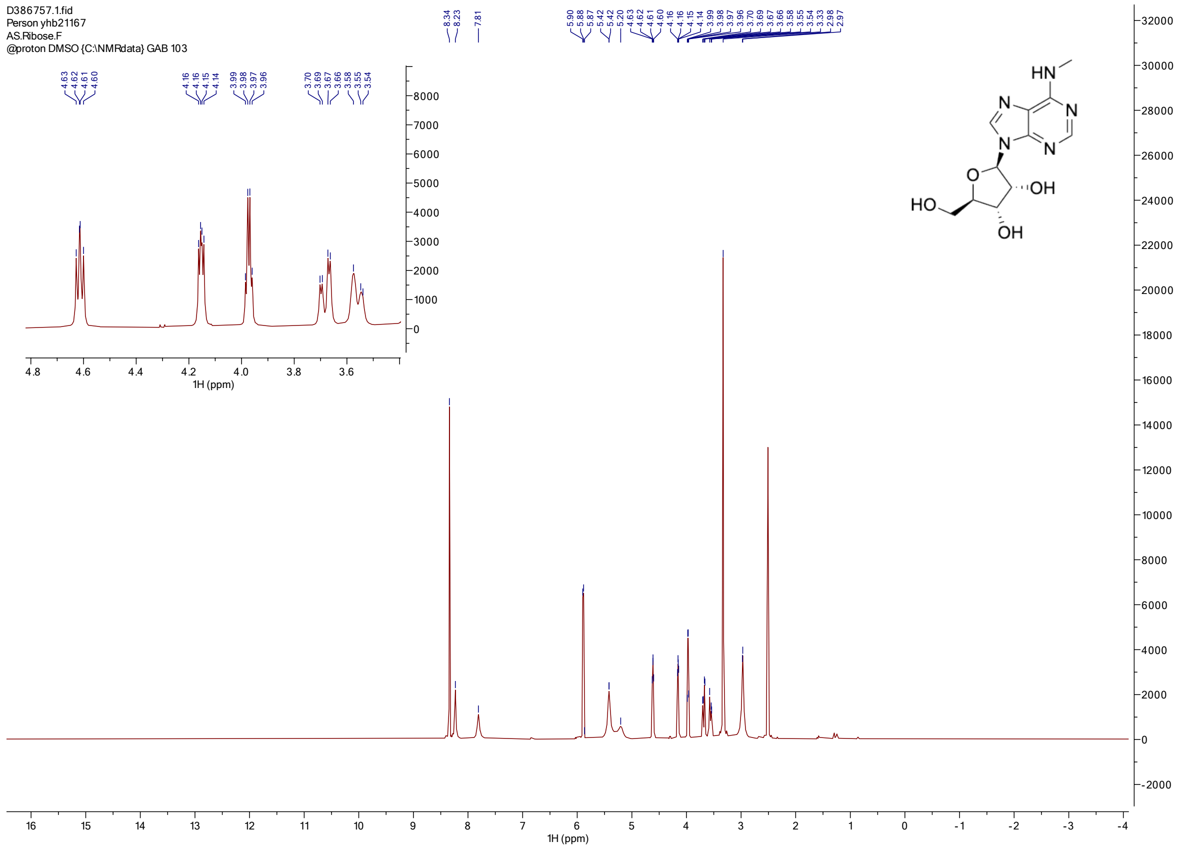

Figure S41 – 1H NMR of compound 13.

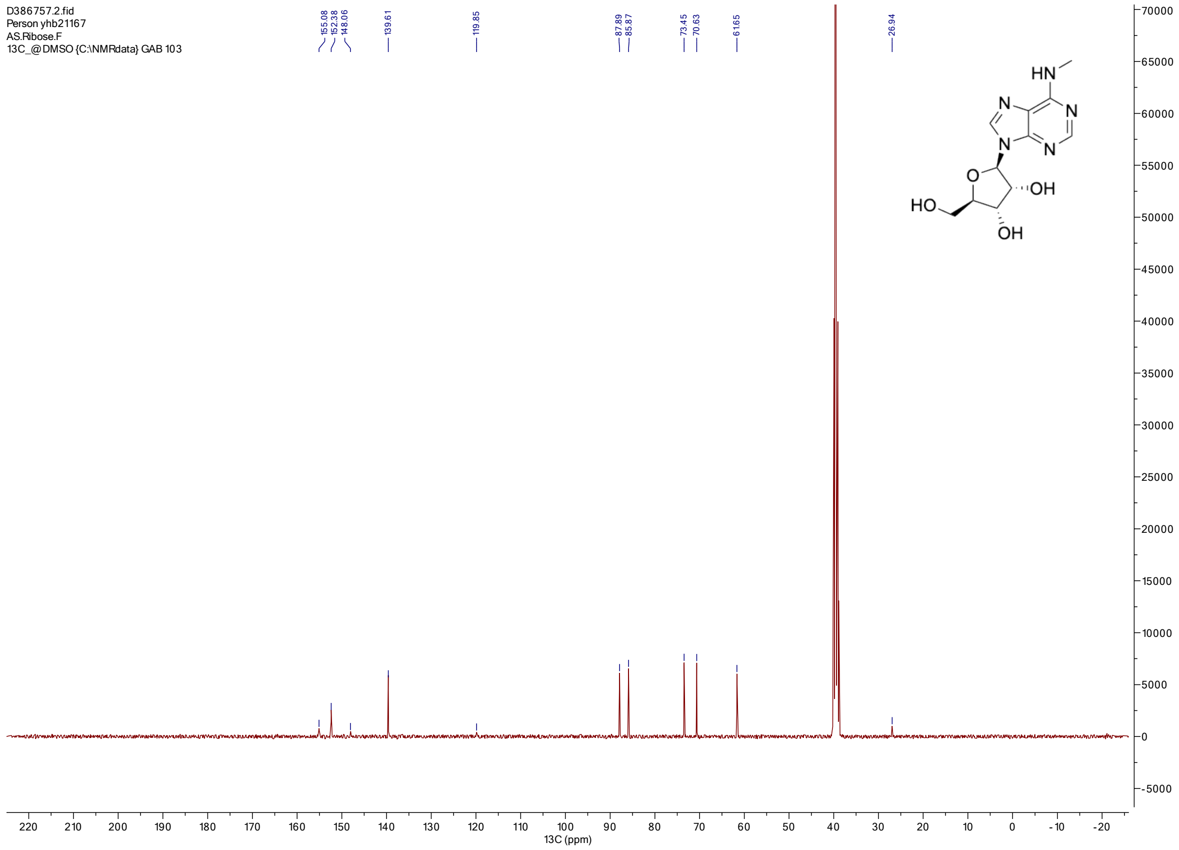

Figure S42 – 13C NMR of compound 13.

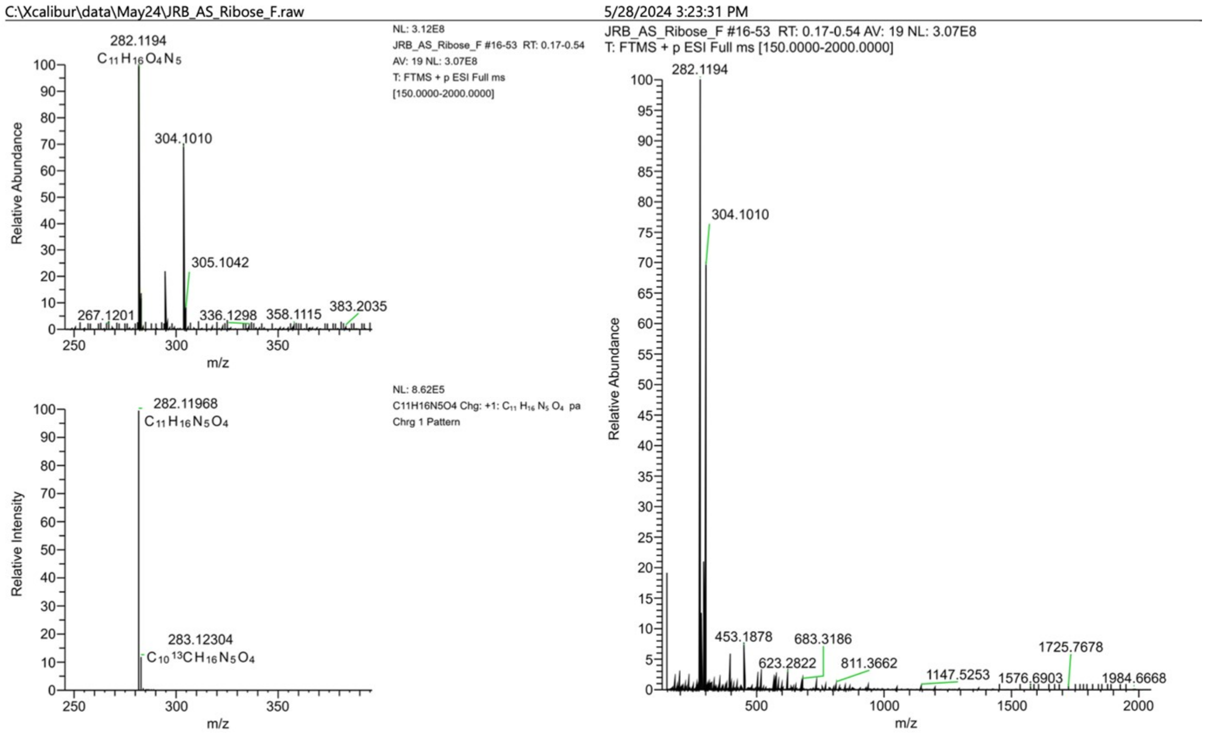

Peak Mass	Display For...	S Fit	RDB	Delta [ppm]	Theo. mass	Rank	Combined S...	# Matched I...	# Missed Iso.	MS Cov. [%]	Pattern Cov....	MSMS Matc...
282.1194	$C_{11}H_{16}O_4N_5$	31.64891756 09933	6.50	-1.06	282.11968	1	95.91	3	2	99.48	98.27	(Collection)
282.1194	$C_9H_{14}O_3N_8$	28.34384843 94902	7.00	3.70	282.11834	2	95.73	3	1	99.48	97.37	(Collection)
282.1194	$C_{10}H_{20}O_8N$	22.54721382 37646	1.50	3.68	282.11834	3	94.6	3	1	98.61	98.15	(Collection)

Figure S43 – HRMS of compound 13.

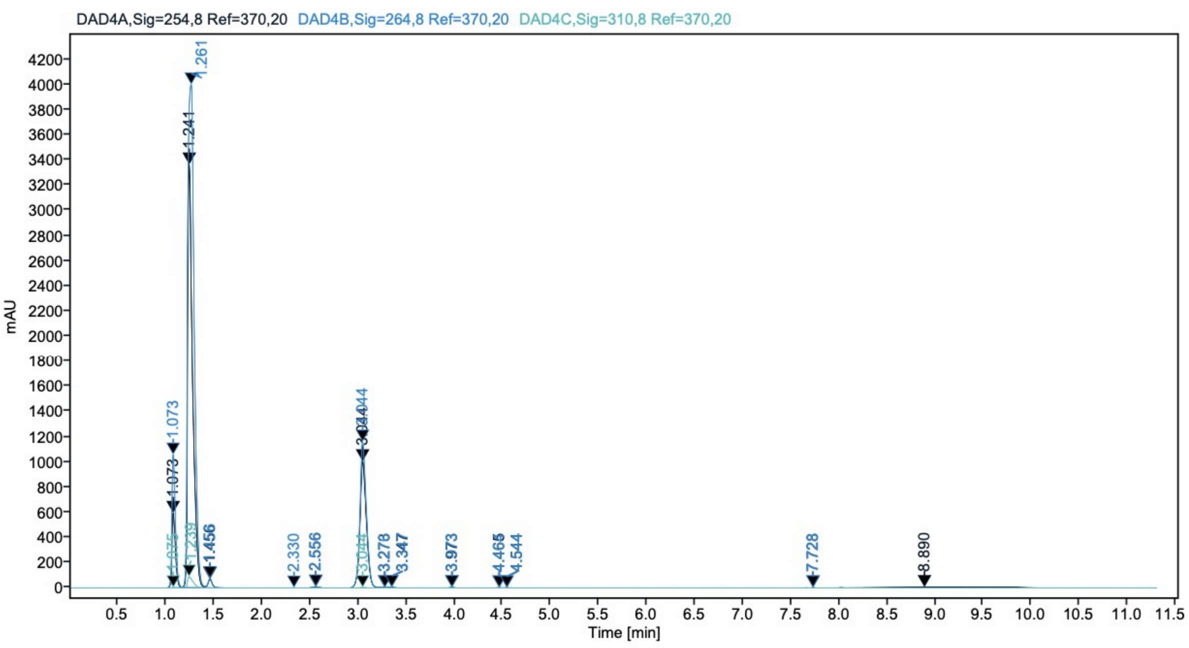

Figure S44 – HPLC trace of the reaction forming compound 13.

2-fluoro-ribosyl-adenosine (12)

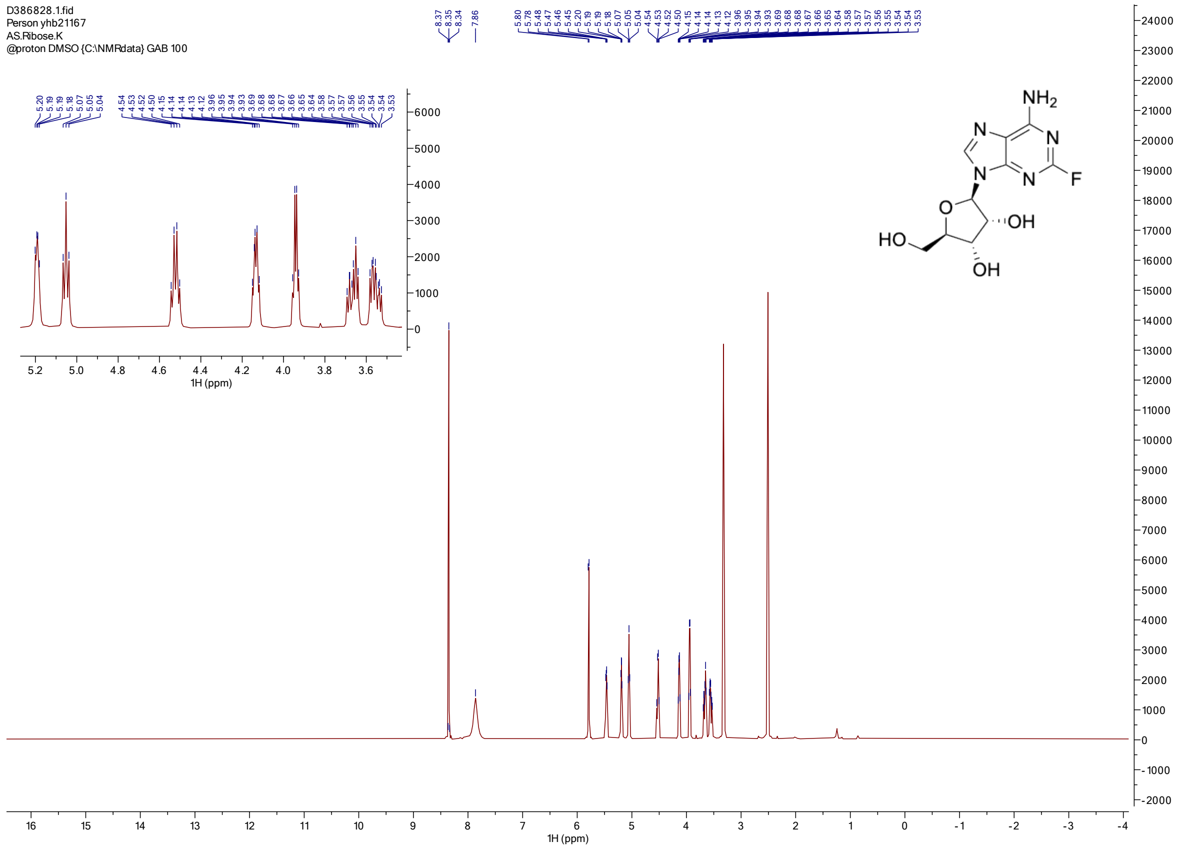

Figure S45 – 1H NMR of compound 12.

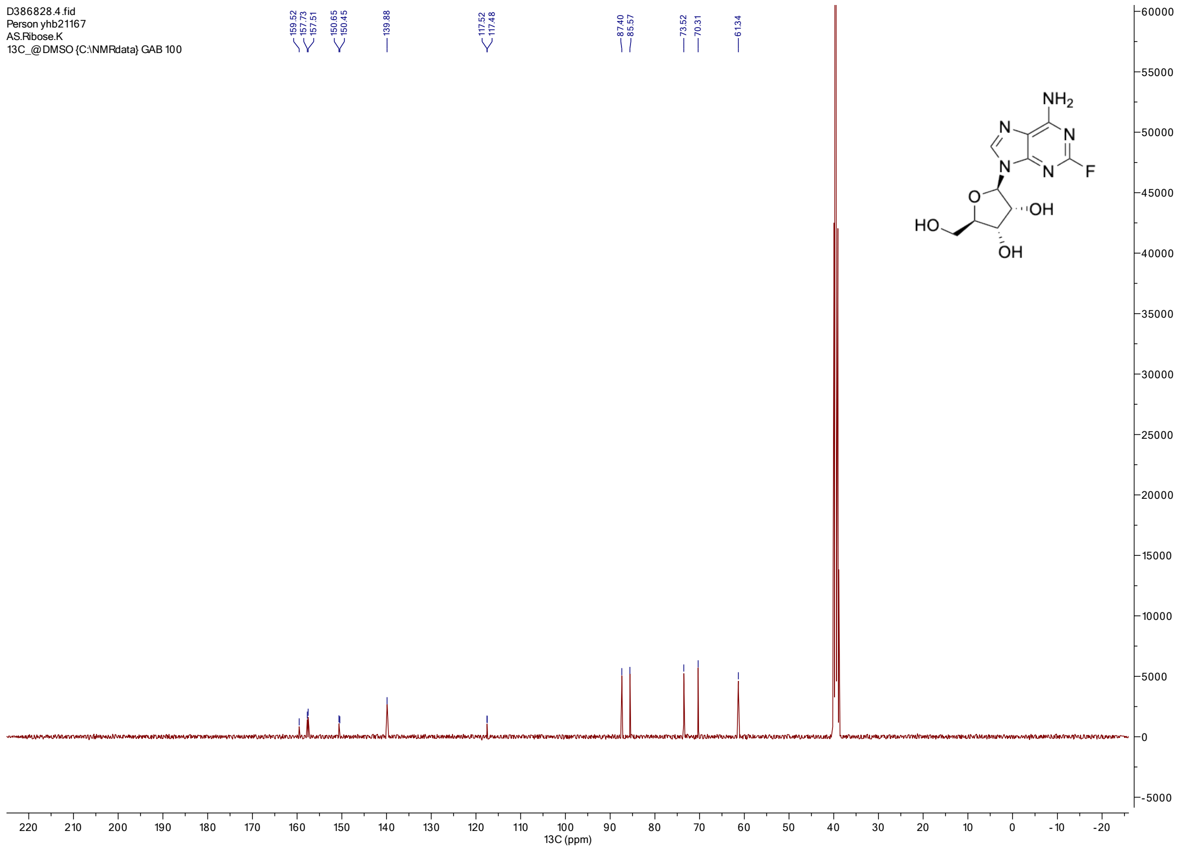

Figure S46 – 13C NMR of compound 12.

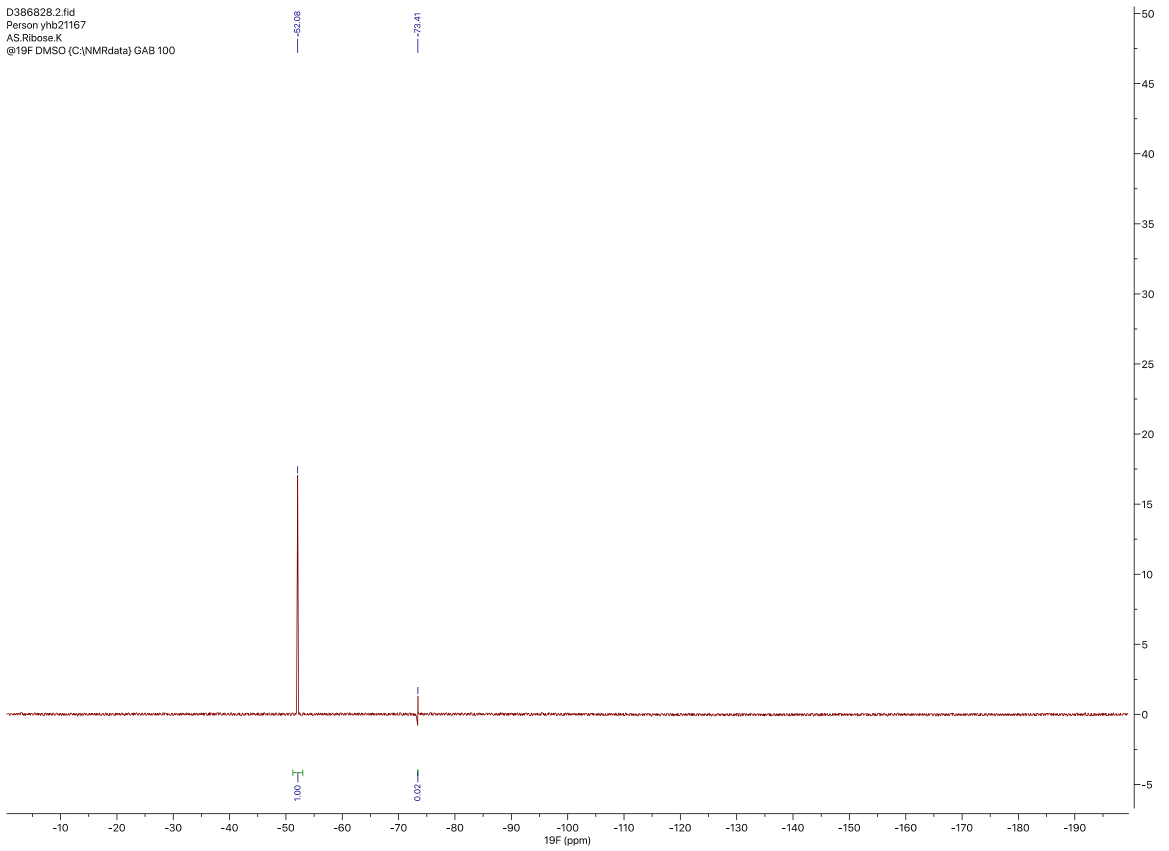

Figure S47 – 19F NMR of compound 12.

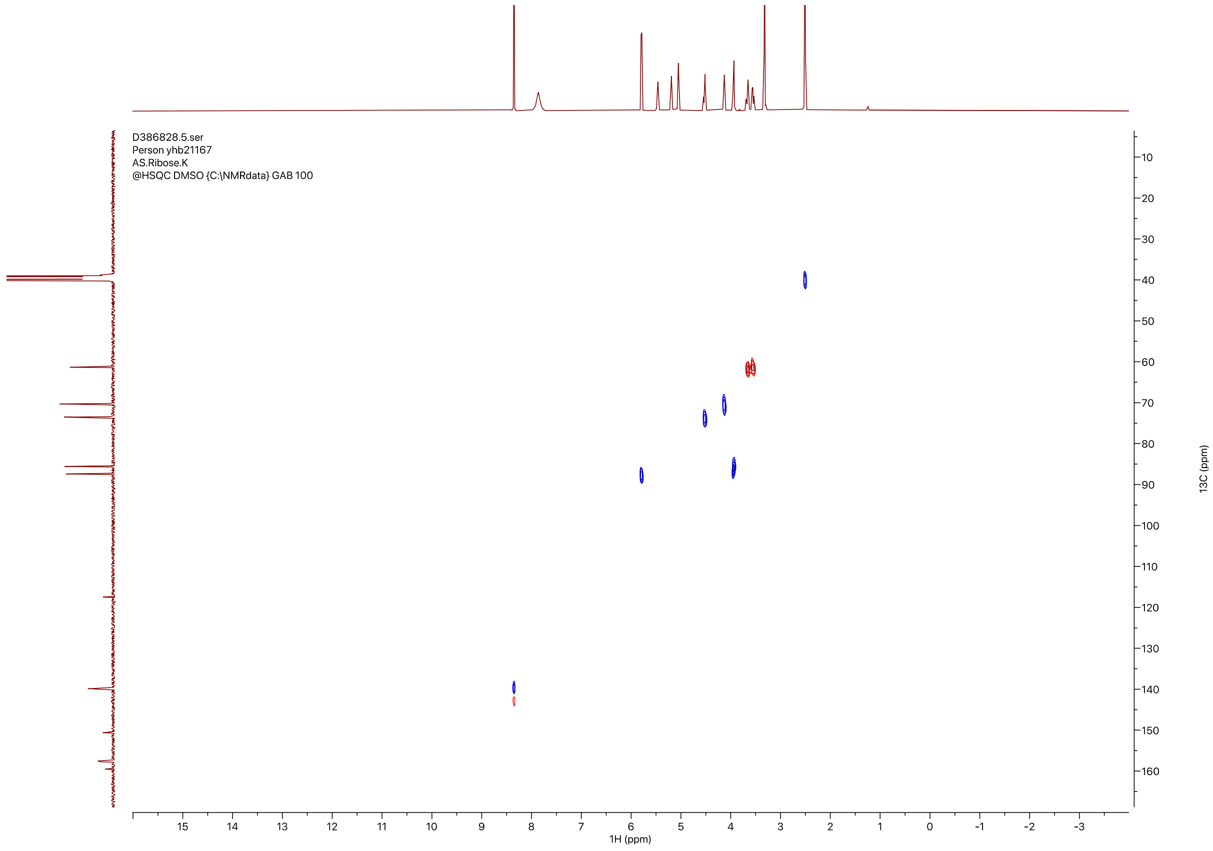

Figure S48 – HSQC of compound 12.

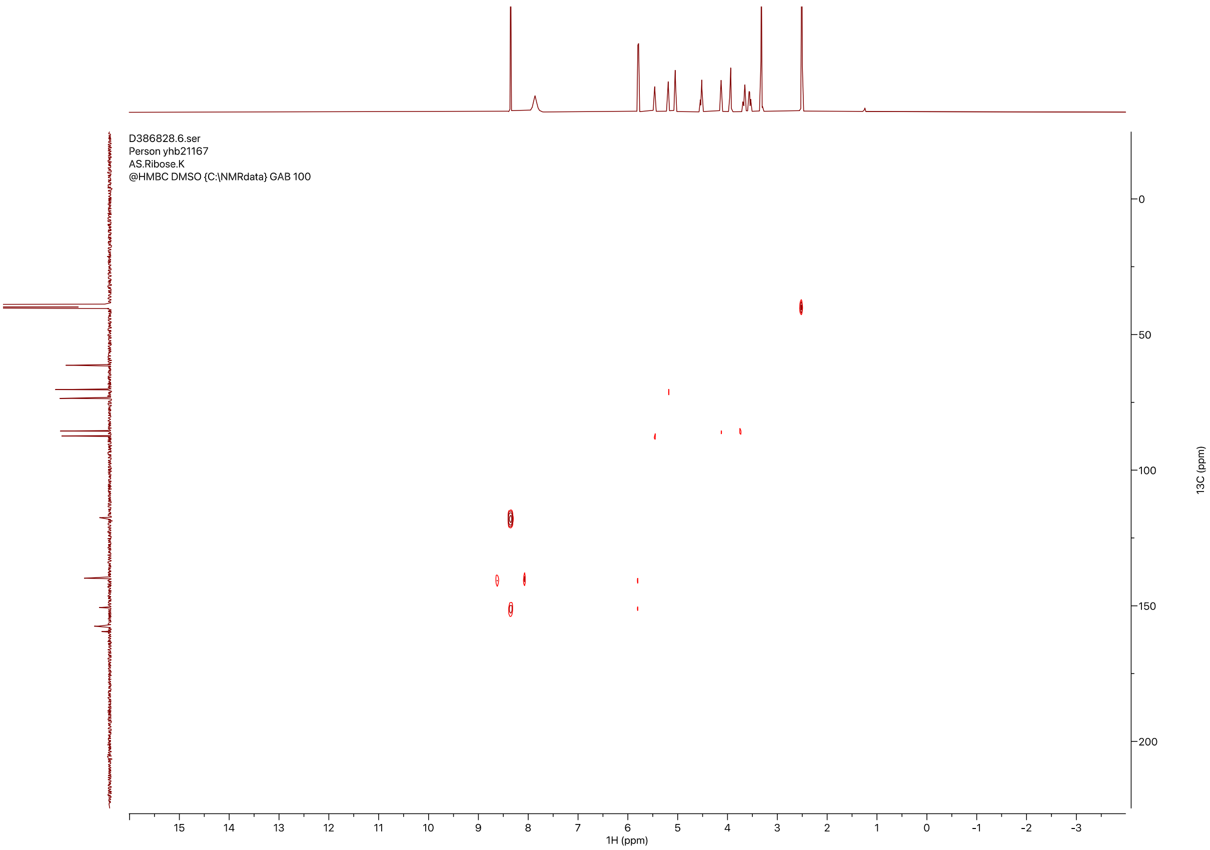

Figure S49 -HMBC of compound 12.

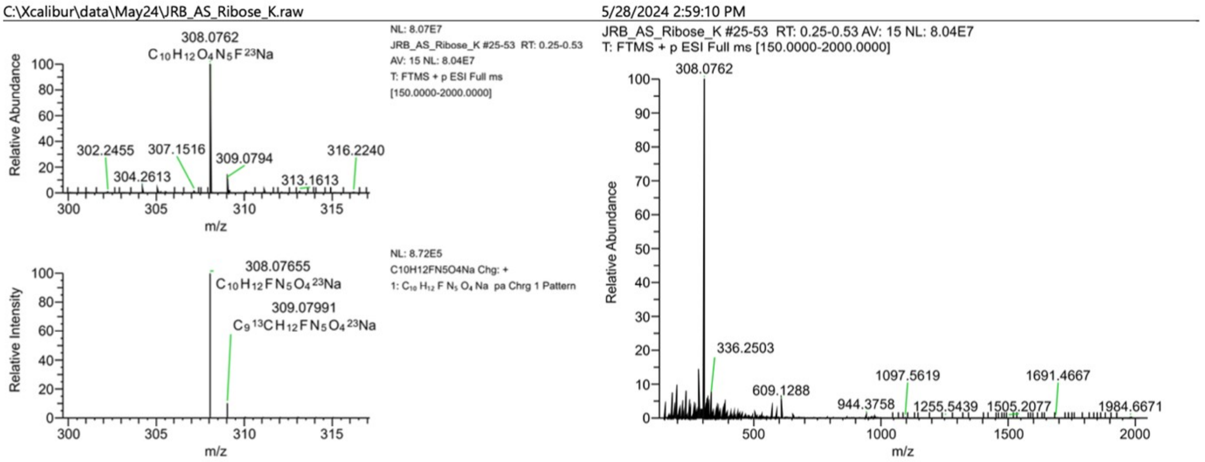

Peak Mass	Display For...	S Fit	RDB	Delta [ppm]	Theo. mass	Rank	Combined S...	# Matched I...	# Missed Iso.	MS Cov. [%]	Pattern Cov....	MSMS Matc...
308.0762	$C_{10}H_{12}O_4N_5F^{23}Na$	69.9480219530015	6.50	-1.21	308.07655	1	94.34	3	0	95.69	100	(Collection)
308.0762	$C_{13}H_8O_2N_8$	42.3509526704321	14.00	-0.95	308.07647	2	92.88	3	0	95.69	100	(Collection)
308.0762	$C_9H_{13}O_7N_4F$	28.9086398092613	5.00	-0.32	308.07628	3	91.35	4	0	94.82	98.35	(Collection)
308.0762	$C_9H_{16}O_8NF^{23}Na$	23.9323747131893	1.50	3.13	308.07522	4	91.09	4	0	94.82	98.16	(Collection)
308.0762	$C_8H_{10}O_3N_8F^{23}Na$	21.5178286783281	7.00	3.15	308.07521	5	90.96	3	1	94.82	96.54	(Collection)
308.0762	$C_8H_{17}O_{11}F$	19.174023023016	0.00	4.02	308.07494	6	90.84	4	0	94.82	97.64	(Collection)
308.0762	$C_{12}H_{12}O_6N_4$	18.143577193473	9.00	3.39	308.07514	7	90.79	4	0	94.82	98.33	(Collection)
308.0762	$C_{13}H_{11}O_3N_5{}^{23}Na$	31.5386710227055	10.50	2.50	308.07541	8	84.07	4	0	86.99	87.7	(Collection)
308.0762	$C_{14}H_{14}O_7N$	13.1283015572298	8.50	-0.97	308.07648	9	82.21	4	0	86.05	84.86	(Collection)
308.0762	$C_{15}H_{13}O_4N_2{}^{23}Na$	12.4059743475467	10.00	-1.86	308.07675	10	82.18	4	0	86.05	84.48	(Collection)

Figure S50 – HRMS of compound 12.

Sample Name : k
Sample ID :
Data Filename : 4 and K_19032024_005.lcd
Method Filename : Admir Trans glyco Run.lcm
Batch Filename : 4 and K.lcb
Vial # : 1-26
Injection Volume : 10 uL
Date Acquired : 19/03/2024 21:51:08
Date Processed : 19/03/2024 22:02:29

Sample Type : Unknown
Acquired by : Shimadzu
Processed by : Shimadzu

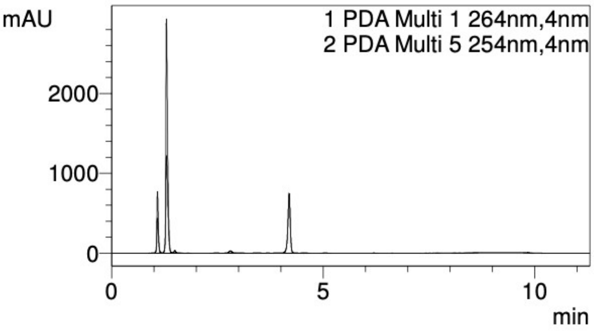

PDA Ch1 264nm

Peak#	Ret. Time	Area	Height	ID#
1	0.896	1029	468	
2	1.086	1905762	760215	
3	1.298	8430723	2840893	
4	1.495	102060	30828	
5	1.802	2755	416	
6	2.481	9299	1572	
7	2.810	142491	29716	
8	2.970	10788	1804	
9	3.093	6097	1514	
10	3.382	3642	859	
11	3.490	13763	3226	
12	3.698	6394	1849	
13	3.950	29391	3003	
14	4.198	2940712	736251	
15	4.506	1261	401	
16	4.591	4429	1179	
17	5.054	10368	2672	
18	6.201	2421	725	
19	7.709	1578	725	
20	8.101	2597	782	
21	8.222	1350	425	
22	8.395	1634	390	
23	8.536	1412	211	
24	8.629	1369	267	
25	9.838	5740	1462	
Total		13639065	4421852	

Figure S51 – HPLC trace of the reaction forming compound 12.

5-trifluorouridine (20)

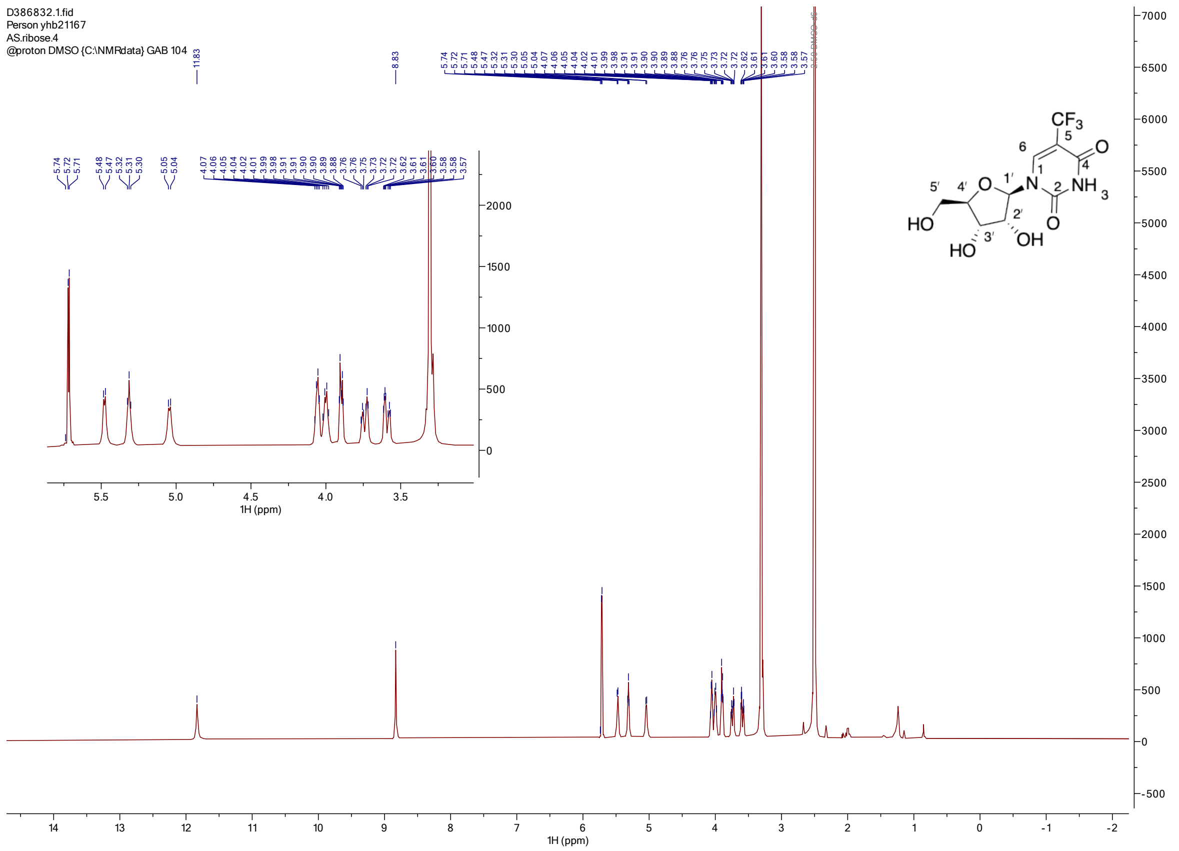

Figure S52 – 1H NMR of compound 20.

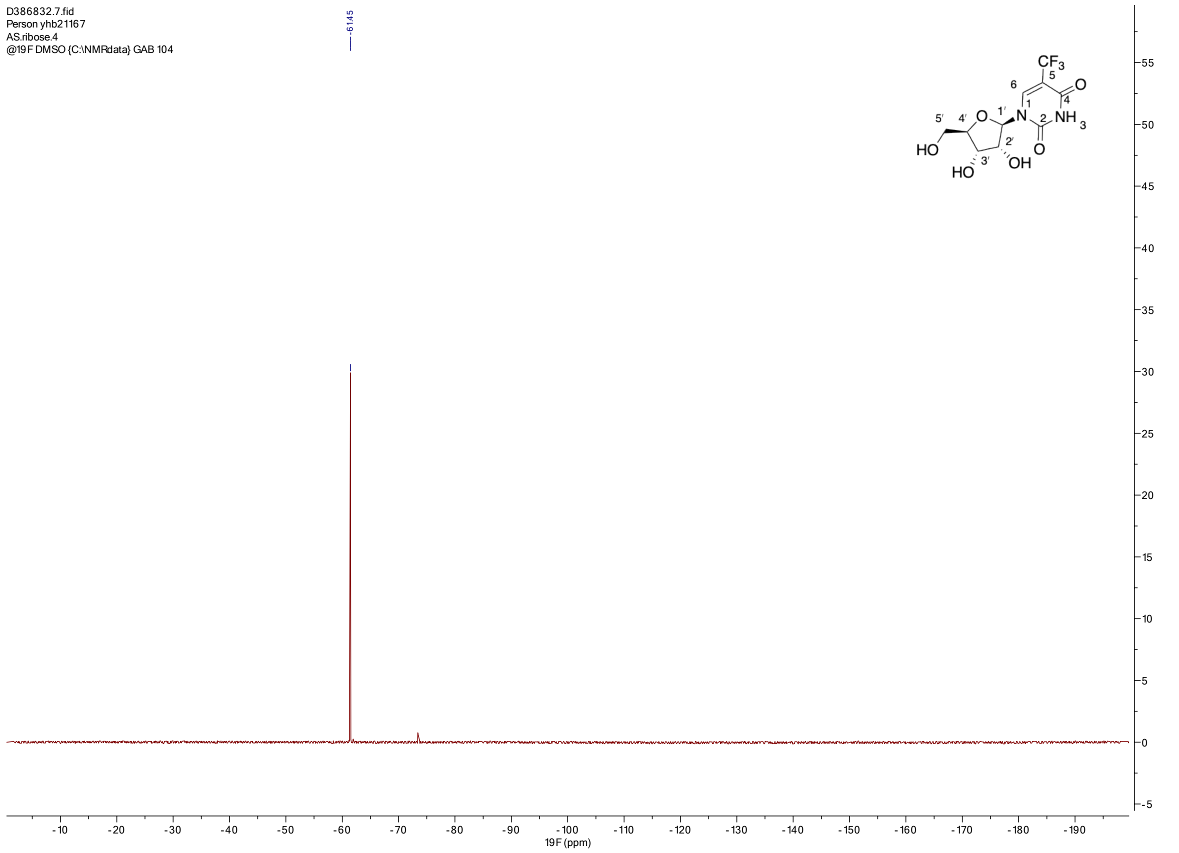

Figure S53 – 19F NMR of compound 20.

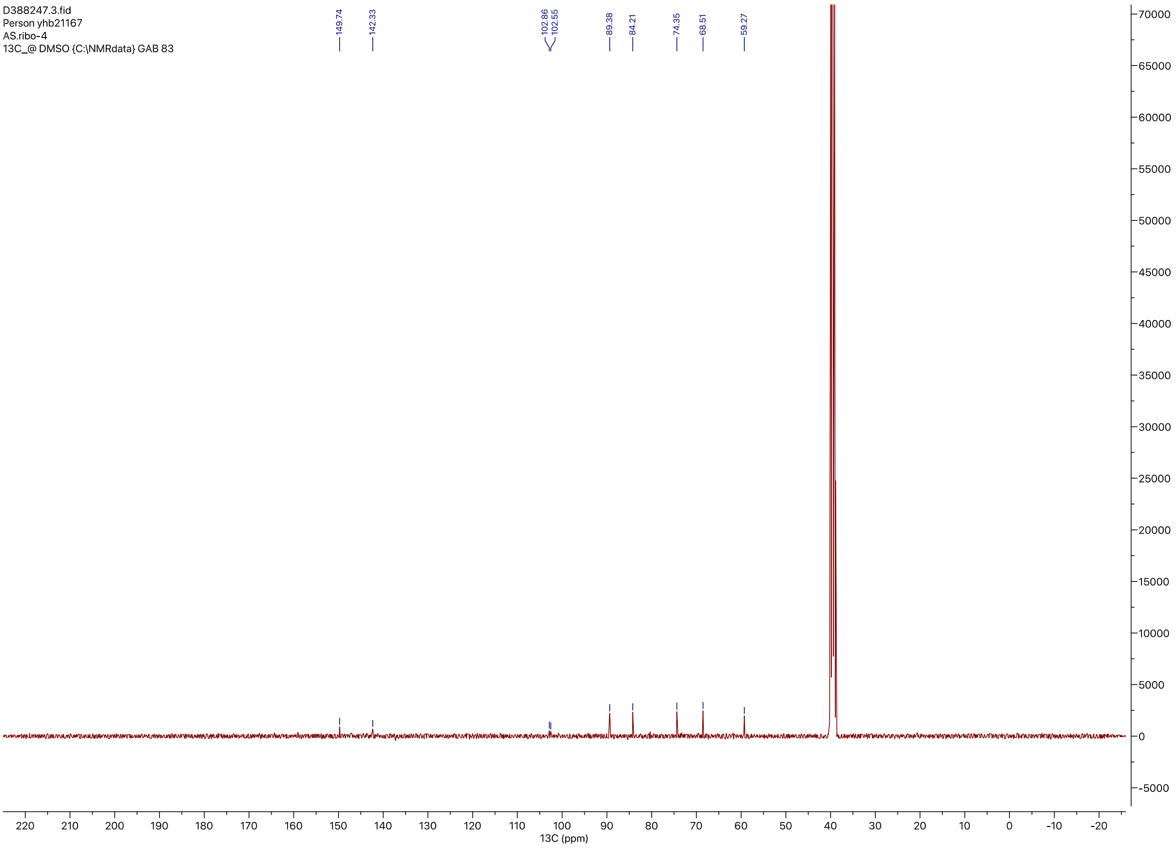

Figure S54 – 13C NMR of compound 20.

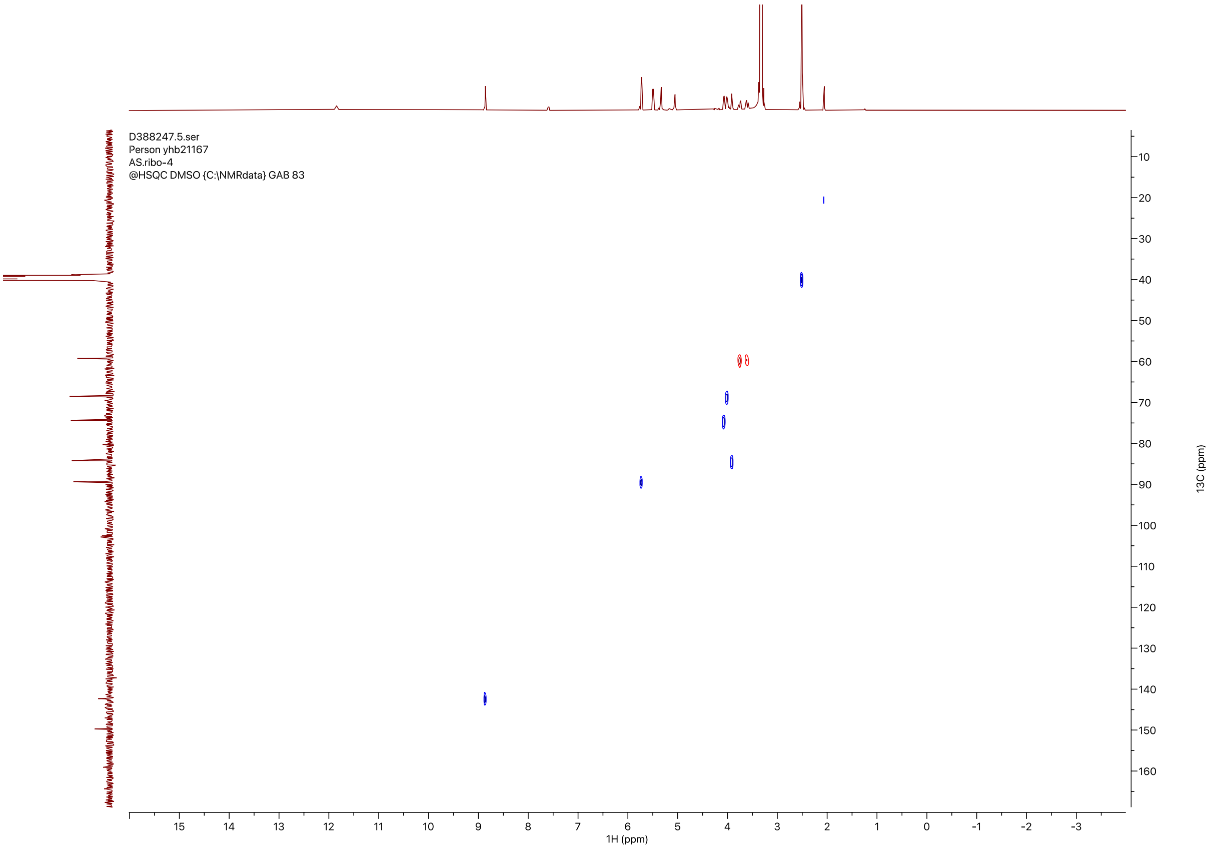

Figure S55 – HSQC of compound 20.

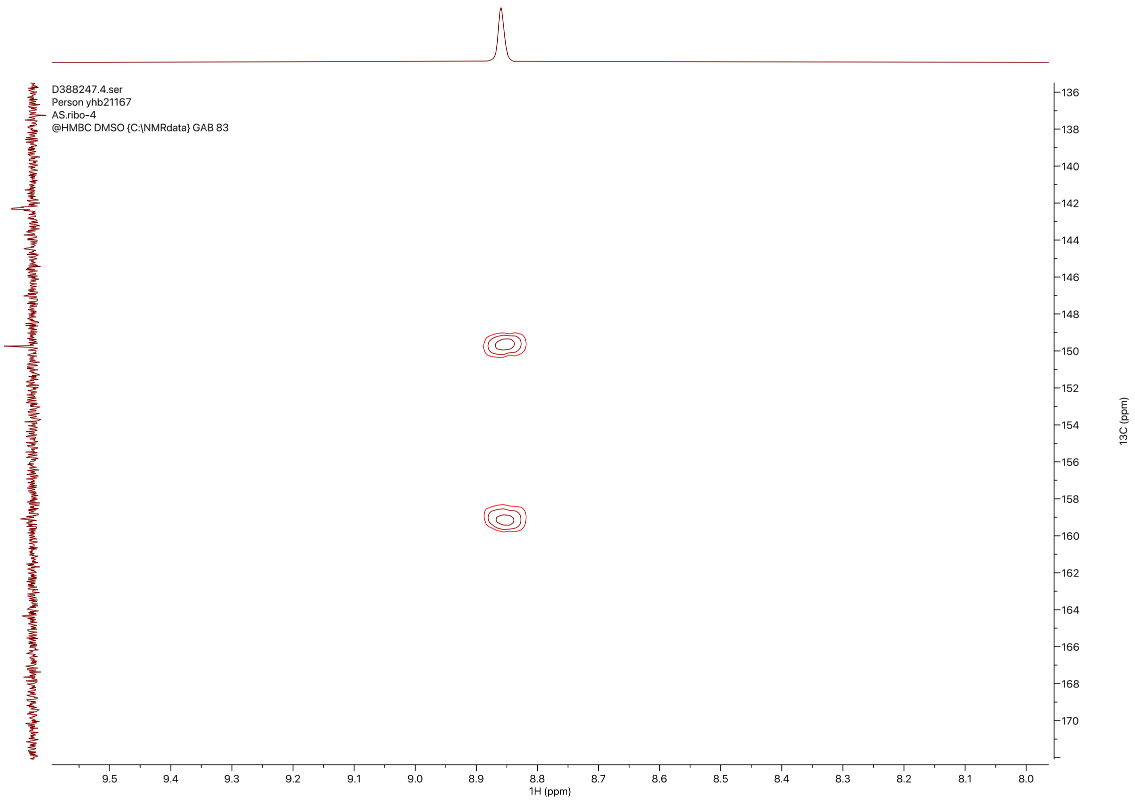

Figure S56 – HMBC of compound 20 showing the cross peaks from C6H to C4 and C6 .

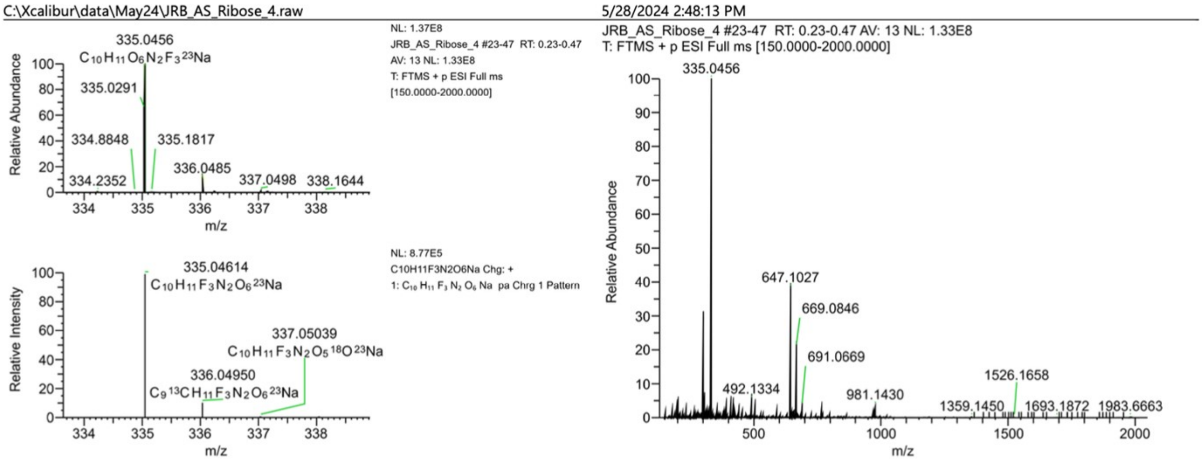

Peak Mass	Display For...	S Fit	RDB	Delta [ppm]	Theo. mass	Rank	Combined S...	# Matched I...	# Missed Iso.	MS Cov. [%]	Pattern Cov....	MSMS Matc...
335.0456	$C_8H_8O_6N_8{}^{23}Na$	56.4920500717264	8.50	-0.88	335.04590	1	94.82	4	0	96.95	100	(Collection)
335.0456	$C_8H_9O_5N_5F_3{}^{23}Na$	54.7954598882225	5.00	2.41	335.04480	2	94.62	3	0	96.84	100	(Collection)
335.0456	$C_{13}H_{10}O_5N_2F_2{}^{23}Na$	51.451718129944	8.50	1.81	335.04500	3	94.55	4	0	96.95	100	(Collection)
335.0456	$C_{10}H_{11}O_6N_2F_3{}^{23}Na$	39.282962396152	4.50	-1.60	335.04614	4	93.81	4	0	96.84	99.87	(Collection)
335.0456	$C_9H_5ON_9F_3{}^{23}Na$	35.7852551456597	10.00	-1.59	335.04614	5	92.38	2	1	95.53	99.47	(Collection)
335.0456	$C_{12}H_4N_9F_2{}^{23}Na$	35.5673647224505	14.00	1.83	335.04499	6	92.37	3	0	95.53	99.31	(Collection)
335.0456	$C_{11}H_5O_3N_8F_2$	35.476809990131	12.50	2.65	335.04472	7	92.37	3	0	95.53	98.98	(Collection)
335.0456	$C_8H_6O_4N_8F_3$	33.3421323877608	8.50	-0.77	335.04586	8	92.25	3	0	95.53	99	(Collection)
335.0456	$C_9H_{12}O_9NF_3$	26.9932747478188	3.00	-0.78	335.04587	9	91.92	4	0	95.53	97.93	(Collection)
335.0456	$C_9H_{14}O_{11}N{}^{23}Na$	26.8118592115407	3.00	-0.90	335.04591	10	91.91	4	0	95.53	97.54	(Collection)

Figure S57 – HRMS of compound 20.

Sample Name : 4
Sample ID :
Data Filename : runs_25032024_003.lcd
Method Filename : Admir Trans glyco Run.lcm
Batch Filename : runs.lcb
Vial # : 1-63
Injection Volume : 10 uL
Date Acquired : 25/03/2024 11:23:03
Date Processed : 25/03/2024 11:34:23

Sample Type : Unknown
Acquired by : Shimadzu
Processed by : Shimadzu

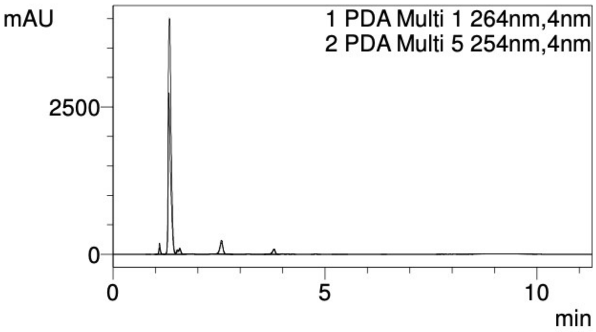

PDA Ch1 264nm

Peak#	Ret. Time	Area	Height	ID#
1	0.812	1298	445	
2	1.100	436475	183918	
3	1.335	19166266	3987437	
4	1.579	549655	99232	
5	1.867	45744	8776	
6	2.335	14001	1922	
7	2.562	1019476	194018	
8	3.204	21302	3751	
9	3.445	3556	637	
10	3.801	414742	87331	
11	4.006	26488	8010	
12	4.119	7373	1620	
13	4.249	6173	1663	
14	4.789	9635	2255	
15	5.239	1833	533	
16	5.438	3056	758	
17	6.377	1158	423	
18	7.674	1571	581	
19	7.754	6667	3310	
20	8.137	1373	462	
21	8.248	1232	307	
22	8.418	2607	1179	
23	8.650	2779	324	
24	9.608	4613	175	
25	9.850	7752	1689	
Total		21756823	4590757	

Figure S58 – HPLC trace of the reaction forming compound 20.

5-fluorouridine (19)

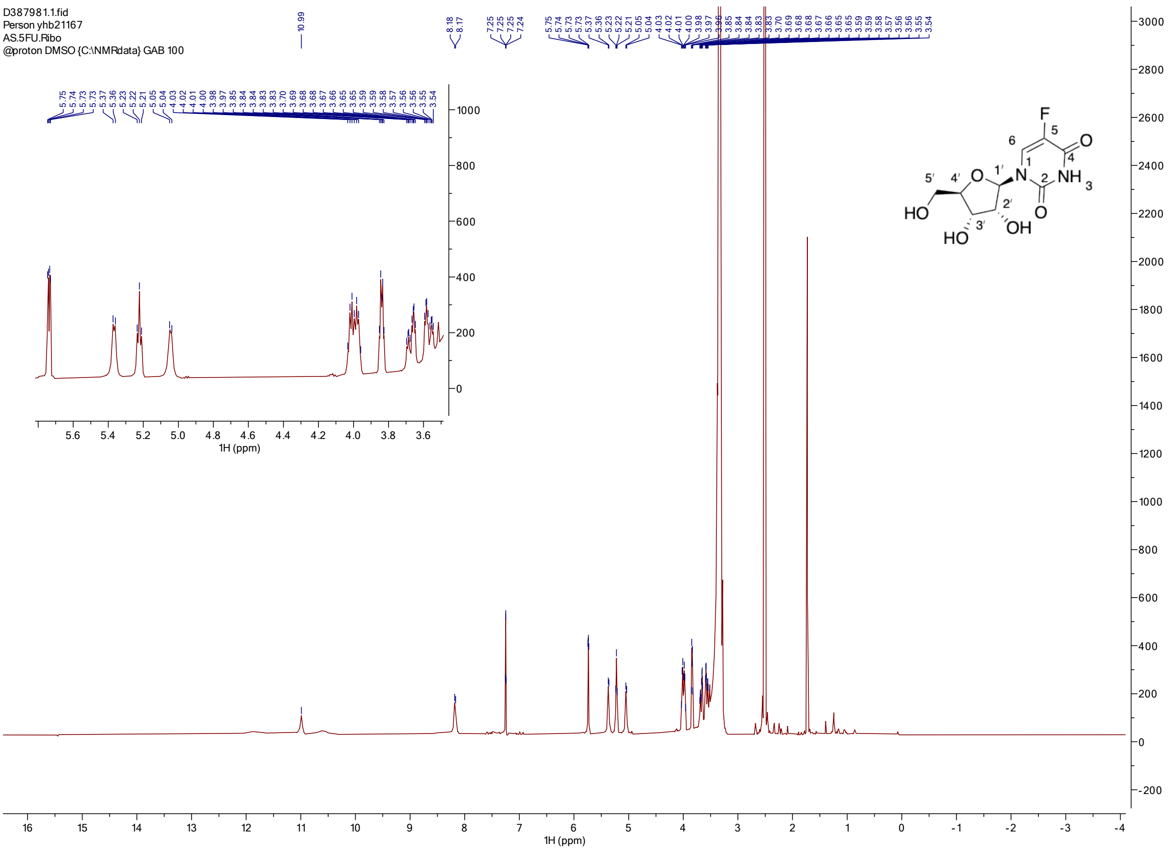

Figure S59 – 1H NMR of compound 19.

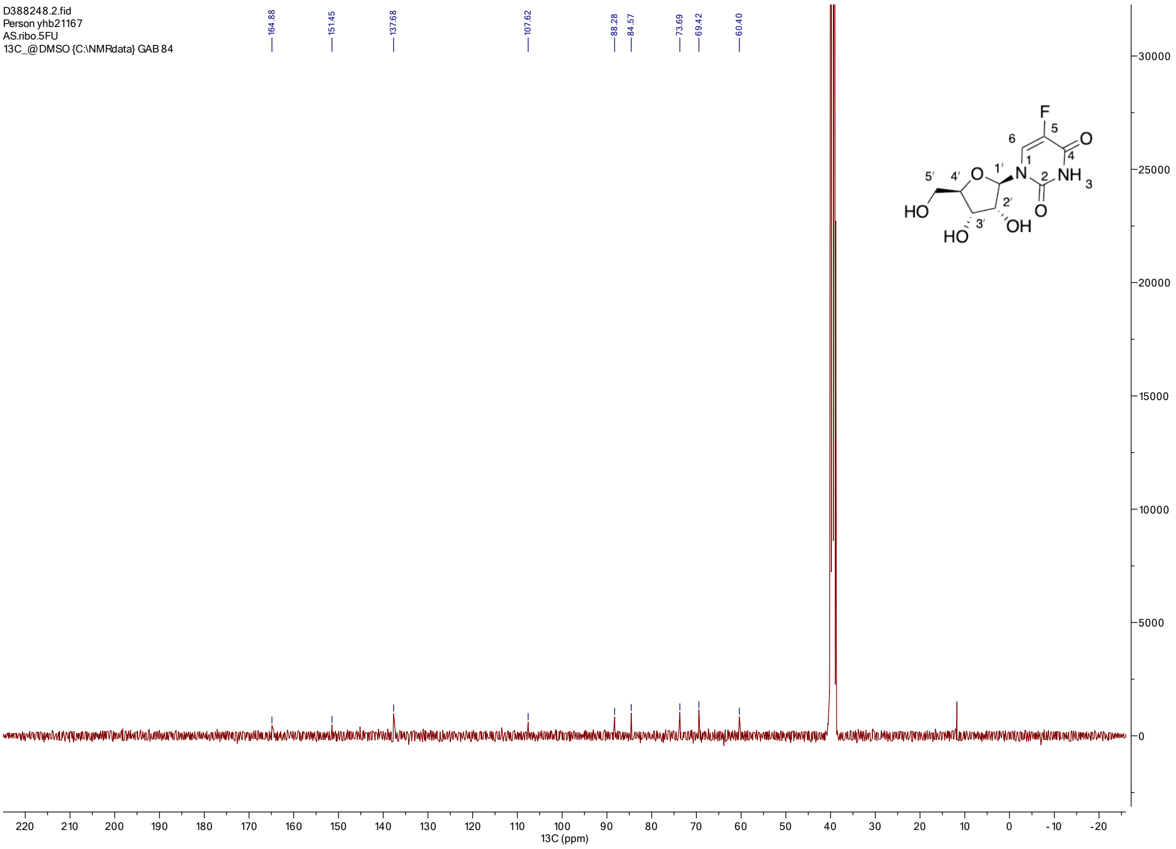

Figure S60 – 13C NMR of compound 20.

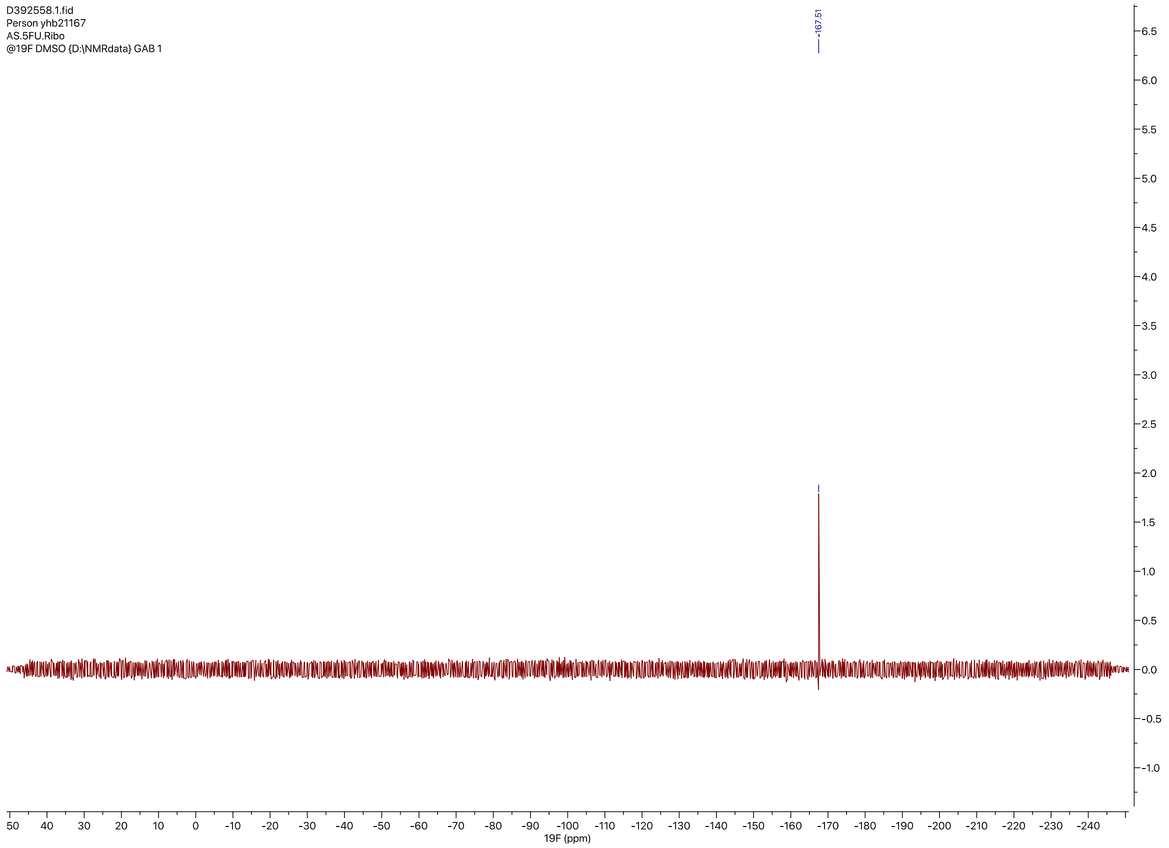

Figure S61 – 19F NMR of compound 19.

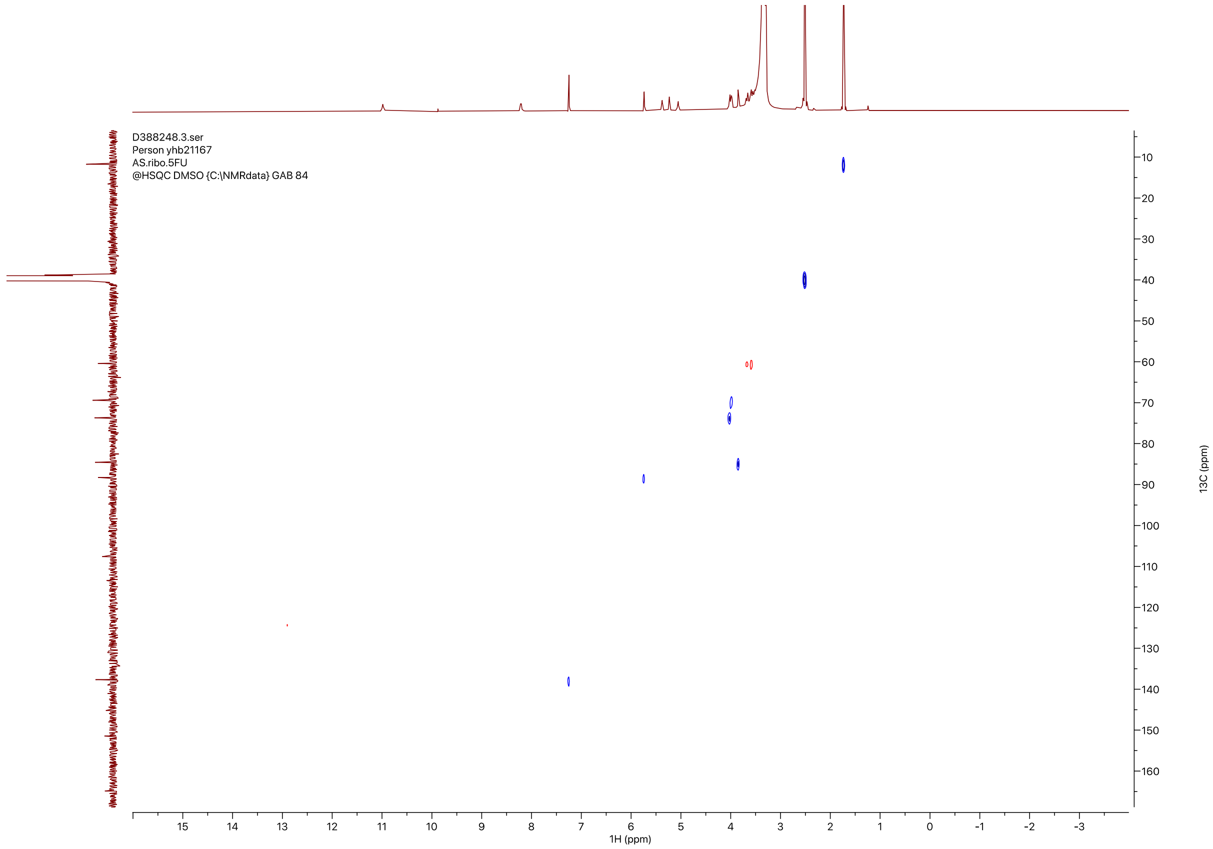

Figure S62 – HSQC NMR of compound 19.

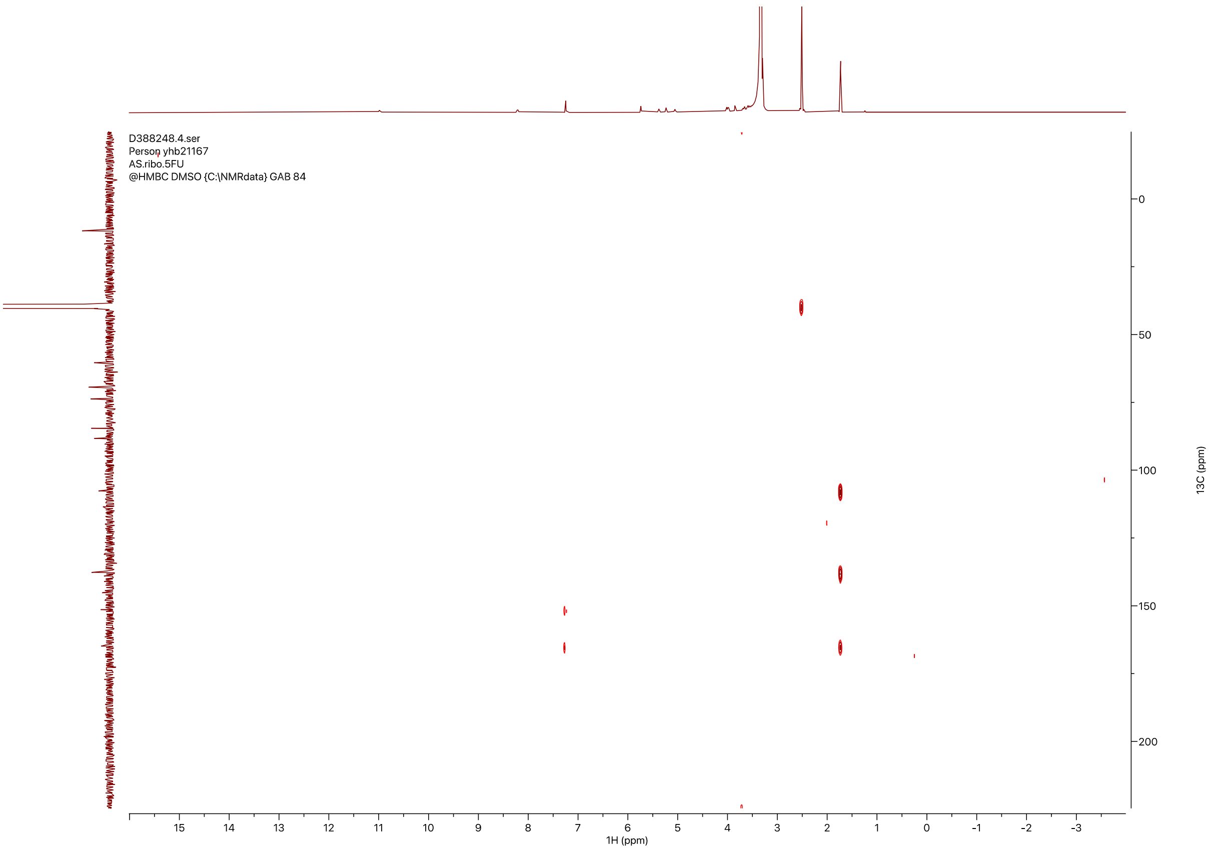

Figure S63 – HMBC of compound 19.

Sample Name	: 5fu		
Sample ID	:		
Data Filename	: runs_25032024_009.lcd		
Method Filename	: Admir Trans glyco Run.lcm		
Batch Filename	: runs.lcb		
Vial #	: 1-69	Sample Type	: Unknown
Injection Volume	: 10 uL		
Date Acquired	: 25/03/2024 12:33:44	Acquired by	: Shimadzu
Date Processed	: 25/03/2024 12:45:05	Processed by	: Shimadzu

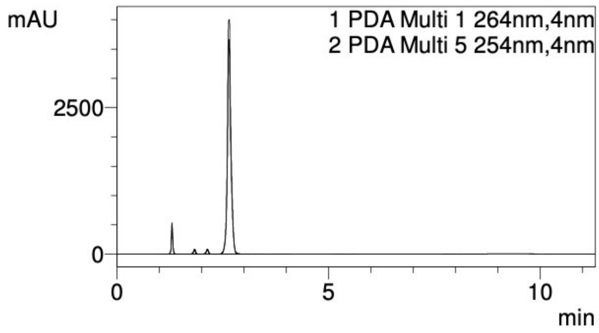

PDA Ch1 264nm

Peak#	Ret. Time	Area	Height	ID#
1	1.101	2527	1008	
2	1.301	1272519	526964	
3	1.573	11251	3081	
4	1.834	332304	86571	
5	2.137	347871	83057	
6	2.649	24597207	3999476	
7	3.291	8911	2381	
8	3.828	1258	340	
9	5.315	14013	3638	
10	5.521	6969	1557	
11	7.740	6092	3309	
12	8.131	1542	500	
13	8.238	1498	363	
14	8.406	2973	1285	
15	8.643	3251	330	
16	9.835	4439	1133	
Total		26614625	4714993	

Figure S64 – HPLC trace of the reaction forming compound 19.

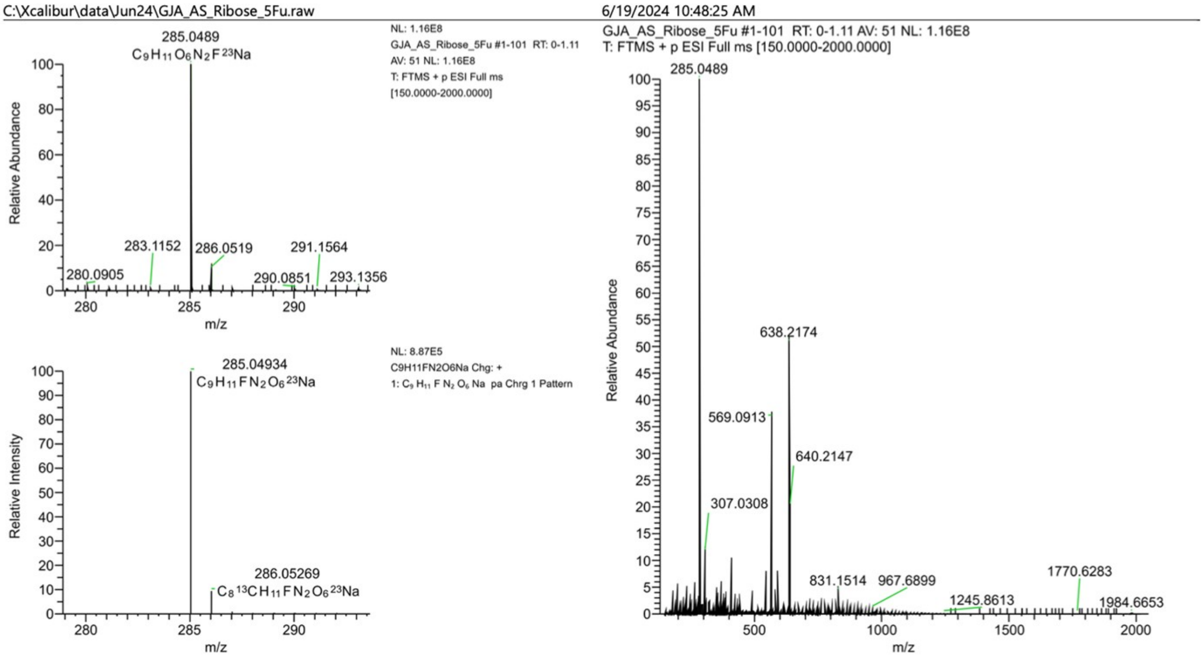

Peak Mass	Display For...	S Fit	RDB	Delta [ppm]	Theo. mass	Rank	Combined S...	# Matched I...	# Missed Iso.	MS Cov. [%]	Pattern Cov....	MSMS Matc...
285.0489	$C_9H_{11}O_6N_2F^{23}Na$	38.5049989938708	4.50	-1.54	285.04934	1	94.44	4	0	97.54	99.89	(Collection)
285.0489	$C_8H_{12}O_9NF$	27.1968420739867	3.00	-0.58	285.04906	2	92.7	4	0	96.33	98.05	(Collection)
285.0489	$C_{10}H_5O_3N_8$	22.5287572650977	12.50	3.45	285.04791	3	92.45	3	1	96.33	96.57	(Collection)
285.0489	$C_8H_5ON_9F^{23}Na$	19.075783065629	10.00	-1.53	285.04933	4	92.27	2	3	96.33	96.35	(Collection)

Figure S65 – HRMS of compound 19.

5-fluorocytidine (23)

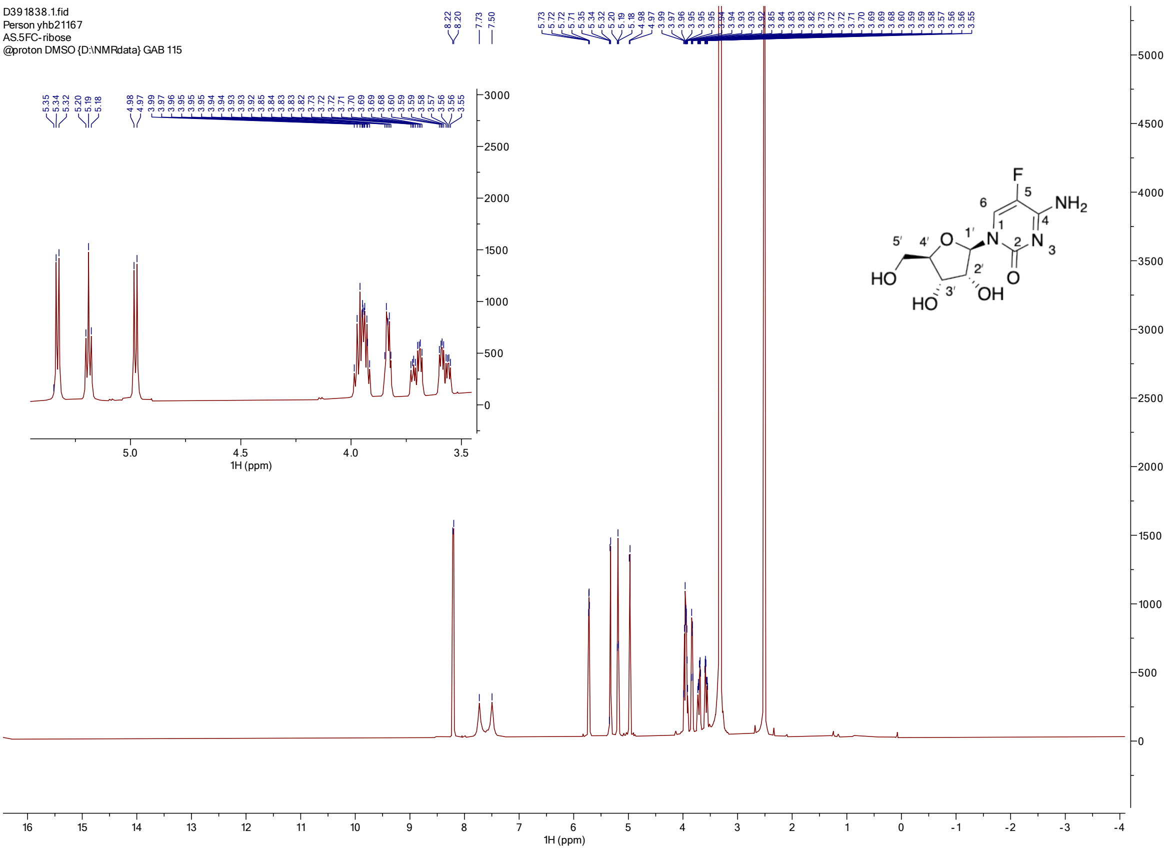

Figure S66 – 1H NMR of compound 23.

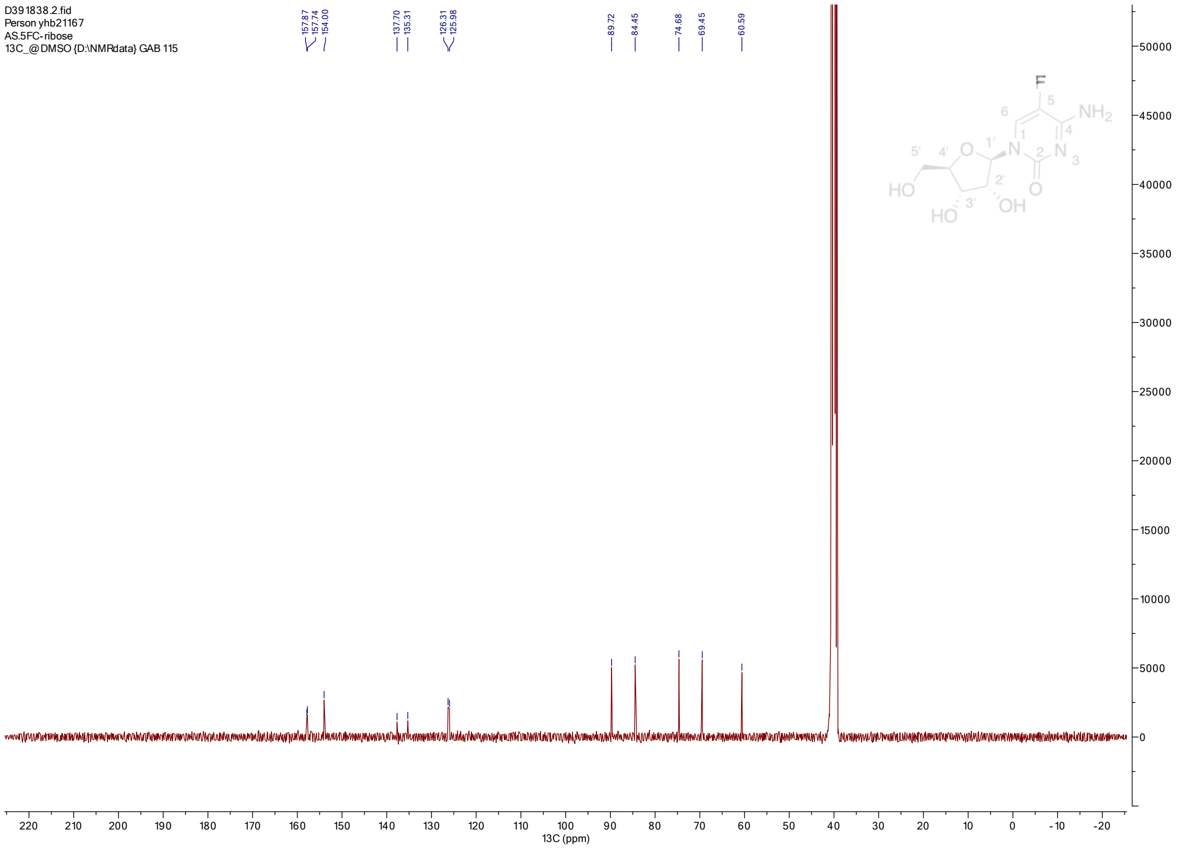

Figure S67 – 13C NMR of compound 23.

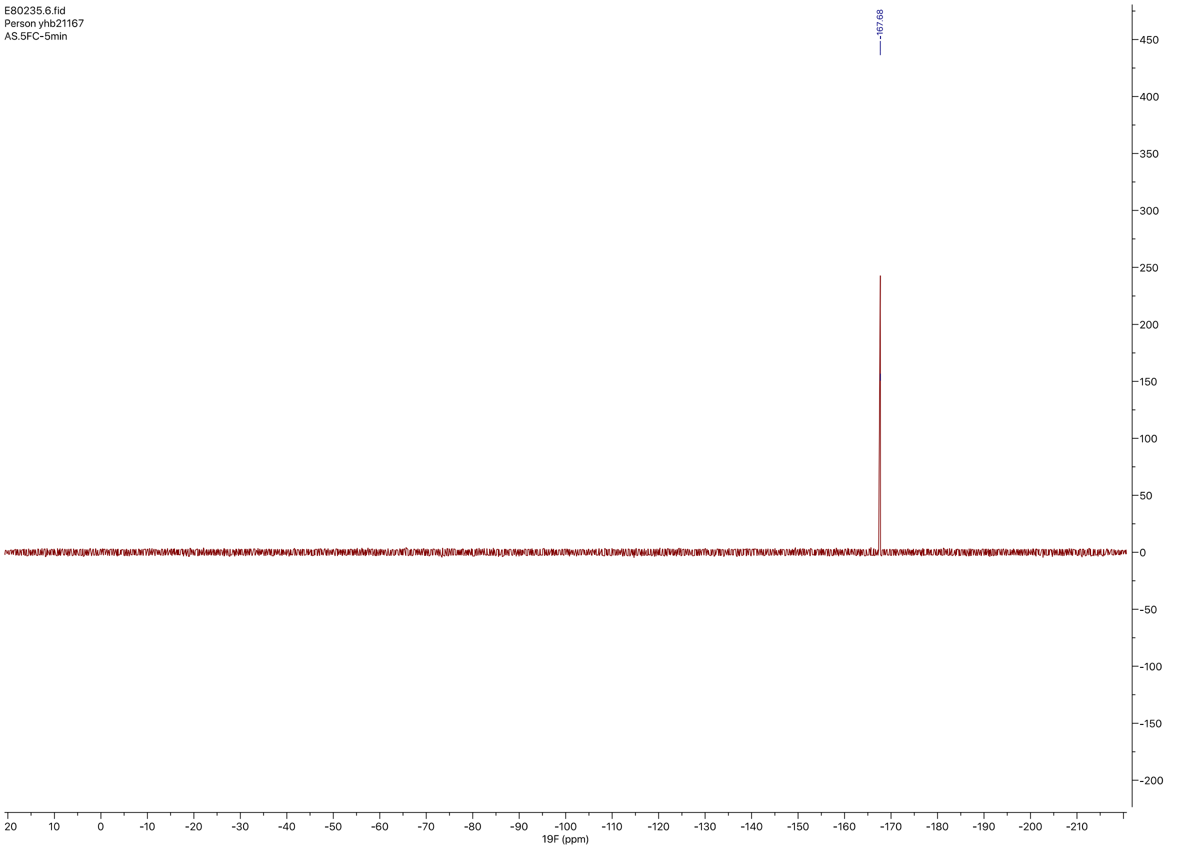

Figure S68 – 19F NMR of compound 23.

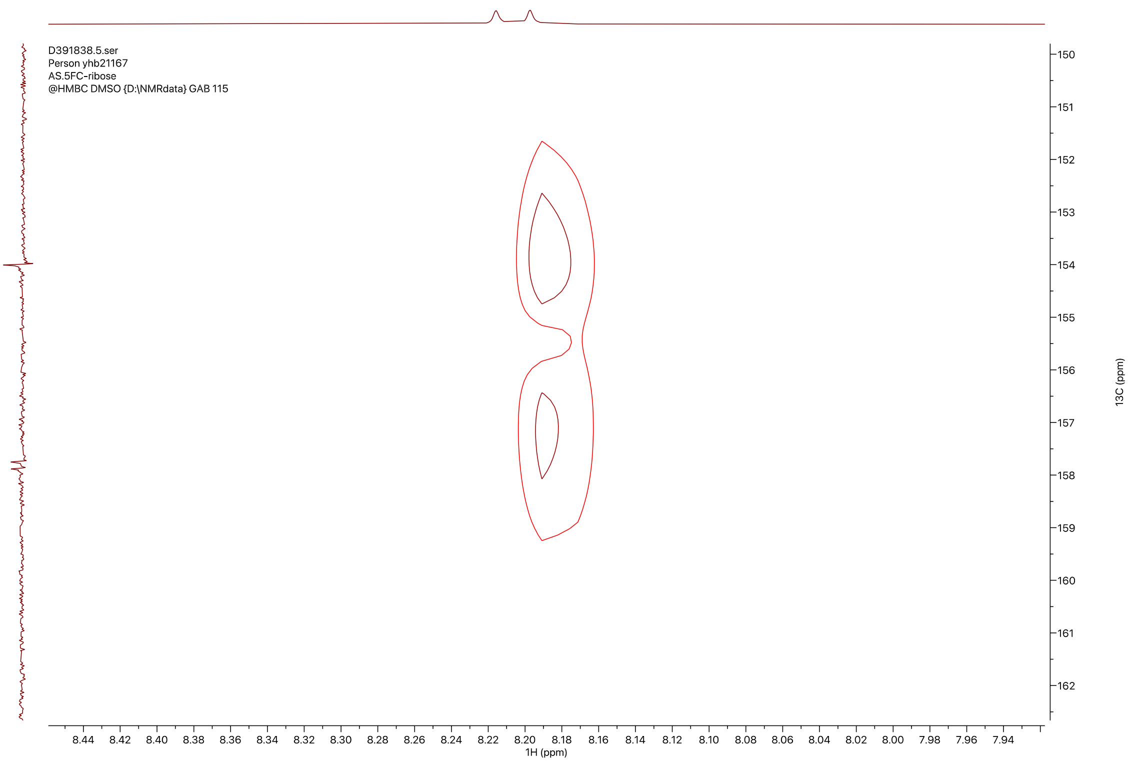

Figure S69 – HMBC cross peak from C6H showing C2 and C4 of compound 23.

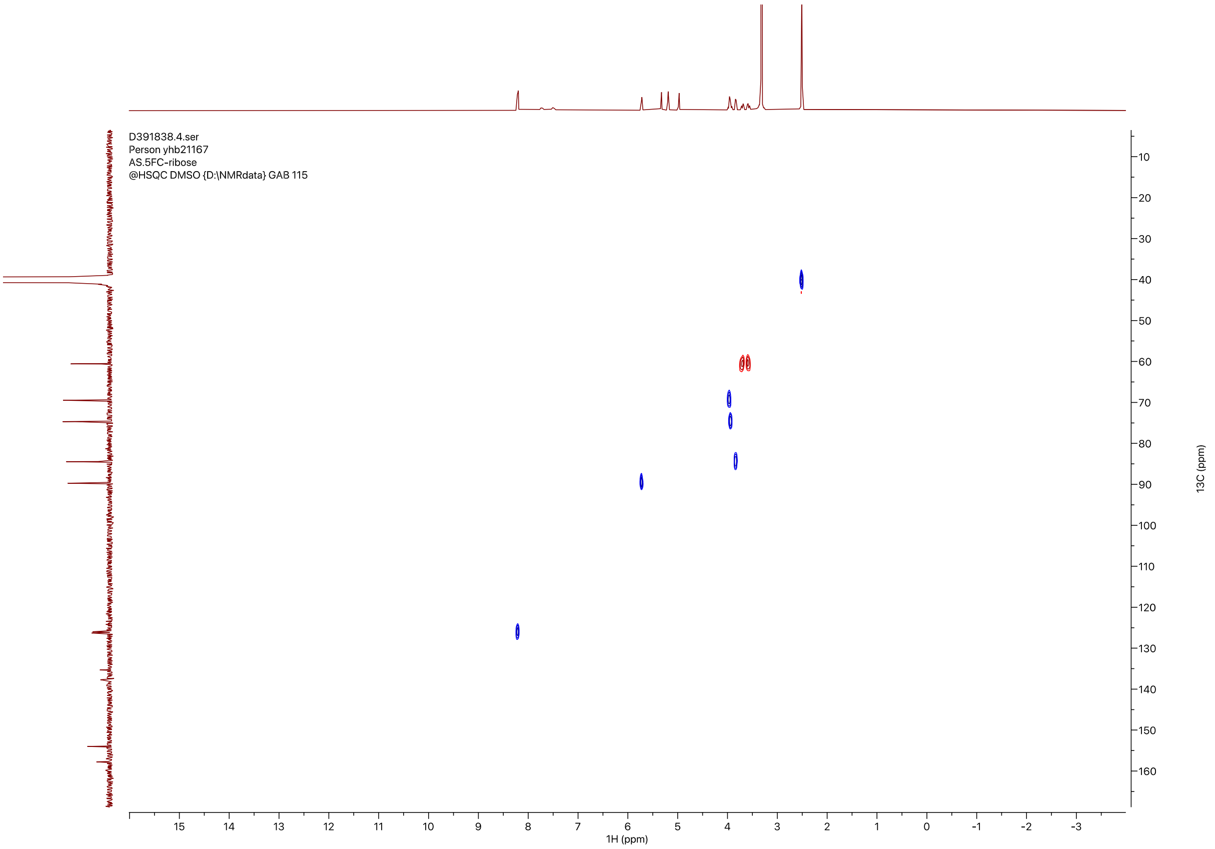

Figure S70 – HSQC of compound 23.

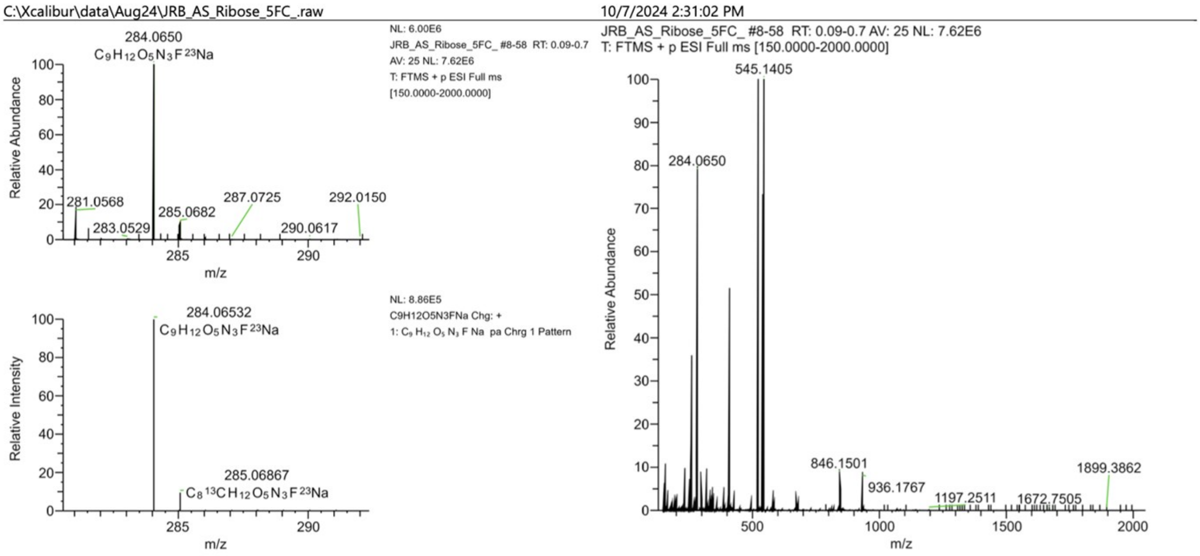

Peak Mass	Display For...	S Fit	RDB	Delta [ppm]	Theo. mass	Rank	Combined S...	# Matched I...	# Missed Iso.	MS Cov. [%]	Pattern Cov....	MSMS Matc...
284.0650	$C_9H_{12}O_5N_3F^{23}Na$	65.0461358456206	4.50	-1.28	284.06532	1	97.46	4	0	99.26	100	(Collection)
284.0650	$C_8H_{13}O_8N_2F$	43.6204500577607	3.00	-0.31	284.06504	2	95.38	4	0	98.26	98.37	(Collection)
284.0650	$C_7H_7O_3N_9F$	22.5697156594378	8.50	-0.29	284.06504	3	94.18	3	1	98.15	96.32	(Collection)
284.0650	$C_8H_6N_{10}F^{23}Na$	20.2990211876009	10.00	-1.26	284.06531	4	94.06	2	3	98.15	96.12	(Collection)
284.0650	$C_{10}H_6O_2N_9$	20.2649317595876	12.50	3.73	284.06390	5	94.05	3	1	98.15	96.4	(Collection)
284.0650	$C_{11}H_{12}O_7N_2$	18.0376657110187	7.00	3.72	284.06390	6	93.94	4	0	98.15	98.28	(Collection)
284.0650	$C_7H_{10}O_4N_6F^{23}Na$	21.0471261338303	5.00	3.45	284.06398	7	86.8	3	1	90.46	90.99	(Collection)

Figure S71 – HRMS of compound 23.

Sample Name	: 5fc		
Sample ID	:		
Data Filename	: runs_25032024_012.lcd		
Method Filename	: Admir Trans glyco Run.lcm		
Batch Filename	: runs.lcb		
Vial #	: 1-72	Sample Type	: Unknown
Injection Volume	: 10 uL		
Date Acquired	: 25/03/2024 13:09:09	Acquired by	: Shimadzu
Date Processed	: 25/03/2024 13:20:29	Processed by	: Shimadzu

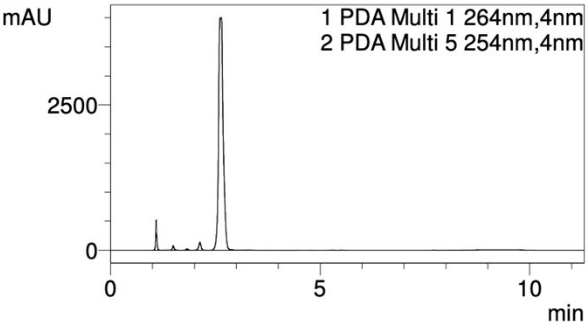

PDA Ch1 264nm

Peak#	Ret. Time	Area	Height	ID#
1	1.090	1147930	499388	
2	1.299	14404	4984	
3	1.381	10964	3719	
4	1.498	261206	80238	
5	1.830	94508	24687	
6	2.134	581259	141964	
7	2.641	33131722	3999966	
8	3.284	13193	3510	
9	3.818	1625	478	
10	4.332	1224	307	
11	4.852	1056	211	
12	5.306	20654	5386	
13	5.510	9762	2194	
14	7.698	7660	3574	
15	7.773	1524	668	
16	8.126	1712	454	
17	8.236	1625	383	
18	8.403	3183	1271	
19	8.638	3568	434	
20	8.758	1454	251	
21	9.829	3579	1217	
Total		35313811	4775284	

Figure S72 – HPLC trace of the reaction to form compound 23.

5-methylcytosine (24)

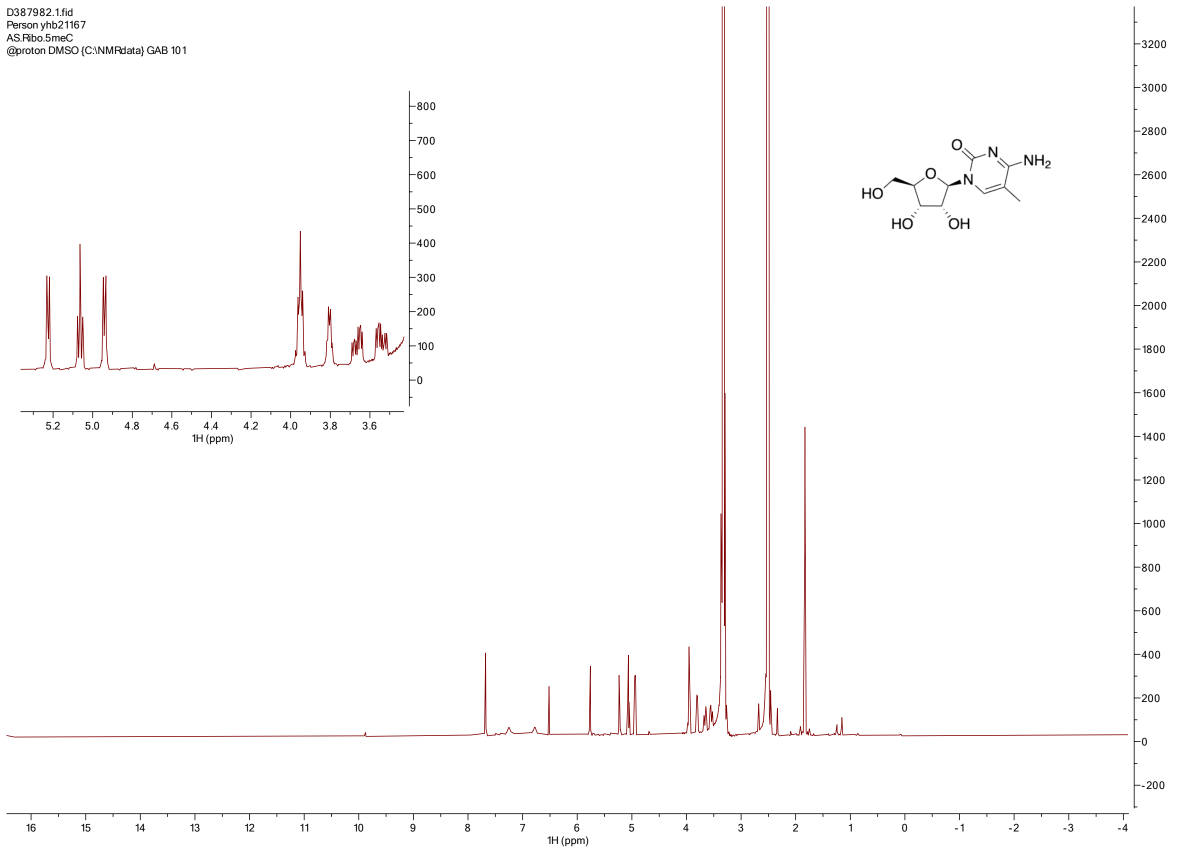

Figure S73 – 1H NMR of compound 24.

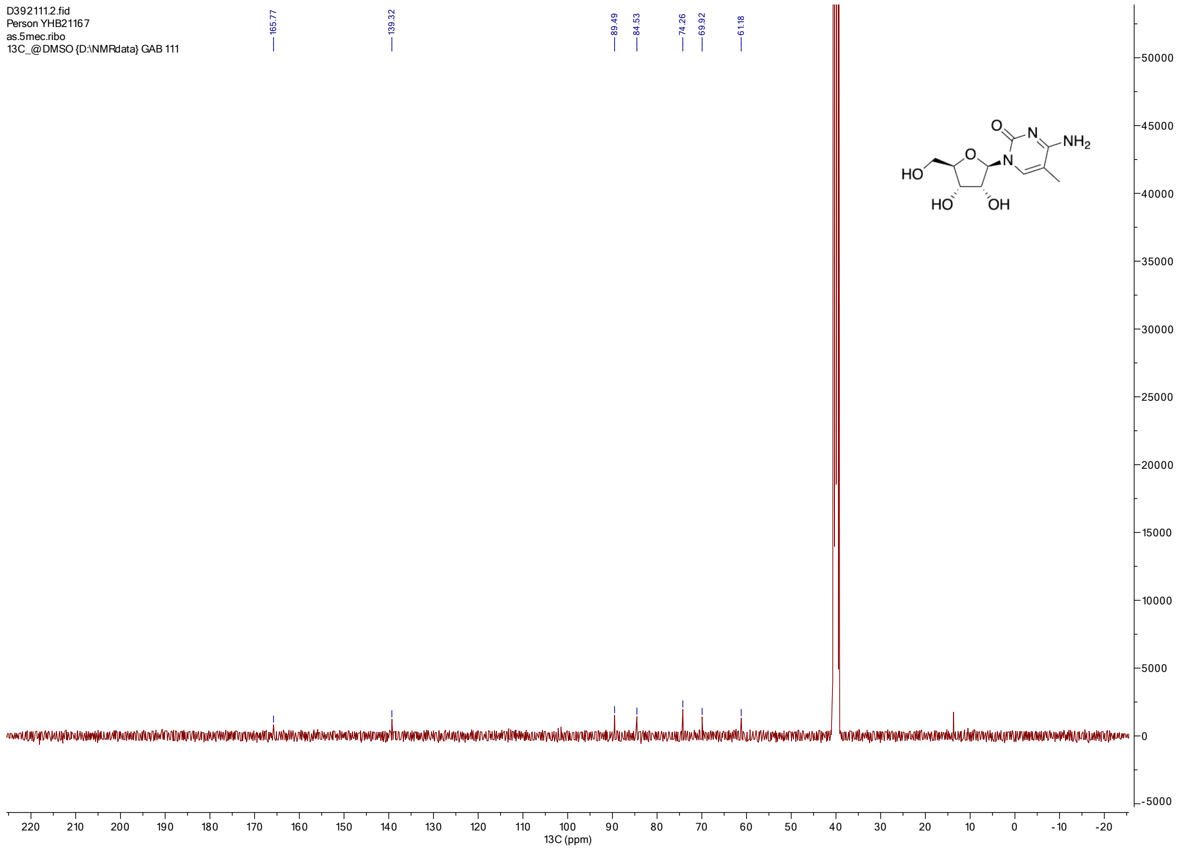

Figure S74 – 13C NMR of compound 24

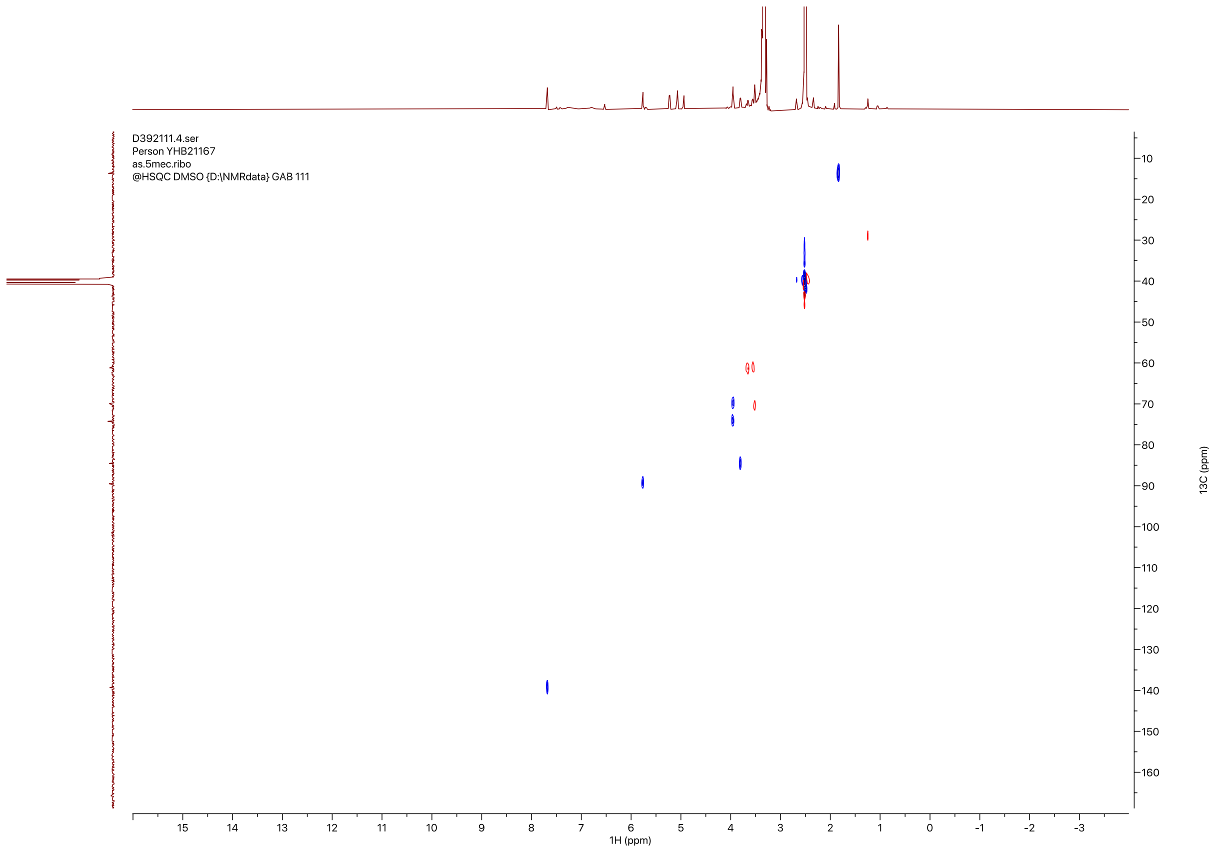

Figure S75 – HSQC NMR of compound 24

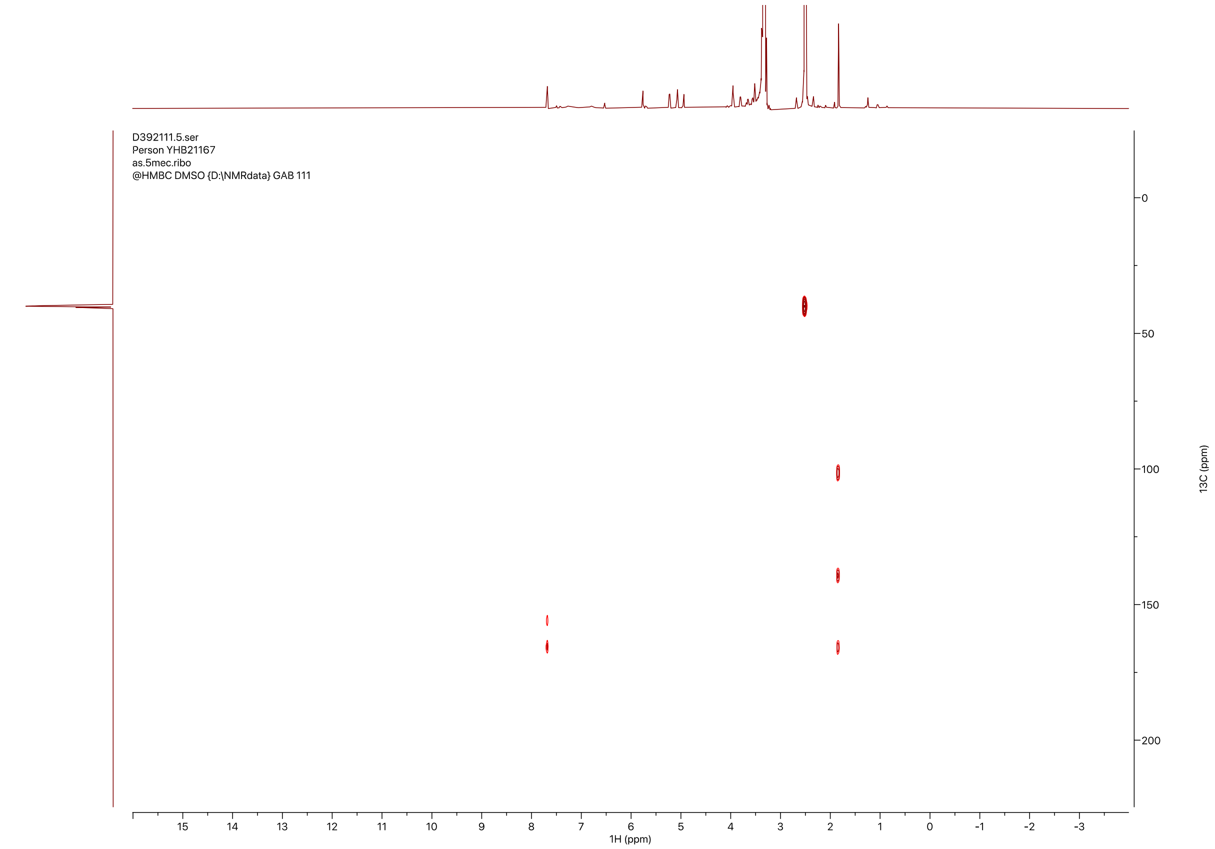

Figure S76 – HMBC of compound 24

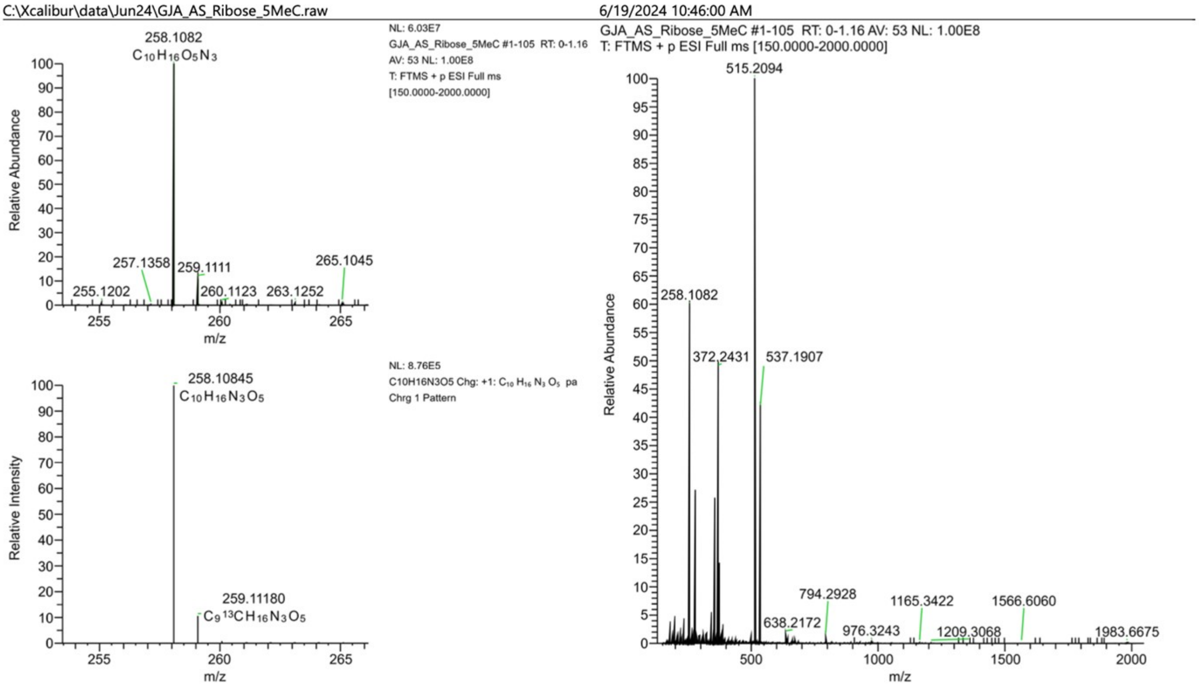

Peak Mass	Display For...	S Fit	RDB	Delta [ppm]	Theo. mass	Rank	Combined S...	# Matched I...	# Missed Iso.	MS Cov. [%]	Pattern Cov....	MSMS Matc...
258.1082	$C_{10}H_{16}O_5N_3$	41.42716720 88332	4.50	-1.05	258.10845	1	95.38	5	0	98.38	99.02	(Collection)
258.1082	$C_9H_{10}N_{10}$	18.89430870 94556	10.00	-1.03	258.10844	2	93.24	3	2	97.37	96.08	(Collection)
258.1082	$C_8H_{14}O_4N_6$	20.34446717 34124	5.00	4.16	258.10710	3	84.82	4	0	88.4	90.15	(Collection)

Figure S77 – HRMS of compound 24

Sample Name : 5mec
Sample ID :
Data Filename : runs_25032024_015.lcd
Method Filename : Admir Trans glyco Run.lcm
Batch Filename : runs.lcb
Vial # : 1-75
Sample Type : Unknown
Injection Volume : 10 uL
Date Acquired : 25/03/2024 13:44:34
Acquired by : Shimadzu
Date Processed : 25/03/2024 13:55:54
Processed by : Shimadzu

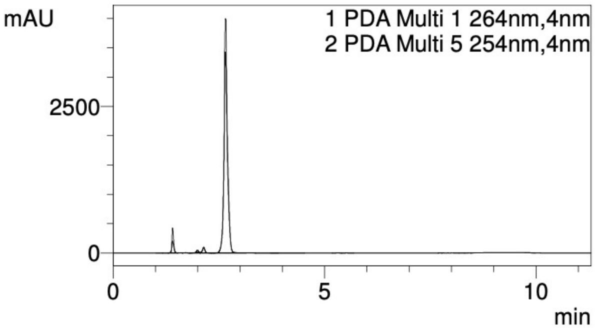

PDA Ch1 264nm

Peak#	Ret. Time	Area	Height	ID#
1	1.104	1428	534	
2	1.280	1687	663	
3	1.408	1256485	420196	
4	1.580	14131	3670	
5	1.716	21834	6044	
6	1.996	220504	47941	
7	2.144	423138	102246	
8	2.663	23397647	3993146	
9	3.298	8643	2258	
10	3.833	1134	308	
11	5.319	13480	3449	
12	5.525	6481	1407	
13	7.726	6377	3509	
14	8.138	1367	404	
15	8.249	1302	284	
16	8.419	2685	1211	
17	8.656	2816	338	
18	9.829	4795	782	
Total		25385932	4588390	

Figure S78 – HPLC trace of the reaction forming compound 24.

5-ethynyluridine (21)

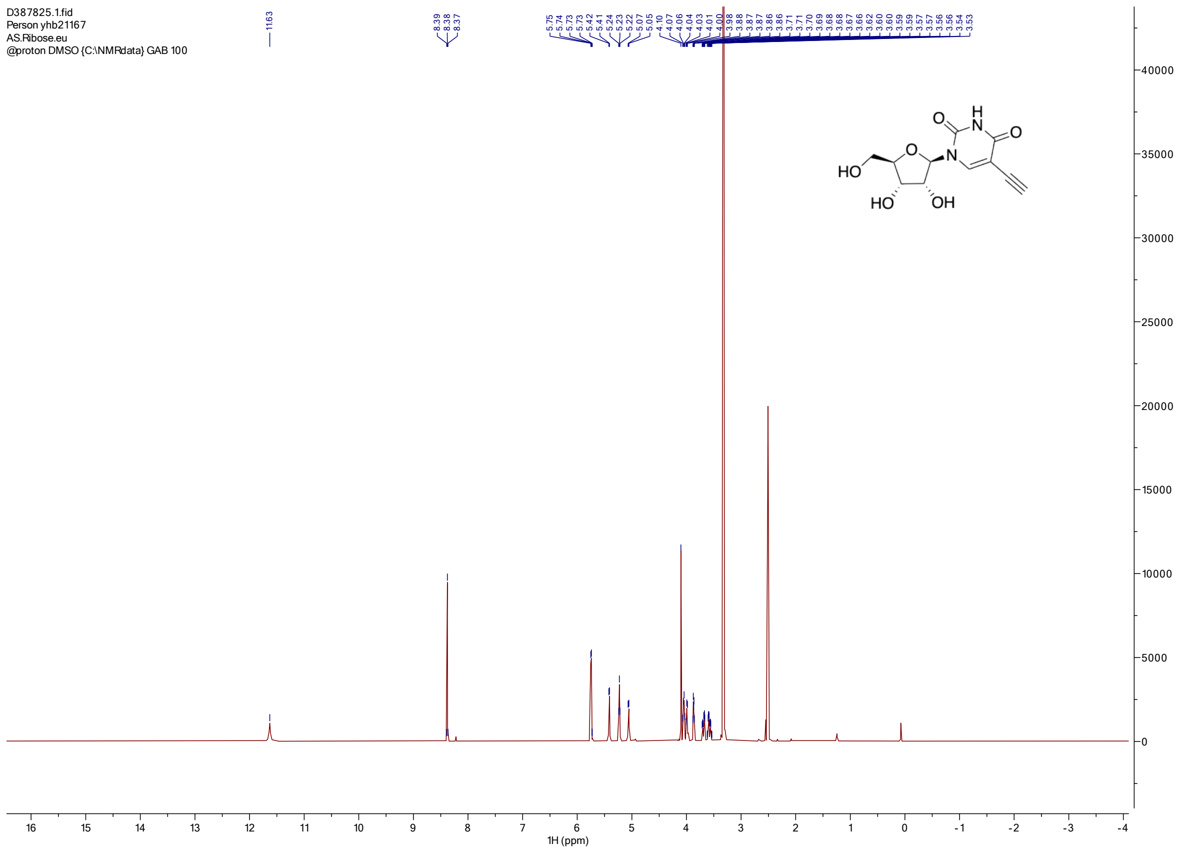

Figure S79 – 1H NMR of compound 21.

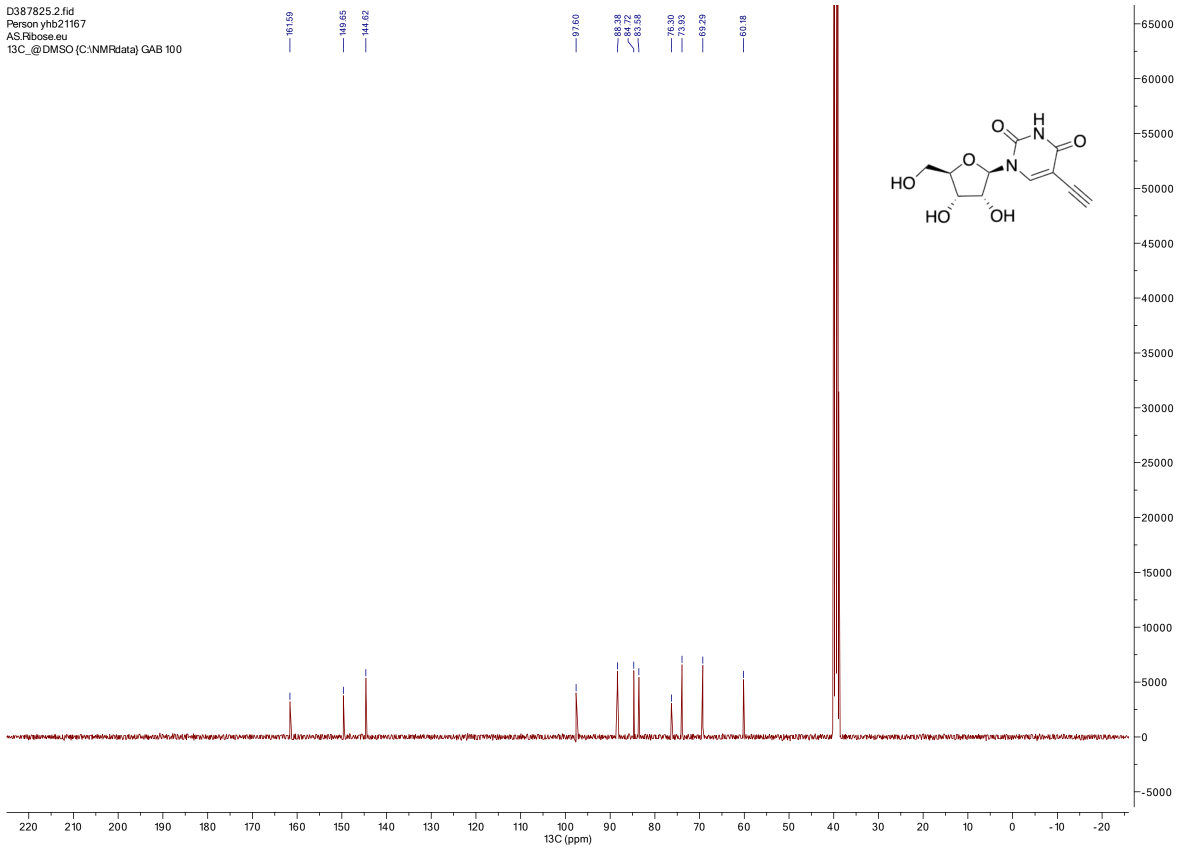

Figure S80 – 13C NMR of compound 21.

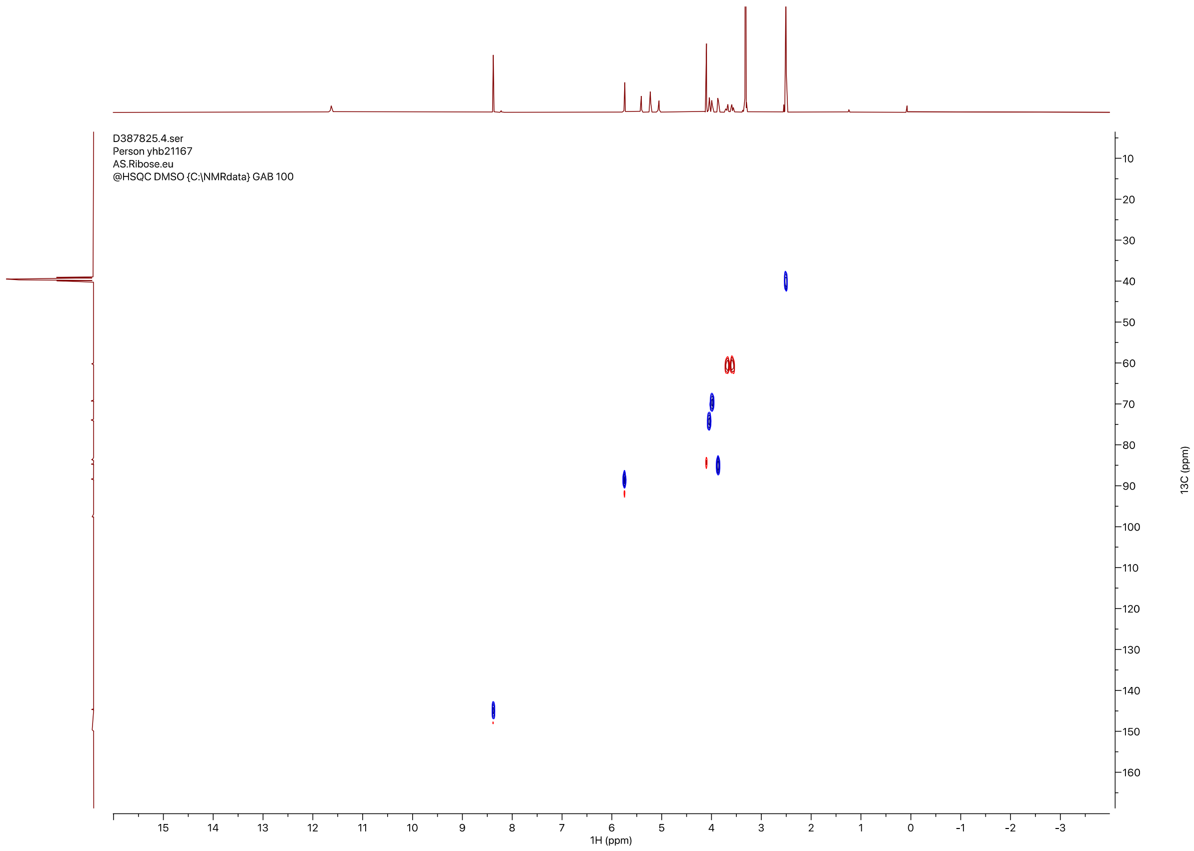

Figure S81 – HSQC of compound 21.

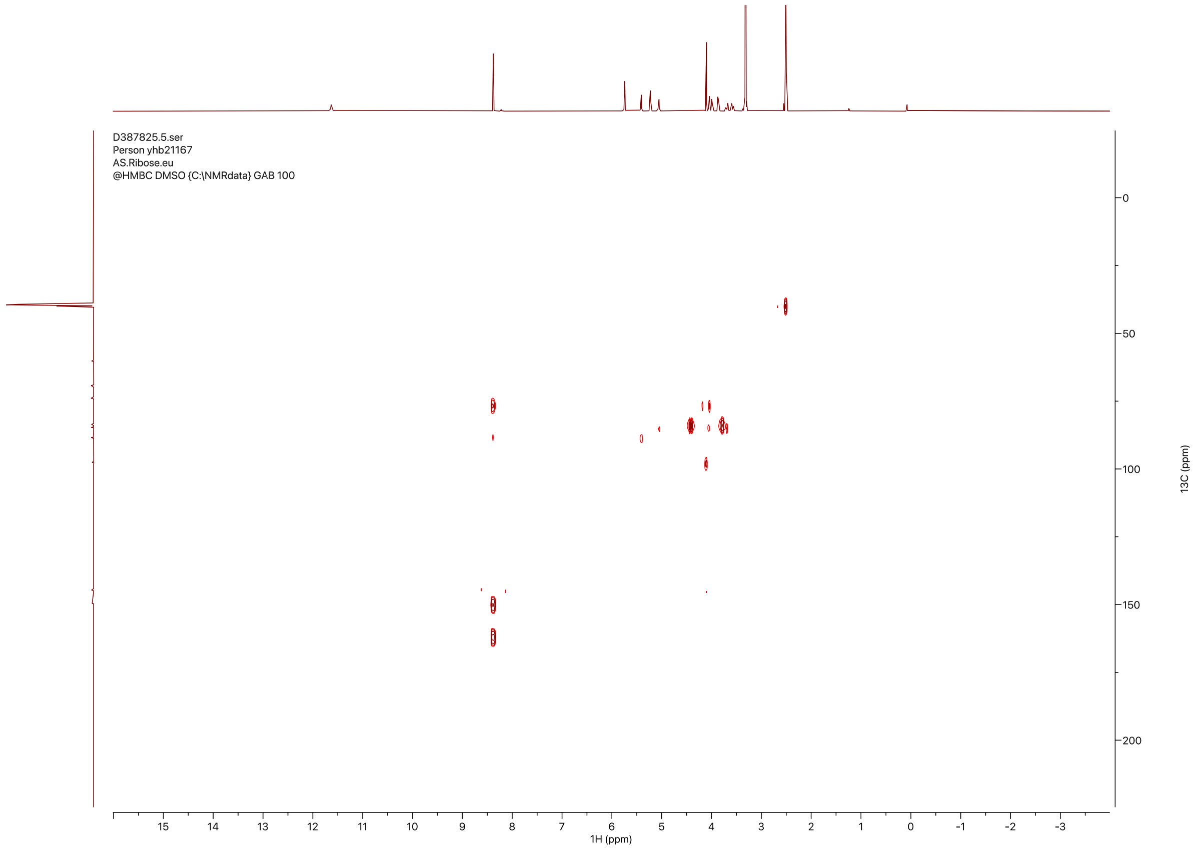

Figure S82- HMBC of compound 21.

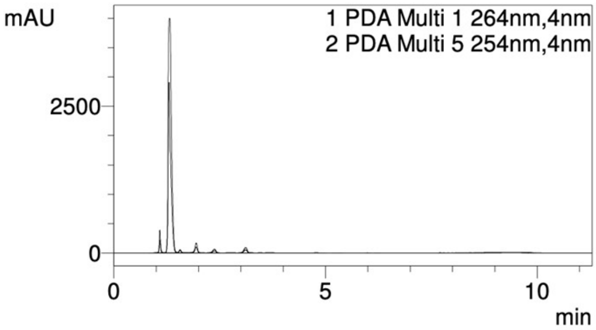

PDA Ch1 264nm

Peak#	Ret. Time	Area	Height	ID#
1	0.883	1943	708	
2	1.088	827400	370228	
3	1.320	22148341	3997720	
4	1.567	204948	52801	
5	1.948	761837	168168	
6	2.377	367320	64706	
7	2.769	26860	2141	
8	3.116	529536	93281	
9	3.464	11123	1788	
10	3.727	32700	4233	
11	3.941	2243	360	
12	4.088	2100	327	
13	4.229	2256	335	
14	4.478	4041	409	
15	4.788	12807	2326	
16	4.944	1123	206	
17	5.296	1234	164	
18	5.990	1392	504	
19	6.235	1618	356	
20	7.704	4052	1815	
21	7.787	1303	483	
22	8.134	1729	404	
23	8.247	1622	366	
24	8.414	3643	1332	
25	8.651	4694	479	
26	8.771	1944	323	
27	9.000	2488	129	
28	9.656	3247	151	
29	9.843	5898	1514	
Total		24971441	4767754	

Figure S83 – HPLC trace of the reaction forming compound 21.

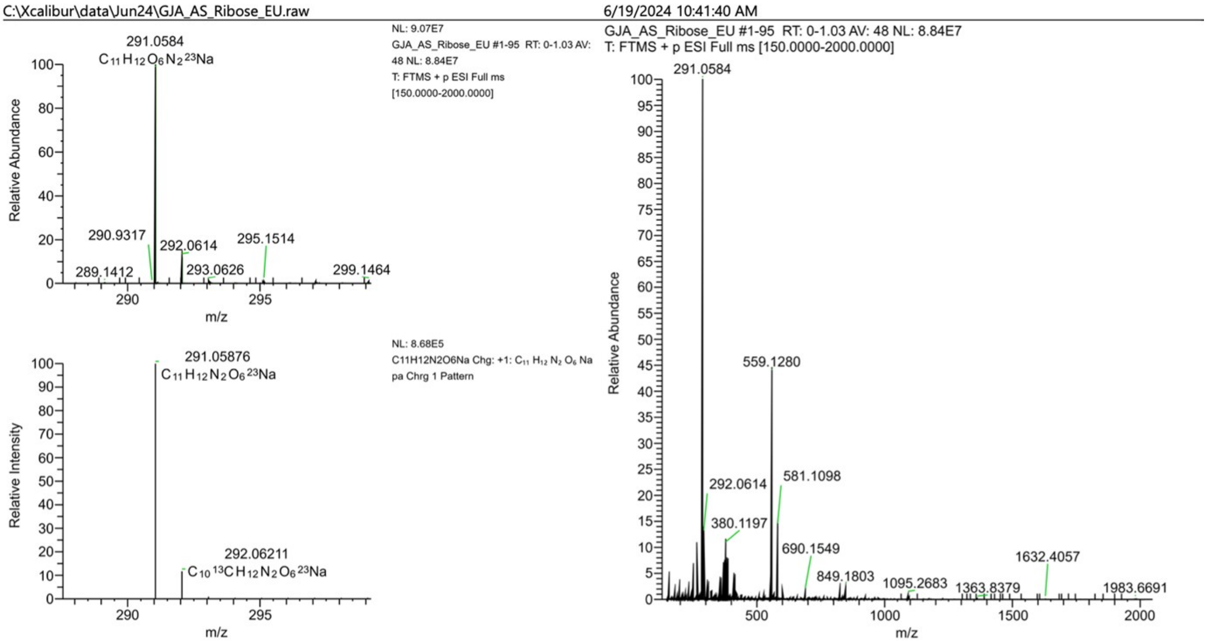

Peak Mass	Display For...	S Fit	RDB	Delta [ppm]	Theo. mass	Rank	Combined S...	# Matched I...	# Missed Iso.	MS Cov. [%]	Pattern Cov....	MSMS Matc...
291.0584	$C_{11}H_{12}O_6N_2{}^{23}Na$	39.2606878445992	6.50	-1.40	291.05876	1	94.97	4	0	98.06	99.86	(Collection)
291.0584	$C_9H_{10}O_5N_5{}^{23}Na$	24.4213048660247	7.00	3.22	291.05741	2	94.19	4	1	98.06	98.21	(Collection)
291.0584	$C_{10}H_{13}O_9N$	26.993456390945	5.00	-0.45	291.05848	3	93.12	4	0	96.8	97.93	(Collection)
291.0584	$C_{10}H_6ON_9{}^{23}Na$	24.6612382800328	12.00	-1.38	291.05875	4	93	2	2	96.8	96.48	(Collection)
291.0584	$C_9H_7O_4N_8$	22.0456328691433	10.50	-0.43	291.05848	5	92.86	3	1	96.8	96.39	(Collection)

Figure S84 – HRMS trace of compound 21.

5-azidouridine (22)

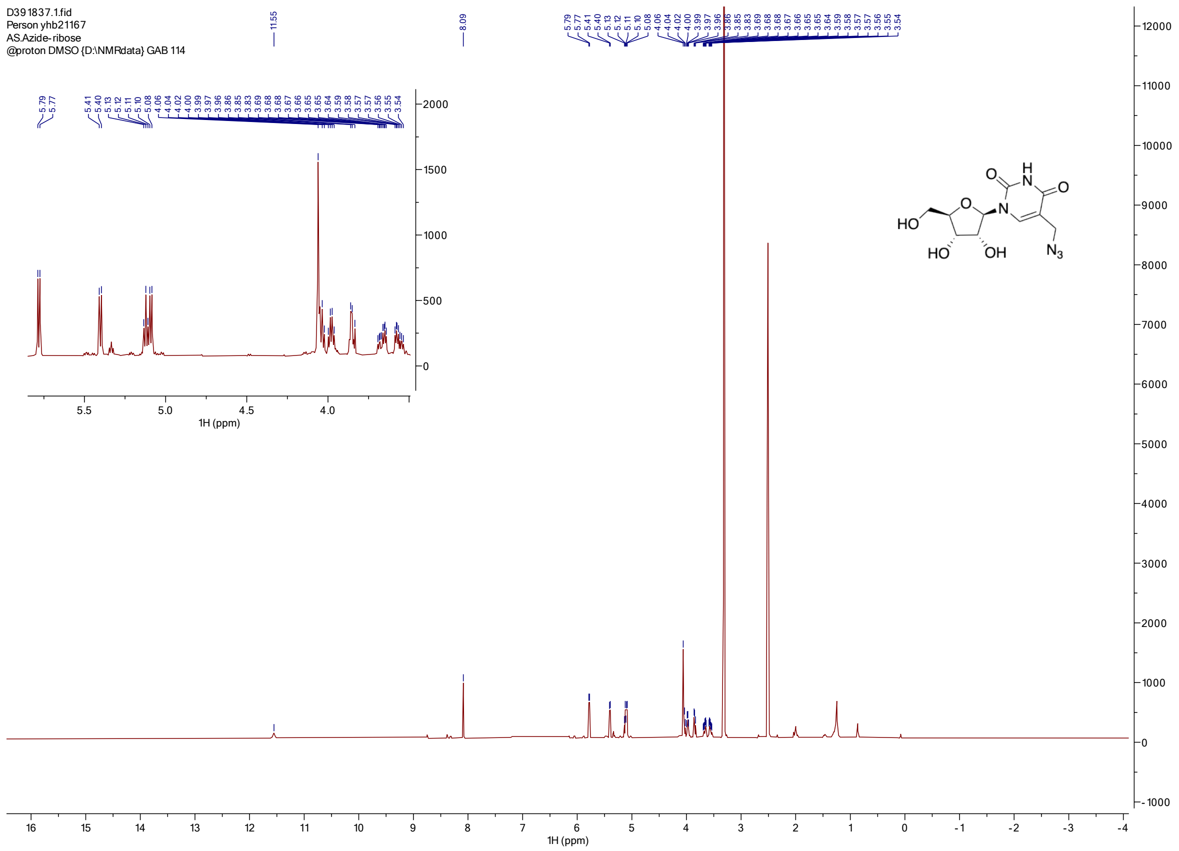

Figure S85 – 1H NMR of compound 22.

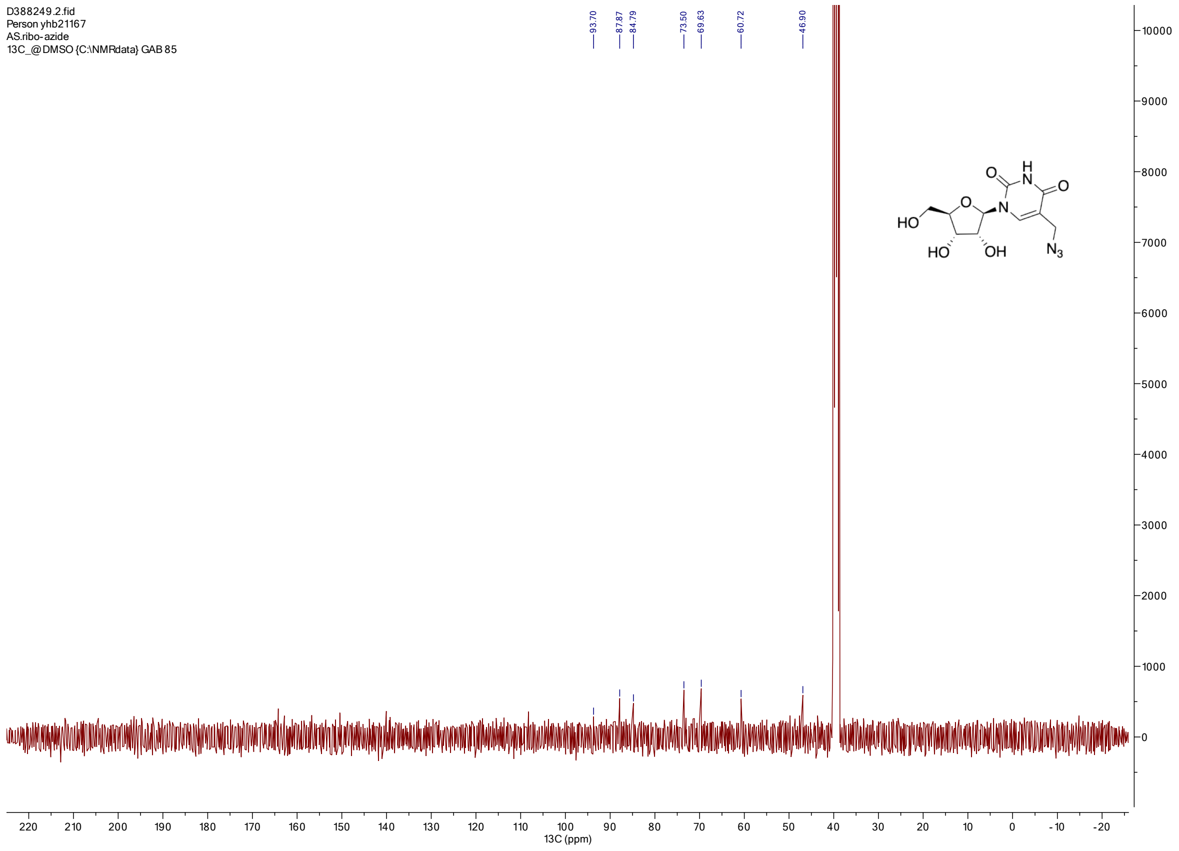

Figure S86 – 13C NMR of compound 22.

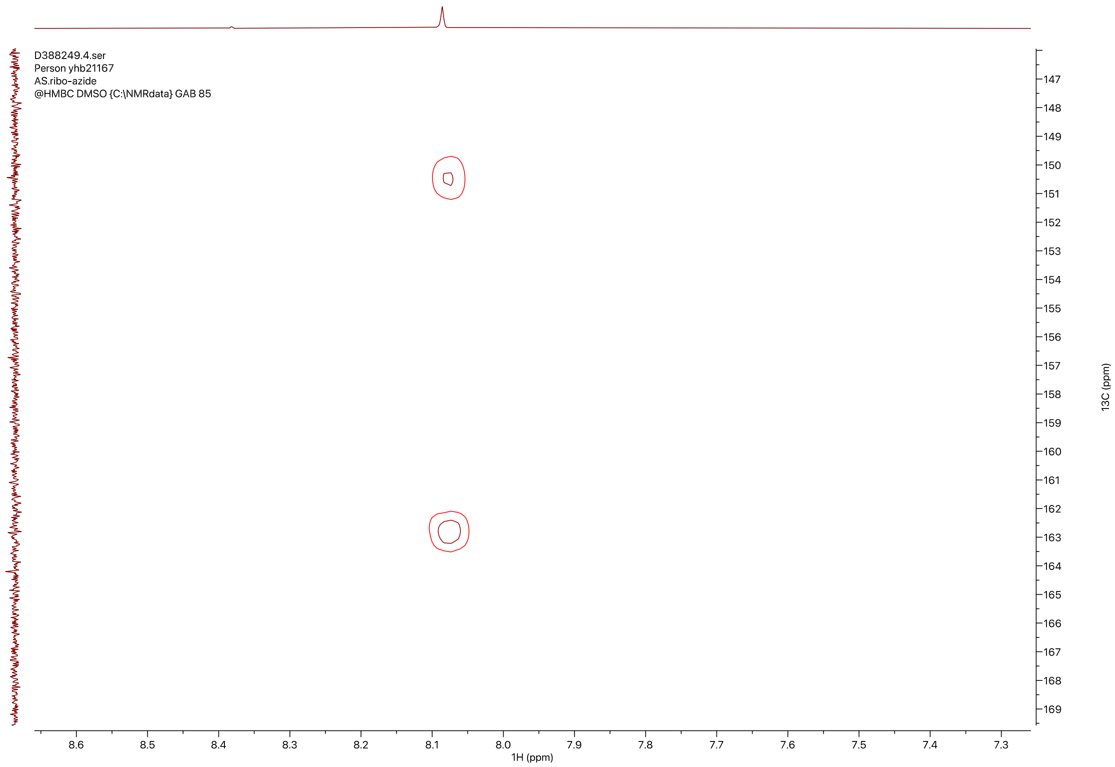

Figure S87 – HMBC of compound 22.

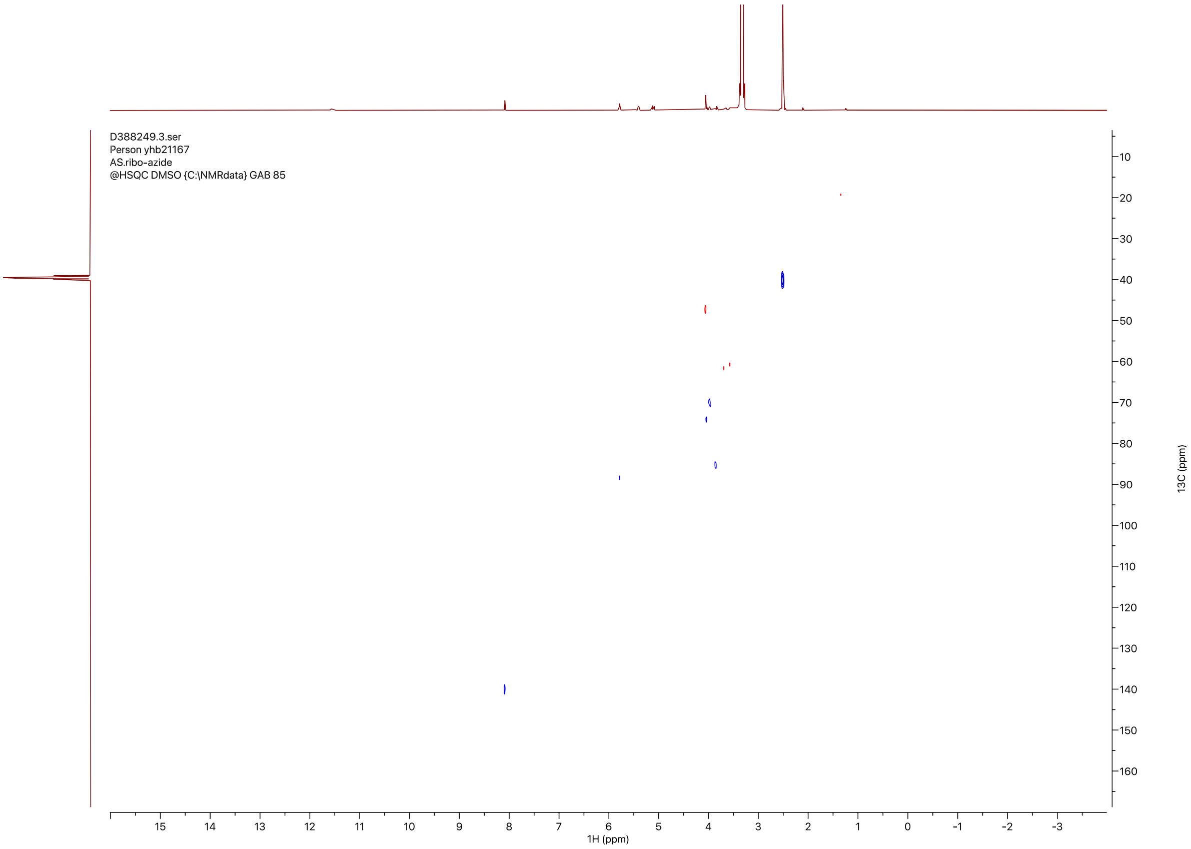

Figure S88 – HSQC of compound 22.

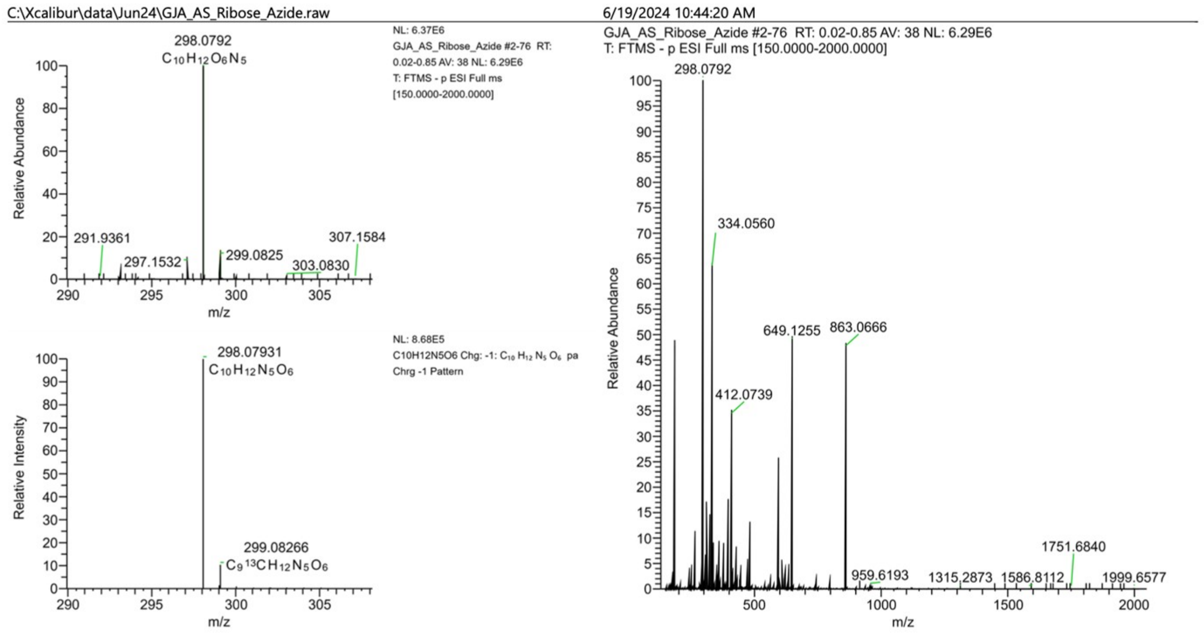

Peak Mass	Display For...	S Fit	RDB	Delta [ppm]	Theo. mass	Rank	Combined S...	# Matched I...	# Missed Iso.	MS Cov. [%]	Pattern Cov....	MSMS Matc...
298.0792	$C_{10}H_{12}O_6N_5$	47.2246124627381	7.50	-0.28	298.07931	1	95.38	4	0	98.05	99.87	(Collection)
298.0792	$C_9H_{16}O_{10}N$	18.3152523888921	2.50	4.21	298.07797	2	92.72	4	0	96.85	97.78	(Collection)
298.0792	$C_8H_{10}O_5N_8$	17.0923941122925	8.00	4.22	298.07796	3	84.68	3	1	88.44	89.46	(Collection)
298.0792	$C_{11}H_8O_2N_9$	7.06379603524497	12.50	-4.77	298.08064	4	83.01	3	1	87.23	85.93	(Collection)
298.0792	$C_{12}H_{14}O_7N_2$	7.01913931825121	7.00	-4.79	298.08065	5	83.01	4	0	87.23	86.66	(Collection)

Figure S89 – HRMS of compound 22.

Sample Name : azide
Sample ID :
Data Filename : azide_25032024_003.lcd
Method Filename : Admir Trans glyco Run.lcm
Batch Filename : azide.lcb
Vial # : 1-93
Injection Volume : 10 uL
Date Acquired : 25/03/2024 10:47:35
Date Processed : 25/03/2024 10:58:55

Sample Type : Unknown
Acquired by : Shimadzu
Processed by : Shimadzu

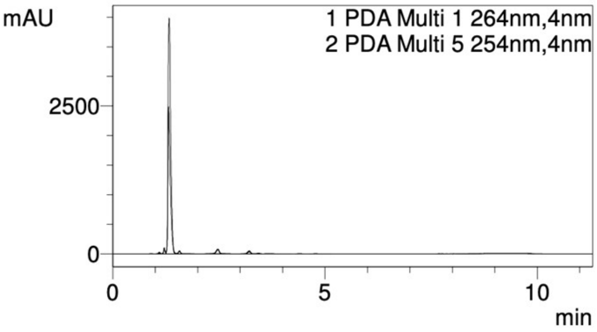

PDA Ch1 264nm

Peak#	Ret. Time	Area	Height	ID#
1	0.885	7941	4185	
2	1.095	78762	29834	
3	1.215	232118	98246	
4	1.330	17453054	3947693	
5	1.573	175743	49390	
6	1.770	20912	3754	
7	1.896	4873	545	
8	2.475	435008	78420	
9	2.722	6794	883	
10	2.880	1560	310	
11	3.220	248620	50960	
12	3.434	43892	9431	
13	3.631	5511	1282	
14	3.734	16456	3254	
15	4.017	1054	272	
16	4.151	2042	505	
17	4.407	11949	2673	
18	4.787	8570	1913	
19	5.040	1458	269	
20	7.682	2684	1140	
21	7.758	2312	1061	
22	8.134	1305	484	
23	8.241	1245	318	
24	8.409	3197	1155	
25	8.649	2143	294	
26	9.840	3937	1170	
Total		18773142	4289441	

Figure S90 – HPLC trace forming compound 22.

6-Bromo-deazapurine-2'-arabino-fluoro-ribose (29)

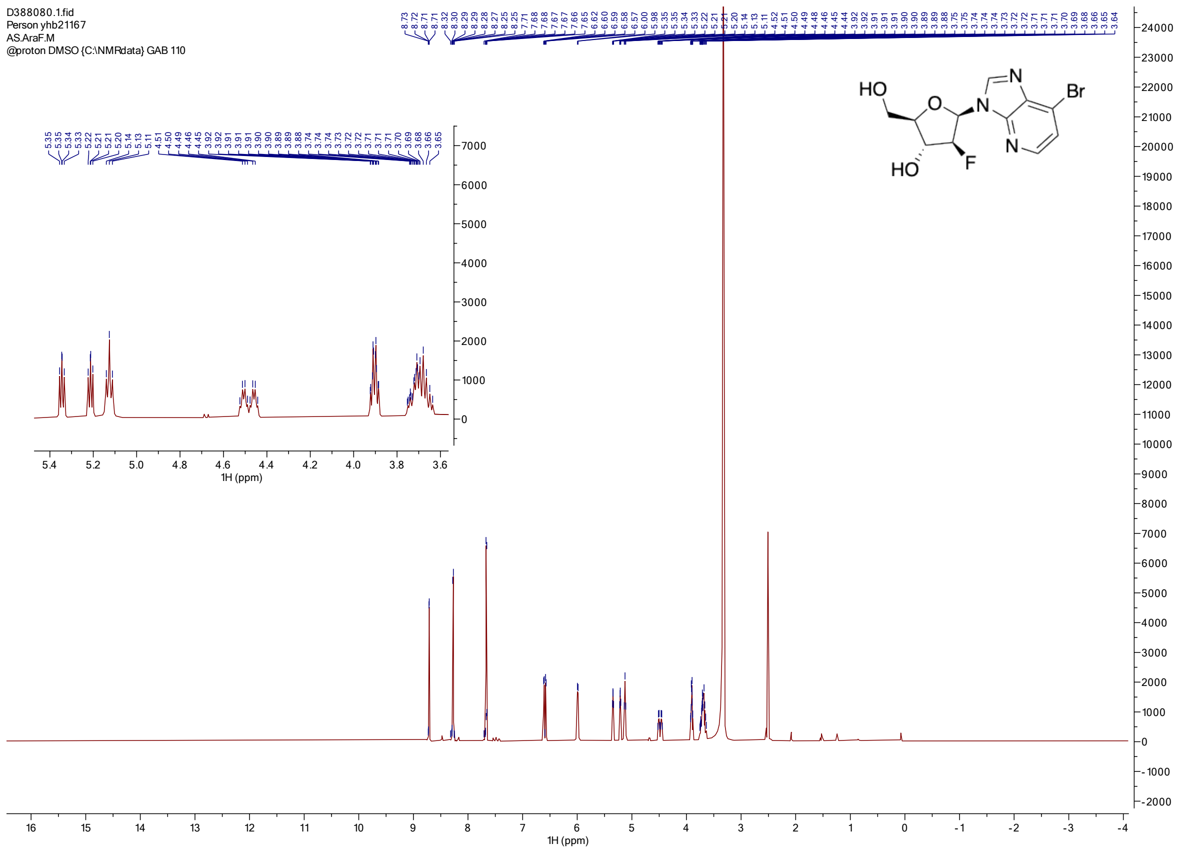

Figure S91 – 1H NMR of compound 29.

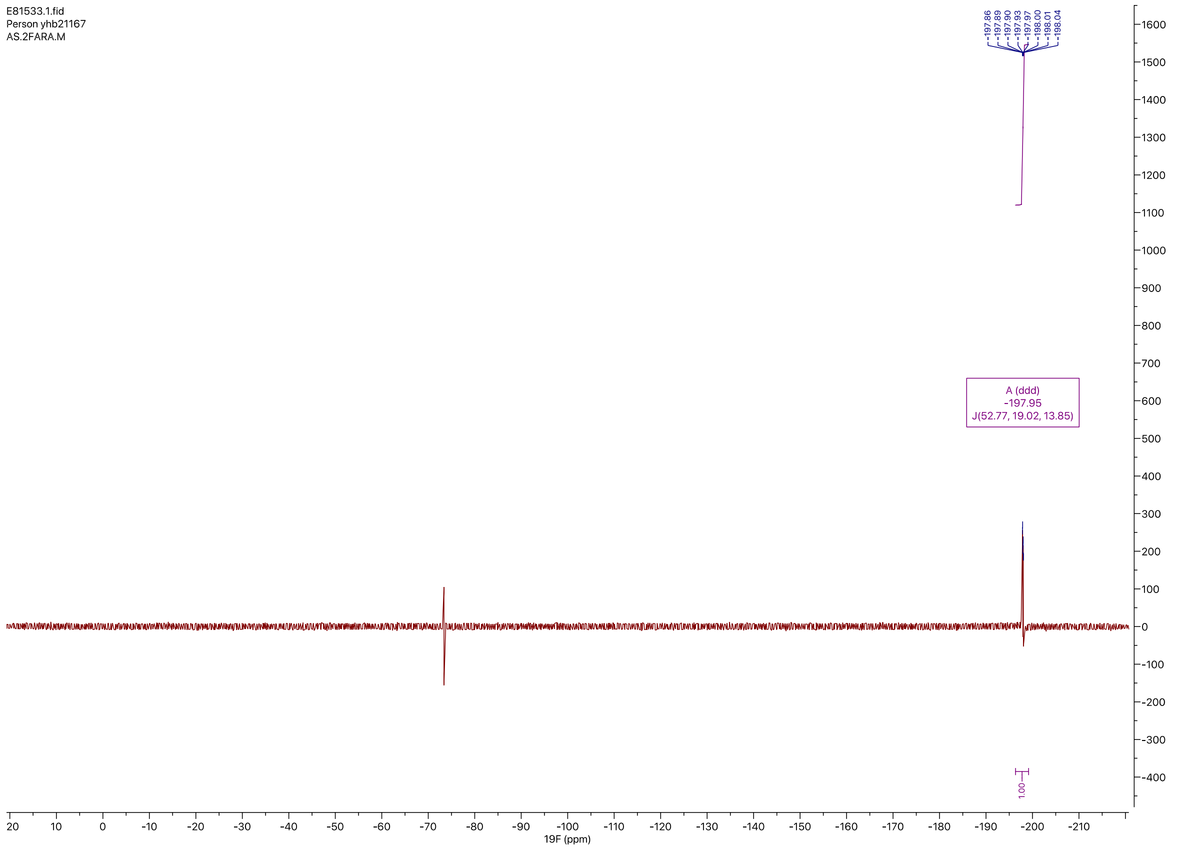

Figure S92 – 19F NMR of compound 29 (-73.4 ppm corresponds to TFA).

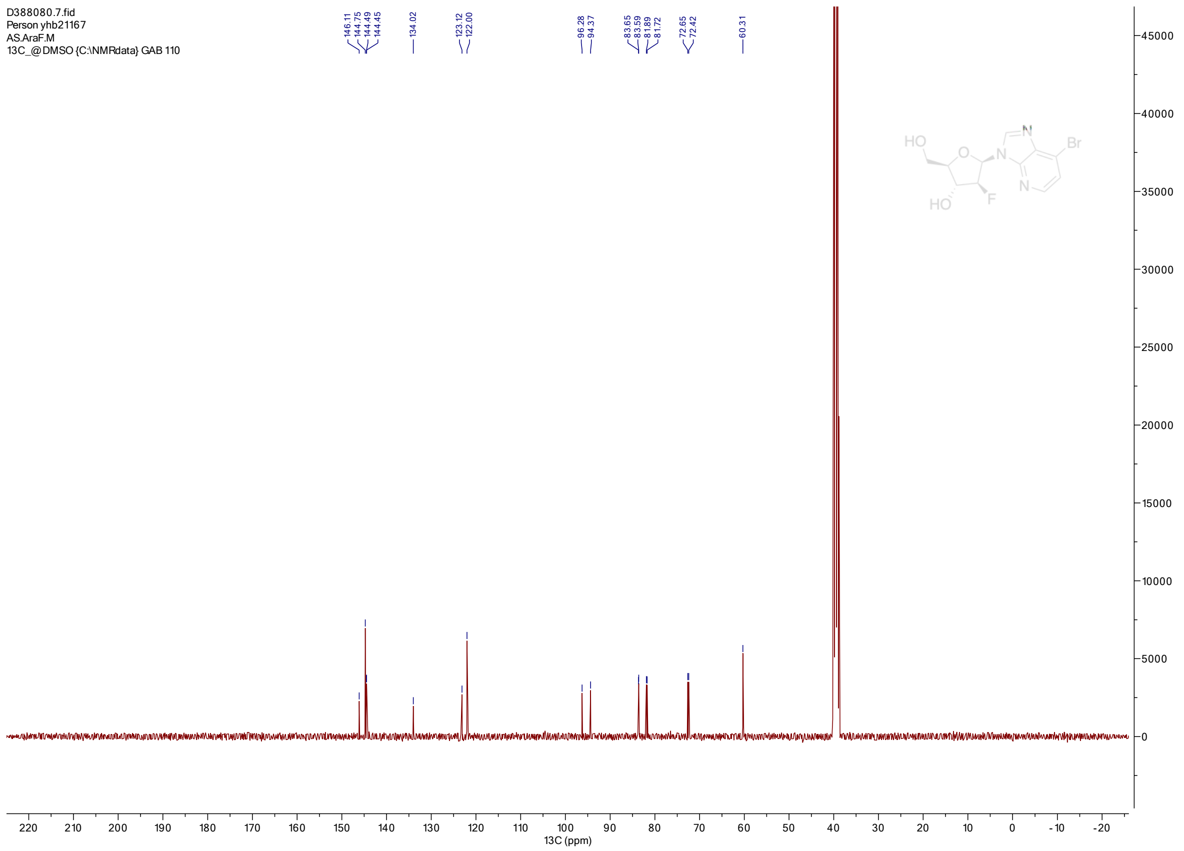

Figure S93 – 13C NMR of compound 29.

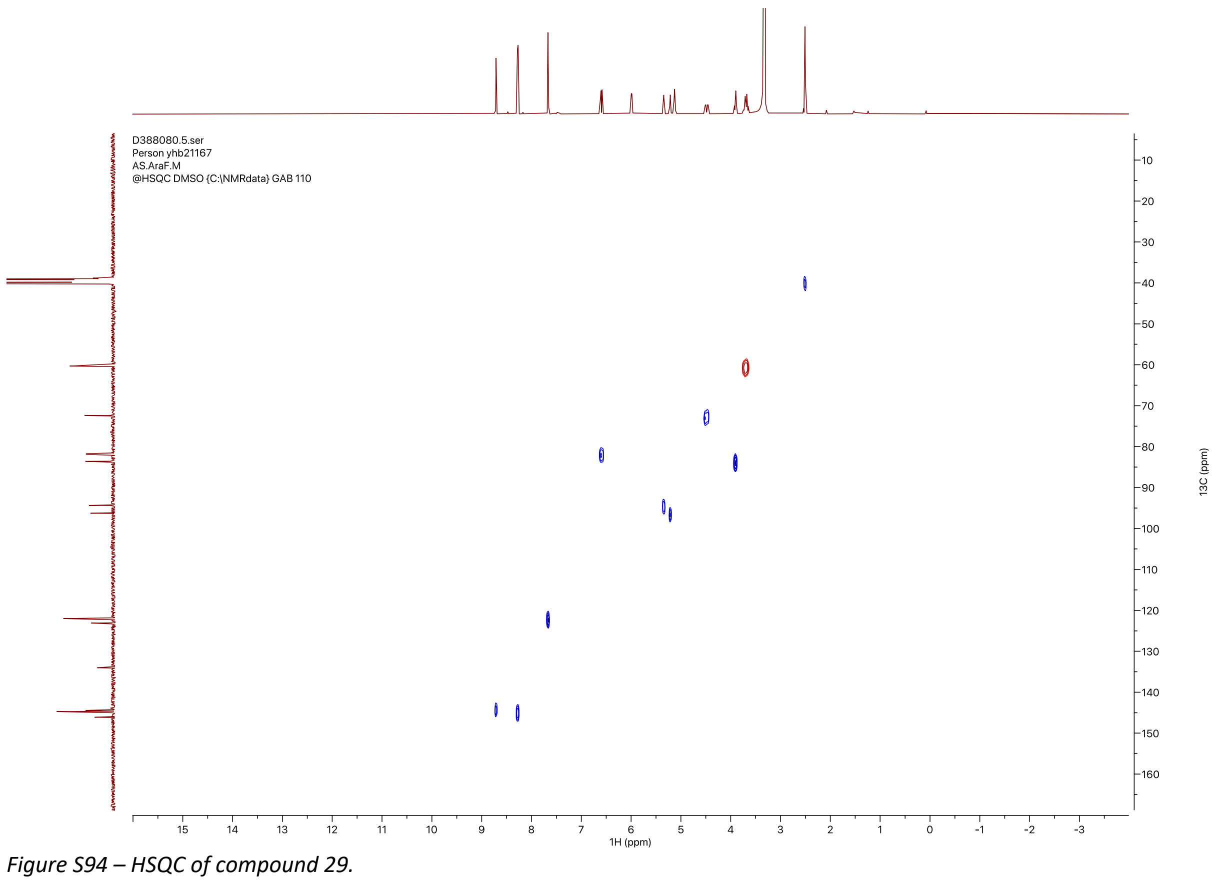

Figure S94 – HSQC of compound 29.

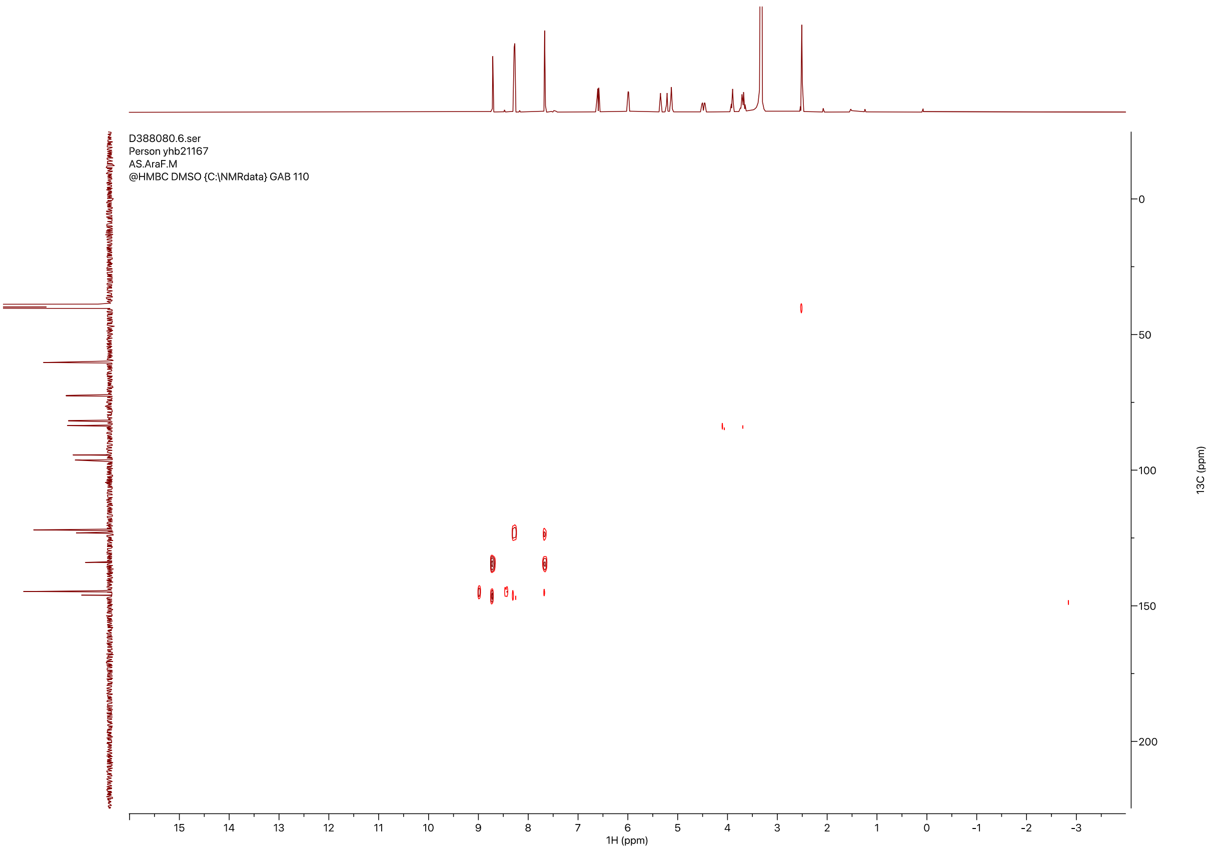

Figure S95 – HMBC of compound 29.

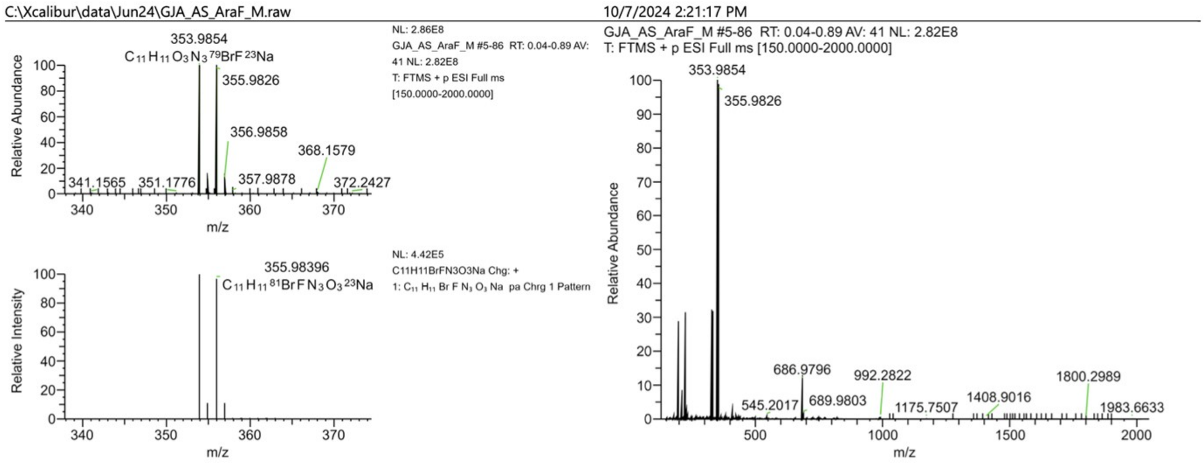

Peak Mass	Display For...	S Fit	RDB	Delta [ppm]	Theo. mass	Rank	Combined S...	# Matched I...	# Missed Iso.	MS Cov. [%]	Pattern Cov....	MSMS Matc...
353.9854	$C_{11}H_{11}O_3N_3{}^{79}BrF^{23}Na$	50.95462067 68232	6.50	-1.81	353.98600	1	96.38	5	0	98.9	100	(Collection)
353.9854	$C_{12}H_5N_9{}^{79}Br$	49.47691577 55966	14.50	2.21	353.98458	2	95.85	5	0	98.42	99.65	(Collection)
353.9854	$C_9H_9O_2N_6{}^{79}BrF^{23}Na$	43.60830658 31975	7.00	1.98	353.98466	3	95.54	5	0	98.42	99.65	(Collection)
353.9854	$C_9H_6ON_9{}^{79}BrF$	38.99187006 97081	10.50	-1.02	353.98572	4	95.29	5	0	98.42	99.73	(Collection)
353.9854	$C_{13}H_{11}O_5N_2{}^{79}Br$	35.95695955 09515	9.00	2.19	353.98459	5	95.13	6	0	98.42	99.17	(Collection)
353.9854	$C_{10}H_{12}O_6N_2{}^{79}BrF$	34.63553628 94705	5.00	-1.04	353.98573	6	95.06	6	0	98.42	99.21	(Collection)
353.9854	$C_{12}O_7N_7$	28.19167948 82148	16.50	-0.03	353.98537	7	48.62	2	2	49.75	98	(Collection)
353.9854	$C_{13}H_6O_{12}$	26.20851179 0464	11.00	-0.04	353.98538	8	48.51	2	2	49.75	97.01	(Collection)
353.9854	$C_9H_4O_9N_4F^{23}Na$	26.08870716 07498	9.00	-0.26	353.98545	9	48.51	2	2	49.75	97.88	(Collection)
353.9854	$C_{12}H_3O_8N_4{}^{23}Na$	22.99406960 58846	13.00	2.97	353.98431	10	48.34	2	2	49.75	97.84	(Collection)

Figure S96 – HRMS of compound 29.

**Sample name:**	f-ara-M		
**Description:**			
**Sample amount:**	0.000	**Sample type:**	Sample
**Instrument:**	Shimadzu LC	**Location:**	1:2
**Injection date:**	2024-06-07 11:30:49+01:00	**Injection:**	1 of 1
**Acq. method:**	Transglycosylation run-1.5ml-min.amx	**Injection volume:**	10.000 µL

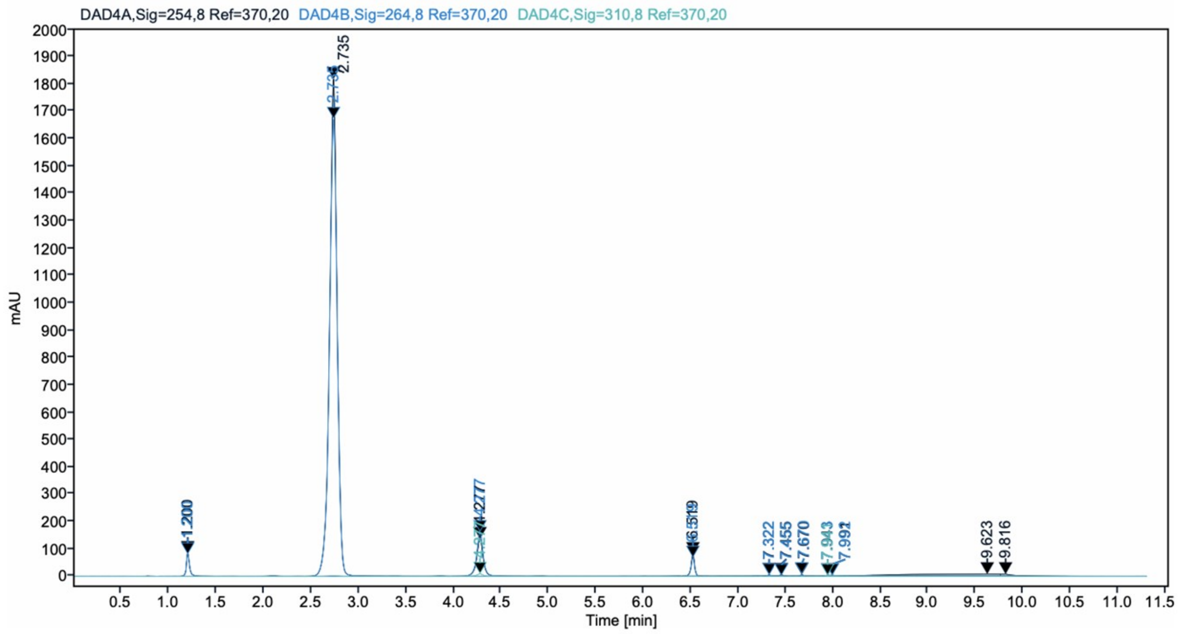

Figure S97 – HPLC trace of the reaction forming compound 29.

6-Bromo-deazapurine-2'-fluoro-ribose (27)

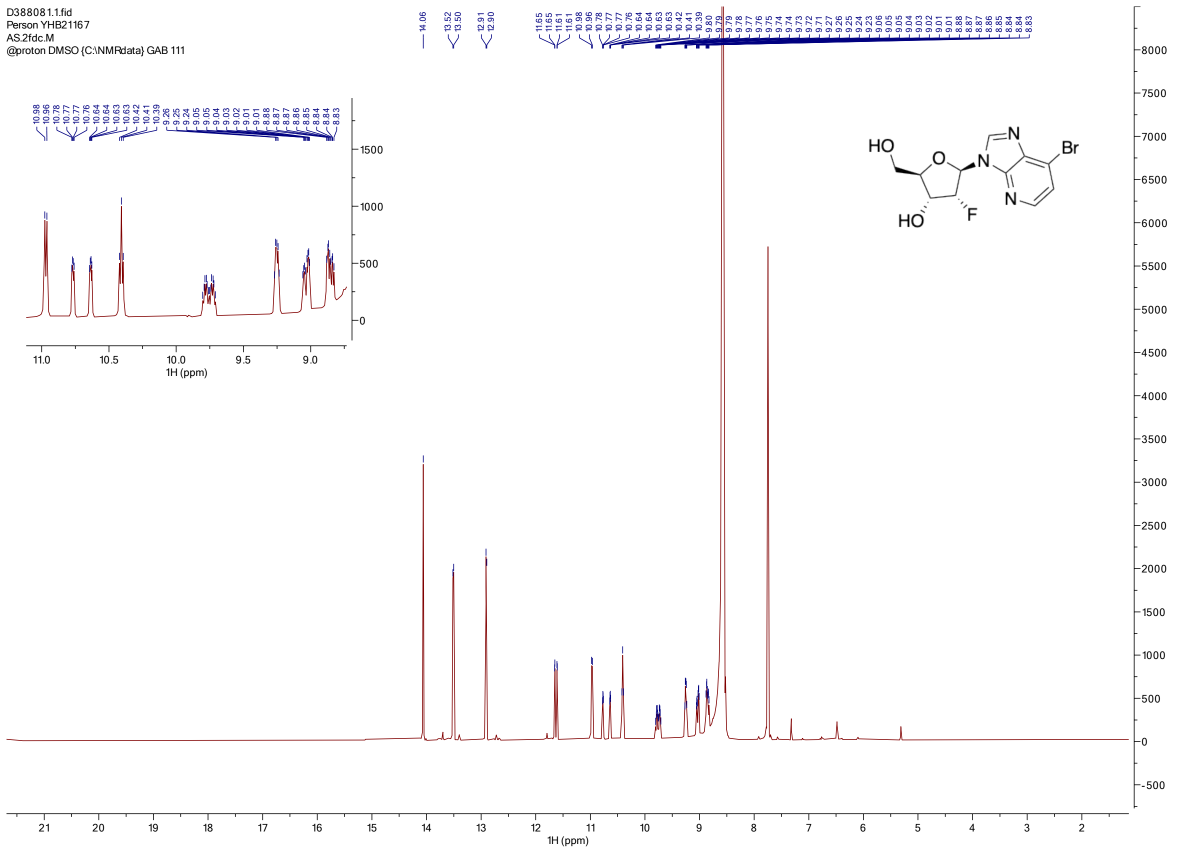

Figure S98 – 1H NMR of compound 27.

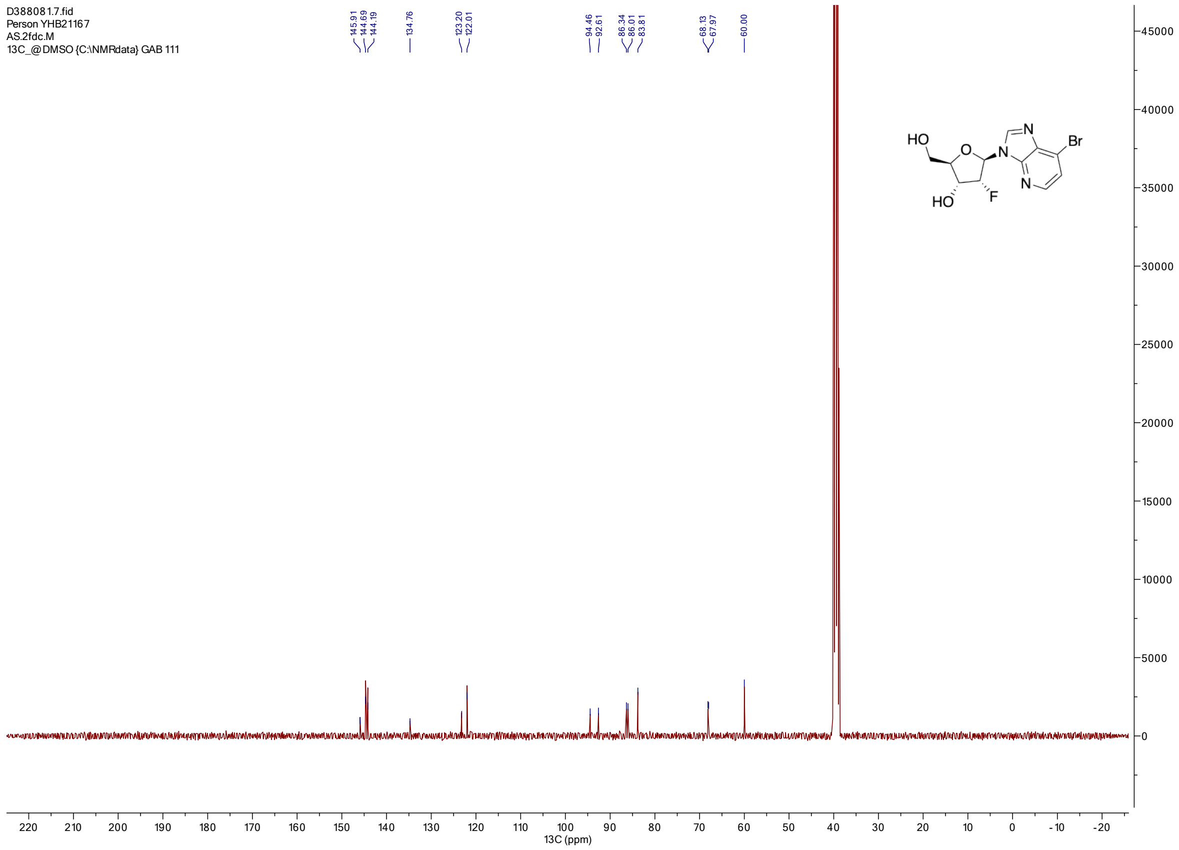

Figure S99 – 13C NMR of compound 27.

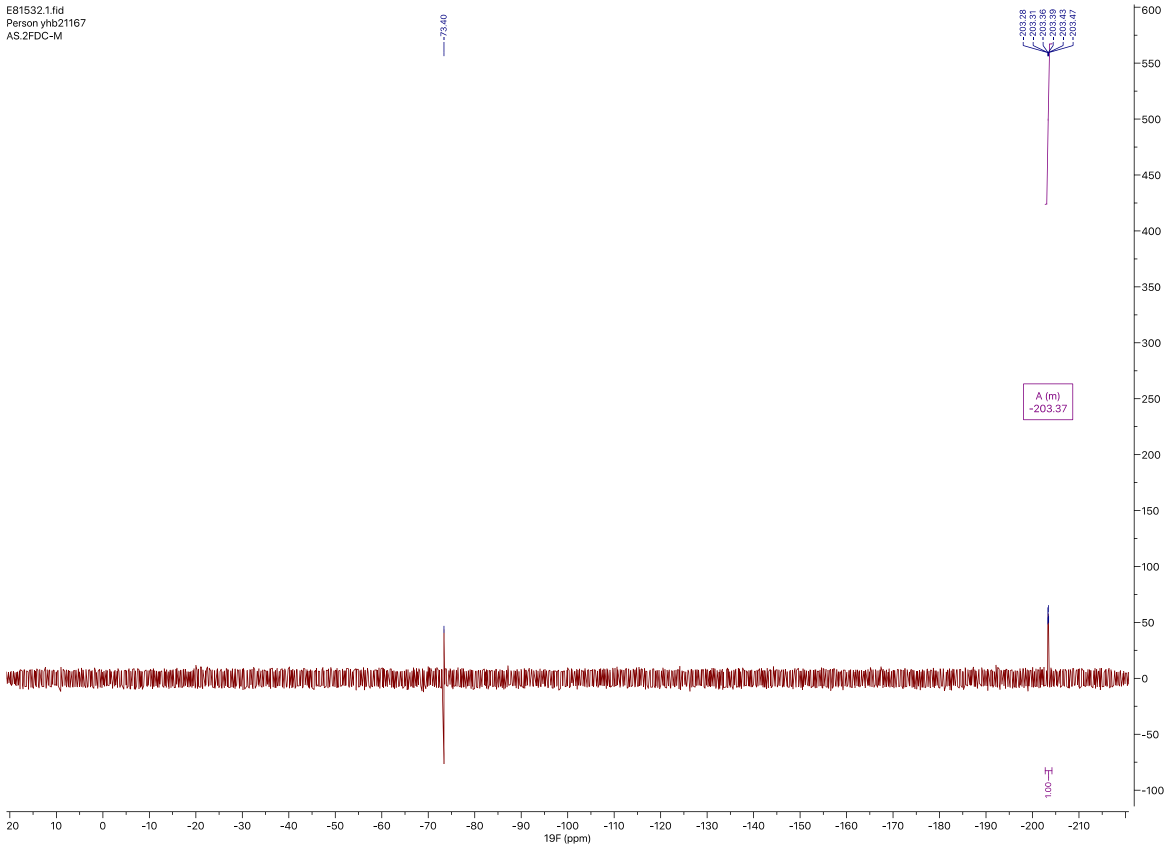

Figure S100 – 19F NMR of compound 27 (-73.4 ppm corresponds to TFA).

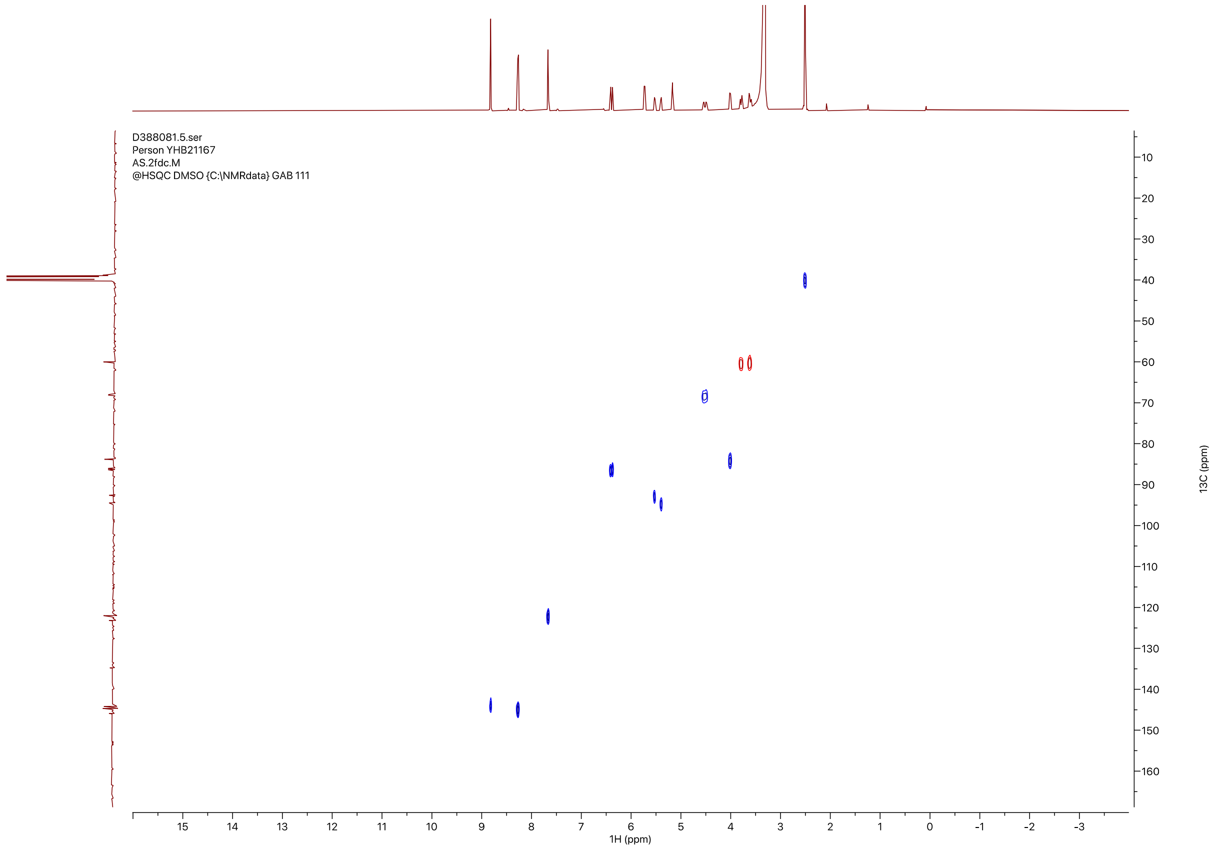

Figure S101 – HSQC of compound 27.

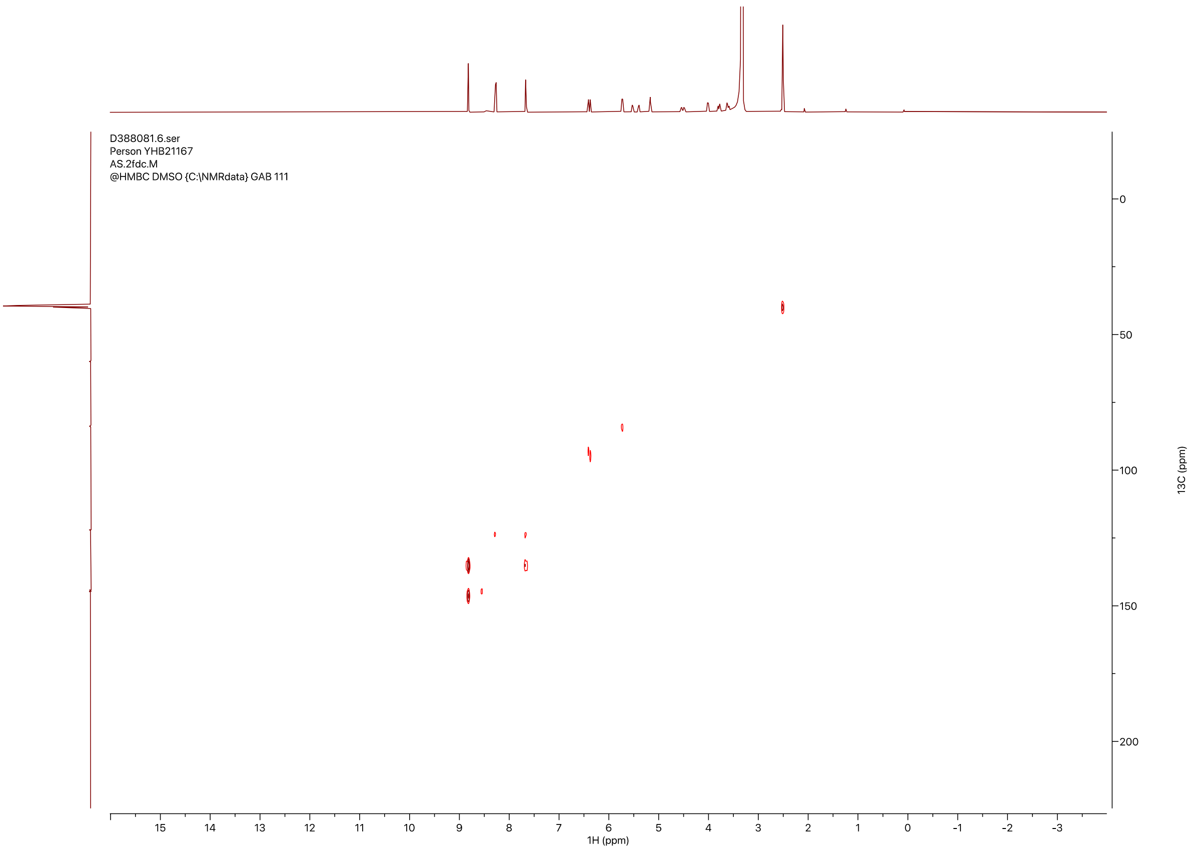

Figure S102 – HMBC of compound 27.

[illegible]

**Sample name:**	2fdc-m		
**Description:**			
**Sample amount:**	0.000	**Sample type:**	Sample
**Instrument:**	Shimadzu LC	**Location:**	1:16
**Injection date:**	2024-06-07 12:42:01+01:00	**Injection:**	1 of 1
**Acq. method:**	Transglycosylation run-1.5ml-min.amx	**Injection volume:**	10.000 µL

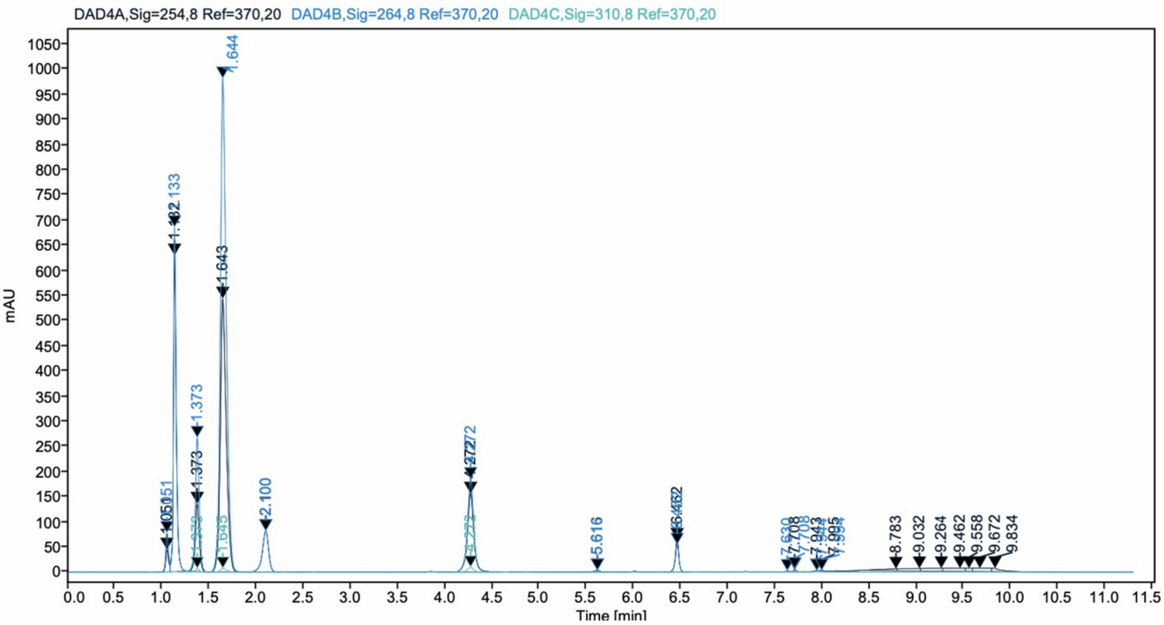

Figure S103 – HPLC trace of the reaction forming compound 27.

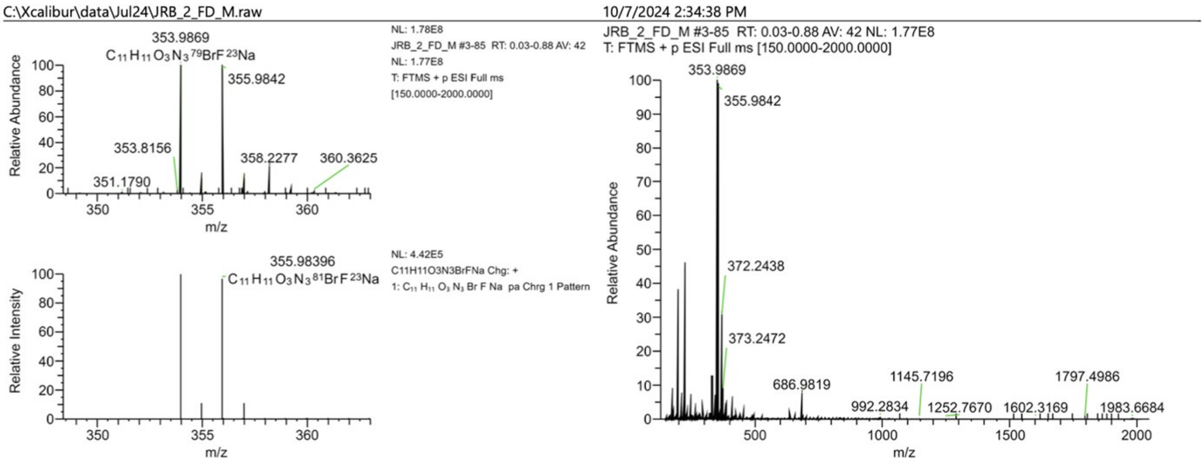

Peak Mass	Display For...	S Fit	RDB	Delta [ppm]	Theo. mass	Rank	Combined S...	# Matched I...	# Missed Iso.	MS Cov. [%]	Pattern Cov....	MSMS Matc...
353.9869	$C_{11}H_{11}O_3N_3{}^{79}BrF^{23}Na$	76.3594869620547	6.50	2.57	353.98600	1	89.39	5	0	90.11	100	(Collection)
353.9869	$C_{11}H_8O_2N_6{}^{79}BrF$	67.9289069944871	10.00	-0.43	353.98707	2	88.95	5	0	90.11	100	(Collection)
353.9869	$C_9H_6ON_9{}^{79}BrF$	36.8926155398265	10.50	3.36	353.98572	3	86.91	5	0	89.69	99.73	(Collection)
353.9869	$C_{10}H_{12}O_6N_2{}^{79}BrF$	34.9048293514297	5.00	3.35	353.98573	4	86.8	6	0	89.69	99.21	(Collection)
353.9869	$C_{13}H_{13}O_4{}^{79}BrF^{23}Na$	31.7359313038421	6.00	-1.22	353.98735	5	86.64	6	0	89.69	99.27	(Collection)
353.9869	$C_{11}H_6O_{10}NF^{23}Na$	29.1942416242034	8.50	0.34	353.98679	6	44.45	2	2	45.3	97.57	(Collection)
353.9869	$C_{10}O_5N_8F^{23}Na$	28.6063498418542	14.00	0.35	353.98679	7	44.42	2	2	45.3	98.56	(Collection)
353.9869	$C_{10}H_7O_{13}F$	28.3002579220626	7.00	1.11	353.98652	8	44.4	2	2	45.3	97.07	(Collection)
353.9869	$C_9HO_8N_7F$	25.2586831105188	12.50	1.13	353.98651	9	44.24	2	2	45.3	98.05	(Collection)
353.9869	$C_{11}H_3O_9N_4F$	24.0236256767394	12.00	-2.67	353.98786	10	44.18	2	2	45.3	97.74	(Collection)

Figure S104 – HRMS trace of compound 27.

2-chloro-adenosine-2'-fluoro-ribose (28)

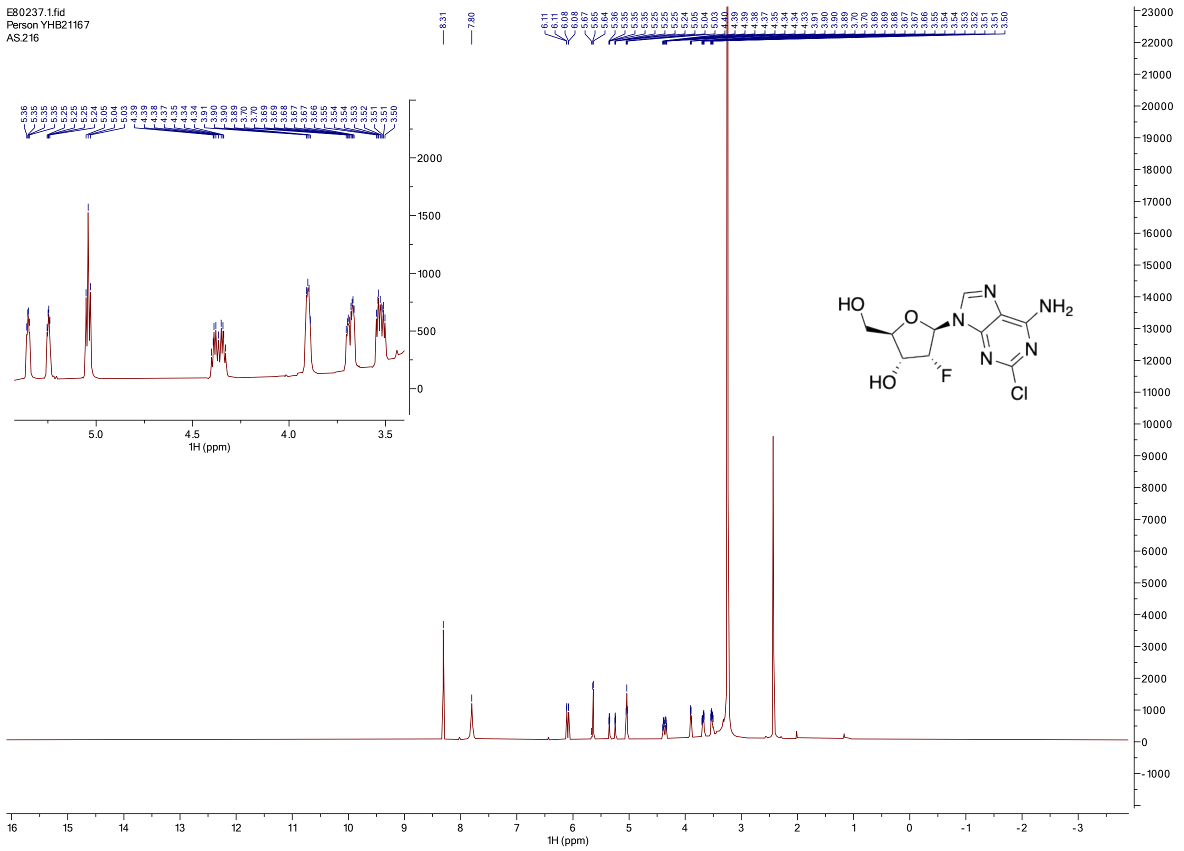

Figure S105 - 1H NMR of compound 28.

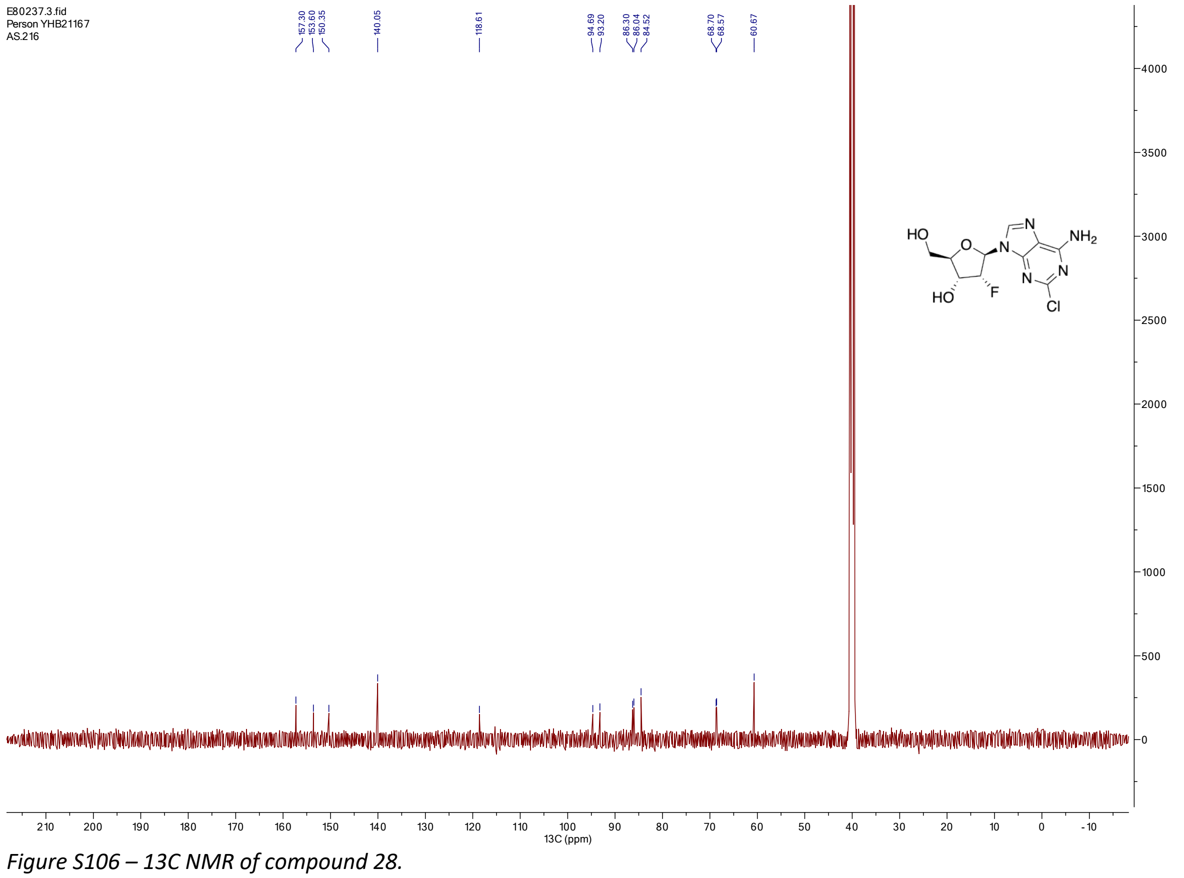

Figure S106 – 13C NMR of compound 28.

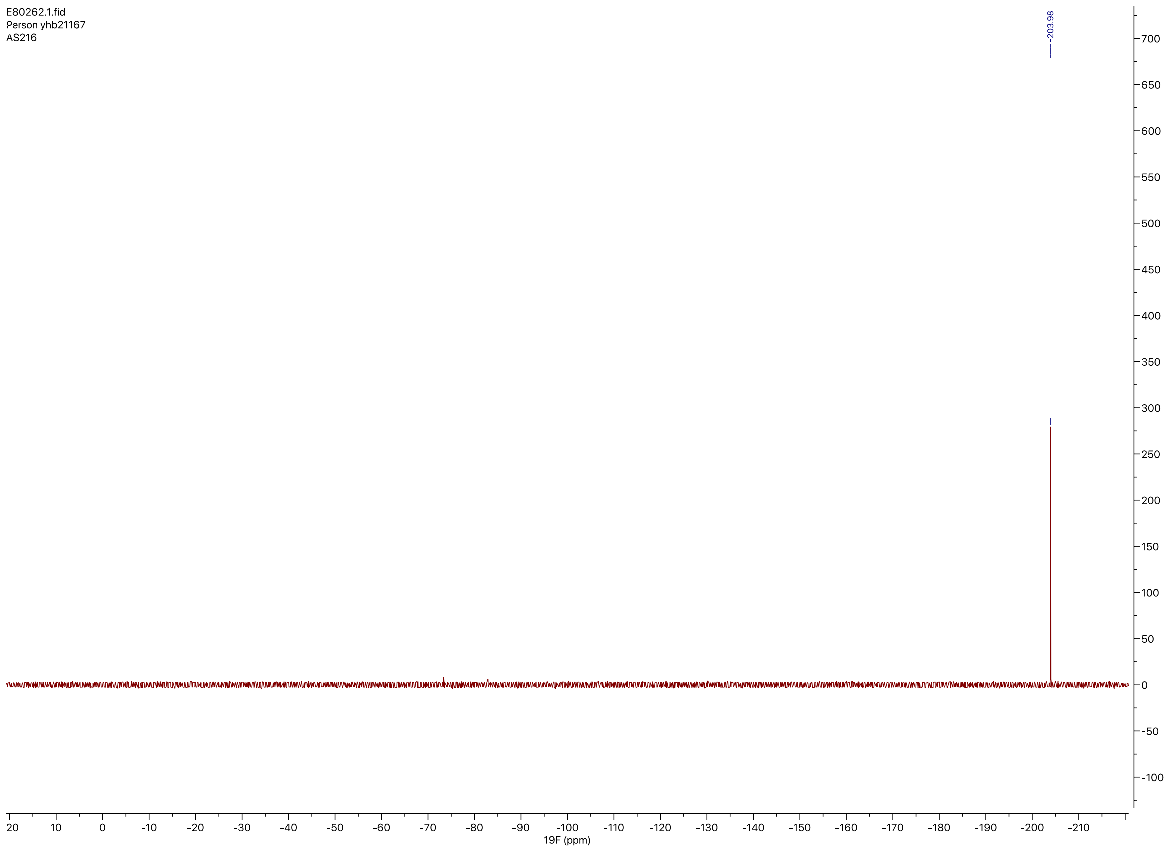

Figure S107 – 19F NMR of compound 28

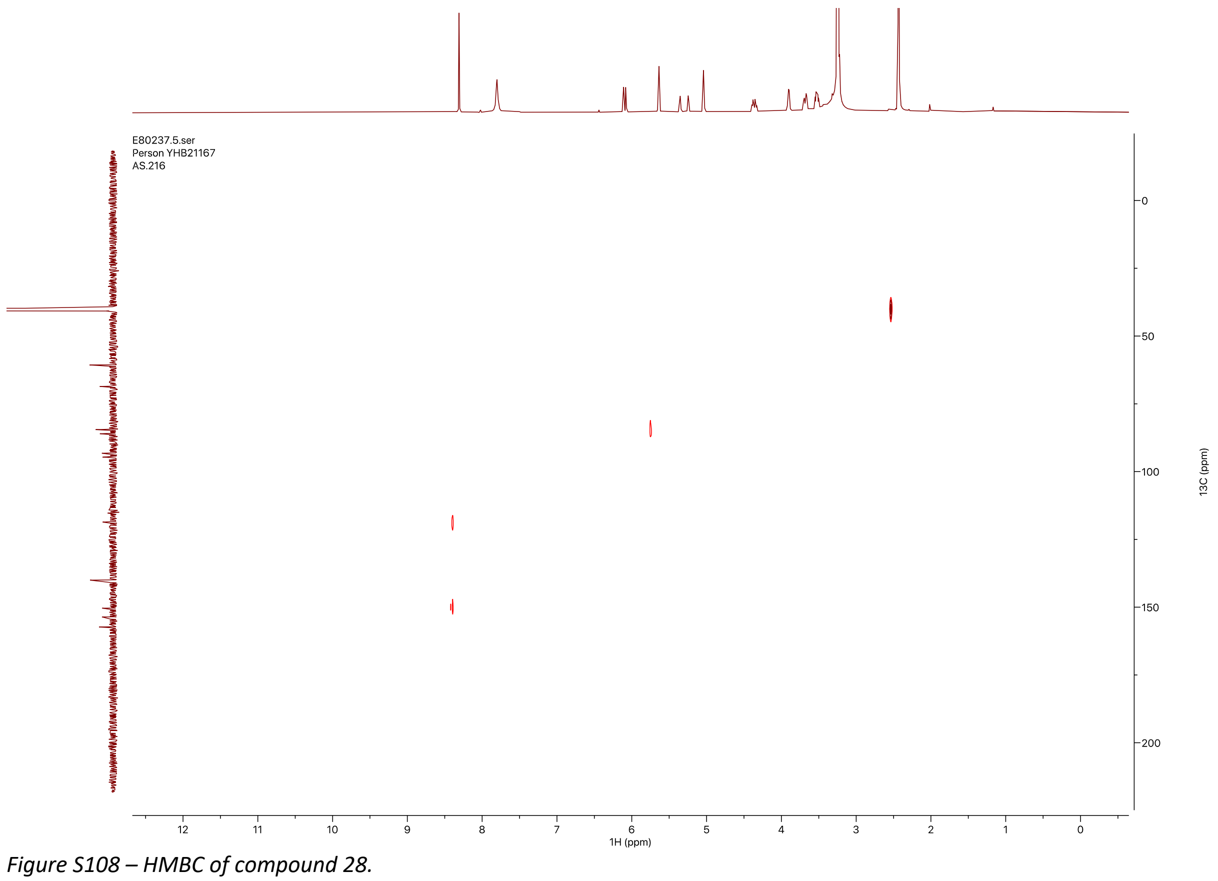

Figure S108 – HMBC of compound 28.

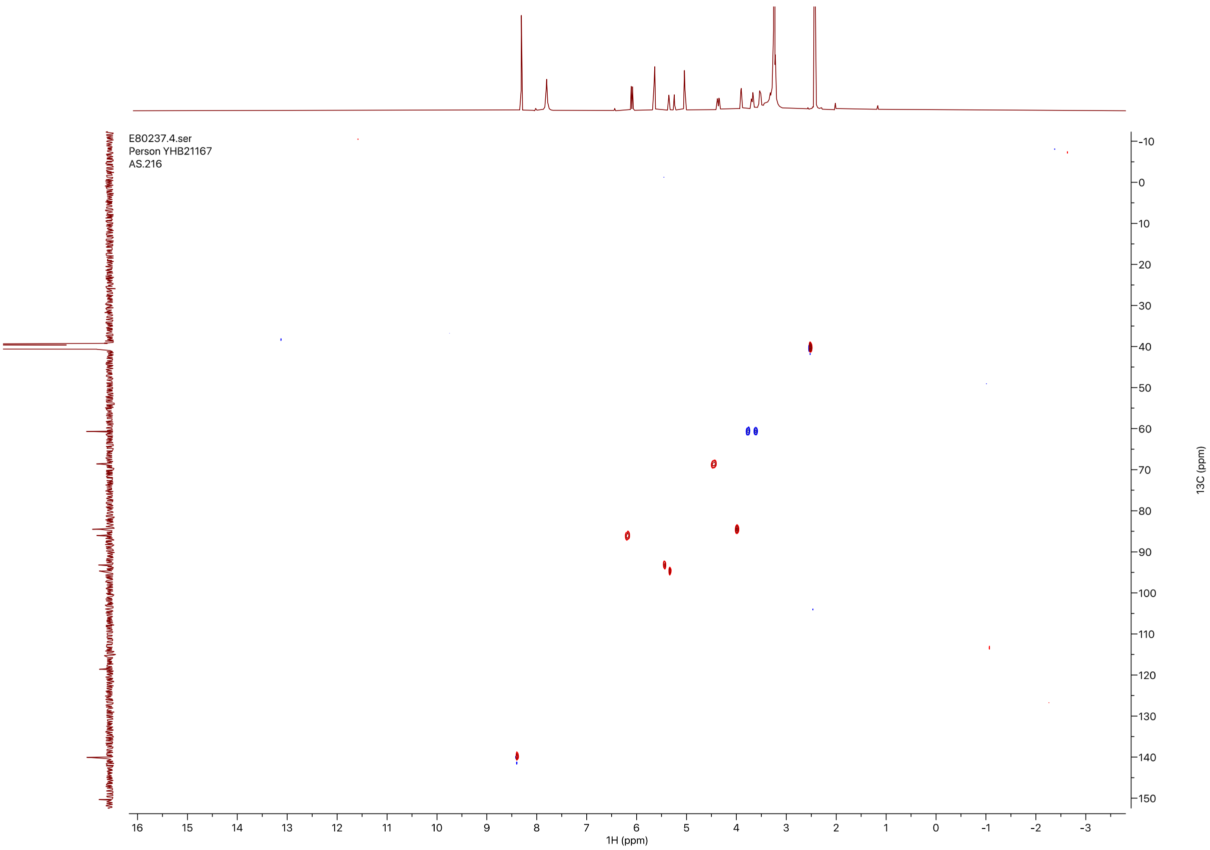

Figure S109 – HSQC NMR of compound 28.

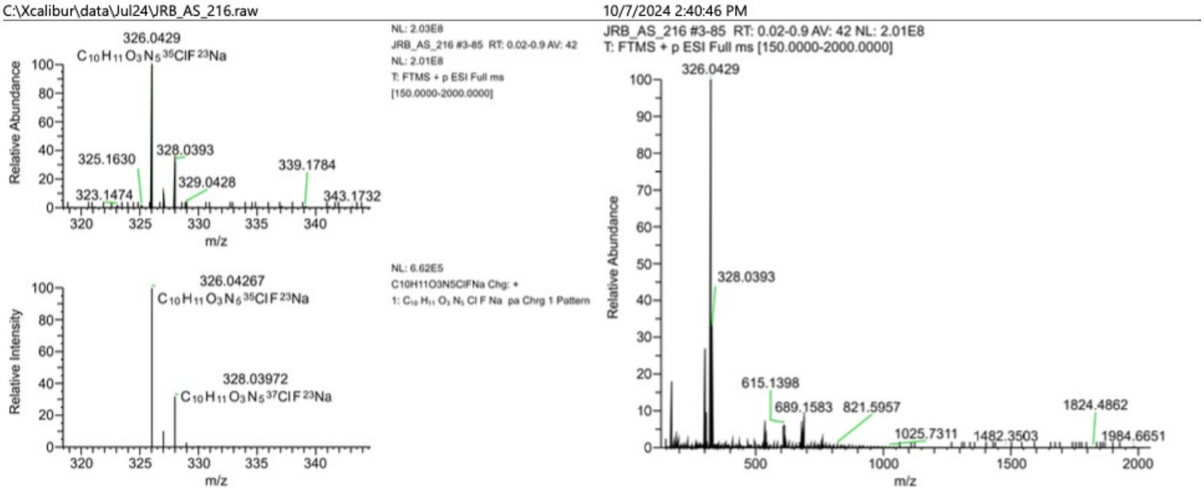

Peak Mass	Display For...	S Fit	RDB	Delta [ppm]	Theo. mass	Rank	Combined S...	# Matched I...	# Missed Iso.	MS Cov. [%]	Pattern Cov....	MSMS Matc...
326.0429	$C_{10}H_{11}O_3N_5{}^{35}ClF^{23}Na$	75.1946240346319	6.50	0.79	326.04267	1	97.49	5	0	98.73	100	(Collection)
326.0429	$C_9H_{12}O_6N_4{}^{35}ClF$	53.2197429044823	5.00	1.64	326.04239	2	96.16	5	0	98.54	99.67	(Collection)
326.0429	$C_{10}H_8O_2N_8{}^{35}ClF$	46.2091384016164	10.00	-2.47	326.04373	3	95.96	5	0	98.73	100	(Collection)
326.0429	$C_9H_{15}O_7N^{35}ClF^{23}Na$	33.6568786120133	1.50	4.90	326.04133	4	95.13	5	0	98.54	99.63	(Collection)
326.0429	$C_{11}H_{14}O_7N^{35}ClF$	23.4152153411576	4.50	-2.48	326.04373	5	92.5	5	0	96.34	96.97	(Collection)
326.0429	$C_8H_9O_2N_8{}^{35}ClF^{23}Na$	23.1521917641615	7.00	4.91	326.04132	6	88.26	5	0	91.87	93.71	(Collection)
326.0429	$C_{11}H_3O_2N_{10}F$	27.9024344711696	15.00	3.15	326.04190	7	72.02	2	1	74.47	99.16	(Collection)
326.0429	$C_{12}H_9O_7N_3F$	10.612068438047	9.50	3.13	326.04190	8	64.79	2	2	67.8	86.59	(Collection)
326.0429	$C_{12}H_{13}O_4N_2{}^{35}ClF^{23}Na$	8.11957108702233	6.00	-3.32	326.04401	9	64.66	5	0	67.8	66.73	(Collection)

Figure S110 – HRMS of compound 28.

**Sample name:**	2FD		
**Description:**			
**Sample amount:**	0.000	**Sample type:**	Sample
**Instrument:**	Shimadzu LC	**Location:**	1:20
**Injection date:**	2024-06-25 11:07:40+01:00	**Injection:**	1 of 1
**Acq. method:**	Transglycosylation run-1.5ml-min.amx	**Injection volume:**	10.000 µL

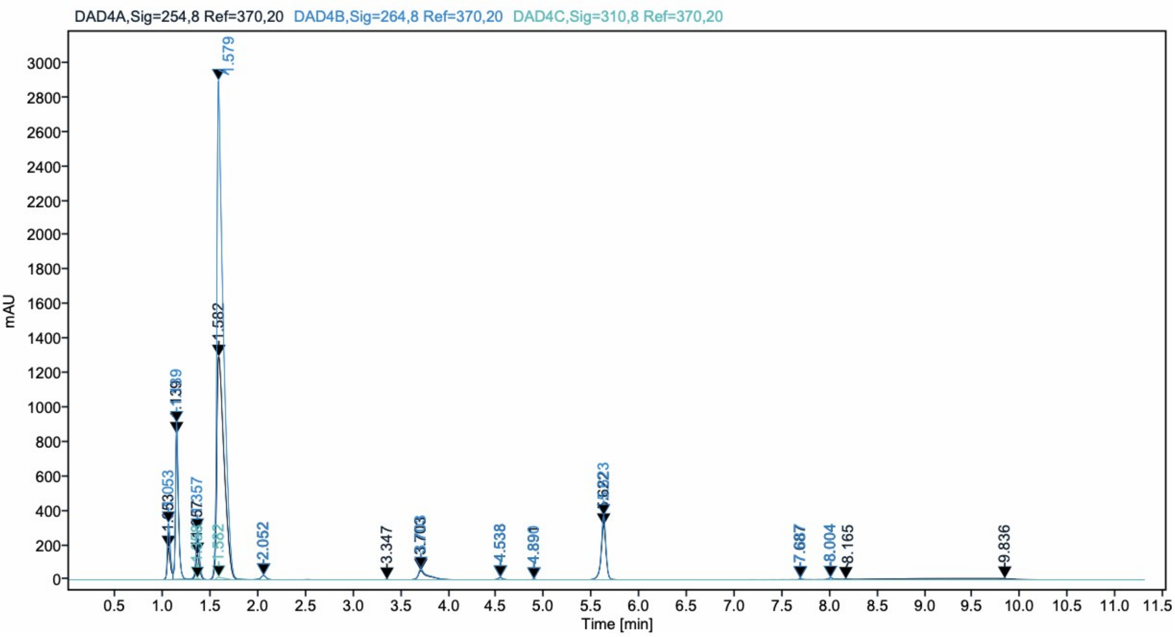

Figure S111 – HPLC trace of the reaction forming compound 28.

2-chloro-adenosine-2'-arabino-fluoro-ribose (30)

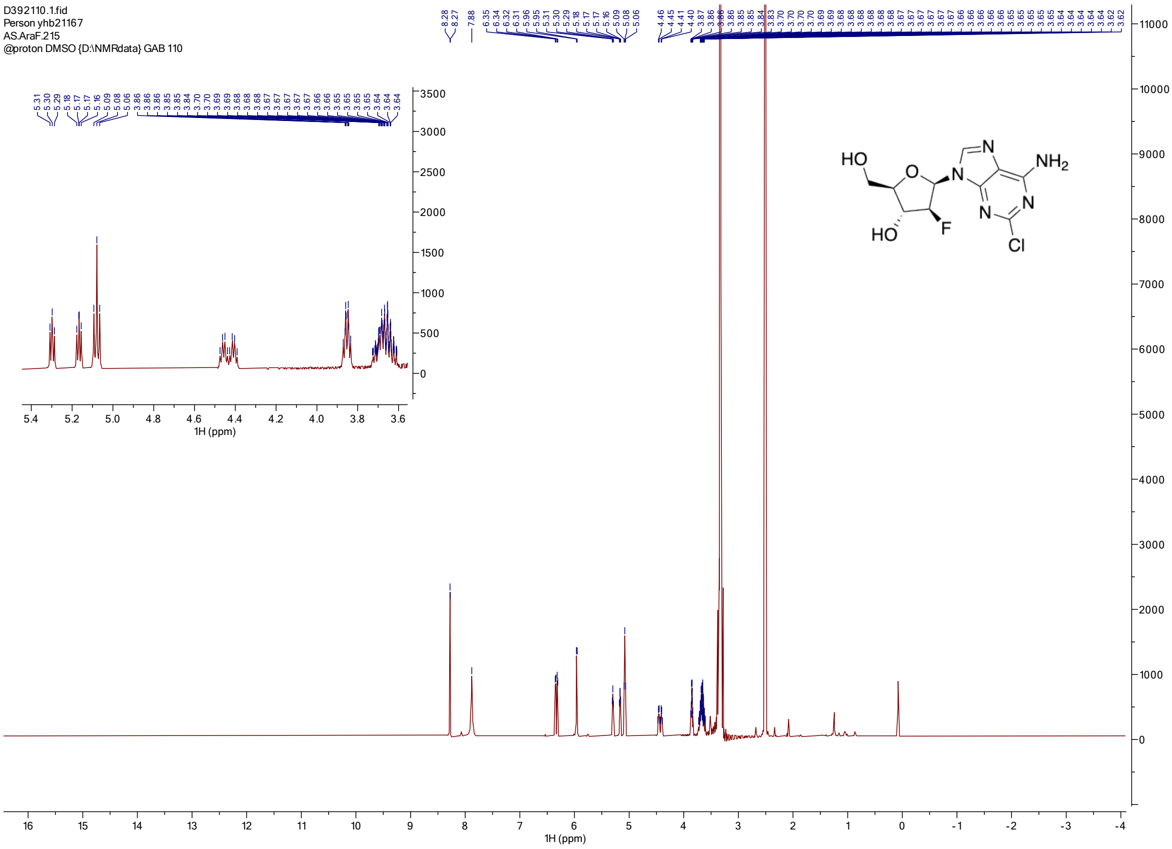

Figure S112 – 1H NMR of compound 30.

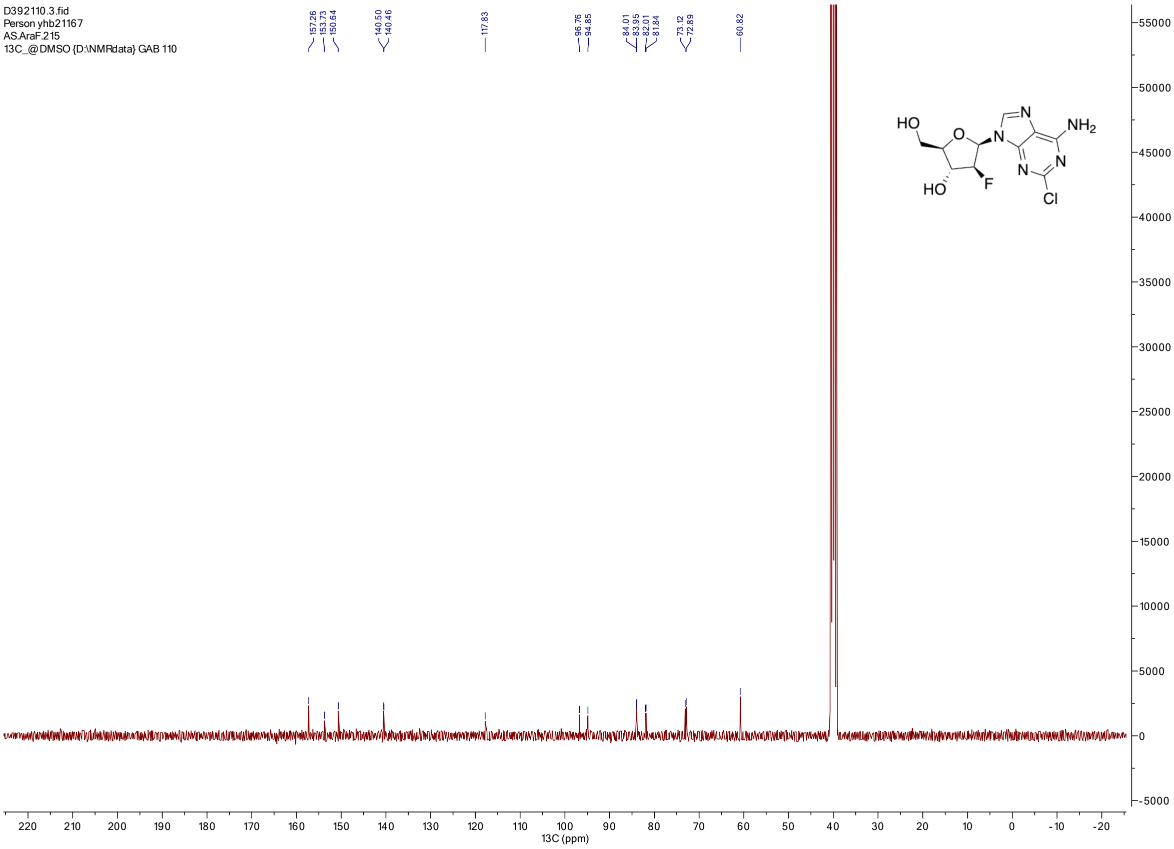

Figure S113 – 13C NMR of compound 30.

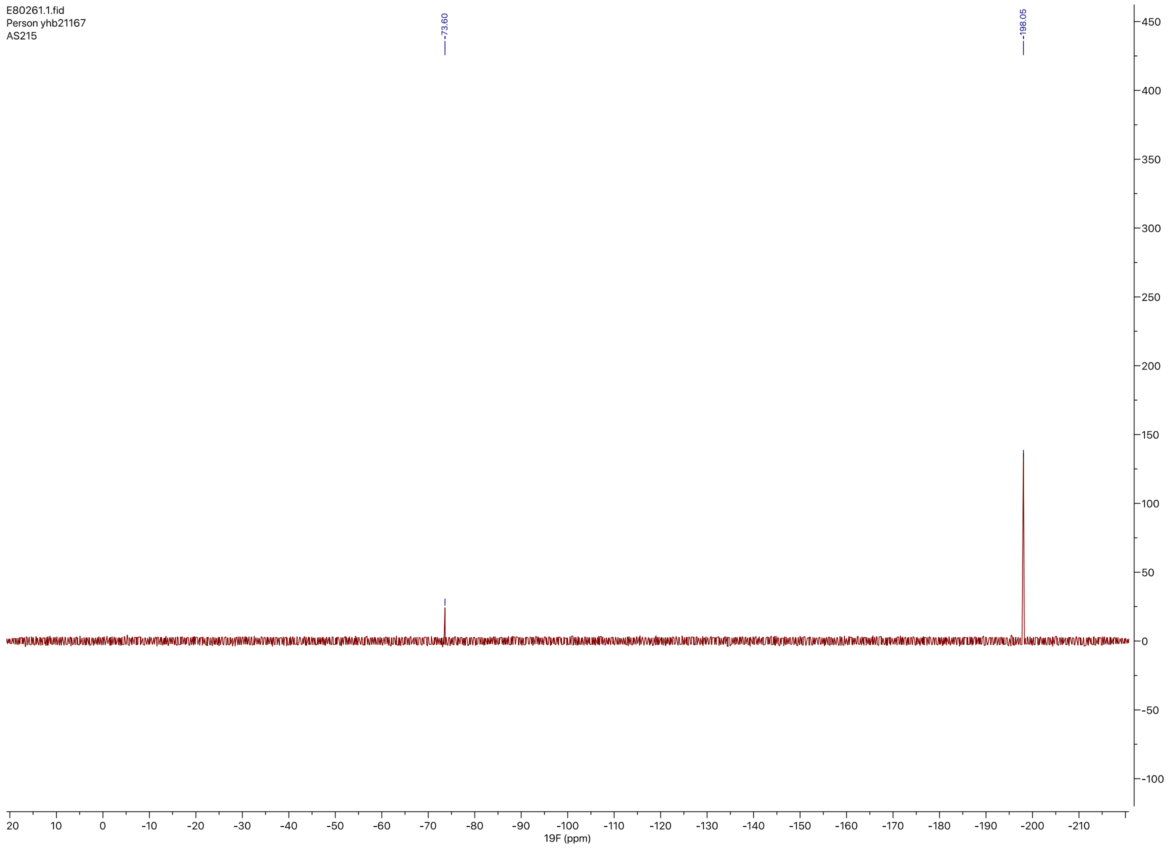

Figure S114 – 19F NMR of compound 30.

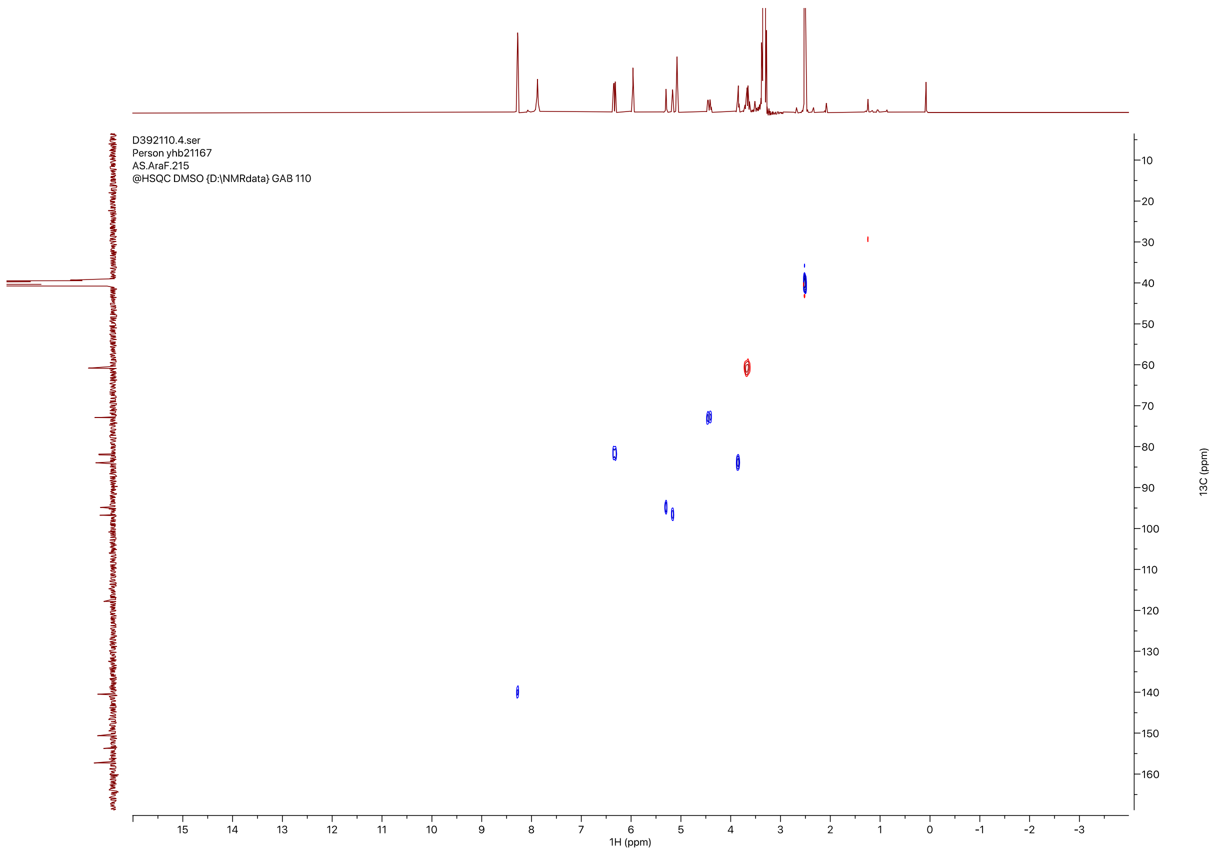

Figure S115 – HSQC of compound 30.

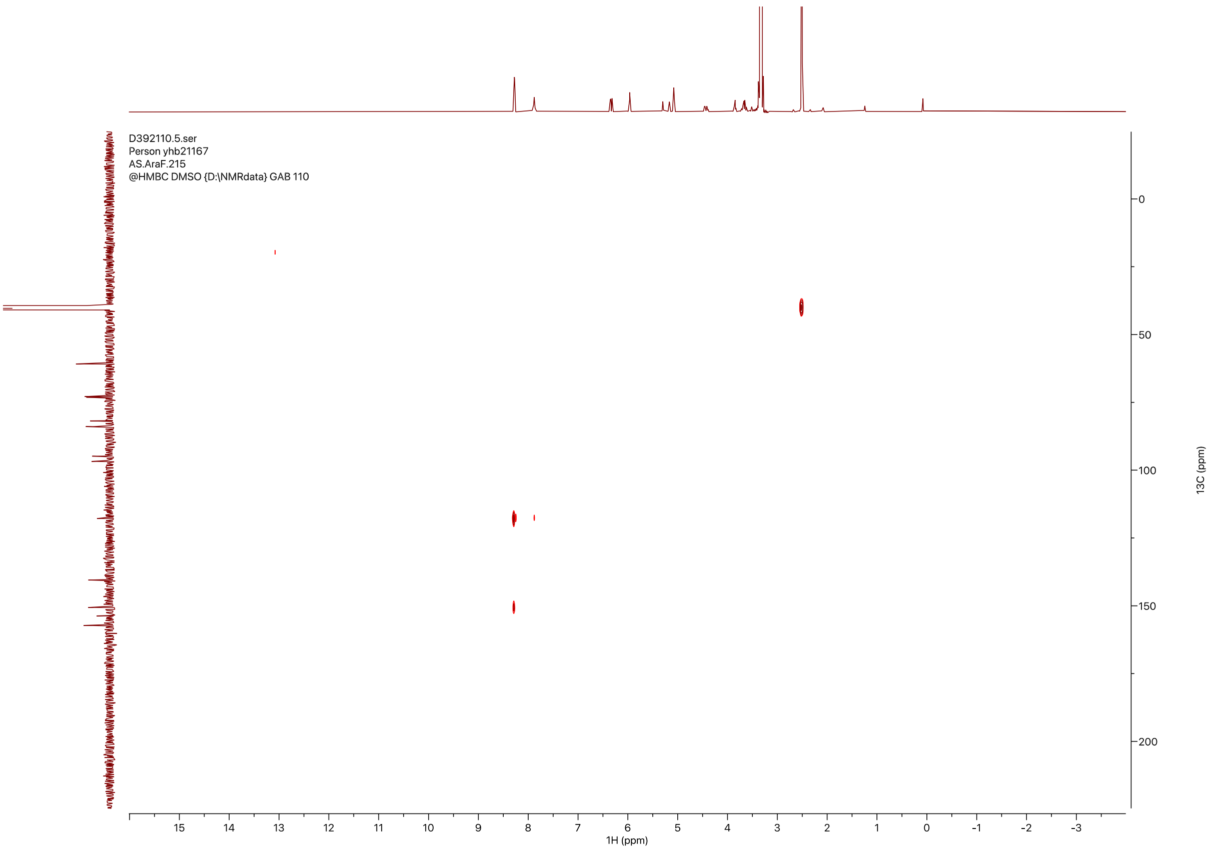

Figure S116 – HMBC of compound 30.

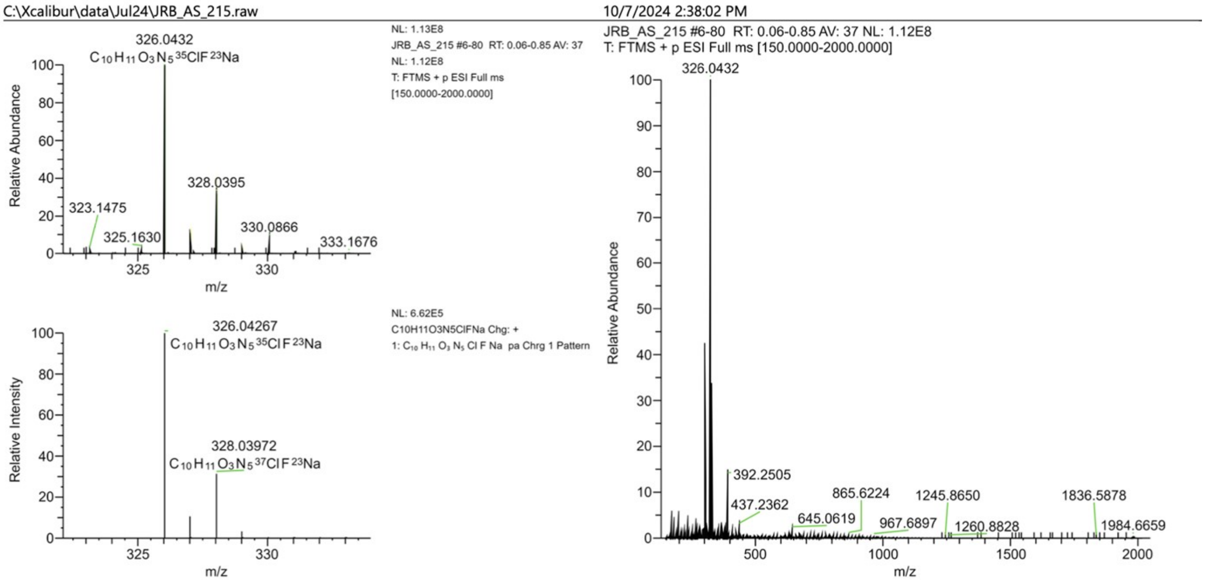

Peak Mass	Display For...	S Fit	RDB	Delta [ppm]	Theo. mass	Rank	Combined S...	# Matched I...	# Missed Iso.	MS Cov. [%]	Pattern Cov....	MSMS Matc...
326.0432	$C_{10}H_{11}O_3N_5{}^{35}ClF{}^{23}Na$	75.7664967931228	6.50	1.50	326.04267	1	96.4	5	0	97.54	100	(Collection)
326.0432	$C_{10}H_8O_2N_8{}^{35}ClF$	57.8272726537054	10.00	-1.76	326.04373	2	95.45	5	0	97.54	100	(Collection)
326.0432	$C_9H_{12}O_6N_4{}^{35}ClF$	51.3159806737275	5.00	2.34	326.04239	3	94.94	5	0	97.36	99.66	(Collection)
326.0432	$C_{11}H_{14}O_7N{}^{35}ClF$	36.5613893000083	4.50	-1.78	326.04373	4	94.16	5	0	97.36	99.6	(Collection)
326.0432	$C_{12}H_{13}O_4N_2{}^{35}ClF{}^{23}Na$	15.5460653222849	6.00	-2.62	326.04401	5	84.55	5	0	88.39	88.07	(Collection)
326.0432	$C_{11}H_3O_2N_{10}F$	25.2020903837969	15.00	3.85	326.04190	6	71.1	2	1	73.65	99.15	(Collection)
326.0432	$C_{12}H_9O_7N_3F$	9.25324376100703	9.50	3.83	326.04190	7	63.83	2	2	66.87	86.58	(Collection)

Figure S117 – HRMS of compound 30.

**Sample name:**	AraF		
**Description:**			
**Sample amount:**	0.000	**Sample type:**	Sample
**Instrument:**	Shimadzu LC	**Location:**	1:17
**Injection date:**	2024-06-25 10:32:06+01:00	**Injection:**	1 of 1
**Acq. method:**	Transglycosylation run-1.5ml-min.amx	**Injection volume:**	10.000 µL

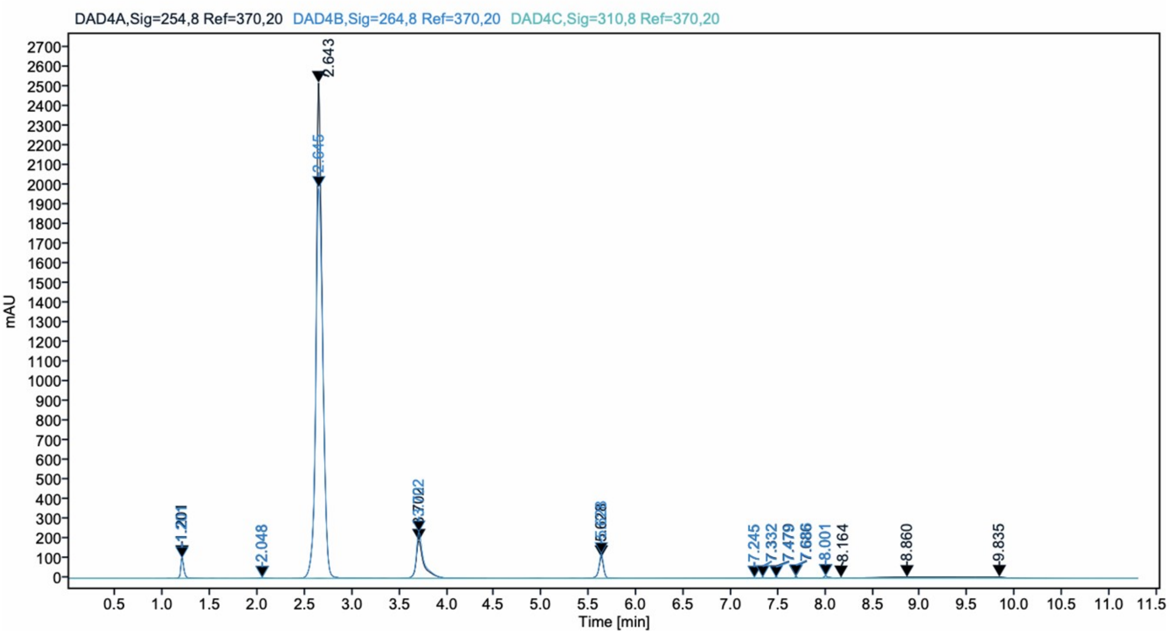

Figure S118 – HPLC trace of the reaction forming compound 30.

2’,3’-dideoxy-3’-thiothymidine (31)

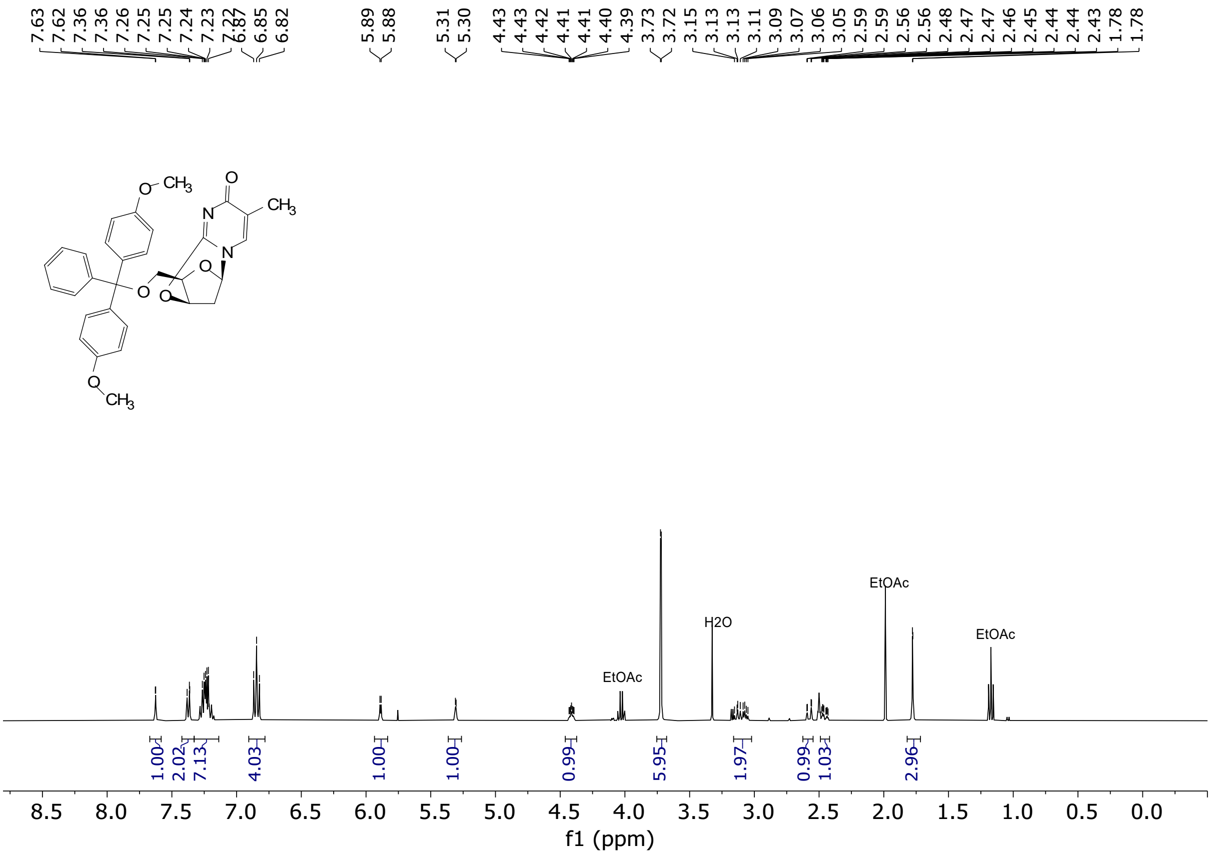

Figure S119 - ^{1}H NMR (400 MHz, $(CD_3)_2SO$) spectrum of 37.

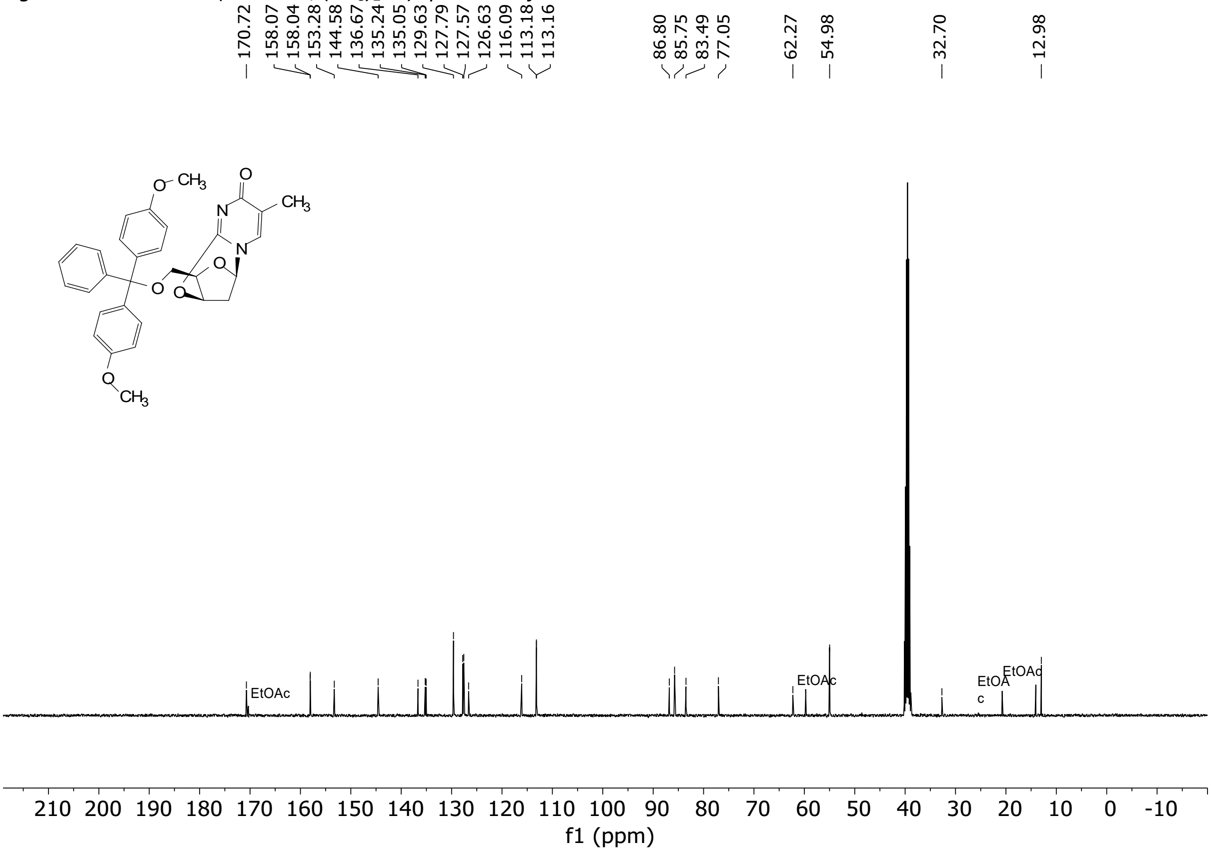

Figure S120 - $^{13}C\{^{1}H\}$ NMR (101 MHz, $(CD_3)_2SO$) spectrum of 37.

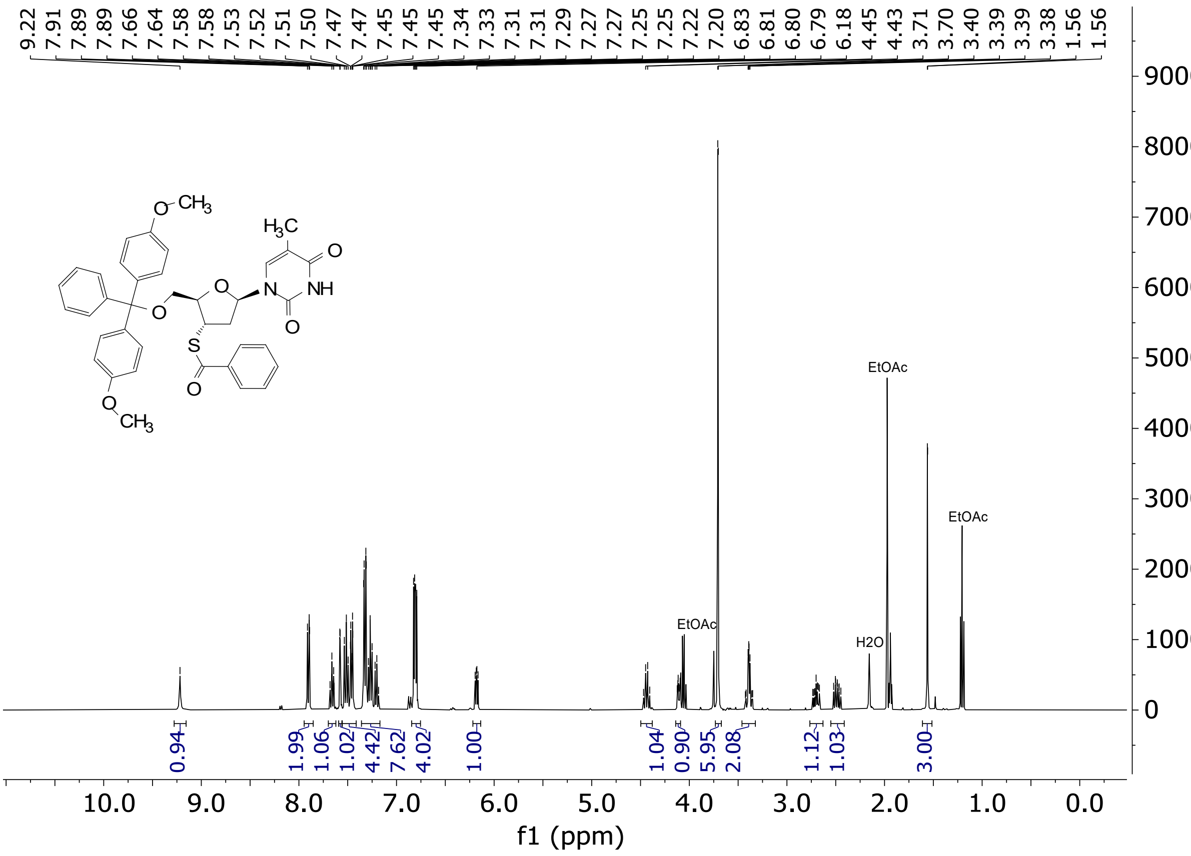

Figure S121 - ^{1}H NMR (400 MHz, CD_3CN) spectrum of 38

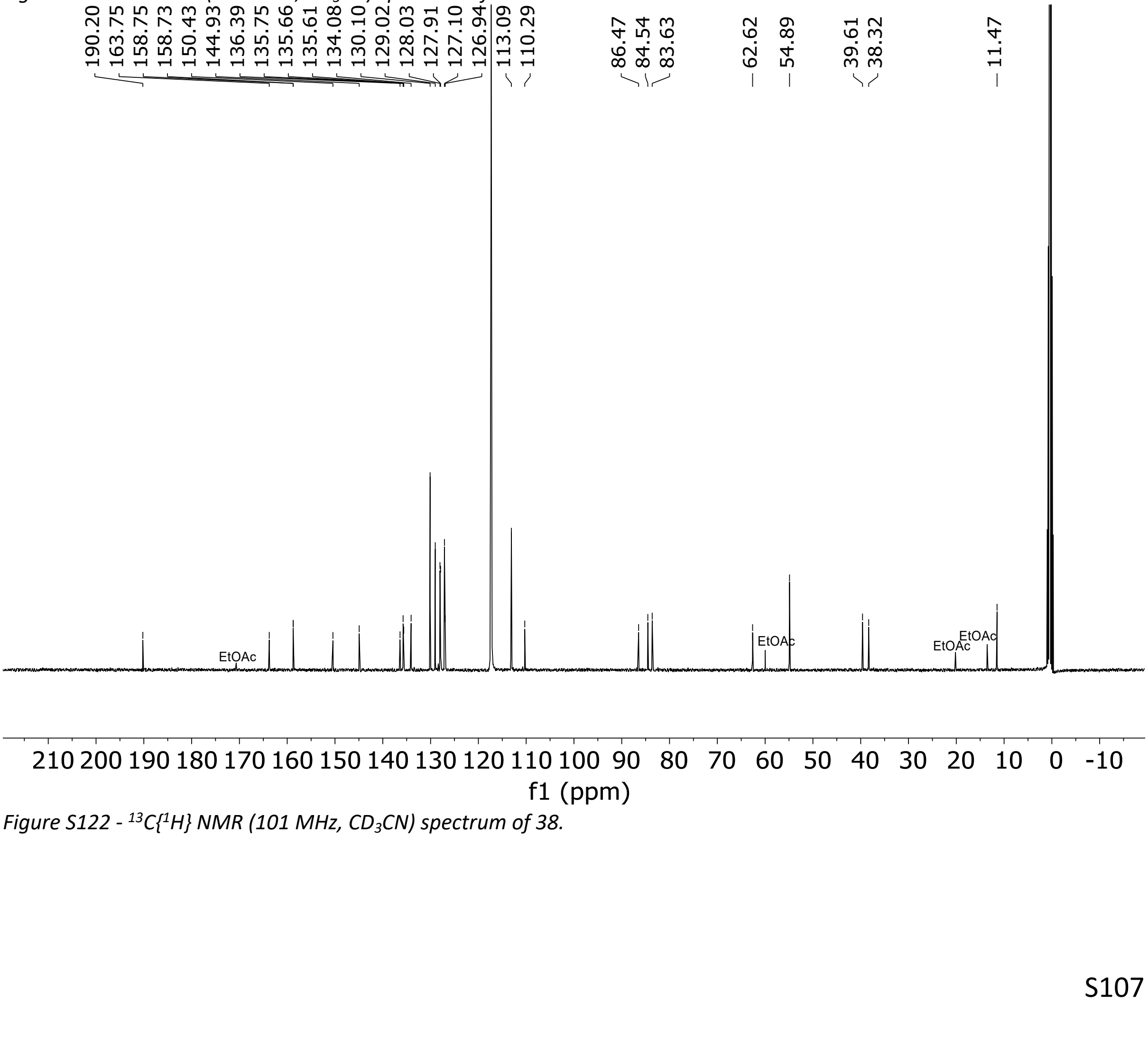

Figure S122 - $^{13}C\{^{1}H\}$ NMR (101 MHz, CD_3CN) spectrum of 38.

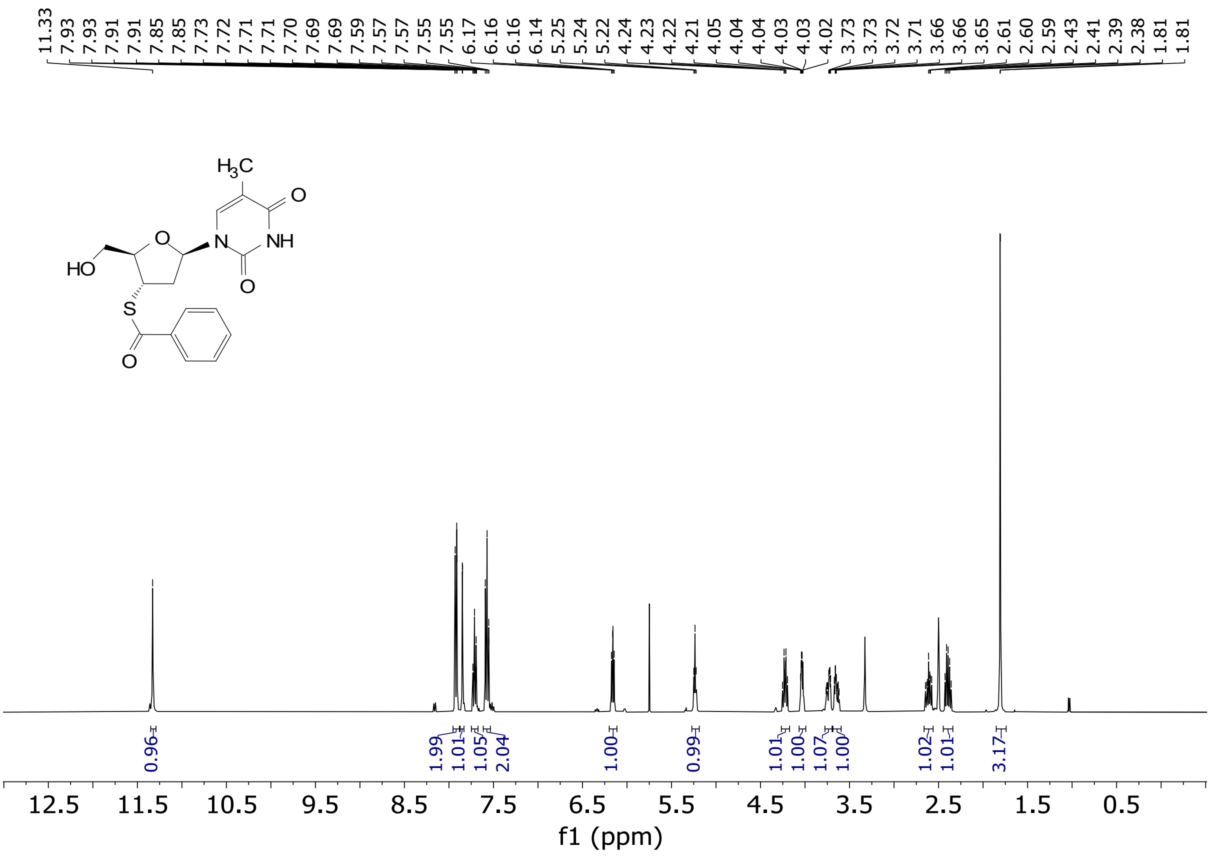

Figure S123 - ^{1}H NMR (400 MHz, $(CD_3)_2SO$) spectrum of 39.

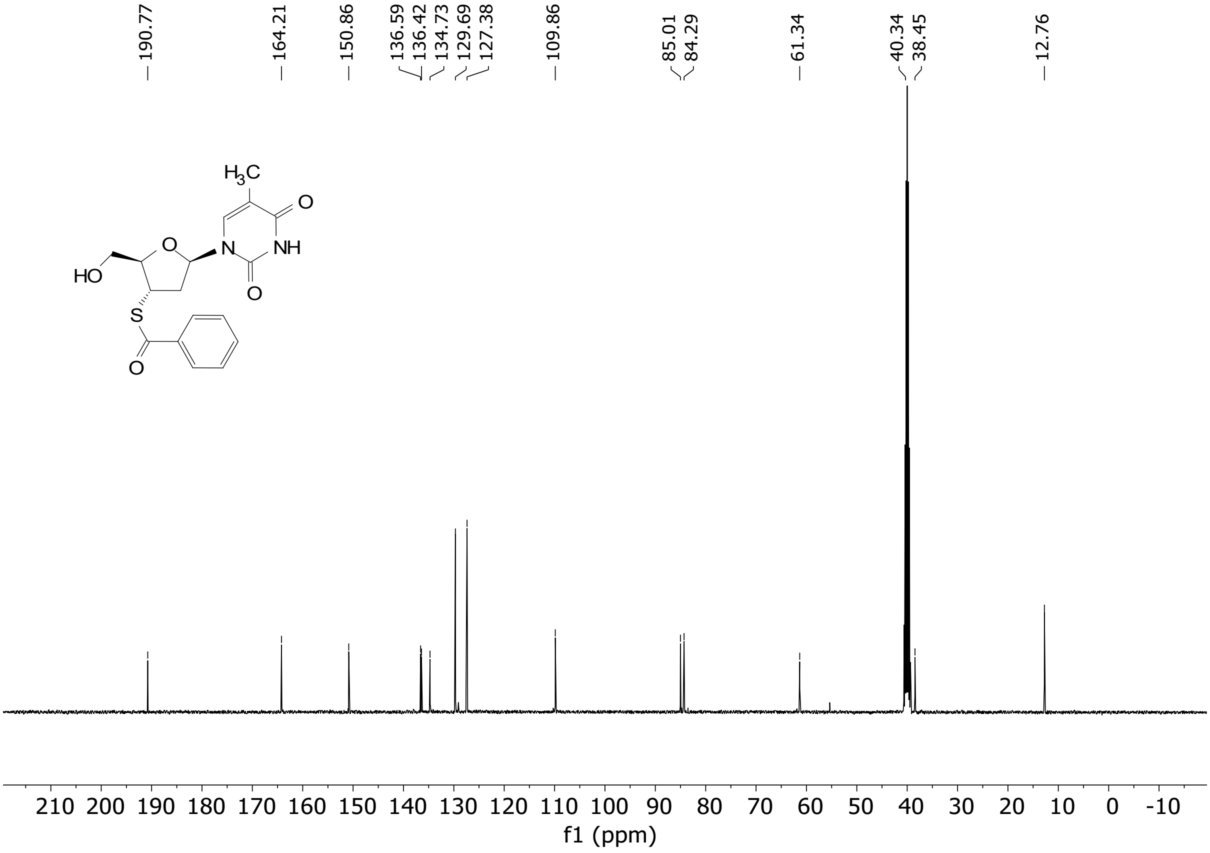

Figure S124 - $^{13}C\{^{1}H\}$ NMR (101 MHz, $(CD_3)_2SO$) spectrum of 39. (Note: chemical shift at 40.34 ppm overlaps with solvent chemical shift pattern.)

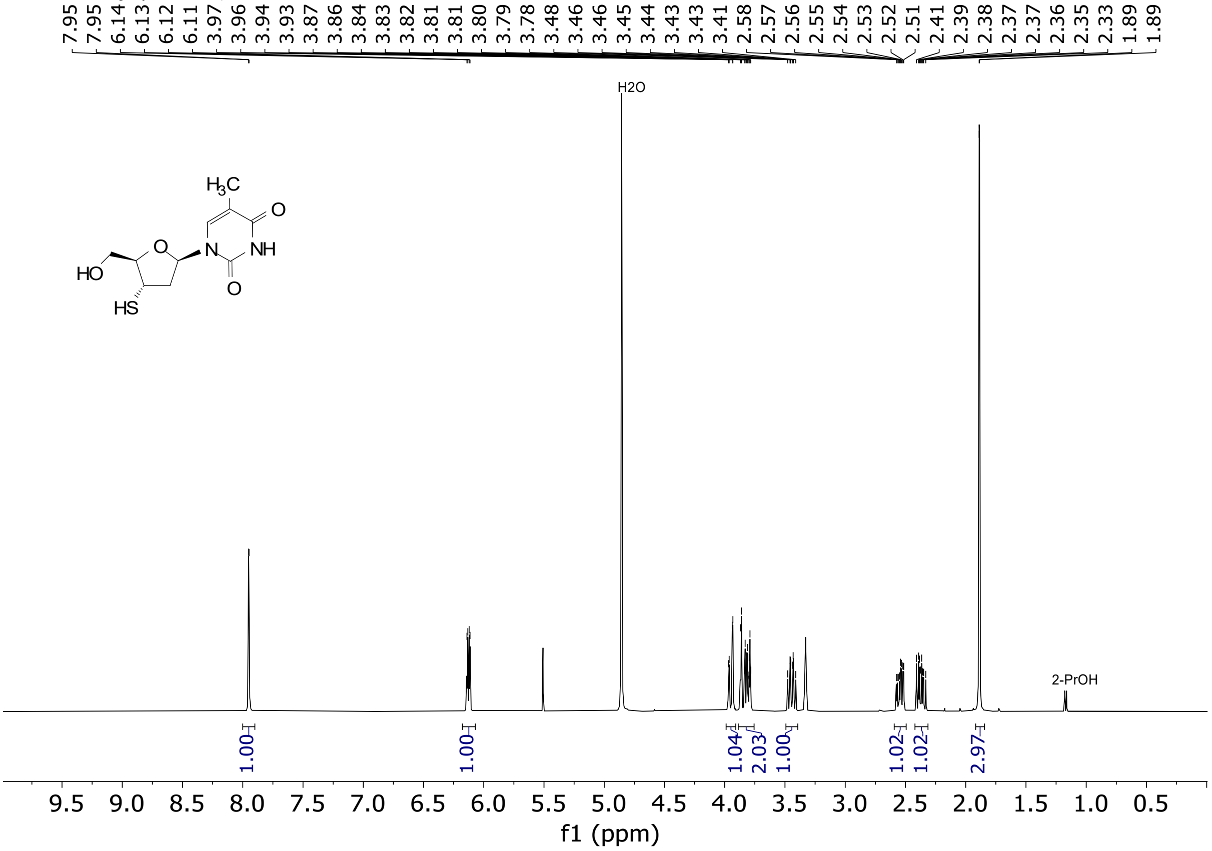

Figure S125 - ^{1}H NMR (400 MHz, MeOD) spectrum of 31.

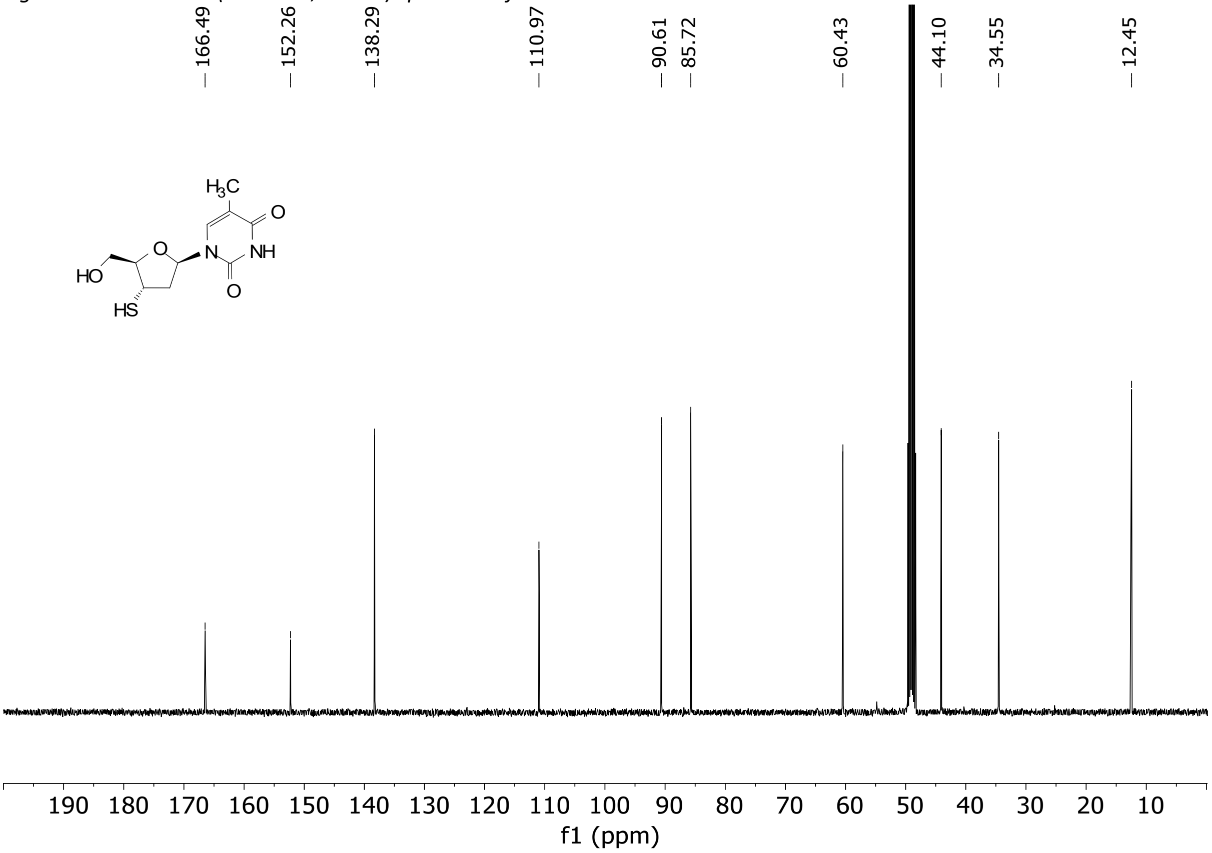

Figure S126 - $^{13}C\{^{1}H\}$ NMR (101 MHz, MeOD) spectrum of 31.

2'-amino-2',3'-dideoxyuridine (32)

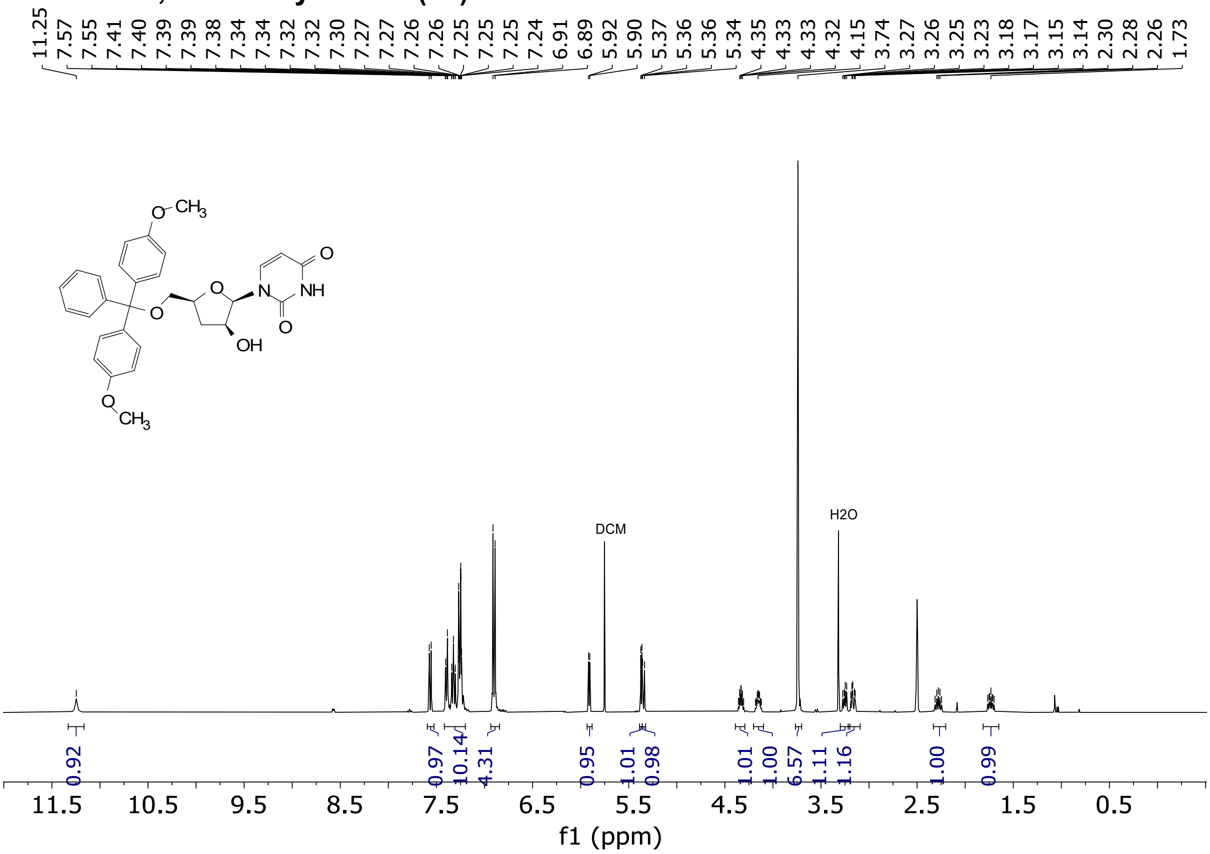

Figure S127 - ^{1}H NMR (400 MHz, $(CD_3)_2SO$) spectrum of 40.

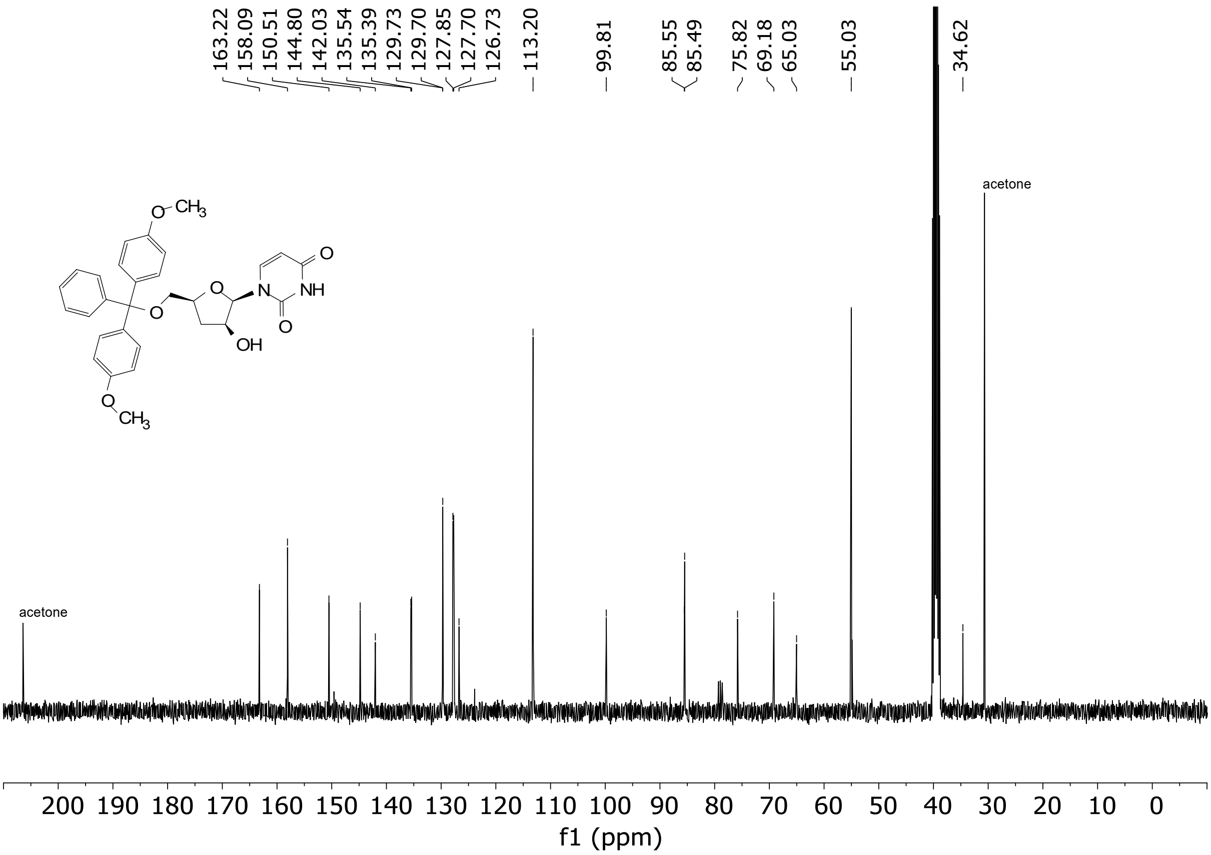

Figure S128 - ^{13}C{^{1}H} NMR (101 MHz, $(CD_3)_2SO$) spectrum of 40.

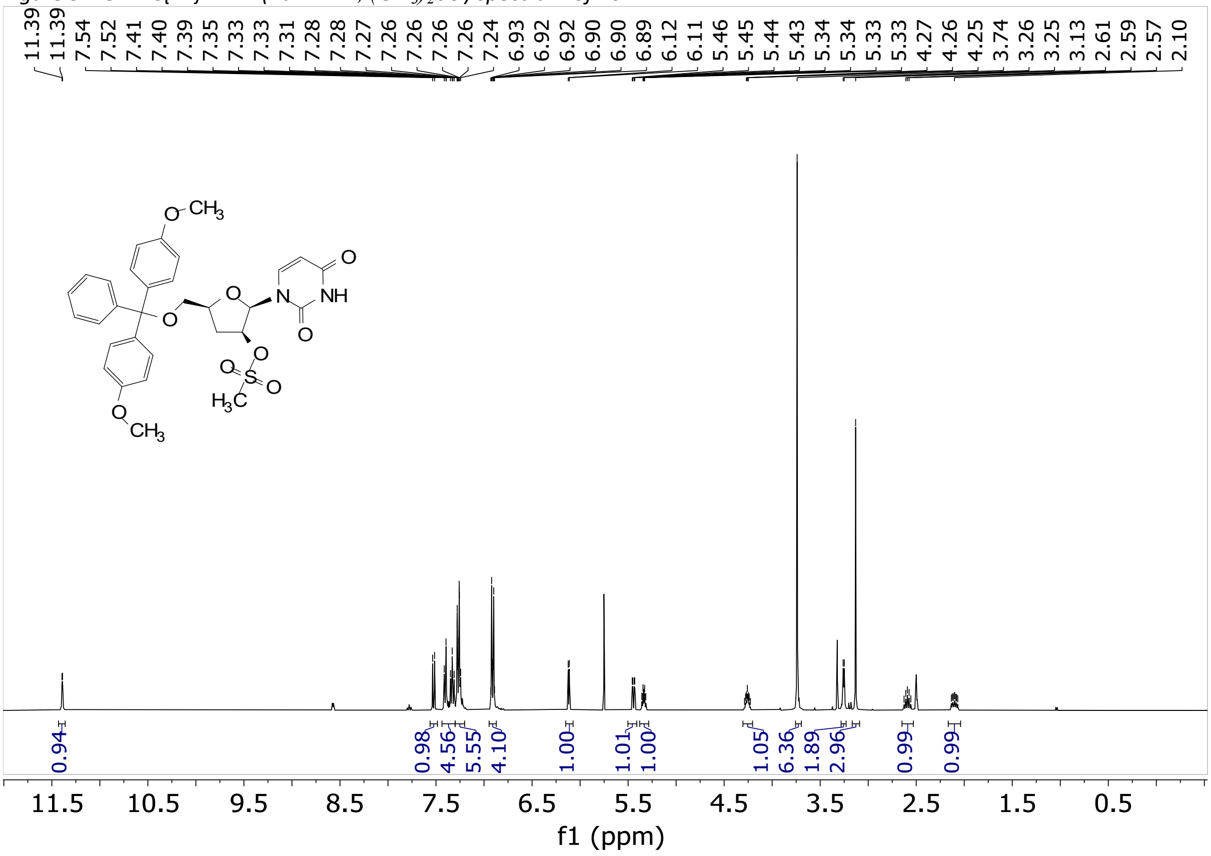

Figure S129 - ^{1}H NMR (400 MHz, $(CD_3)_2SO$) spectrum of 41.

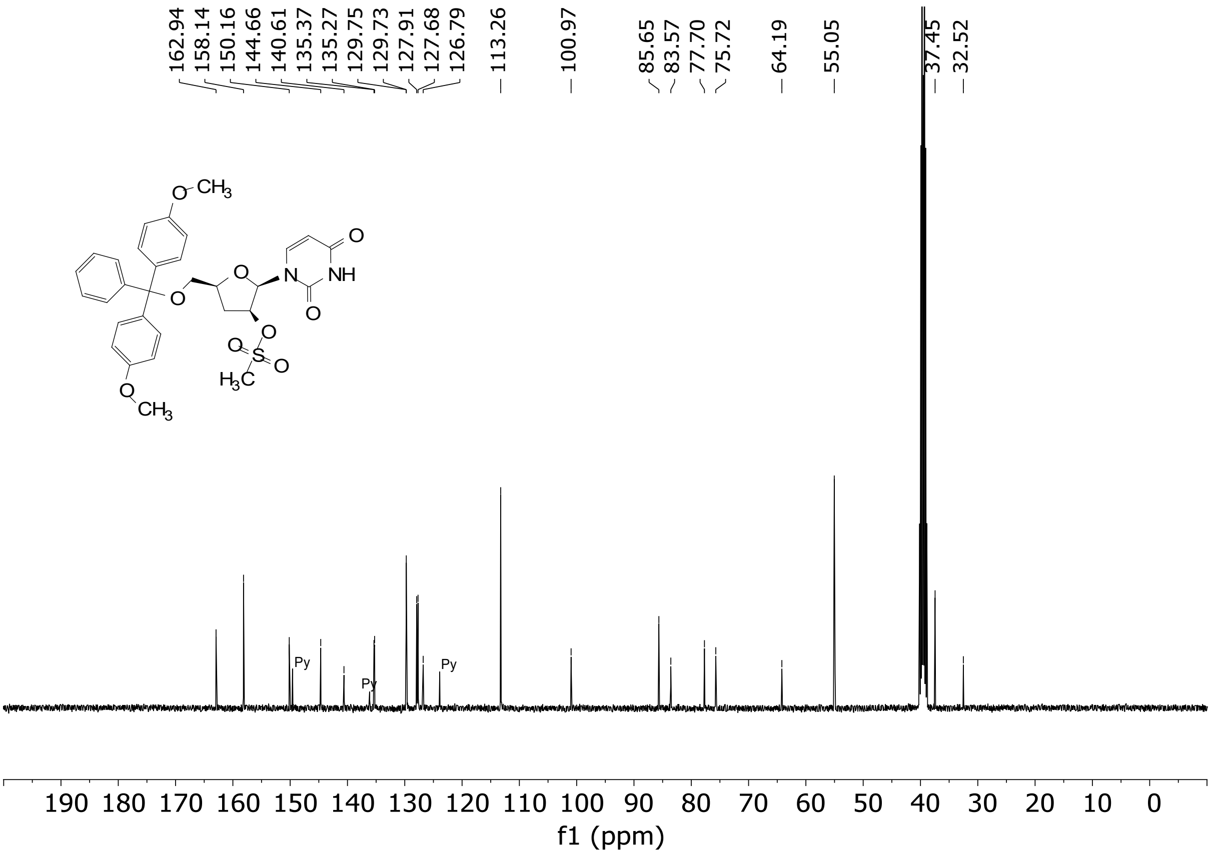

Figure S130 - $^{13}C\{^1H\}$ NMR (101 MHz, $(CD_3)_2SO$) spectrum of 41.

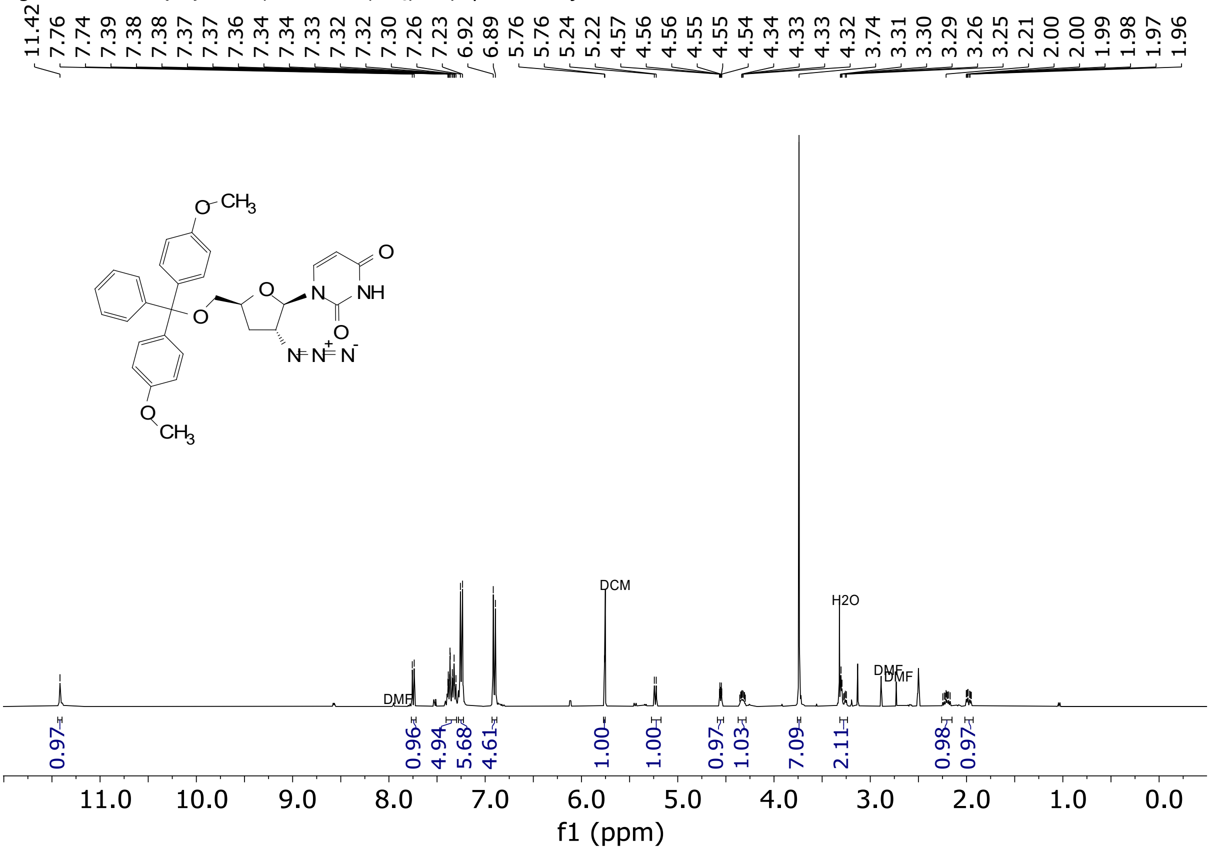

Figure S131 - ^1H NMR (400 MHz, $(CD_3)_2SO$) spectrum of 42.

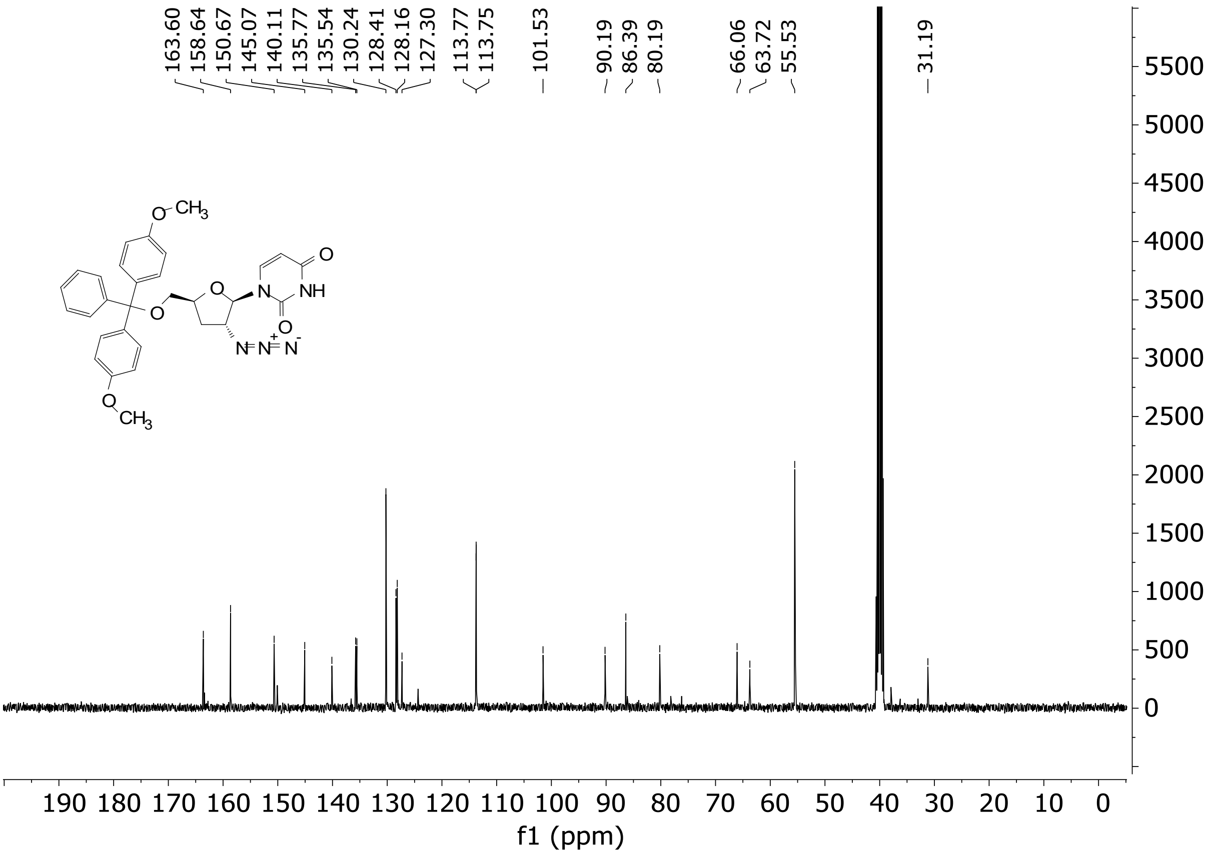

Figure S132 - $^{13}C\{^{1}H\}$ NMR (101 MHz, $(CD_3)_2SO$) spectrum of 42.

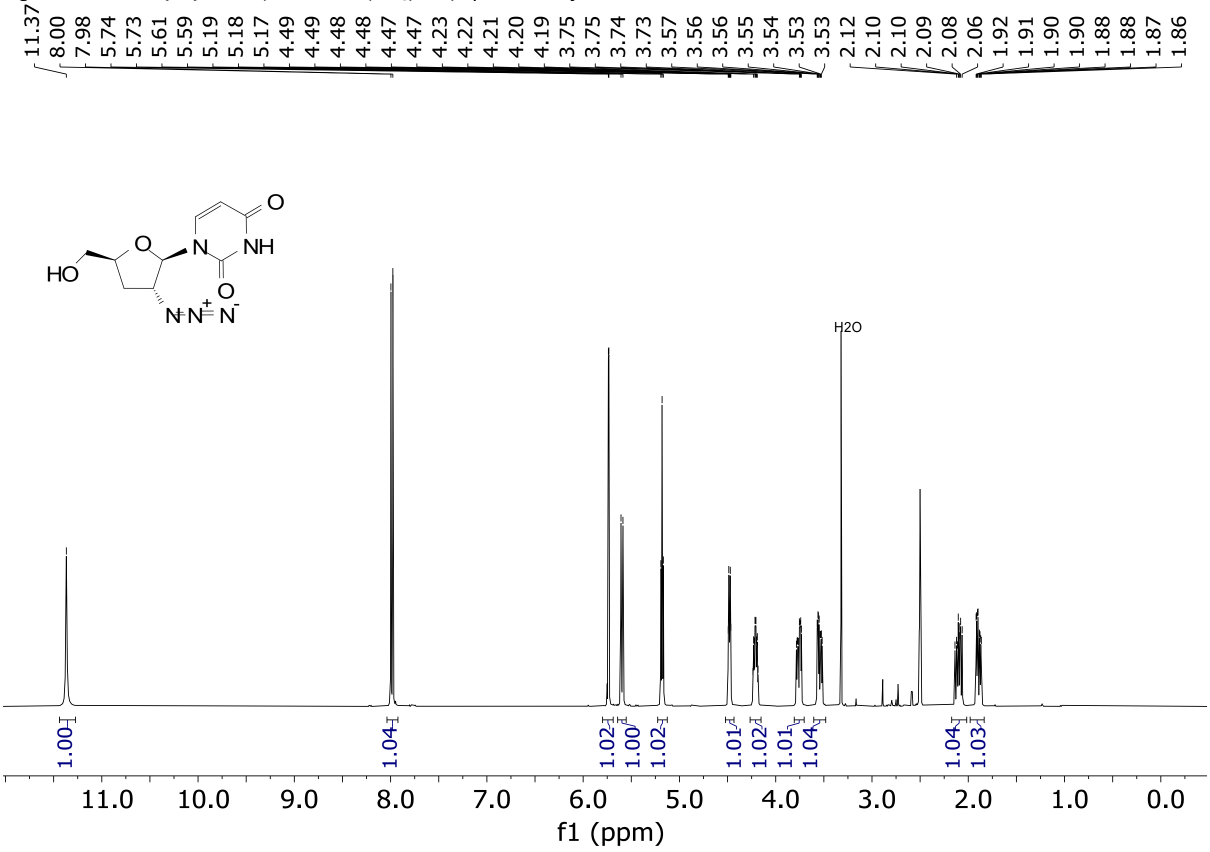

Figure S133 - ^{1}H NMR (400 MHz, $(CD_3)_2SO$) spectrum of 43.

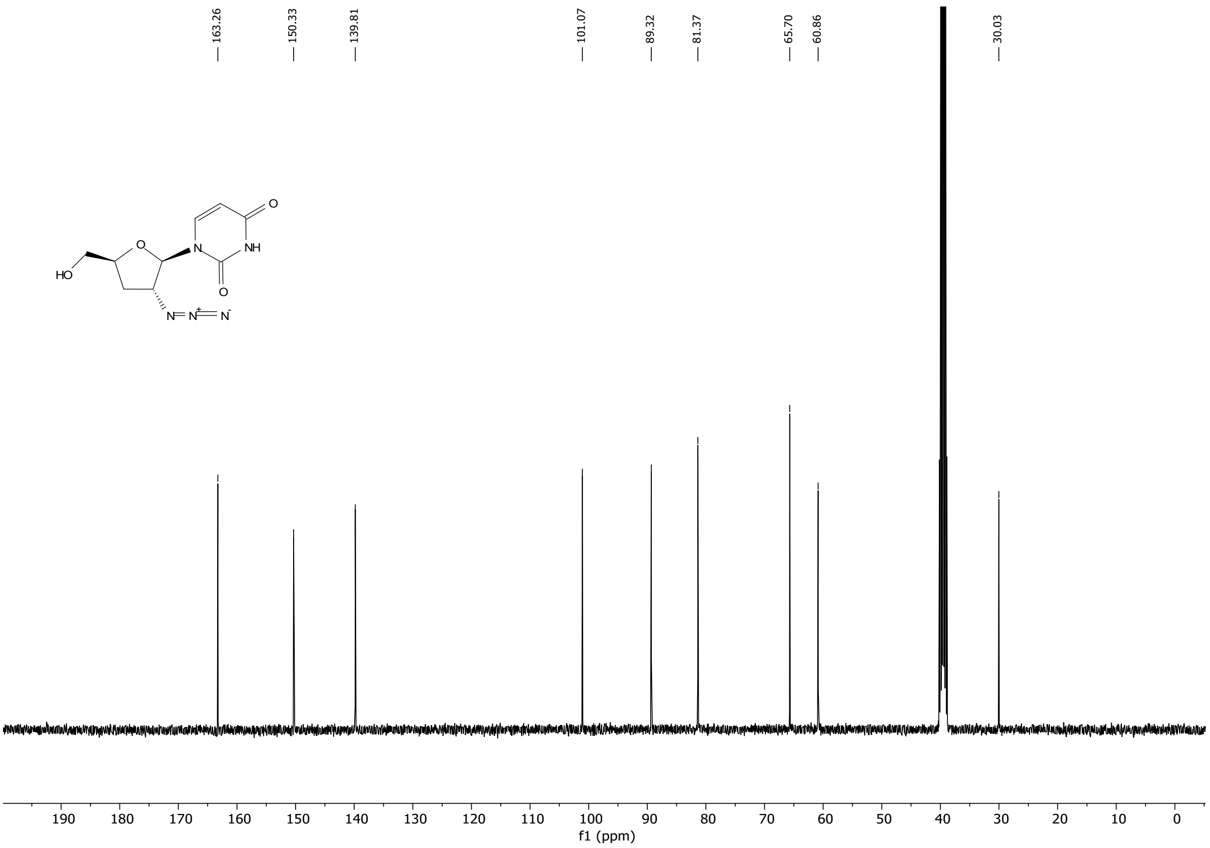

Figure S134. $^{13}C\{^{1}H\}$ NMR (101 MHz, $(CD_3)_2SO$) spectrum of 43.

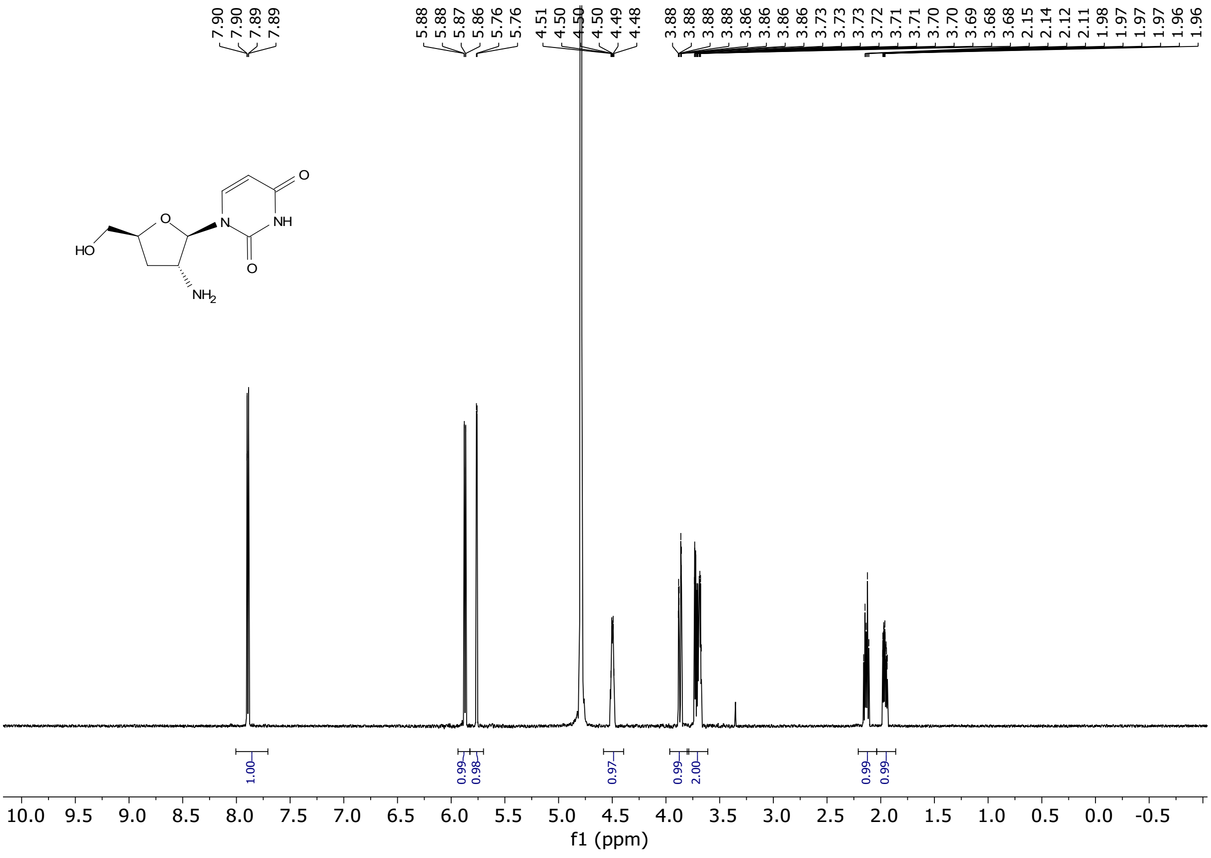

Figure S135 - ^{1}H NMR (600 MHz, D_2O) spectrum of 32.

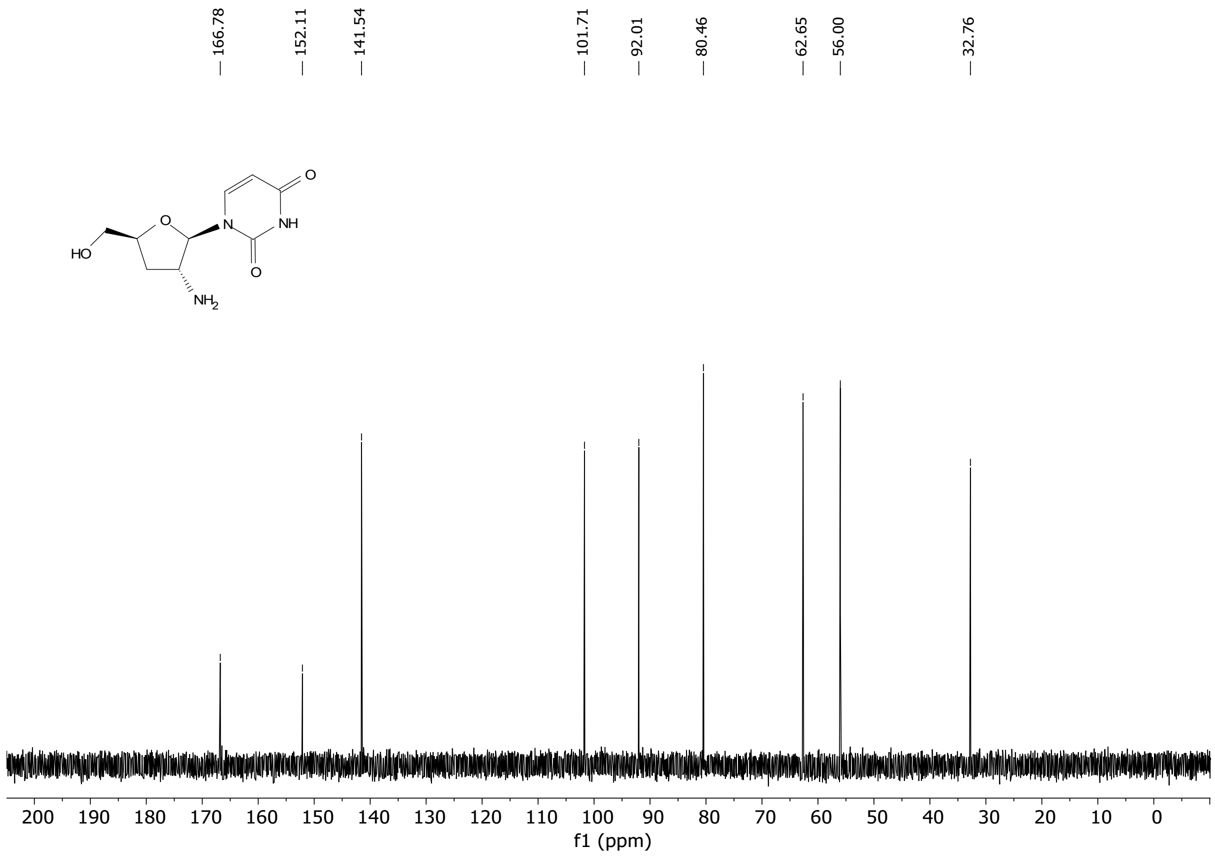

Figure S136 - $^{13}C\{^{1}H\}$ NMR (151 MHz, D_2O) spectrum of 32.

2’-amino-2’-deoxyuridine (33)

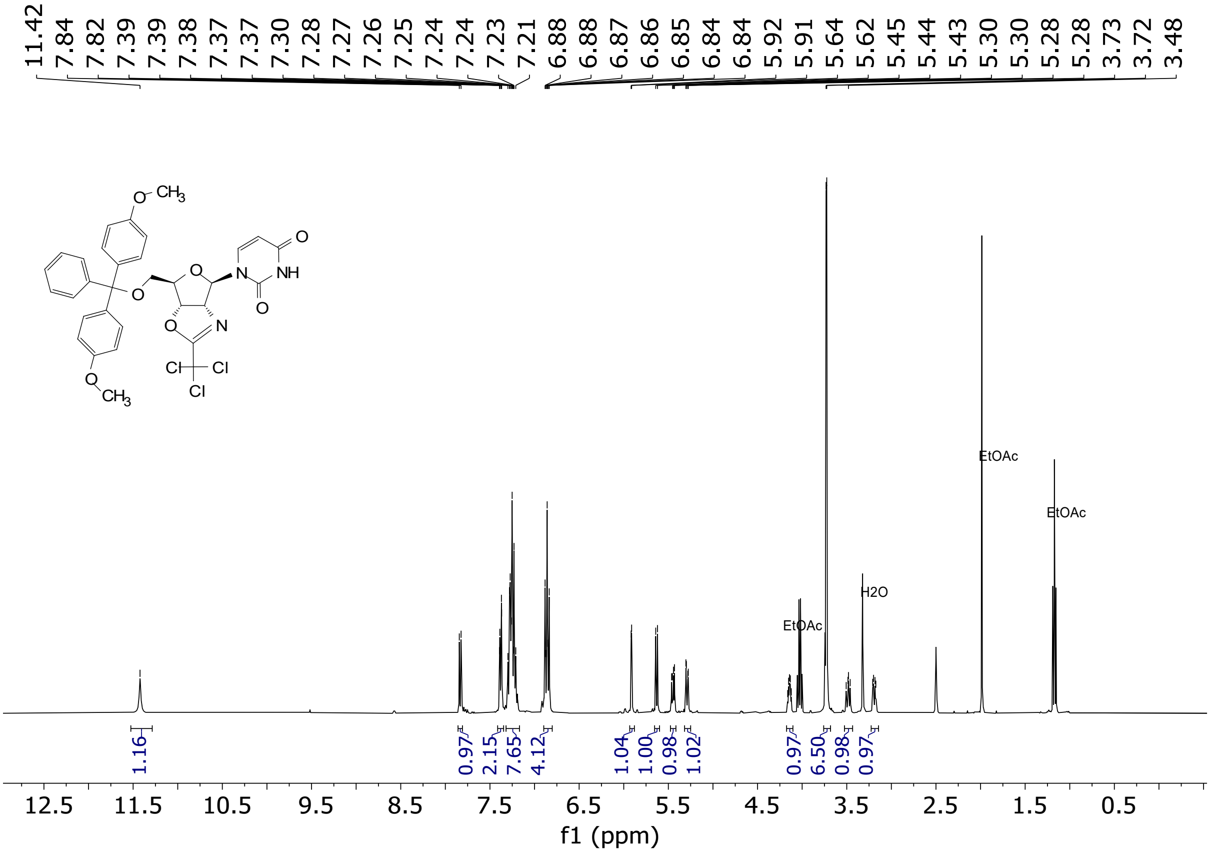

Figure S137 - ¹H NMR (400 MHz, $(CD_3)_2SO$) spectrum of 44.

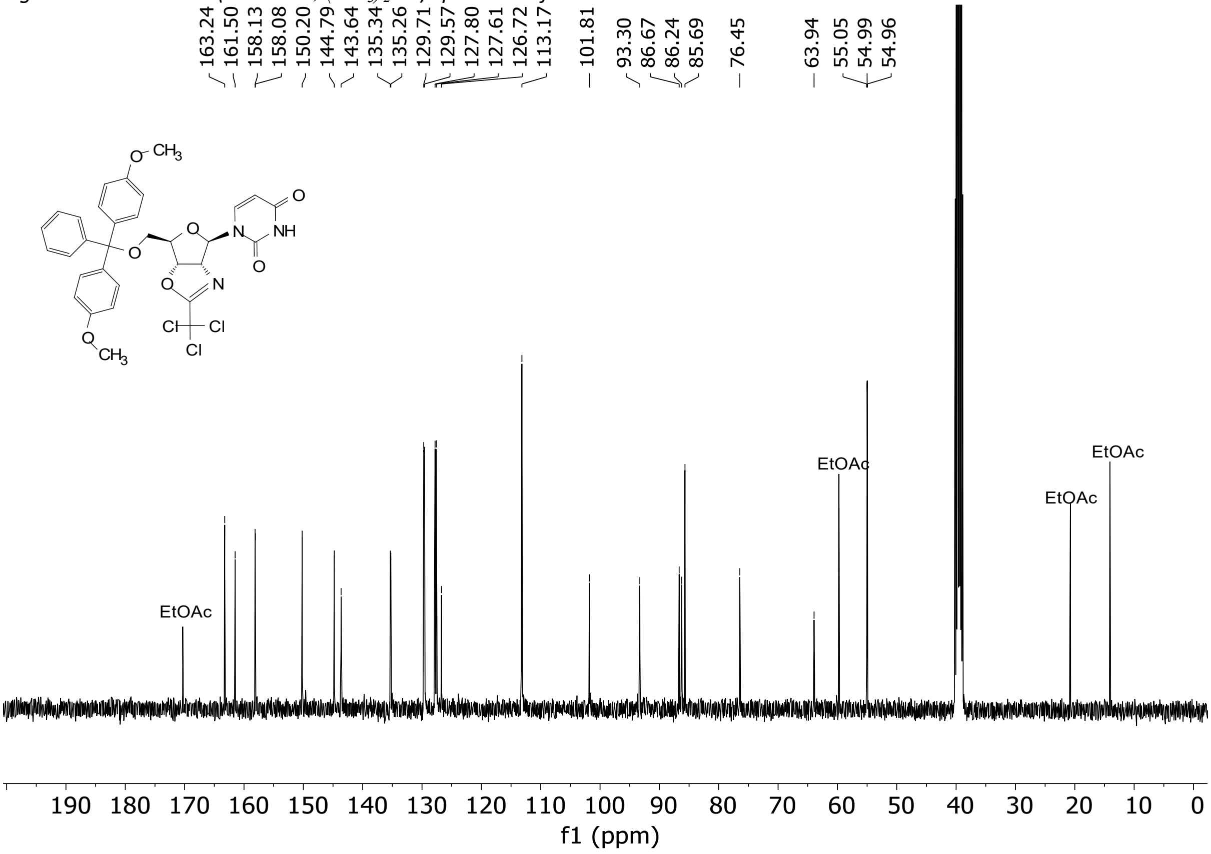

Figure S138 - $^{13}C\{^1H\}$ NMR (101 MHz, $(CD_3)_2SO$) spectrum of 44.

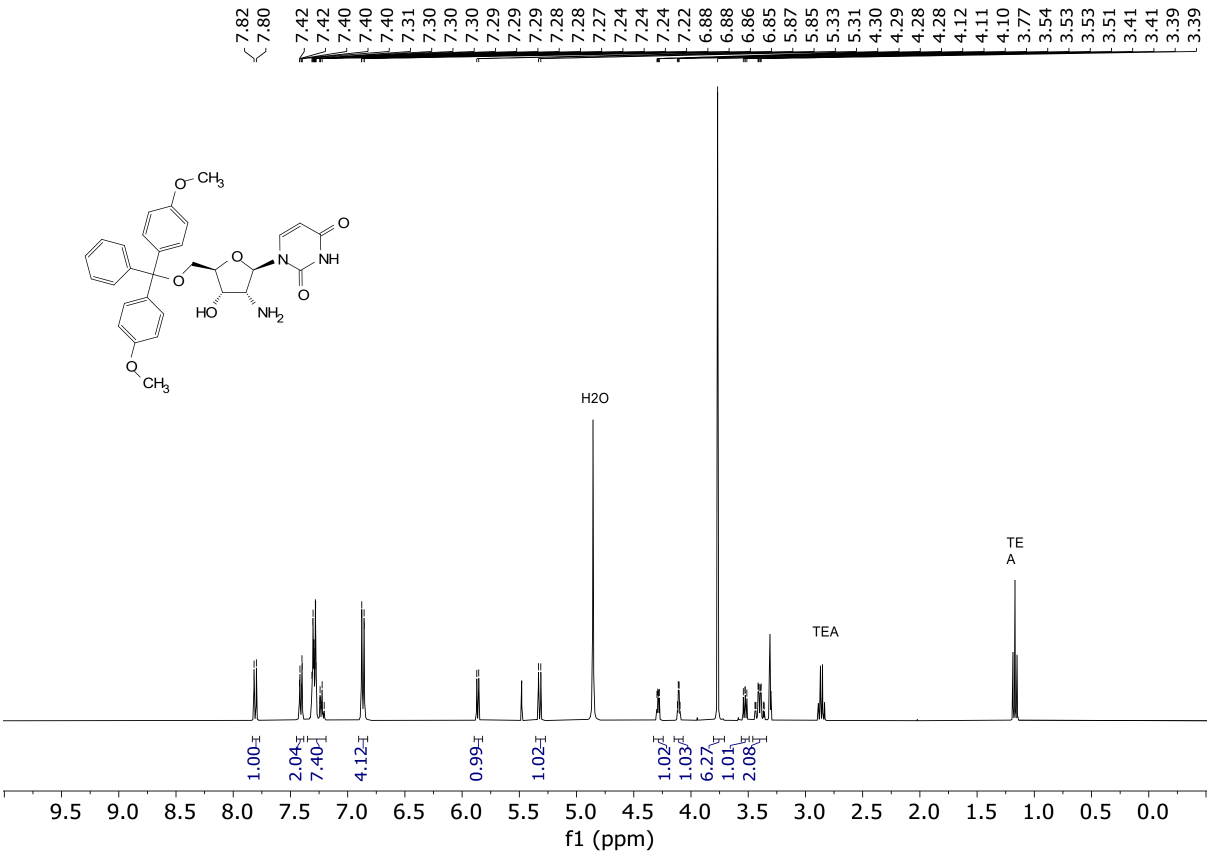

Figure S139 - ^{1}H NMR (400 MHz, MeOD) spectrum of 45.

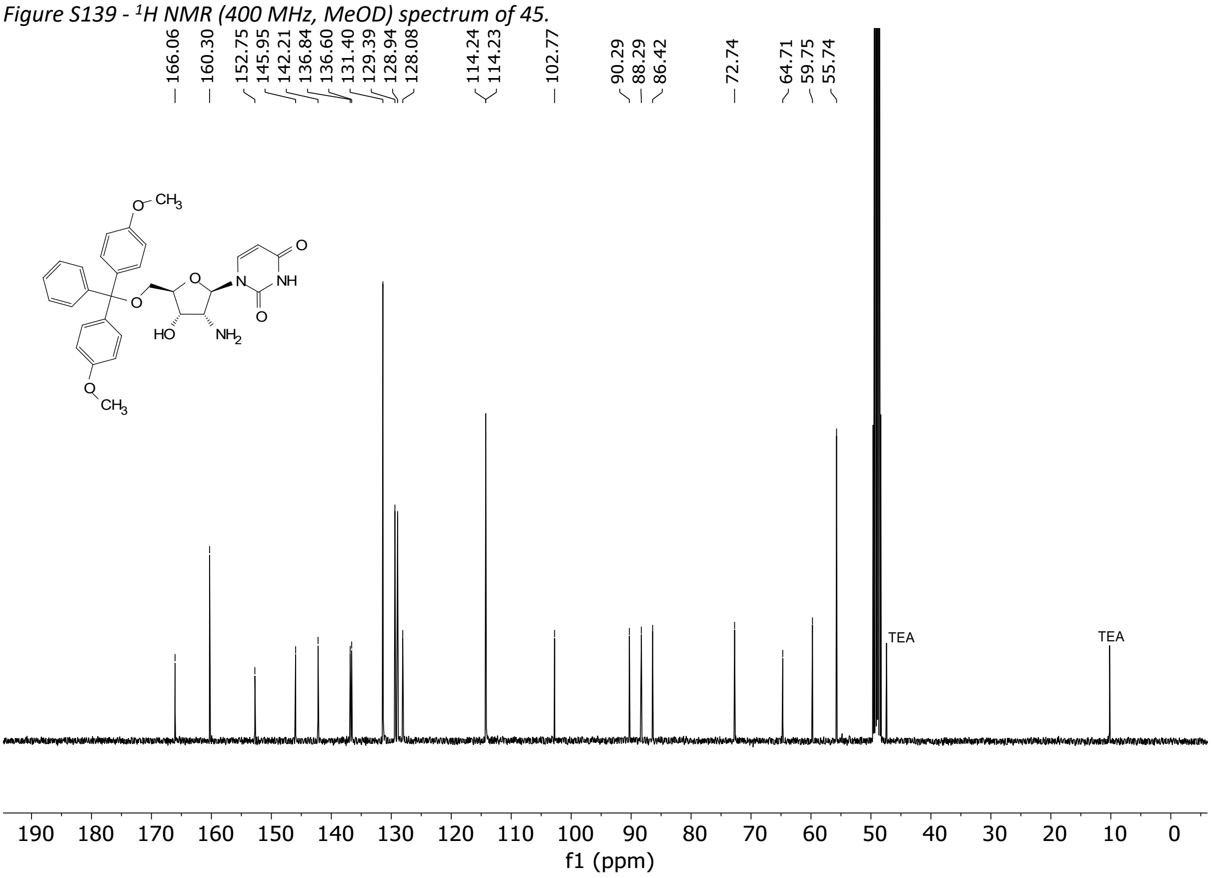

Figure S140 - ^{13}C{^{1}H} NMR (101 MHz, MeOD) spectrum of 45.

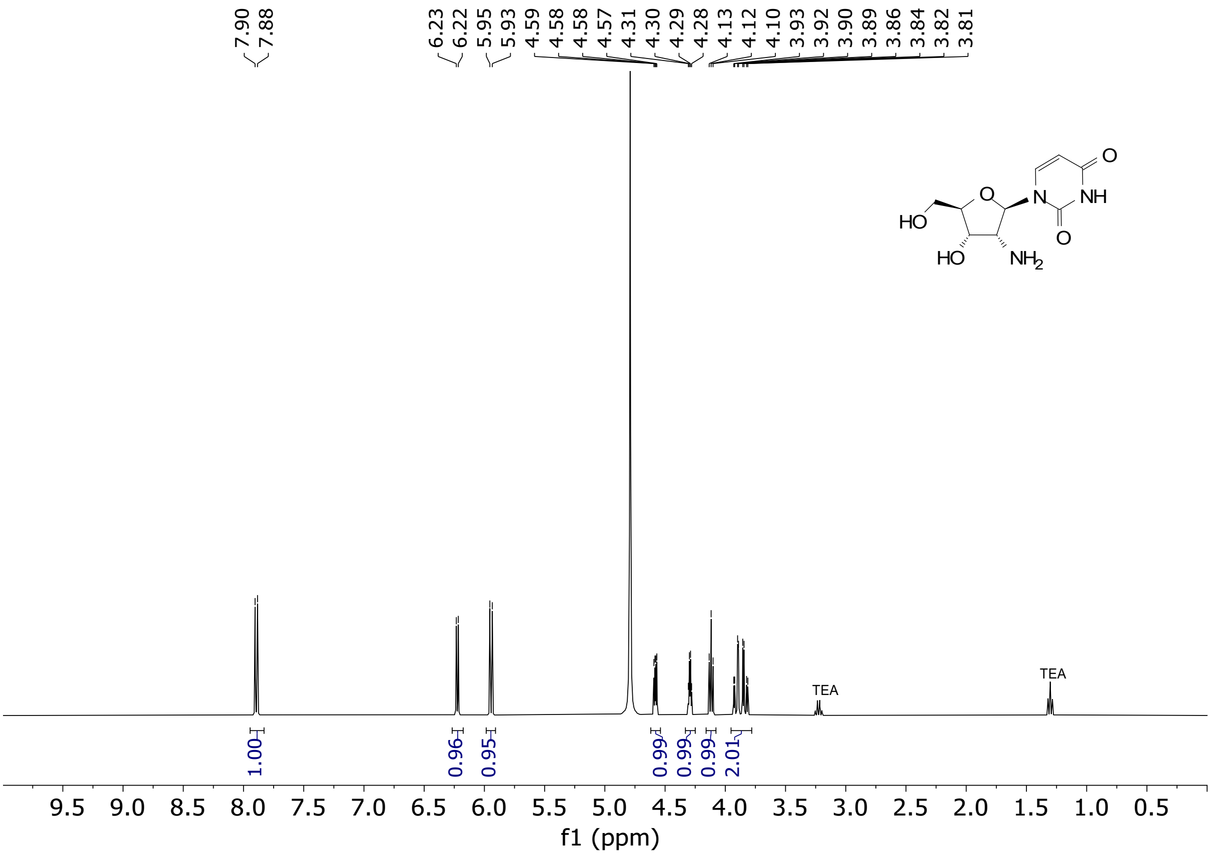

Figure S141 - ^{1}H NMR (400 MHz, D_2O) spectrum of 33.

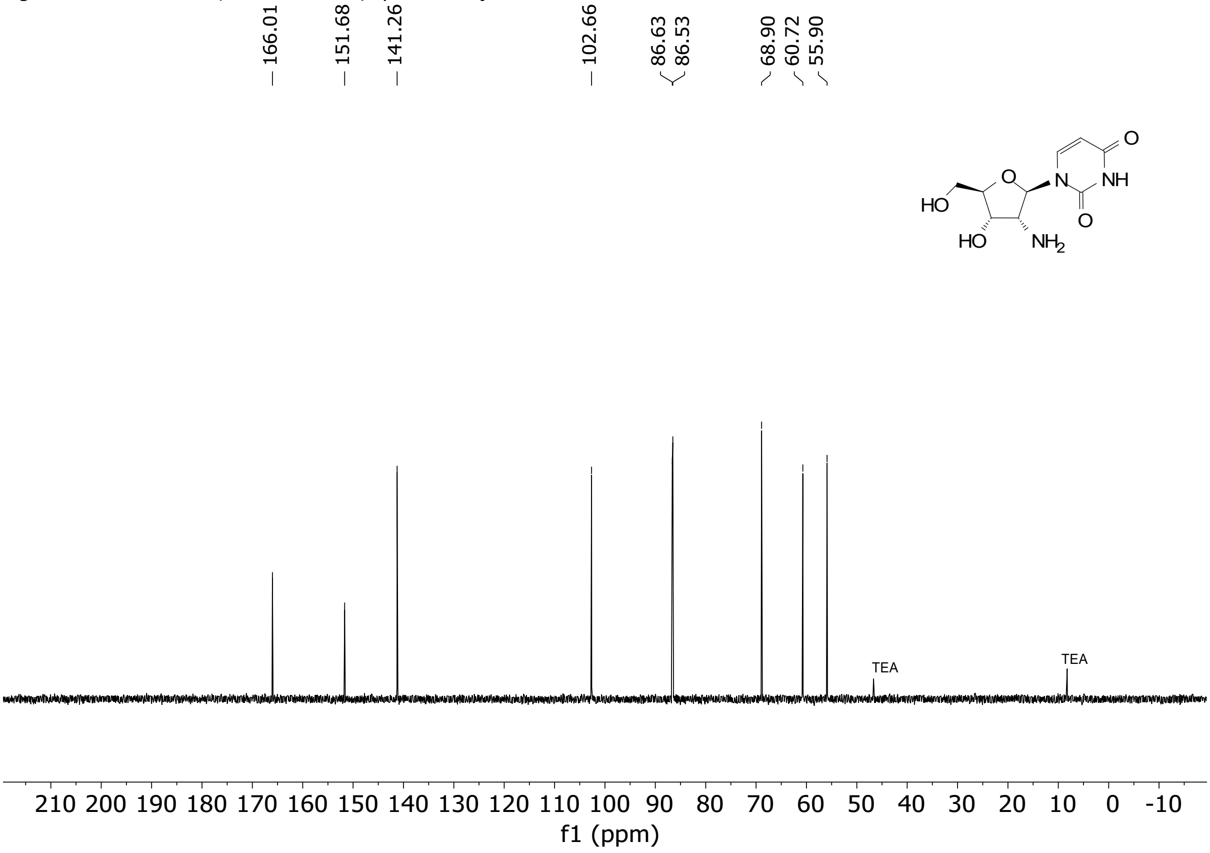

Figure S142 - $^{13}C\{^{1}H\}$ NMR (101 MHz, D_2O) spectrum of 33.

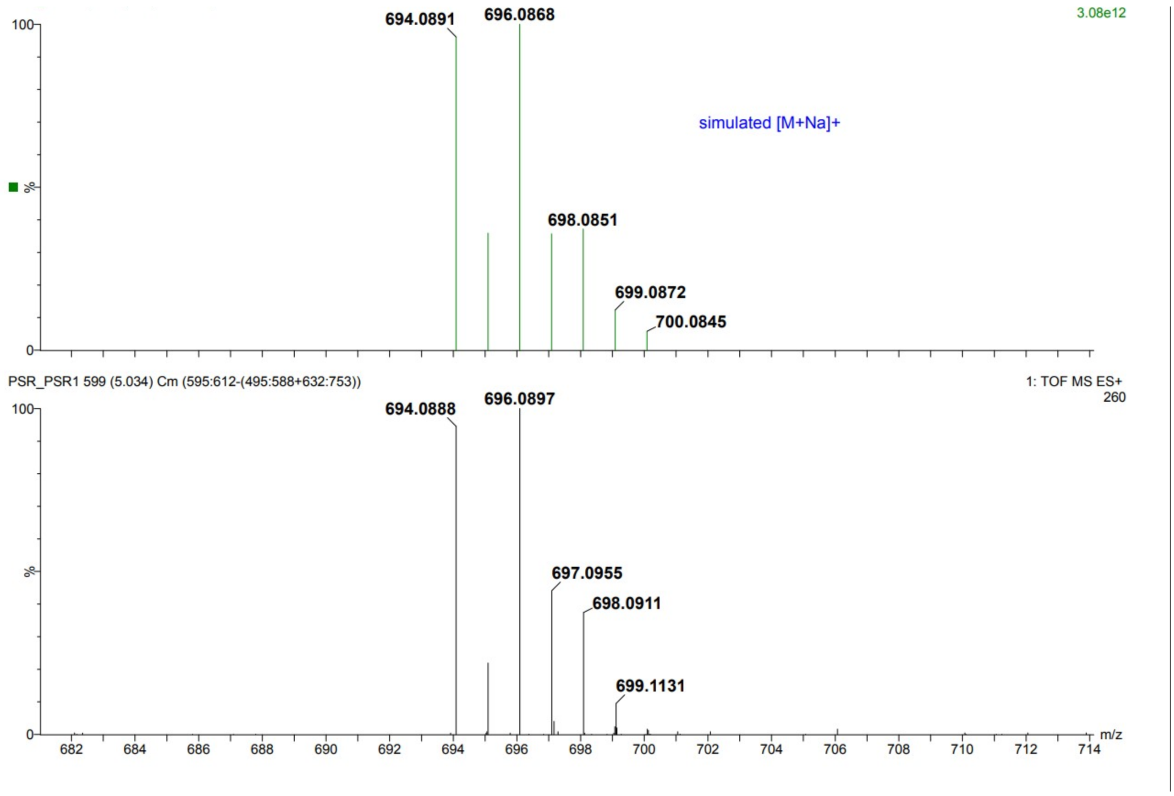

Figure S143 - Simulated and experimental HRMS isotopic pattern measured for 44 ion [M+Na]+.

8. References

(1) Downey, A. M.; Richter, C.; Pohl, R.; Mahrwald, R.; Hocek, M. Direct One-Pot Synthesis of Nucleosides from Unprotected or 5-O-Monoprotected D-Ribose. *Org Lett* **2015**, *17*, 4604-4607.

(2) Mikhailopoulo, I. A.; Kalinichenko, E. N.; Podkopaeva, T. L.; Wenzel, T.; Rosemeyer, H.; Seela, F. L. Synthesis of 1-Deazaadenosine Analogues of (2'→5') ApApA. *Nucleosides and Nucleotides* **2006**, *15*, 445-464.

(3) Shet, H.; Patel, M.; Waikar, J. M.; More, P. M.; Sanghvi, Y. S.; Kapdi, A. R. Room-Temperature Dialkylamination of Chloroheteroarenes Using a Cu(II)/PTABS Catalytic System. *Chem Asian J* **2023**, *18*, e202201006.

(4) Seitz, F.; Jungnickel, T.; Kleiber, N.; Kretschmer, J.; Dietzsch, J.; Adelmann, J.; Bohnsack, K. E.; Bohnsack, M. T.; Höbartner, C. Atomic Mutagenesis of N6-Methyladenosine Reveals Distinct Recognition Modes by Human m6A Reader and Eraser Proteins. *Journal of the American Chemical Society* **2024**, *146*, 7803-7810.

(5) Gundersen, L.-L.; Brændvang, M. A Novel Method for the Introduction of Fluorine into the Purine 2-Position: Synthesis of 2-Fluoroadenosine and a Formal Synthesis of the Antileukemic Drug Fludarabine. *Synthesis* **2006**, *2006*, 2993-2995.

(6) Yasumoto, M.; Moriyama, A.; Unemi, N.; Hashimoto, S.; Suzue, T. Studies of antitumor agents. 1. Resolution of racemic 1-(tetrahydro-2-furanyl)-k-fluorouracil into the R and S isomers and examination of the biological activities of the isomers. *J Med Chem* **1977**, *20*, 1592-1594.

(7) Takatsuki, K.; Ohgushi, S.; Kohmoto, S.; Kishikawa, K.; Yamamoto, M. A simple and efficient synthesis of puromycin, 2,2'-anhydro-pyrimidine nucleosides, cytidines and 2',3'-

anhydroadenosine from 3',5'-O-sulfinyl xylo-nucleosides. *Nucleosides Nucleotides Nucleic Acids* **2006**, *25*, 719-734.
(8) Beasley, S.; Nguyen, K.; Fazio, M.; Spitale, R. C. Protected pyrimidine nucleosides for cell-specific metabolic labeling of RNA. *Tetrahedron Lett* **2018**, *59*, 3912-3915.
(9) Leszczynska, G.; Leonczak, P.; Wozniak, K.; Malkiewicz, A. Chemical synthesis of the 5-taurinomethyl(-2-thio)uridine modified anticodon arm of the human mitochondrial tRNA(Leu(UUR)) and tRNA(Lys). *RNA* **2014**, *20*, 938-947.
(10) Thomas, H. J.; Tiwari, K. N.; Clayton, S. J.; Secrist, J. A.; Montgomery, J. A. Synthesis and Biologic Activity of Purine 2′-Deoxy-2′-fluoro-ribonucleosides. *Nucleosides and Nucleotides* **1994**, *13*, 309-323.
(11) Montgomery, J. A.; Shortnacy-Fowler, A. T.; Clayton, S. D.; Riordan, J. M.; Secrist, J. A., 3rd. Synthesis and biologic activity of 2'-fluoro-2-halo derivatives of 9-beta-D-arabinofuranosyladenine. *J Med Chem* **1992**, *35*, 397-401.
(12) Kabsch, W. XDS. *Acta Crystallographica Section D* **2010**, *66*, 125-132.
(13) Evans, P. Scaling and assessment of data quality. *Acta Crystallogr D Biol Crystallogr* **2006**, *62*, 72-82.
(14) Winter, G. xia2: an expert system for macromolecular crystallography data reduction. *Journal of Applied Crystallography* **2010**, *43*, 186-190.
(15) Vagin, A.; Teplyakov, A. MOLREP: an Automated Program for Molecular Replacement. *Journal of Applied Crystallography* **1997**, *30*, 1022-1025.
(16) Armstrong, S.; Cook, W. J.; Short, S. A.; Ealick, S. E. Crystal structures of nucleoside 2-deoxyribosyltransferase in native and ligand-bound forms reveal architecture of the active site. *Structure* **1996**, *4*, 97-107.
(17) Emsley, P.; Cowtan, K. Coot: model-building tools for molecular graphics. *Acta Crystallogr D Biol Crystallogr* **2004**, *60*, 2126-2132.
(18) Murshudov, G. N.; Vagin, A. A.; Dodson, E. J. Refinement of macromolecular structures by the maximum-likelihood method. *Acta Crystallogr D Biol Crystallogr* **1997**, *53*, 240-255.
(19) Gaynor, J. W.; Brazier, J.; Cosstick, R. Synthesis of 3'- S-phosphorothiolate oligonucleotides for their potential use in RNA interference. *Nucleosides Nucleotides Nucleic Acids* **2007**, *26*, 709-712.
(20) Kawana, M.; Kuzuhara, H. General method for the synthesis of 2′-azido-2′,3′-dideoxynucleosides by the use of [1,2]-hydride shift and β-elimination reactions. *J. Chem. Soc., Perkin Trans. 1* **1992**, 469-478.
(21) McGee, D. P.; Vaughn-Settle, A.; Vargeese, C.; Zhai, Y. 2'-Amino-2'-deoxyuridine via an Intramolecular Cyclization of a Trichloroacetimidate. *J Org Chem* **1996**, *61*, 781-785.
